# Within-Host Stochastic Emergence Dynamics of Immune-Escape Mutants

**DOI:** 10.1371/journal.pcbi.1004149

**Published:** 2015-03-18

**Authors:** Matthew Hartfield, Samuel Alizon

**Affiliations:** Laboratoire MIVEGEC (UMR CNRS 5290, IRD 224, UM1, UM2), 911 avenue Agropolis, Montpellier, France; Imperial College London, UNITED KINGDOM

## Abstract

Predicting the emergence of new pathogenic strains is a key goal of evolutionary epidemiology. However, the majority of existing studies have focussed on emergence at the population level, and not within a host. In particular, the coexistence of pre-existing and mutated strains triggers a heightened immune response due to the larger total pathogen population; this feedback can smother mutated strains before they reach an ample size and establish. Here, we extend previous work for measuring emergence probabilities in non-equilibrium populations, to within-host models of acute infections. We create a mathematical model to investigate the emergence probability of a fitter strain if it mutates from a self-limiting strain that is guaranteed to go extinct in the long-term. We show that ongoing immune cell proliferation during the initial stages of infection causes a drastic reduction in the probability of emergence of mutated strains; we further outline how this effect can be accurately measured. Further analysis of the model shows that, in the short-term, mutant strains that enlarge their replication rate due to evolving an increased growth rate are more favoured than strains that suffer a lower immune-mediated death rate (‘immune tolerance’), as the latter does not completely evade ongoing immune proliferation due to inter-parasitic competition. We end by discussing the model in relation to within-host evolution of human pathogens (including HIV, hepatitis C virus, and cancer), and how ongoing immune growth can affect their evolutionary dynamics.

## Author Summary

The ongoing evolution of infectious diseases provides a constant health threat. This evolution can either result in the production of new pathogens, or new strains of existing pathogens that escape prevailing drug treatments or immune responses. The latter process, also known as immune escape, is a predominant reason for the persistence of several viruses, including HIV and hepatitis C virus (HCV), in their human host. As a consequence, the within-host emergence of new strains has been the intense focus of modelling studies. However, existing models have neglected important feedbacks that affects this emergence probability. Specifically, once a mutated pathogen arises that spreads more quickly than the initial (resident) strain, it potentially triggers a heightened immune response that can eliminate the mutated strain before it spreads. Our study outlines novel mathematical modelling techniques that accurately quantify how ongoing immune growth reduces the emergence probability of mutated pathogenic strains over the course of an infection. Analysis of this model suggests that, in order to enlarge its emergence probability, it is evolutionary beneficial for a mutated strain to increase its growth rate rather than tolerate immunity by having a lower immune-mediated death-rate. Our model can be readily applied to existing within-host data, as demonstrated with application to HIV, HCV, and cancer dynamics.

## Introduction

Parasites and pathogens pose a continuous threat to human, livestock, and plant health since new strains can readily emerge, via mutation or recombination, from pre-existing strains. Generally, the focus has been on detection of emerging diseases at the population level, in order to track and control their spread [1, 2]. Modelling approaches to predicting emergence have therefore primarily concentrated on detecting infections arising between individual hosts [3, 4], and the contribution of within-host processes to pathogen emergence has often been overlooked. It is now well known that within-host evolution has strong effects on the epidemiology of many pathogens (reviewed in [5]), and can substantially affect the course of an infection, as illustrated by the cases of HIV [6] and hepatits C virus (HCV) [7].

Each step of the within-host evolutionary dynamics consists of the emergence of a rare mutant that takes over the pathogen population. Since mutated infections always initially appear as a few copies, they are prone to extinction so their emergence is best captured using stochastic dynamics (as opposed to deterministic approaches). Only a handful of previous models have investigated this stochastic within-host process. A widespread use of within-host models in static populations is to calculate the probability that infections will evolve drug resistance, and what regime is needed to avoid treatment failure [8–11]. One exception [12] studied viral emergence within a host, if most mutations were deleterious, in order to determine how different viral replication mechanisms affected the establishment of beneficial alleles.

Parasite and pathogen evolution can radically affect the course of infections in hosts able to mount immune responses. For instance, HIV is known to successfully fix mutations within individuals which enable it to evade immune pressures [6]. This is in line with evidence that target cell limitation cannot account for HIV dynamics, and that immune limitation also needs to be present [13]. Arguably, this continual evasion of immunity prompts the chronic nature of HIV infections (see [14, 15] for an illustration of these ‘Red Queen’ dynamics). In the case of HCV, it has also been found that chronic infections were associated with higher rates of within-host evolution [16]. Concerning a different chronic disease, it is now well-known that the ability of malignant cells to rapidly expand in size, ignoring biological signals to arrest growth (e.g. through mutating the *p*53 tumour suppression gene), and escaping the patient’s immune system are key steps in cancer development [17]. All these scenarios can be analysed in the larger framework of evolutionary rescue [18, 19], where a change in the environment (in this case, the activation of an immune response) will cause the population to go extinct unless it evolves (develops an increased replicative ability, then subsequently rise to a large enough size to avoid stochastic loss). Although chronic infections are those most likely to evolve strains that evade immune pressures, evidence exists that such immune escape also plays a role in acute infections, like influenza [20]. Note that the latter evidence arises from serial passage experiments; although these tend to maximise selection at the within-host level, they still include a slight selective pressure for increased transmission.

Modelling mutant emergence in an immune evasion context raises a technical challenge, as it is a non-equilibrium process. In most of the within-host models presented above [8–10, 12], it is sufficient to know the initial state of the system to calculate emergence probability of immune or treatment escape. A non-equilibrium model was studied by Alexander and Bonhoeffer [11], who accounted for the reduction in available target cells when determining the emergence of drug resistance using an evolutionary rescue framework. With immune-escape however, the problem is that the immune state when the mutant infection appears is only temporary. In this case, there will be an initial strain present within the host, which triggers immunity. This strain can then mutate into a faster-replicating form, but if the mutated strain arises at a low frequency, immunity can destroy it before it has a chance to spread. This effect can be exacerbated by the fact that the mutated strain also prompts an increased immune response, so the emerging infection has a stronger defence to initially compete with (assuming immune growth is proportional to the total size of the pathogen population). This feedback, where increased immune growth prevents emergence of mutated strains with higher replication rates, can strongly affect the appearance of mutated strains within-host, and needs to be accounted for. Existing emergence models have not yet accounted for such within-host population feedbacks, especially those arising from the immune system.

Recently, Hartfield and Alizon [21] tackled a related problem, regarding how epidemiological feedbacks affect disease emergence at the host population level. In their model, a faster-replicating strain emerged via mutation from a pre-existing infection; however, the continuing outbreak caused by the initial strain removed susceptible individuals from the population, which limited the initial spread of the mutated strain. It was shown that the ongoing depletion of susceptible individuals due to the initial strain spreading has a stronger effect on reducing pathogen emergence, than assumed by just scaling down the reproductive ratio by the frequency of susceptible individuals present when the mutated infection appears. That is, the feedback produced by the first strain in removing susceptible individuals caused a drastic decrease in the emergence probability of the mutated strain.

Building on this previous study, we derive here an analytical approximation for the probability that an immune-escape mutant will emerge and maintain itself within a host. We use ‘immune-escape’ in the sense that while the mutated strain can be killed by immunity, it can ultimately outgrow immune growth and chronically persist. Furthermore, we use an acute infection setting, which are commonly used to study the within-host dynamics of ‘flu-like’ diseases [22–28]. Analysis of the model demonstrates how the ongoing proliferation of immune cells acts to decrease the emergence probability of mutated strains. Acute infection models are also useful in studying the first stages of chronic infections, where one observes exponential growth of the virus population, followed by a decline in the first weeks of infection. In addition, there are two questions that are worth investigating with this model from a biological standpoint. First, what is the fittest evolutionary strategy for an escape mutant: is it to overgrow the immune response (that is, increase its inherent replication rate to enable its persistence, even when immune cells are at capacity), or to tolerate it (prevent immunity from killing as many pathogens per immune cell)? Both these actions will lead to an increase in the pathogen’s net reproduction rate and can thus be described as immune evasion, but it is unclear if one process is favoured over the other. In addition, note that disabling the immune response is not possible, since the wild-type infection activates it. Second, to what extent do we need to account for the ongoing proliferation of the immune response? In other words, if we calculate the emergence probability based on the system state when the mutation occurs, how inaccurate would this estimate be? We end by discussing our results in the light of what is known about within-host evolution for several human infections.

## Materials and Methods

### Model outline

In order to find an analytical solution for the within-host emergence of a mutated strain, we follow the approach of Hartfield and Alizon [21], which investigated the appearance of a new infectious pathogen from a pre-existing strain at the population level. To consider the within-host case, we construct and subsequently analyse a specific scenario of acute, immunising infections. Here, the first strain will go extinct because it does not replicate at a high enough rate. However, before vanishing, it can mutate into a form that grows unboundedly; we are interested in calculating the probability of this event occurring. We focus on this case to cover a general range of within-host evolution scenarios, which is important since there is yet no consensus on how to best model within-host infections (reviewed in [29]). Although it is feasible that the mutated strain has a maximum population size, mathematical analysis of pathogen emergence only needs to consider the dynamics of mutants when they are present in a few copies, rather than the long-term behaviour once they have already established. Therefore, the general results outlined in this paper are also broadly applicable to cases where the infection population sizes are bounded.

Our analytical approach involves using a set of deterministic differential equations to ascertain pathogen spread in a stochastic birth-death process, where an infection (or immune cell) can only either die or produce 1 offspring. This process is one of the most common ways of investigating stochastic disease spread [30]. A list of nomenclature used in the model is outlined in Table 1. Assume there exists an initial pathogen, or infected cell-line, the size of which at time *t* is denoted *x*_1_(*t*). This line grows in size over time according to the following equation, which is well-used for within-host infection models [29]:

$$\begin{array}{c} \frac{\text{d}x_{1}}{\text{d}t}=x_{1}\left(\varphi_{1}-\sigma_{1}y\right) \end{array}$$ (1)

Here, *φ*_1_ is the growth rate of the infection, *σ*_1_ is the rate of destruction of the pathogen per immune cell, and *y*(*t*) is the number of immune cells (i.e. lymphocytes). For simplicity, we assume that there is complete cross-immunity between the various pathogen strains, so it is not necessary to model immune cell diversity.

**Table 1:** Glossary of notation.

Symbol	Usage
*t*	Time (measured as number of generation since start of process)
*φ*_1_, *φ*_2_	Growth rate of initial, mutated infection
*σ*_1_, *σ*_2_	Immune-mediated death-rate of initial, mutated infection
*x*_1_, *x*_2_	Size of initial, mutated infection
*x*_*init*_	Size of *x*_1_ at time *t* = 0 (or *y* = *y*_*init*_)
*y*	Size of immune response
*y*_*init*_	Initial size of immune response at *t* = 0
*K*	Maximum size of immune response
*r*	Unscaled growth rate of immune response
*R*_1_, *R*_2_	Reproductive ratio of original or mutated infection, *φ*_*i*_/*σ*_*i*_
R*	‘Effective’ initial reproductive ratio in the presence of immunity, *R* − *y*_0_
*ρ*	Scaled immune growth rate, *r*/*σ*_1_
*μ*	Individual mutation rate from first to second infection
Π	Emergence probability of second strain in an non-equilibrium population
*P*	Overall emergence probability of second strain
$P_{ext}^{*}$	Extinction probability, given an ‘effective’ reproductive ratio

The growth of the immune cell population is modelled using a logistic-growth curve:

$$\begin{array}{c} \frac{\text{d}y}{\text{d}t}=rx_{1}y\;\left(1-\frac{y}{K}\right) \end{array}$$ (2)

Here, *r* is the proliferation rate of immune cells, and *K* is the maximum population size they can achieve. This formulation is an extension of the model developed by Gilchrist and Sasaki [24] to study acute infections. The main difference in that in their model, the density of immune cells is allowed to reach any value in order to clear the infection. Here, we impose that immune density does not go above a maximum threshold *K*, which correspond to an intrinsic limitation in the host resources allocated to immunity.

Profile plots of typical infection responses are shown in Section 1 of S1 Text. If *φ*_1_/*σ*_1_ < *K*, then the first strain will increase in size until the immune-cells reach a maximum. After this point, the infection will decrease towards eventual extinction, while the immune response will be maintained at a non-zero size. However, if *φ*_1_/*σ*_1_ ≥ *K* then the first strain will continue to expand. A formal rational for this behaviour will be shown below.

To proceed with finding an analytical solution for the emergence probability, we proceed as in previous analyses [21, 24, 31], and note that since *y* is monotonically increasing, we can use the immune cell population size as a surrogate measure of time. By dividing Equation 1 by Equation 2, we obtain a differential equation for *x*_1_ as a function of *y*:

$$\begin{array}{c} \frac{\text{d}x_{1}}{\text{d}y}=\frac{\left(\varphi_{1}-\sigma_{1}y\right)x_{1}}{rx_{1}y\left(1-\frac{y}{K}\right)} \end{array}$$ (3)

To simplify subsequent analyses, we make the following substitutions. We define the reproductive rate of the infection, where there is a single immune cell (*y* = 1) equals *R*_1_ = *φ*_1_/*σ*_1_. We also set *ρ* = *r*/*σ*_1_ (this can be formally shown by rescaling time by *τ* = *σ*_1_ *t*). We use the notation *R*_1_ to draw parallels between the scaled pathogenic replication rate, and the reproductive ratio *R*_0_ in population-level, epidemiological models [32]. After making the required substitutions, Equation 3 can be rewritten as:

$$\begin{array}{c} \frac{\text{d}x_{1}}{\text{d}y}=\frac{K(R_{1}-y)}{\rho y(K-y)} \end{array}$$ (4)

Equation 4 formally shows that the infected cell line increases in size (d*x*_1_/d*y* > 0) if *y* < *R*_1_ = *φ*_1_/*σ*_1_, and decreases if *y* > *R*_1_. A corollary of this result is that if *R*_1_ of a infection exceeds *K*, then it cannot go extinct in the long term.

This differential equation is straightforward to solve (Section 1 of S1 Text), and yields the following function for *x*_1_(*y*):

$$\begin{array}{c} x_{1}\;\left(y\right)=x_{init}+\frac{1}{\rho }\left(\log\left[{\left(\frac{y}{y_{init}}\right)}^{R_{1}}\;{\left(\frac{K-y}{K-y_{init}}\right)}^{\left(K-R_{1}\right)}\right]\right) \end{array}$$ (5)

where *x*_*init*_ and *y*_*init*_ are, respectively, the number of pathogen and immune cells at the start of the process (time *t* = 0), and log is the natural logarithm. It is clear from Equation 4 that the maximum value of *x*_1_ occurs for *y* = *R*_1_. By substituting this value into Equation 5, we obtain the maximum value of *x*_1_ (denoted *x*_*M*_) as:

$$\begin{array}{c} x_{M}=x_{init}+\frac{1}{\rho }\left(\log\left[{\left(\frac{R_{1}}{y_{init}}\right)}^{R_{1}}\;{\left(\frac{K-R_{1}}{K-y_{init}}\right)}^{\left(K-R_{1}\right)}\right]\right) \end{array}$$ (6)

Note that *ρ* has no effect on the position of the peak (that is, the value of *y* leading to the maximal infection level). Since it affects the growth rate of the immune response (and therefore also of the pathogen), it does determine the maximum value itself. As this maximum is inversely proportional to *ρ*, smaller immune growth rates lead to larger peaks. Finally, the maximum infection time (as a function of *y*) needed for the first infection to go extinct can be determined by solving *x*_1_(*y*) = 0 numerically (Section 1 of S1 Text).

### Formulating emergence probability

Our goal is to calculate the probability of ‘evolutionary rescue’. That is, the initial strain has *R*_1_ < *K* so is guaranteed to go extinct in the long-term. However, a mutated form (with reproductive ratio *R*_2_) could arise with *R*_2_ > *K*, and if it does not go extinct when rare, it can outgrow immune proliferation. Previous theory on the emergence of novel pathogenic strains [21] showed that if mutated strains arise at rate *μ* per time step, the overall emergence probability *P* is given by:

$$\begin{array}{c} P=1-\exp\left(-\mu \int_{y_{init}}^{y_{M}}x_{1}\;\left(y\right)\Pi \left(y\right)\text{d}y\right) \end{array}$$ (7)

for *y*_*M*_ the maximum immune size for which it is possible for immune escape to arise, and Π(*y*) the emergence probability of an escape mutation were it to appear. The formulation of each of these will be discussed in turn.

#### Emergence probability, Π(*y*)

The emergence probability of the new strain has to account for both the increase in immune response before the mutation occurs, which reduces the ‘effective’ growth-rate of a mutated strain when it appears due to an heightened immune size (greater *y* in the model); and the continuing growth of immunity once it has appeared. Both these points factor in how the overall immune strength affects emergence probabilities, but this strength can increase over time. In theory, this emergence probability can be calculated from first principles using a time-inhomogeneous branching-process equation, which accounts for the birth and death of pathogens as the lymphocyte population size is changing (see [4, 21] for examples of branching-process formulations in epidemiology). However, due to the complexity of the model, specifically the non-linear form of the change in immune response (Equation 2), it is not possible to derive an exact analytical solution for Π (Section 1 of S1 Text).

We proceed by comparing a previously-found solution to Π in a changing susceptible population to our model, and adapting the result accordingly. When investigating a second strain emerging from a pre-existing epidemic, Hartfield and Alizon [21] found that a good approximation for Π is a function of the form:

$$\begin{array}{c} \Pi =\left(\frac{R^{✱}}{\rho \left(x_{1}+x_{2}\right)y\left(K-y\right)+R^{✱}}\right)\;\left(1-P_{ext}^{✱}\right) \end{array}$$ (8)

where R* is the ‘effective’ reproductive ratio of the mutated strain at the start of the process, which is dependent on the size of a secondary resource (e.g. susceptible individuals, immune cells); and $P_{ext}^{*}$ is the extinction probability in a birth-death model, given the ‘effective’ reproductive ratio is reduced depending on the frequency of the same secondary resource. Equation 8 arose during analysis of pathogen emergence in an epidemiological SIR framework [21], by noting how for a single strain, the ratio of the rate of change of infected and susceptible individuals over time reflected the emergence probability. This logic was then carried over to a two-strain scenario, so it details how the ongoing spread of the first pathogen affected emergence of mutated strains. To give an example, when applied to a model of pathogen emergence in an SIR setting, the effective reproductive ratio R* would equal the standard reproductive ratio *R*_0_, reduced by a factor *S*_0_/*N*, if there initially existed *S*_0_ susceptible individuals out of a total population of size *N*. There would also be a term in the denominator that is proportional to the rate of spread of the initial, unmutated strain.

However, in the current model, mutated pathogen lines have a reduced emergence probability due to ongoing production of immunity triggered by the first strain. The rational behind Equation 8 is that the *ρ*(*x*_1_ + *x*_2_)*y*(*K* − *y*) accounts for the reduction in emergence probability due to the continuing onset of immunity, given a total infection size (*x*_1_ + *x*_2_). If it reaches a steady-state [that is, *ρ*(*x*_1_ + *x*_2_)*y*(*K* − *y*) → 0] then Π collapses mathematically to $\left(1-P_{ext}^{*}\right)$. Hence Equation 8 accounts for how the presence of infection triggers immune responses that restrict the emergence probability of mutated infections; this is the feedback not yet considered in previous within-host evolution models.

We use Equation 8 in our model by setting R* = *R*_2_ − *y*_*init*_, which is the rescaled growth rate of the mutated strain, corrected for the fact that the baseline immunity rate will reduce its initial selective advantage. Note we use *y*_*init*_ instead of *y*(*t*) in this argument, since the R* term should describe the baseline replication rate if the second strain is only present. For the emergence probability, we first note that as with Equations 3 and 4, a single mutated cell has an effective birth rate of *R*_2_ and death rate of *y*. Standard results from birth-death models states that the mean growth rate is equal to *R*_2_ − *y*, with variance equal to *R*_2_ + *y* [33]. We therefore determine the baseline emergence probability by substituting these terms into the classic result by Feller [34]:

$$\begin{array}{c} \left(1-P_{ext}^{✱}\right)=1-\exp\left(-2\frac{\left(R_{2}-y\right)}{\left(R_{2}+y\right)}\right) \end{array}$$ (9)

Note that Equation 9 is not constant over time since it is a function of *y*. Overall, we obtain the following approximation for Π:

$$\begin{array}{c} \Pi \left(y\right)=\left(\frac{R_{2}-y_{init}}{\rho \left(x_{1}\left(y\right)+1\right)y\left(K-y\right)+R_{2}-y_{init}}\right)\;\left(1-\exp\left(-2\frac{\left(R_{2}-y\right)}{\left(R_{2}+y\right)}\right)\right) \end{array}$$ (10)

Also note that we use *x*_1_(*y*)+1 in the denominator, since the total pathogen population is accounted for by the initial pathogen size when the mutant appears, *x*_1_(*y*), plus a single mutated individual.

#### Maximum value of *y*, *y*_*M*_

The emergence of mutated infections can be arrested in one of two ways. Either the immune response becomes high enough so as to drive the first infection to extinction (*x*_1_(*y*) = 0), or so that any further emergence is impossible (Π(*y*) = 0). Therefore, *y*_*M*_ can be easily found by finding the smallest solution out of *x*_1_(*y*) = 0 or Π(*y*) = 0. These equations have to be solved numerically (Section 1 of S1 Text).

#### Example plots of *x*_1_ and Π

**Fig 1:**
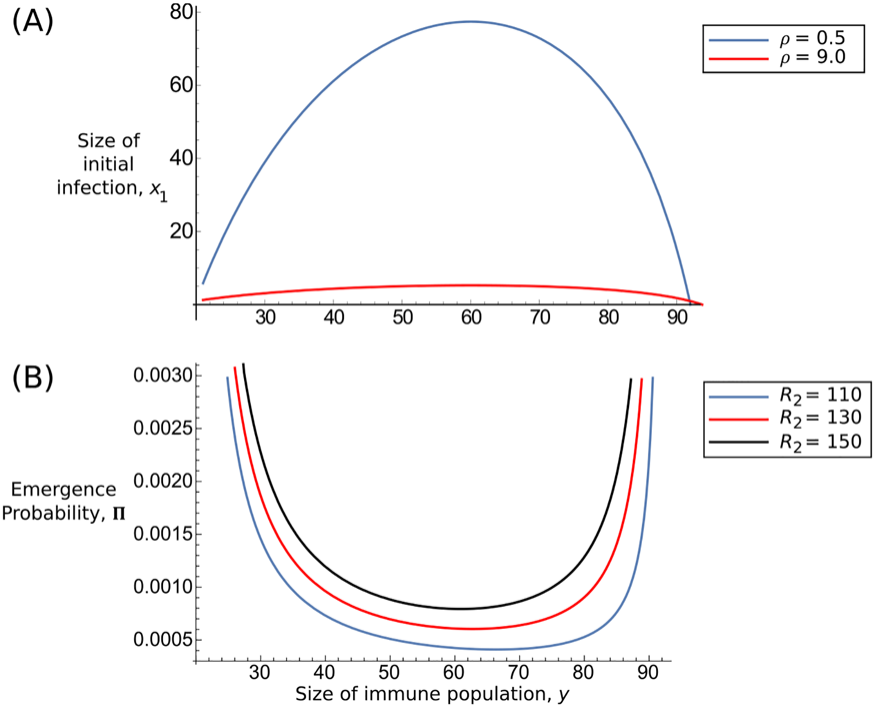
Sample behaviour of *x*_1_ and Π. Example plots of Equations 5 (A) and 10 (B), as a function of the immune-cell population *y*. Different line colours reflect either different values of *ρ* in (A), or *R*_2_ in (B). Other parameters are *K* = 100, *R*_1_ = 60, *x*_*init*_ = 1, *y*_*init*_ = 20.

Fig. 1 give example behaviour of both *x*_1_ and Π, as determined by Equations 5 and 10 respectively. *x*_1_ exhibits an inverted-U shape, with the maximum size decreasing if *ρ* increases, as expected from the form of Equation 5. From a biological standpoint, it illustrates the fact that the parasite population grows until the immune response is activated, at which point it decreases. Π, on the other hand, demonstrates a U shape. Emergence is initially high due to fewer immune cells being present, and again as the first strain dies out, since the immune population is not proliferating as fast and is less likely to remove mutated strains as they appear. Results are qualitatively similar for different parameter values, with Π further decreasing with higher values of *ρ* (Section 1 of S1 Text).

### Simulation methods

We verified our analytical solution by comparing it to simulation data. Simulations were written in C and based on the Gillespie Algorithm with tau-leaping [35, 36]; source code has been deposited online in the Dryad data depository (doi:10.5061/dryad.df1vk). The time step was set to be very low: Δ*τ* = 0.00005. This is because the tau-leaping algorithm is accurate only if the expected number of events per time step is small [37]; since the growth rates of the pathogen strains and the lymphocytes are both large, a small time step is needed to make the simulation valid.

The growth of both the original and mutated strains are simulated using scaled parameter rates. That is, the birth rate per time step for each pathogen is Poisson-distributed with mean (*R*_*i*_ ⋅ *x*_*i*_ ⋅ Δ*τ*), and death rate with mean (*x*_*i*_ ⋅ *y* ⋅ Δ*τ*) for *i* = 1, 2. This is done to reduce the number of parameters in the model, and also enables accurate comparison with the scaled results.

The change in size of the immune response is determined by standard logistic-growth dynamics for the Gillespie algorithm. That is, the Poisson mean number of births per time step equaled *ρ*_*b*_(*x*_1_ + *x*_2_)*y*, for *ρ*_*b*_ is the birth rate parameter, and the mean number of deaths equalled *y*(*x*_1_ + *x*_2_)(*ρ*_*d*_ + *ρ*(*y*/*K*)), where *ρ*_*d*_ is the death rate and *ρ* = *ρ*_*b*_ − *ρ*_*d*_. Since there are no distinct *ρ*_*b*_ and *ρ*_*d*_ terms in the model (just *ρ*), then we set *ρ*_*d*_ = 1 and varied *ρ*_*b*_, so *ρ* = *ρ*_*b*_ − 1. The net growth rate *ρ* was varied, generally between 0.5 and 19 depending on the size of the other parameters.

Note that in the analytical solution, it is assumed that the immune response does not die off. In order to maintain this assumption, we set *y*_*init*_ = 20 in the simulations. This also makes intuitive sense, because it is unlikely that the initial immune response is limited to just one cell. If the immune response goes extinct before both infected strains go extinct, or the mutated strain emerges, then that run is discarded, the simulation is reset and restarted.

In simulations, *R*_1_ is either set to 60 if *K* = 100, *R*_1_ = 100 if *K* = 250 or 1000, or *R*_1_ = 2,000 for *K* = 10,000. *R*_2_ was varied, ensuring that *R*_2_ > *K* so the infected strain can outgrow the immune response (see Equation 4). The mutation rate was also varied over several orders of magnitude. The first strain is reintroduced (from an initial frequency of 1 cell) 10,000,000 times as separate replication runs. Since the emergence probabilities were predicted to be low, a large number of runs were needed in order to produced a meaningful estimate of emergence probability. The second strain is said to have emerged once it exceeded 20 cells; since meaningful values of *R*_2_ were large, only a handful of cells would have been needed to guarantee emergence, hence a low outbreak threshold could be used [38].

## Results

### Comparison of analytical results with simulations data

**Fig 2:**
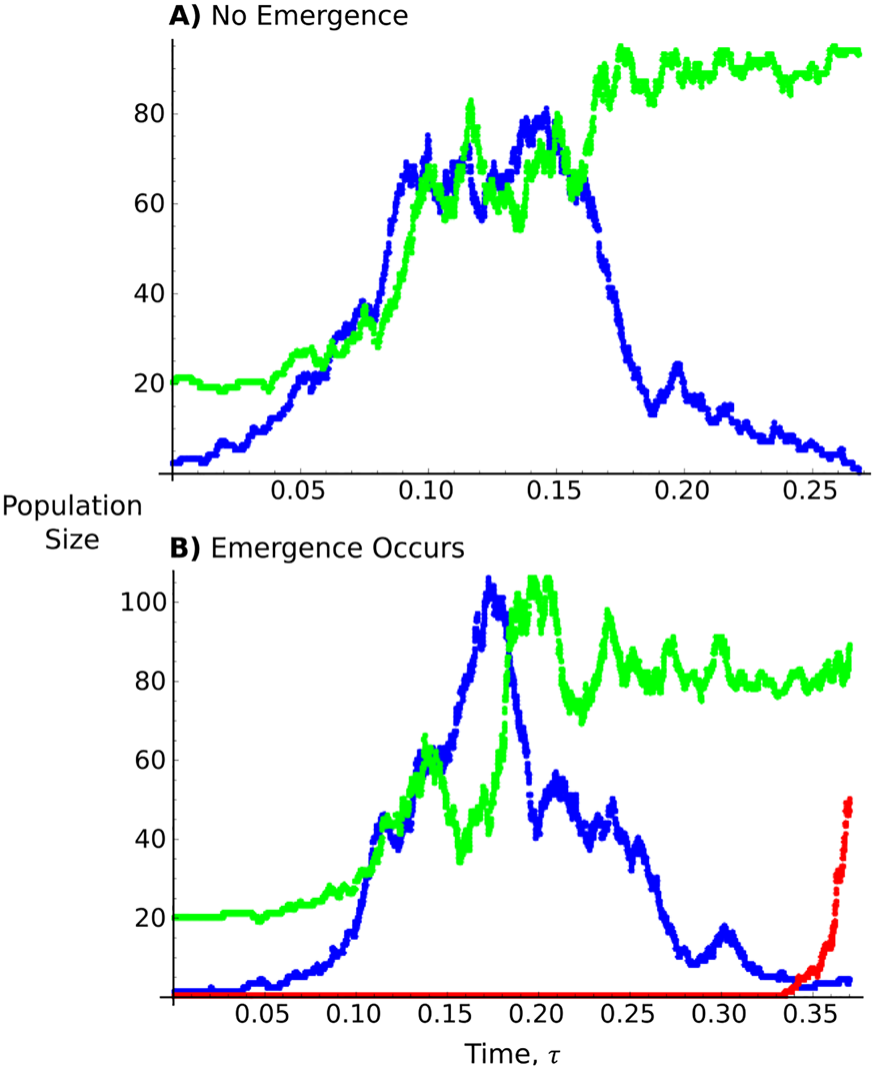
Example of simulation runs. Profiles of example simulation runs over time. In (A), the first strain goes extinct before a mutated pathogen arises, while in (B) emergence occurs. Blue dots represent the initial pathogen, red dots in (B) represent the mutated strain, while green dots show immune cell proliferation. The constant stream of red dots in (B) indicate the mutated strain at zero copies. Parameters are *K* = 100, *R*_1_ = 60, *R*_2_ = 200, *ρ* = 0.5, and *μ* = 0.01.

The first results we checked were individual profiles of the stochastic simulations, since these can be used to demonstrate the behaviour of within-host emergence. In the majority of cases, the first strain increase in size, but once immune proliferation also reaches its maximum then the first strain goes extinct soon after (Fig. 2(a)). However, emergence of the mutated strain can occur even if the immune cells are at their maximum size (Fig. 2(b)). This is to be expected, given the form of Π (Equation 10), which demonstrates that emergence is likeliest once the immune cells reach a steady-state, so ongoing proliferation does not restrict their establishment. Conversely, emergence probability is reduced if the immune response is spreading; this is because when the mutated infection is rare, it is less likely to reproduce as immune cells increase in number. Since the mutated infection population is only present in a few copies, this negative effect this immune growth has on reproductive ability will be drastic (see also equivalent population genetics results by Otto and Whitlock [39]).

**Fig 3:**
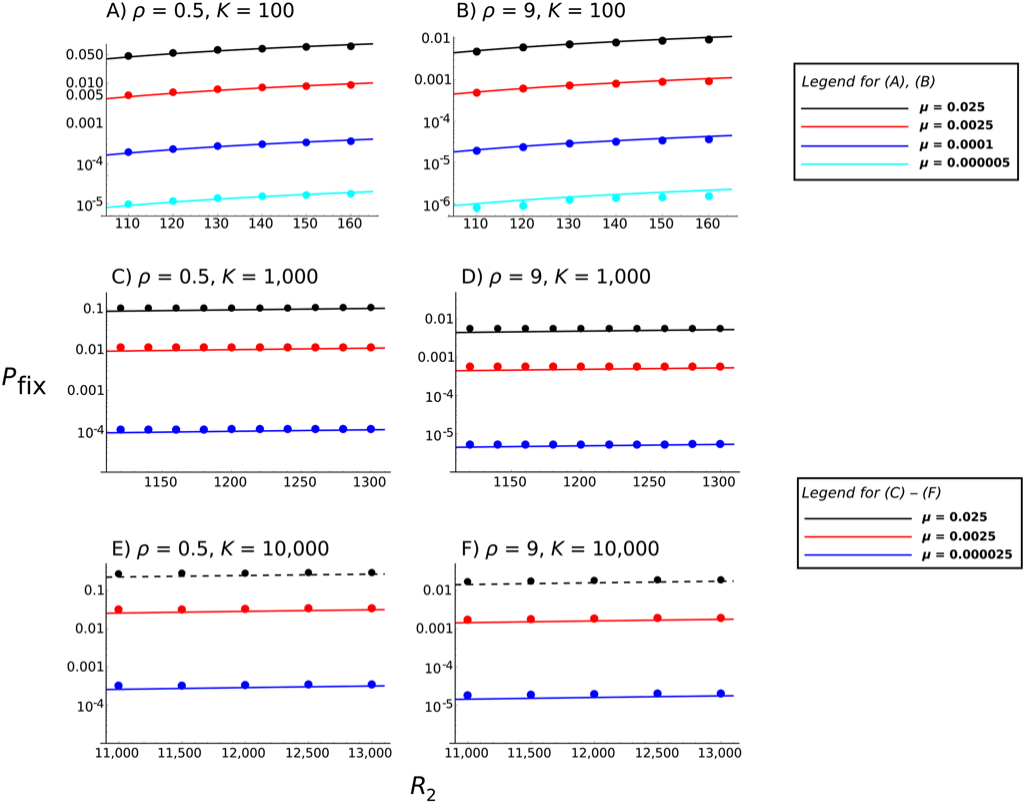
Comparison of simulations with analytical solutions. Comparisons of the full analytical solution (Equation 7, with Π given by Equation 10) with simulation results. Solid lines represent analytical solutions; points are simulation calculations. Graphs are plotted as a function of the reproductive ratio of the second strain, *R*_2_. Note that the *y* axis is plotted on a log scale. Different colours denote different mutation rates, as shown in the accompanying legend. Other parameters are (A and B) *K* = 100, *R*_1_ = 60; (C and D) *K* = 1,000, *R*_1_ = 100; or (E and F) *K* = 10,000, *R*_1_ = 2000. In all panels, *x*_*init*_ = 1 and *y*_*init*_ = 20. *ρ* equals either 0.5 (A, C, and E) or 9 (B, D, and F). All error bars, as calculated using binomial confidence intervals, lie within the points. Further results are shown in Section 2 of S1 Text.

Fig. 3 compares the full analytical solution (Equation 7, with Π given by Equation 10) against simulation data. If *K* = 100, *R*_1_ = 60, we see that there is an accurate overlap between the two for a variety of mutation rates that span several orders of magnitude (Fig. 3(a) and (b)). This match demonstrates how our analytical solutions can be used to accurately predict emergence probabilities in the face of different scenarios, including antibiotic resistance and tumour formation, as both these processes are characterised by high mutation rates.

We also tested a parameter set where the carrying capacity and initial growth rate was much higher (*K* = 1,000 and *R*_1_ = 100, or *K* = 10,000 and *R*_1_ = 2,000). Fig. 3(c)-(f) demonstrates that the analytical results slightly underestimate the simulation results to a small degree, especially if the mutated strain’s growth rate is high and *R*_2_ is close to *K*, but becomes more accurate as *R*_2_ increases and generally provides a good approximation. These inaccuracies probably arise due to our analytical solution not fully accounting for the increased variance in both infection and immune growth rates that can arise if parameters are large, as in this model [30]. However, the error does not appear to be great, so the model can still be used to provide accurate estimates of emergence probability for this parameter set.

Finally, we also tested how well analytical solutions work for cases where *R*_1_ < *R*_2_ < *K*. Although such a mutated strain replicates more quickly, Equation 4 shows that it will die out in the long-term. Hence our analytical solutions might not correctly reflect the emergence probability of these infections. Nevertheless, even in this case, Equation 7 accurately matches up with simulation results in this parameter range, although some inaccuracies arise for *K* = 1,000 (S1 Fig).

### Effect of ongoing immunity on emergence

**Fig 4:**
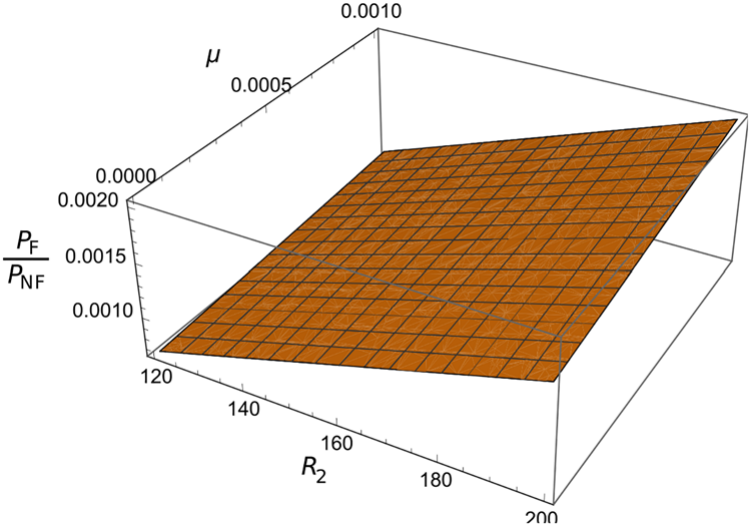
Quantifying effect of population-level feedbacks. 3D plots comparing the total emergence probability of a mutated strain with feedbacks (denoted *P*_*F*_), to one where feedbacks are not considered (denoted *P*_*NF*_), as a function of mutation rate *μ* and *R*_2_. Other parameters are *K* = 100, *R*_1_ = 60, *ρ* = 5, *x*_*init*_ = 1, *y*_*init*_ = 20.

To exemplify why it is important to account for ongoing immune growth, we compared our full solution for the emergence probability (Equation 7, with Π equal to Equation 10), to a ‘naive’ estimate that does not assume ongoing immune proliferation (Π = 1 − 1/*R*_2_). We see that our result leads to a greatly reduced emergence probability, with values from our model being ∼1,000 times lower than the naive estimate (Fig. 4, and Section 3 of S1 Text). Similar results apply if feedbacks only affect the mutant after it has arose (that is, $\Pi =(1-P_{ext}^{*})$ as given by Equation 9). Hence, as in previous non-equilibrium models [21], one needs to account for dynamical feedbacks, both before and after the mutated strain appears, in order to account for the reduced emergence probability in these scenarios.

### Comparing pathogen growth against death rate

We next studied what process has a larger effect on pathogen emergence. Immune escape can either be achieved by overgrowing the immune response (increasing the intrinsic pathogen growth rate *φ*), or by tolerating it (reducing the immune-mediated death rate *σ*). One might expect that the two processes would lead to similar increase in escape probability, since both affect the effective reproductive rate R*. However, this intuition need not hold in the face of immune-mediated feedbacks. We commence with a heuristic analysis based on the deterministic model to predict general behaviour for a newly-emerging strain, then use numerical analyses to check this reasoning.

If there are two strains spreading concurrently, the deterministic rate of change of immunity, *y*, and the second strain *x*_2_, is given by the following set of differential equations:

$$\begin{array}{ccc} \frac{\text{d}x_{2}}{\text{d}t} & = & x_{2}\left(\varphi_{2}-\sigma_{2}y\right) \end{array}$$ (11a)

$$\begin{array}{ccc} \frac{\text{d}y}{\text{d}t} & = & r\left(x_{1}+x_{2}\right)y\;\left(1-\frac{y}{K}\right) \end{array}$$ (11b)

Further recall that that the basic pathogen growth rate with only one immune cell is *R*_2_ = *φ*_2_/*σ*_2_. In order to augment its spread, the infection can either increase its intrinsic growth rate by a certain factor (i.e. change *φ*_2_ → *φ*_2_ *c*, where *c* > 1 is a numerical constant), or instead tolerate the immune response (mathematically equivalent to the transformation *σ*_2_ → *σ*_2_/*c*). We can investigate the effect of both these rescaled variables on the rate of change of pathogen spread by substituting them into Equation 4. After making the substitution *φ*_2_ → *φ*_2_ *c*, the pathogen rate of change becomes:

$$\begin{array}{c} \frac{\text{d}x_{2}}{\text{d}y}=\frac{K(cR_{2}-y)}{\rho y(K-y)}\left(\frac{x_{2}}{x_{1}+x_{2}}\right)\;\left(\frac{\sigma_{2}}{\sigma_{1}}\right) \end{array}$$ (12)

The *x*_2_/(*x*_1_ + *x*_2_) term arises due to the presence of the pre-existing initial strain *x*_1_, and the death-rate ratio *σ*_2_/*σ*_1_ appears since *ρ* was initially scaled by *σ*_1_; this term disappears if we assume equal death rates. Apart from these terms, Equation 12 is conceptually the same as Equation 4, but with *R* scaled by a factor *c*, as expected. However, if we instead scale *σ*_2_ → *σ*_2_ / *c*, a different result emerges:

$$\begin{array}{c} \frac{\text{d}x_{2}}{\text{d}y}=\frac{K(cR_{2}-y)}{c\rho y(K-y)}\left(\frac{x_{2}}{x_{1}+x_{2}}\right)\;\left(\frac{\sigma_{2}}{\sigma_{1}}\right) \end{array}$$ (13)

Here, not only is the reproductive ratio *R*_2_ scaled, but the immune growth term *ρ* is also altered. Mathematically, this is a simple consequence of the fact that the growth rate is scaled by 1/*σ*_1_, so any rescaling of *σ*_2_ also affects *ρ* through its effect on the *σ*_2_/*σ*_1_ ratio. Biologically, this outcome reflects the fact that a rescaling of immune-mediated death would not have the same effect on the growth rate than changing *φ*_2_ by the same ratio, since pathogen death is also a function of the immune population, *y* (see Equation 1). So although tolerating immunity might increase the infection growth rate, it comes at a cost of increasing the effective growth rate of immunity, as each immune cell would have a larger average impact on pathogen death.

Therefore, while a rise in growth rate (*φ*_2_) would only affect *R*_2_, reducing the death rate (*σ*_2_) comes at a cost of increasing the effective proliferation rate of immune cells. Hence, one expects that it is more advantageous for an infection to increase *φ*_2_ and outgrow the immune response, instead of reducing *σ*_2_ and tolerating it. Furthermore, note that the emergence probability Π in the presence of epidemiological feedbacks contains a term of order 1/*ρ* (as with d*x*_2_/d*y*), so the effective rise in immunity will further lead to a negative overall effect on emergence probability.

**Fig 5:**
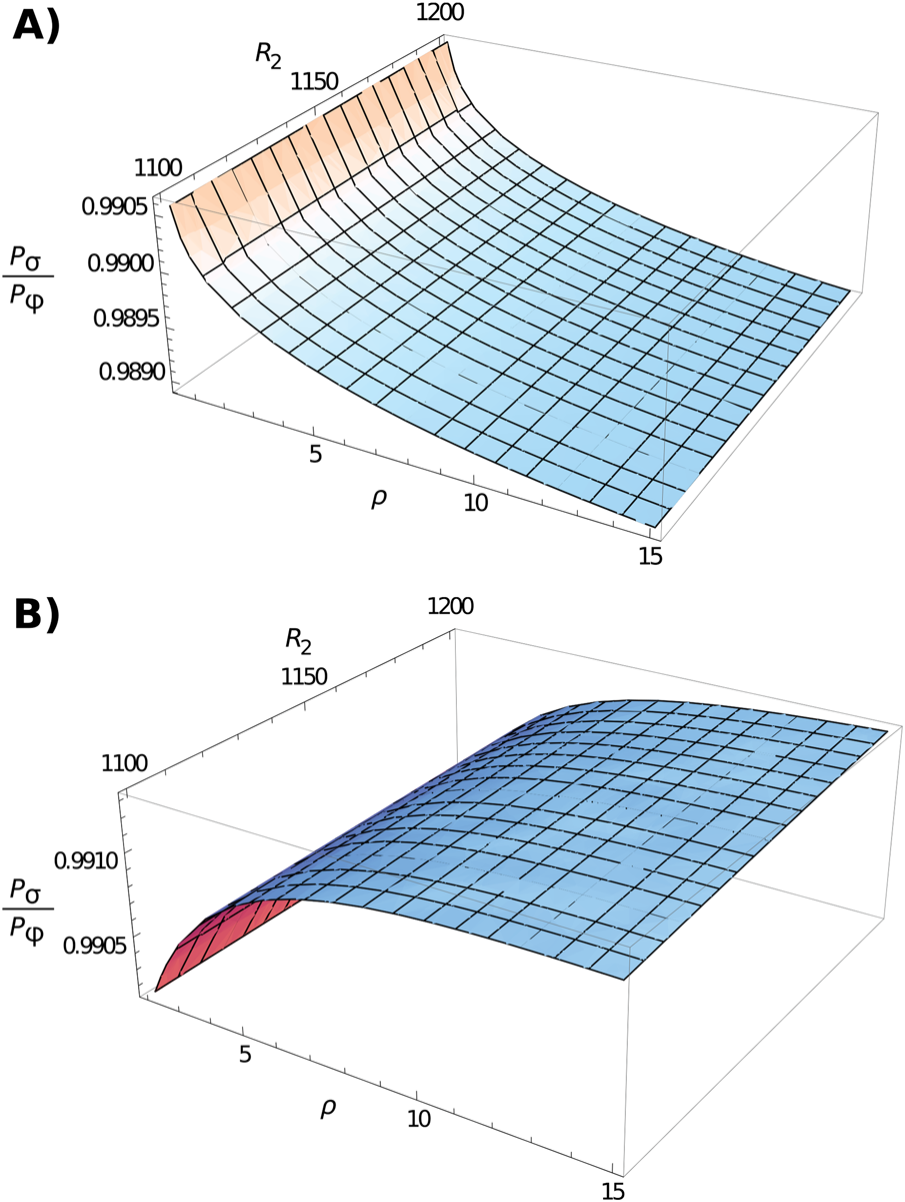
Comparing pathogen growth against death rate. 3D plot comparing the emergence probability if the death rate *σ* is reduced by a factor 1/*c* = 1.01 (here denoted *P*_*σ*_), to the case where the growth rate *φ* is increased by *c* = 1.01 (denoted *P*_*φ*_). Plot (A) is for the case where immune response d*y* / d*x* = *ρxy*(1 − *y*/*K*), as in our model, and (B) assumes d*y* / d*x* = *ρφxy*(1 − *y*/*K*) to enable comparison of our model with [27]. Other parameters are *K* = 1,000, *R* = 100, *x*_*init*_ = 1, *y*_*init*_ = 20, *μ* = 0.025 and *σ*_1_ = 1 for (B).

We verified this intuition by comparing the analytical results for cases where *φ*_2_ is increased by a set factor (so that only *R*_2_ is changed by this rescaling), to outcomes where *σ*_2_ is scaled (which will not just affect *R*_2_ but will also increase *ρ* by the same factor, as outlined above). Fig. 5(a) demonstrates how, for large parameter values, increasing *φ*_2_ only produces a higher overall emergence probability. Qualitatively similar results arise if using different parameter values.

#### Comparison with earlier results

These results appear to contradict a previous analysis by Alizon [27]. Using nested models, it was shown that in order to maximise the epidemiological (between-host) reproductive ratio *R*_0_, increasing the growth rate might not always be the best option for a pathogen because it shortens the duration of the infection.

There are several changes between the models that could explain the different outcomes. Principally, [27] aimed to determine the long-term *R*_0_ across a population, while our model concerns the emergence of new strains intra-host. In addition, [27] used deterministic equations, and did not investigate the stochastic emergence of new strains (that is, whether those with a smaller *R*_0_ are likely to emerge if they appear at a low frequency). However, one major difference that is worthy of further investigation concerns the different immune-response functions used in each model.

In [27], the model assumed that the dynamics of the immune cell population took the form d*y* / d*t* = *rφ* ^*b*^ *xy* (Equation 3 in that paper). Hence, the immune response was not just stimulated by the presence of an infection, but is also dependent on pathogen replication. The reason for this assumption is that the expression of pathogen peptides on the infected cell surface is strongly dependent on the replication activity [40]. This is why latent viruses, such as herpes virus, can go unnoticed from the immune system and persist for long periods of time. Interestingly, if *b* = 1 then, according to this form of d*y* / d*t*, overgrowing and tolerating the immune response will have equal effects on pathogen emergence. This can be seen by forming d*x*_2_ / d*y* as before, and after substituting either *φ*_2_ → *φ*_2_ *c* or *σ*_2_ → *σ*_2_/*c*, one sees that each transformation results in the same rescaled differential equation (Section 3 of S1 Text):

$$\begin{array}{c} \frac{\text{d}x_{2}}{\text{d}y}=\frac{K(cR_{2}-y)}{cR_{2}\sigma_{1}\rho y\left(K-y\right)}\left(\frac{x_{2}}{x_{1}+x_{2}}\right) \end{array}$$ (14)

Hence, if immunity is also linearly dependent on pathogenic growth, then increasing growth and reducing death will have equal effects on long-term emergence in a static population. However, due to the ongoing spread of immunity in our model, increasing immune tolerance might still cause the greatest negative impact on the emergence of mutated strains. This is due to its ensuing effect of increasing the ‘effective’ immune growth, which can restrict new strains from emerging (as reflected in the 1/*ρ* term in Π). This is indeed what is observed; an example of this behaviour is given in Fig. 5(b). Note that increasing *ρ* in this case leads to opposing behaviour than to what is seen for our model, in the sense that higher *ρ* reduces the advantage of increased *φ*_2_. Presumably, this is due to the *φ*_2_ term affecting the immune growth-rate, which can offset the reduction in emergence probability caused by the ongoing immune growth.

## Discussion

Since infectious diseases can exhibit high mutation rates, infectious diseases always pose a strong risk that they can evolve into new strains, especially ones that circumvent host defences. While this question has been extensively studied at the epidemiological level, the question of evolutionary emergence within hosts has received less scrutiny. Furthermore, few existing models have accounted for population dynamics feedbacks that can impact and restrict the emergence of mutated strains.

Here, we fill a key gap in existing modelling knowledge by deriving analytical methods for determining the emergence of new strains via within-host evolution. To this end, we tailor a broadly-applicable population dynamics model to the interaction between pathogens and immune cells, a system which share similarities with predator-prey models [29]. As with previous work studying feedbacks at the epidemiological level [21], we highlight how ongoing population spread (in this case, of immune cells) can strongly limit the emergence of new strains. This result is reflected in the fact that the emergence probability is greatly reduced due to ongoing immune proliferation (Equation 10); accounting for this feedback shows how it strongly restricts emergence of mutated strains (Section 3 of S1 Text).

After verifying the accuracy of the model, we subsequently determined whether it is more advantageous for a new infection to increase its growth rate (*φ*), or instead increase tolerance to the immune response (decrease *σ*), both of which will increase a pathogen’s reproductive ratio *R* and hence qualifies as immune escape phenomena. We demonstrate that, rather surprisingly, it always pays in the short-term for a pathogen to increase its replication rate, and to try and outgrow the immune response. The rationale behind this behaviour is that if immune tolerance rises by a certain factor, it will not cause an equal reduction on pathogen death since immunity is still present at a high frequency. Intuitively, the presence of multiple strains will cause indirect competition between them, since the presence of both causes a higher spread of immunity which reduces the mutated strain’s growth rate when rare. This phenomenon manifests itself mathematically as an increase in the scaled immune growth rate, *ρ*. Since increasing effective immunity would also reduce the emergence probability of new strains (Equation 10), it is evolutionary advantageous for pathogens to evolve a higher growth rate instead (Fig. 5).

One elegant outcome of the modelling framework is that these general outcomes should be robust to the function used to model immune-cell proliferation. It is a concern in epidemiological modelling that key results are heavily dependent on the specific mathematical functions used [41], and several models have been previously used to reflect immune growth [29]. However, most of these functions approximate to exponential growth when immunity initially appears; furthermore, the form of the emergence probability (Equation 8) makes clear that any immune growth will feedback onto the emergence of the mutated strain, limiting its appearance. For this reason, our conclusion that infectious diseases would benefit more from higher growth rates should also be robust to how immunity spreads over time.

There exists evidence, in line with these findings, that HIV increases its growth rate over the course of an infection [42]. However, the observed increase is small. A slight increase might only be necessary if too large a growth rate would trigger a larger immune response, or kill off the host too quickly and thus limit the epidemiological spread of the pathogen (both effects have been previously investigated by [27]). It has been proposed that immune escape for HIV becomes less efficient over time, which might be the cause of a low increase in replication rate [15]. Hence it might be mechanistically more feasible for a pathogen to instead evade the immune response, by mutating in or close to a virus epitope, so as to avoid recognition by lymphocytes that would otherwise neutralise it (a variant on the ‘immune tolerance’ scenario outlined above). These type of escape mutations have been widely documented in HIV infections, which is one reason why the virus is able to persist for long periods of time, and also why vaccination is so difficult [43, 44]. Similarly, although not a chronic illness, evidence exists demonstrating that influenza A/H1N1 evades immunity via evolution causing increased virus affinity to cell receptors, which enlarges its replication rate [20]. To further investigate this issue, future models need to be created that combine short-term effects of pathogen emergence, as used here, with long-term forecasts of the pathogenic steady-state (since our model only deals with the initial, emergence stage).

More generally, the huge challenge exerted by the immune system on pathogen populations *de facto* generates apparent competition between different infection strains (or even species) co-infecting the same host [45]. In this case, it has been postulated that the faster-replicating strain could persist in the long-term by sheer force of numbers [46], as predicted with our model. Several empirical studies exist that support this intuition, as observed with malaria [47, 48] and schistosomes [49] (although competition for another resource, such as red-blood cells for malaria infections, could have partly explained these results).

### Implications of the model for different cases of immune escape

Our model can also be used to shed light on different processes of within-host emergence that have been observed in clinical studies. Different diseases show varying outcomes with regards to the production of immune-escape mutations. Two extensively-studied human diseases, HIV and HCV, are both characterised by a successive emergence of new strains over time. In particular, HIV is well-known to produce ‘escape mutations’ that evade T cell-mediated immunity [43, 44]. On a within-host phylogeny, this behaviour is characterised by creation of new subclades [5–7]. This behaviour can be intuitively explained by noting that HIV has extremely high mutation rates, estimated at ∼0.2 errors made per replication cycle and the ability to produce 10^10^ – 10^12^ virions per day [6] (although mutation creation might be limited by a lower *N*_*e*_, which has been estimated to lie between 1,000 [50] and 100,000 [51]). Furthermore, a sizeable proportion of mutations are beneficial, with estimates of adaptive substitutions in the *env* gene placed at ∼55% [52] (although the actual proportion of spontaneous beneficial mutations will be less than this, as observed from mutagenesis studies with viruses [53, 54]). What may seem surprising is that given this evolutionary potential, we do not see a more pronounced increase in HIV replication rate throughout the course of an infection. Yet, conversely, immune escape is very strongly selected for [14]. One possibility could be that given the constrains on RNA virus genomes [55], evading the immune response might be easier to achieve than increasing the replication rate. Furthermore, if the maximum immunity population size is high, it could be too complicated mechanistically to outgrow it, rather than than simply evading it.

HCV also shows a similar propensity to produce immune-escape mutations, with an estimated substitution rate of 1.2×10^−4^ per replication cycle and 10^12^ virions generated each day [7] (although, as with HIV, the effective population size is greatly lower than this value [7]). Acute hepatitis C infections are characterised by little diversity accumulating over time, except in one specific genetic region (NS5B; [56]). Chronic infections accumulate much more diversity, in line with the hypothesis that they continuously evolve to evade the immune system [16]. These infections are also characterised by different immune profiles: acute infections lead to a high, sustained immune response, which tends to be greatly lowered in chronic infections [57]. Our model suggests that if the immune response naturally increased rapidly (i.e. a higher intrinsic *ρ* in the model), it can greatly limit the emergence of new pathogens as it rises, preventing immune escape and a chronic illness. There exists evidence that mutations in hosts correlate with infection outcome, mainly due to SNPs at the *IL28B* loci, which could cause this effect [58–60]. However, it remains controversial as to what determines virus clearance, especially since there exist evidence for virus control over infection outcome [61]. Therefore, the effect on immune response on the creation of escape mutations requires further study.

Other diseases are characterised by low probability of emergence despite frequent mutation, for which immune feedbacks could be the cause. Cancer growth is characterised by evading pre-programmed cell death and extremely rapid cell replication [17]. Furthermore, genomes present in cancer cells usually carry ‘mutator’ alleles, causing additional cell instability [62]. Therefore, cell mutation can be extremely common, but can generally be stopped by the immune response. Yet it is known that tumours can mutate immune-checkpoint networks to generate protection from the immune system. One prominent example is the up-regulation of ligands for the programmed cell death protein 1 (PD1) pathway, which can block antitumour immune responses [63]. Our model suggests that due to the rapid replication of tumour cells, they could also strongly trigger a heightened immune rate, the increased spread of which will greatly prevent cancer emergence. This mechanism could explain why tumour emergence is rare relative to the potential mutation rate.

### Overall summary

By accounting for the ongoing spread of immunity, we have quantified how this particular effect can feedback onto emergence of mutated pathogens intra-host, and inhibit the appearance of mutated strains. Further analysis of the model demonstrates how it is beneficial for a pathogen to increase its replication rate and attempt to outgrow immunity, as opposed to tolerate it. This model can therefore shed light on expected within-host evolutionary dynamics of infections, as well as determine why extremely rapidly replicating pathogens do not emerge as often as expected.

## Supporting Information

S1 Fig Simulation comparisons where *R*_1_ < *R*_2_ < *K*. Comparisons of the full analytical solution (Equation 7, with Π given by Equation 10) with simulation results. Solid lines represent analytical solutions; points are simulation calculations. Graphs are plotted as a function of the reproductive ratio of the second strain, *R*_2_. Note that the *y* axis is plotted on a log scale. Different colours denote different mutation rates, as shown in the accompanying legend. Other parameters are (A and B) *K* = 100, *R*_1_ = 60; (C and D) *K* = 1,000, *R*_1_ = 100; or (E and F) *K* = 10,000, *R*_1_ = 2000. In all panels, *x*_*init*_ = 1 and *y*_*init*_ = 20. *ρ* equals either 0.5 (A, C, and E) or 9 (B, D, and F). Most error bars, as calculated using binomial confidence intervals, lie within the points if they cannot be seen.
(EPS)

S1 Text Contains additional information on derivations, and further comparisons against simulation results (*Mathematica* NB format). The file is separated into three sections:

**Section 1 Setting up the mathematical model.** In-depth mathematical analyses of the differential equations used, and how to derive the emergence probability if affected by immune growth (Equation 10 in the main text).
**Section 2 Testing against stochastic simulations.** Full list of analytical comparisons with simulation outcomes, which contributed to Fig. 3.
**Section 3 Mathematical analysis of analytical solution.** Further outline of mathematical analysis and numerical computation, demonstrating (i) how immune feedbacks affect population growth, and (ii) how different *φ* and *σ* values affect mutated pathogen emergence.
(PDF)

S2 Text Same as Text S1, but in PDF format. (PDF)
