# Supplementary material for: Within-Host Stochastic Emergence Dynamics of Immune-Escape Mutants
Source: PLoS Comput Biol. 2015 Mar 18;11(3):e1004149. doi: 10.1371/journal.pcbi.1004149 (PMC4365036; doi:10.1371/journal.pcbi.1004149)
Supplement: S1 Text — The file is separated into three sections: Section 1 Setting up the mathematical model. In-depth mathematical analyses of the differential equations used, and how to derive the emergence probability if affected by immune growth (Equation 10 in the main text). Section 2 Testing against stochastic simulations. Full list of analytical comparisons with simulation outcomes, which contributed to Fig. 3. Section 3 Mathematical analysis of analytical solution. Further outline of mathematical analysis and numerical computation, demonstrating (i) how immune feedbacks affect population growth, and (ii) how different φ and σ values affect mutated pathogen emergence. (PDF) [file pcbi.1004149.s002.pdf]

Within-host emergence dynamics of immune-escape mutants

Matthew Hartfield & Samuel Alizon

Laboratoire MIVEGEC (UMR CNRS 5290, IRD 224, UM1, UM2), 911 avenue Agropolis, 34394 Montpellier Cedex 5, France.
Supplementary *Mathematica* File. Comments to matthew.hartfield@gmail.com.

Inputs

```
Clear["`*"];
Needs["ErrorBarPlots`"];
Needs["ErrorBarLogPlots`"]
```

Note that some of the plots below use the “ErrorBarLogPlots” package, which has to be downloaded separately (http://library.wolfram.com/infocenter/MathSource/6747/).

Supplementary Material S1: Setting up the mathematical model

Basic Model outline, numerical evaluation

In this problem there is an initial infected pathogen, or cell–line, the size of which at time *t* is denoted *x*[*t*]. This grows over time deterministically according to the following equation, which is well-used for within-host models (e.g. Alizon and van Baalen 2008):

$$\frac{dx}{dt} = x(\varphi - \sigma y)$$

Where φ is the growth rate, σ the death rate due to immunity, and y the density/population of immune cells. Immune growth is represesnted with a logistic-growth model given the presence of infection:

$$\frac{dy}{dt} = r\,x\,y\left(1 - \frac{y}{K}\right)$$

Here, *r* is the logistic growth rate of immunity, and *K* is the maximum/carrying capacity of the immune response.

In this model, if φ/σ < K (see below for a rational for this), then the first strain will increase in frequency until the immune-cells reach a critical size. After this point, the infection cells will decrease and go extinct, while the immune response will be maintained at a non-zero size. Below shows a numerical evaluation of this ODE set, with φ = 1, σ = r = 0.1, and K = 100. It is clear that with this example, the infection line goes extinct very quickly, after only 6-7 generations. This is because the maximum size

that the infection line can achieve is equal to φ/σ unless K is very small, which we will show below.

```
sol = NDSolve[{x'[t] == (1 - 0.1 y[t]) x[t],
   y'[t] == 0.1 x[t] y[t] (1 - y[t]/100), x[0] == 1, y[0] == 1}, {x, y}, {t, 0, 20}]
```

```
{{x → InterpolatingFunction[{{0., 20.}}, <>],
  y → InterpolatingFunction[{{0., 20.}}, <>]}}
```

```
Plot[Evaluate[{x[t], y[t]} /. sol], {t, 0, 10}]
```

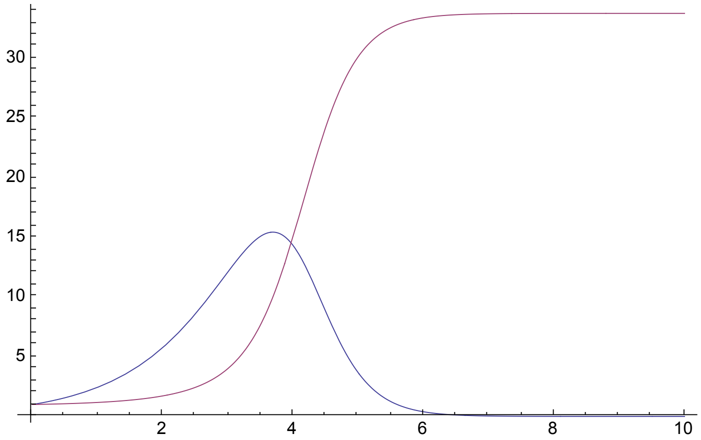

With φ/σ > K, then the parasite can escape immunity and rapildly increase in size. This is exemplified below with φ = 1, σ = r = 0.009 (so $\varphi/\sigma \simeq 111$).

```
sol2 = NDSolve[{x'[t] == (1 - 0.009 y[t]) x[t],
   y'[t] == 0.009 x[t] y[t] (1 - y[t]/100), x[0] == 1, y[0] == 1}, {x, y}, {t, 0, 20}]
```

```
{{x → InterpolatingFunction[{{0., 20.}}, <>],
  y → InterpolatingFunction[{{0., 20.}}, <>]}}
```

```
Plot[Evaluate[{x[t], y[t]} /. sol2], {t, 0, 10}]
```

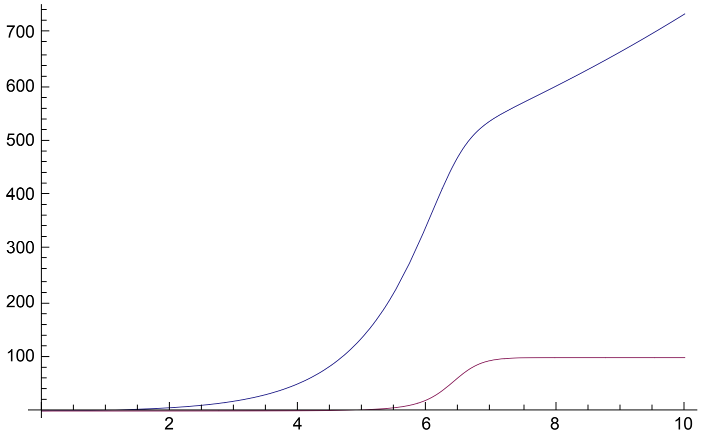

Solving differential equations, for use in potential analytical solution

To make sure we know the general behaviour of the system, as well as work out when realistic behaviour arises, we will now analyse the basic properties of the system to determine the maximum

growth rates, how to create analytical solutions for the infected cell-line, and so on. We start with the initial system of equations.

$$\frac{dx}{dt} == (\phi - \sigma y)\, x$$

$$\frac{dx}{dt} == x\, (-y\, \sigma + \phi)$$

$$\frac{dy}{dt} == r\, x\, y \left(1 - \frac{y}{Ka}\right)$$

$$\frac{dy}{dt} == r\, x\, y \left(1 - \frac{y}{Ka}\right)$$

We then follow the approach of Hartfied and Alizon (2014) by noting that since dy/dt is always ≥ 0, we can simplify this system by using the immune-cell population size as a proxy for time instead, and write x(y). In this case, dx/dy equals:

$$\frac{(\phi - \sigma y)\, x}{r\, x\, y \left(1 - \frac{y}{Ka}\right)} \text{ // FullSimplify}$$

$$\frac{Ka\, (-y\, \sigma + \phi)}{r\, (Ka - y)\, y}$$

To further simplify, we will make the following substitutions. The reproductive rate in the absence of immune response (or when it equals 1 since we assume it is never extinct) equals $R = \varphi/\sigma$. We can also reduce the number of parameters by setting $\rho = r/\sigma$ (this can be formally shown by rescaling time in the initial system by $\tau = \sigma t$). After making substitutions regarding R, ρ, we obtain:

$$\frac{Ka\, (-y\, \sigma + \phi)}{r\, (Ka - y)\, y} \text{ /. } \{\phi \to R\, \sigma\} \text{ // FullSimplify}$$

$$\frac{Ka\, (R - y)\, \sigma}{r\, (Ka - y)\, y}$$

$$\frac{Ka\, (R - y)\, \sigma}{r\, (Ka - y)\, y} \text{ /. } \{r \to \rho\, \sigma\} \text{ // FullSimplify}$$

$$\frac{Ka\, R - Ka\, y}{Ka\, y\, \rho - y^2\, \rho}$$

Or:

$$\frac{Ka\, (R - y)}{y\, \rho\, (Ka - y)} - \left\{\frac{Ka\, R - Ka\, y}{Ka\, y\, \rho - y^2\, \rho}\right\} \text{ // Simplify}$$

$$\{0\}$$

This gives us a simple differential equation that can be easily solved.

$$\text{DSolve}\left[\left\{x'[y] == \frac{Ka\, (R - y)}{y\, \rho\, (Ka - y)},\ x[y0] == x0\right\},\ x[y],\ y\right] \text{ // Simplify}$$

$$\left\{\left\{x[y] \to \frac{1}{\rho} (x0\, \rho + R\, \text{Log}[y] + (Ka - R)\, \text{Log}[-Ka + y] - R\, \text{Log}[y0] - Ka\, \text{Log}[-Ka + y0] + R\, \text{Log}[-Ka + y0])\right\}\right\}$$

```
D[1/ρ (x0 ρ + R Log[y] + (Ka - R) Log[-Ka + y] - R Log[y0] - Ka Log[-Ka + y0] + R Log[-Ka + y0]),
  y] // Simplify
```

$$\frac{\text{Ka R} - \text{Ka y}}{\text{Ka y } \rho - \text{y}^2 \rho}$$

$$\text{D}\left[\text{x0} + \frac{1}{\rho}\left(\text{Log}\left[\left(\frac{\text{y}}{\text{y0}}\right)^{\text{R}}\left(\frac{\text{Ka}-\text{y}}{\text{Ka}-\text{y0}}\right)^{\text{Ka-R}}\right]\right), \text{y}\right] \text{ // Simplify}$$

$$\frac{\text{Ka R} - \text{Ka y}}{\text{Ka y } \rho - \text{y}^2 \rho}$$

$$\text{Log}\left[\left(\frac{\text{y}}{\text{y0}}\right)^{\text{R}}\left(\frac{\text{Ka}-\text{y0}}{\text{Ka}-\text{y}}\right)^{\text{R}}\left(\frac{\text{Ka}-\text{y}}{\text{Ka}-\text{y0}}\right)^{\text{Ka}}\right]$$

$$\text{Log}\left[\left(\frac{\text{Ka}-\text{y}}{\text{Ka}-\text{y0}}\right)^{\text{Ka}}\left(\frac{\text{Ka}-\text{y0}}{\text{Ka}-\text{y}}\right)^{\text{R}}\left(\frac{\text{y}}{\text{y0}}\right)^{\text{R}}\right]$$

$$\frac{-\text{Ka}+\text{y}}{-\text{Ka}+\text{y0}} - \frac{\text{Ka}-\text{y}}{\text{Ka}-\text{y0}} \text{ // Simplify}$$

0

By combining the logarithm terms, we can write this solution in a much more compact form instead.

$$\text{FX3[Ka\_, R\_, }\rho\text{\_, x0\_, y0\_, y\_] := x0} + \frac{1}{\rho}\left(\text{Log}\left[\left(\frac{\text{y}}{\text{y0}}\right)^{\text{R}}\left(\frac{\text{Ka}-\text{y}}{\text{Ka}-\text{y0}}\right)^{\text{Ka-R}}\right]\right)$$

Now let's focus on the behaviour of these solutions. First, what is the maximum value of Y needed? Unfortunately, this cannot be found analytically, and using the simplified form of the solution (where Ka → infinity) will be inaccurate. But we can set up a function to find this numerically ('YMaxN' below).

$$\text{Solve}\left[\text{x0} + \frac{1}{\rho}\left(\text{Log}\left[\left(\frac{\text{y}}{\text{y0}}\right)^{\text{R}}\left(\frac{\text{Ka}-\text{y}}{\text{Ka}-\text{y0}}\right)^{\text{Ka-R}}\right]\right) == 0, \text{y}\right]$$

$$\text{Solve}\left[\text{x0} + \frac{\text{Log}\left[\left(\frac{\text{Ka-y}}{\text{Ka-y0}}\right)^{\text{Ka-R}}\left(\frac{\text{y}}{\text{y0}}\right)^{\text{R}}\right]}{\rho} == 0, \text{y}\right]$$

$$\text{YMaxN[Ka\_, R\_, }\rho\text{\_, x0\_, y0\_] := FindRoot}\left[\text{x0} + \frac{1}{\rho}\left(\text{Log}\left[\left(\frac{\text{y}}{\text{y0}}\right)^{\text{R}}\left(\frac{\text{Ka}-\text{y}}{\text{Ka}-\text{y0}}\right)^{\text{Ka-R}}\right]\right) == 0, \{\text{y, R}\}\right]$$

```
YMaxN[100, 50, 10, 1, 1]
{y → 99.1828}
```

Second question: When is the maximum X value obtained, and what parameters affects this value? From the form of the differential equation, it is clear that it is zero when y = R:

$$\text{Solve}\left[\frac{\text{Ka (R - y)}}{\text{y } \rho \text{ (Ka - y)}} == 0, \text{y}\right]$$

```
{{y → R}}
```

Thus the max value of *x* occurs for *y* = R. Interestingly enough, *ρ* has no effect on the *position* of the peak, although it clearly has some effect on growth rates if we run some numerical simulations of the system.

On the emergence probability, for a static and changing population

From the form of x'[y], it is clear that the first infection will increase in frequency if R-y > 0, where R is the reproductive ratio in the absence of immunity, $\frac{\phi}{\sigma}$. For this model to make sense, R needs to be high initially, since the pathogen dies out if the number of immune cells y > R. For example, if R = 10, then the initial extinction probability equals $\frac{1}{R}$= 0.1, quite low. But once y exceeds 10, extinction is certain.

```
Plot[y/10, {y, 1, 10}]
```

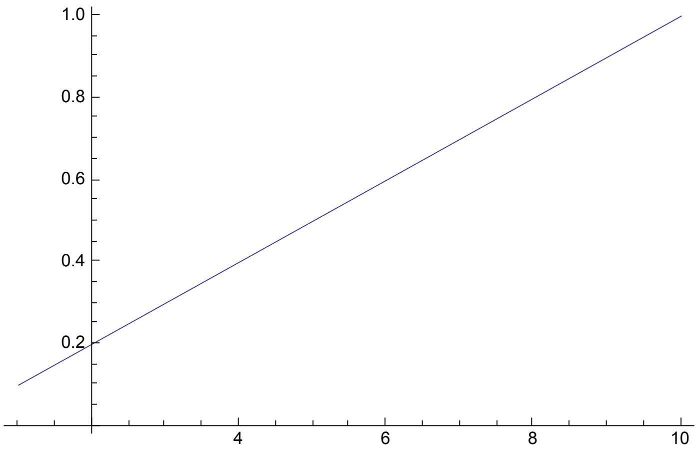

Therefore the same logic must apply with regards to emergence of the new pathogen. Say it has growth rate Φ and death rate (in absence of immunity) of Σ, then its baseline reproductive rate R2 = $\frac{\Phi}{\Sigma}$ must exceed *K* in order for it to have any chance of completely escaping immune proliferation.

The total emergence probability is given by the ‘evolutionary rescue’ equation, 1-Exp[-$\mu\int$x[y]Π[y]dy], where x is the population size of first cell-line and Π is the emergence probability. Preliminary results showed that using Π = 1-y/R2 greatly overestimated emergence probability compared to simulations, so we needed to account for the proliferation of the first parasite. We will first show how it is not possible to derive exact analytical solutions for Π, then how one can create an approximation based on previous solutions.

1) One-strain emergence equation (with x1 = 1). To try and find a solution, we’ll form a branching-process-type differential-equation that we will then solve. Let Q be the extinction probability. Then it might give birth a new cell with prob. ϕ, meanining there will be two cells that might eventially die out; the initial cell might die with prob σ y; or no birth or death will arise with prob (1-φ-σy). We can form an equation for dQ/dt based on this logic; furthermore, by dividing by dy/dt when x = 1, we further obtain an equation for dQ/dy.

$$\frac{\phi\,Q^2 + \sigma\,y - (\phi + \sigma\,y)\,Q}{r\,y\,\left(1 - \frac{y}{Ka}\right)} \text{ // Simplify}$$

$$\frac{Ka\,(-1 + Q)\,(-y\,\sigma + Q\,\phi)}{r\,(Ka - y)\,y}$$

Subbing in values for R, ρ:

```
Ka (-1 + Q) (-y σ + Q ϕ)
------------------------ /. {ϕ → R σ , r → ρ σ} // FullSimplify
     r (Ka - y) y
```

$$\frac{\mathrm{Ka}\,(-1+Q)\,(Q\,R-y)}{(\mathrm{Ka}-y)\,y\,\rho}$$

We can re-write this as:

```
Ka (1 - Q) (y - Q R)     Ka (-1 + Q) (Q R - y)
-------------------- - {---------------------} // Simplify
   (Ka - y) y ρ              (Ka - y) y ρ
```

$\{0\}$

If we assume that dQ/dy = 0, then we obtain classical solutions for the extinction probability.:

```
Solve[Ka (1 - Q) (y - Q R)
      -------------------- == 0, Q]
         (Ka - y) y ρ
```

$$\left\{\{Q\to 1\},\ \left\{Q\to \frac{y}{R}\right\}\right\}$$

But if not, we will try and solve the resulting differential equation...

```
DSolve[{-D[Q[y], y] == Ka (1 - Q[y]) (y - Q[y] R), Q[y0] == y0}, Q[y], y] // Simplify
                       --------------------------            --
                             (Ka - y) y ρ                    R
```

```
{{Q[y] → ((-Ka + y)^(R/ρ) (1 - y/Ka)^(Ka/ρ) (R - y0) y0^(R/ρ) (-Ka + y0)^(Ka/ρ)
      (1 - y0/Ka)^(R/ρ) Hypergeometric2F1[-R/ρ, (Ka - R + ρ)/ρ, 1 - R/ρ, y/Ka] + (1 - y/Ka)^(R/ρ)
      ((1 - y0/Ka)^(R/ρ) (-R (-Ka + y)^(R/ρ) y0^(R/ρ) (-Ka + y0)^(Ka/ρ) + (-Ka + y)^(R/ρ) y0^((R+ρ)/ρ) (-Ka + y0)^(Ka/ρ) +
          R y^(R/ρ) (-Ka + y)^(Ka/ρ) (-Ka + y0)^(R/ρ)) - y^(R/ρ) (-Ka + y)^(Ka/ρ) (R - y0) (-Ka + y0)^(R/ρ)
        (1 - y0/Ka)^(Ka/ρ) Hypergeometric2F1[-R/ρ, (Ka - R + ρ)/ρ, 1 - R/ρ, y0/Ka]))/
   ((-Ka + y)^(R/ρ) (1 - y/Ka)^(Ka/ρ) (R - y0) y0^(R/ρ) (-Ka + y0)^(Ka/ρ) (1 - y0/Ka)^(R/ρ)
      Hypergeometric2F1[-R/ρ, (Ka - R + ρ)/ρ, 1 - R/ρ, y/Ka] +
     y^(R/ρ) (-Ka + y)^(Ka/ρ) (1 - y/Ka)^(R/ρ) (-Ka + y0)^(R/ρ) (R (1 - y0/Ka)^(R/ρ) - (R - y0) (1 - y0/Ka)^(Ka/ρ)
        Hypergeometric2F1[-R/ρ, (Ka - R + ρ)/ρ, 1 - R/ρ, y0/Ka]))}} // FullSimplify
```

```
{{Q[y] →
   ((-Ka + y)^(R/ρ) (R - y0) y0^(R/ρ) (-Ka + y0)^(Ka/ρ) (-1 + Hypergeometric2F1[1, -Ka/ρ, 1 - R/ρ, y/Ka]) +
     y^(R/ρ) (-Ka + y)^(Ka/ρ) (-Ka + y0)^(R/ρ)
      (R + (-R + y0) Hypergeometric2F1[1, -Ka/ρ, 1 - R/ρ, y0/Ka]))/
   ((-Ka + y)^(R/ρ) (R - y0) y0^(R/ρ) (-Ka + y0)^(Ka/ρ) Hypergeometric2F1[1, -Ka/ρ, 1 - R/ρ, y/Ka] + y^(R/ρ)
      (-Ka + y)^(Ka/ρ) (-Ka + y0)^(R/ρ) (R + (-R + y0) Hypergeometric2F1[1, -Ka/ρ, 1 - R/ρ, y0/Ka]))}}
```

Clearly, this is a large solution and a bit of a mess!

```
Guess1[Ka_, R_, ρ_, y0_, y_] :=
 ((-Ka + y)^(R/ρ) (R - y0) y0^(R/ρ) (-Ka + y0)^(Ka/ρ) (-1 + Hypergeometric2F1[1, -Ka/ρ, 1 - R/ρ, y/Ka]) +
    y^(R/ρ) (-Ka + y)^(Ka/ρ) (-Ka + y0)^(R/ρ) (R + (-R + y0) Hypergeometric2F1[1, -Ka/ρ, 1 - R/ρ, y0/Ka]))/
  ((-Ka + y)^(R/ρ) (R - y0) y0^(R/ρ) (-Ka + y0)^(Ka/ρ) Hypergeometric2F1[1, -Ka/ρ, 1 - R/ρ, y/Ka] +
    y^(R/ρ) (-Ka + y)^(Ka/ρ) (-Ka + y0)^(R/ρ) (R + (-R + y0) Hypergeometric2F1[1, -Ka/ρ, 1 - R/ρ, y0/Ka]))
```

Furthermore, it does not seem to evaluate for realistic values, indicating some sort of complexity problem...

```
Plot[Guess1[1000, 30, 1, 20, y], {y, 21, 30}]
```

Infinity::indet : Indeterminate expression ComplexInfinity + ComplexInfinity encountered. >>

Infinity::indet : Indeterminate expression ComplexInfinity + ComplexInfinity encountered. >>

Infinity::indet : Indeterminate expression ComplexInfinity + ComplexInfinity encountered. >>

General::stop : Further output of Infinity::indet will be suppressed during this calculation. >>

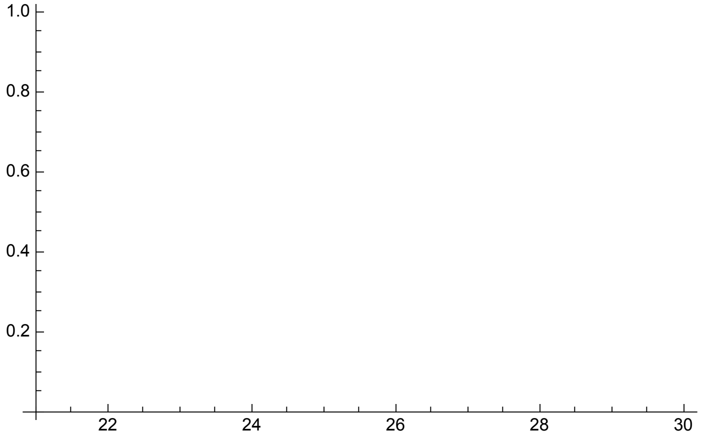

We decided that the best way to proceed was to create an estimate for Π, based on the results found in Hartfield and Alizon 2014. As explained in the main manuscript, we tried taking the emergence probability, (R2-y0)/R2, and add on a ρ(x1+1)y(1-y/K) term to the denominator, to account for immune growth. This term was then multiplied by the diffusion solution for emergence, $\left(1 - \text{Exp}\left[-\frac{2\,(\text{R2}-\text{y})}{\text{R2}+\text{y}}\right]\right)$, to create a scaled equation for the emergence probability.

```
YFixG1X[Ka_, R_, R2_, ρ_, x0_, y0_, y_] :=
  ((R2 - y0) / ((1 + FX3[Ka, R, ρ, x0, y0, y]) (Ka - y) y ρ + R2 - y0)) (1 - Exp[- 2 (R2 - y) / (R2 + y)])
```

Plotting this with known values below suggests that this is valid, up until a discontinuity is reached.

```
Plot[YFixG1X[100, 60, 120, 1, 1, 20, y], {y, 20, 92}]
```

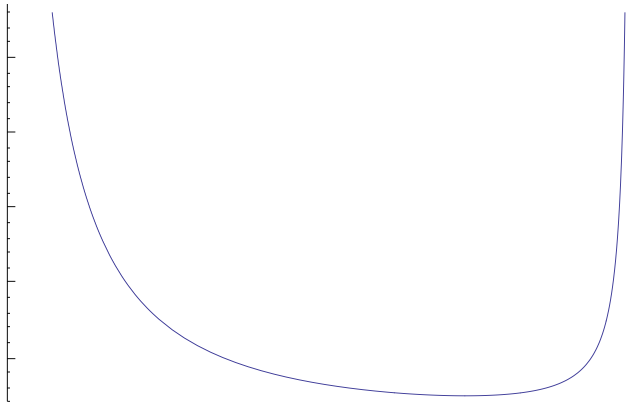

We can numerically find the y value causing this discontinuity, which represents the maximum possible y before emergence becomes inpermissable.

```
YMaxGN[Ka_, R_, R2_, ρ_, x0_, y0_] :=
 Floor[y /. FindRoot[(1 + FX3[Ka, R, ρ, x0, y0, y]) (Ka - y) y ρ + R2 - y0 == 0, {y, 3 R/2}]]
```

```
YMaxGN[100, 60, 120, 1, 1, 20]
```

```
92
```

Caution: If re-using this code, it is **imperative** to test whether the above function has a ‘good’ initial guess for finding the root of the denominator. Otherwise a different zero might be found, leading to inaccurate computation. For example, if the first guess at a root was set at 200 instead:

```
YMaxGN[Ka_, R_, R2_, ρ_, x0_, y0_] :=
 Floor[y /. FindRoot[(1 + FX3[Ka, R, ρ, x0, y0, y]) (Ka - y) y ρ + R2 - y0 == 0, {y, 200}]]
```

```
YMaxGN[100, 60, 120, 1, 1, 20]
```

```
106
```

Which is not correct.

Plots of Equations 5 and 10 (in the main text) for different parameter values

Equation 5 (dynamics of first strain as a function of immune size)

We first re-define the function x1(y) and the maximum value of y, for ease of plotting:

$$\text{FX3}[\text{Ka\_}, \text{R\_}, \rho\_, \text{x0\_}, \text{y0\_}, \text{y\_}] := \text{x0} + \frac{1}{\rho}\left(\text{Log}\left[\left(\frac{y}{\text{y0}}\right)^{R}\left(\frac{\text{Ka} - y}{\text{Ka} - \text{y0}}\right)^{\text{Ka}-R}\right]\right)$$

$$\text{YMaxN}[\text{Ka\_}, \text{R\_}, \rho\_, \text{x0\_}, \text{y0\_}] := y \text{ /. FindRoot}\left[\text{x0} + \frac{1}{\rho}\left(\text{Log}\left[\left(\frac{y}{\text{y0}}\right)^{R}\left(\frac{\text{Ka} - y}{\text{Ka} - \text{y0}}\right)^{\text{Ka}-R}\right]\right) == 0, \{y, R\}\right]$$

Below are plots for K = 100, R1 = 60, ρ = 0.5 (blue) and 9 (red).

```
a2 = Plot[{FX3[100, 60, 9, 1, 20, y]}, {y, 21, y /. YMaxN[100, 60, 9, 1, 20]},
   PlotRange → All, PlotStyle → {Red}, AxesOrigin → {0, 0}];
a1 = Plot[{FX3[100, 60, 0.5, 1, 20, y]}, {y, 21, y /. YMaxN[100, 60, 0.5, 1, 20]}];
Show[a1, a2]
```

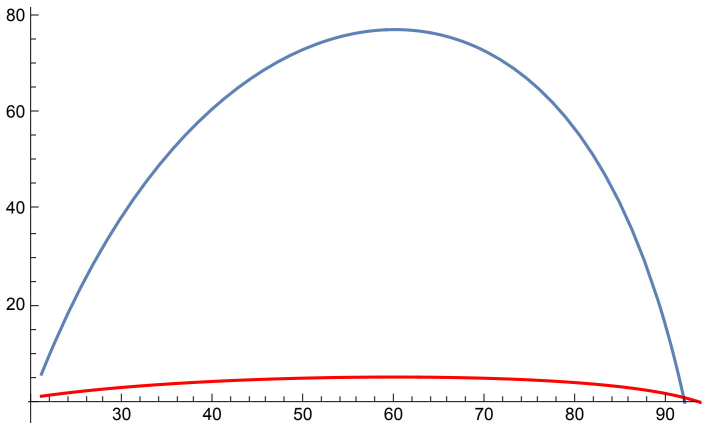

Below are plots for K = 1000, R1 = 100, ρ = 0.5 (blue) and 9 (red).

```
a2 = Plot[{FX3[1000, 100, 9, 1, 20, y]}, {y, 21, y /. YMaxN[1000, 100, 9, 1, 20]},
   PlotRange → All, PlotStyle → {Red}, AxesOrigin → {0, 0}];
a1 = Plot[{FX3[1000, 100, 0.5, 1, 20, y]},
   {y, 21, y /. YMaxN[1000, 100, 0.5, 1, 20]}, AxesOrigin → {0, 0}];
Show[
 a1,
 a2]
```

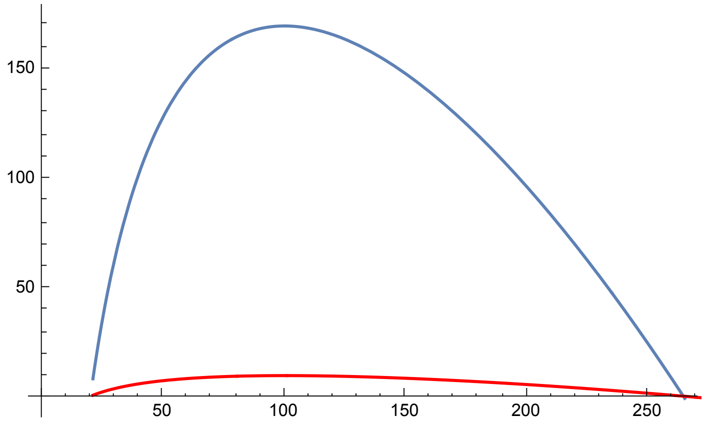

Below are plots for K = 10,000, R1 = 1000, ρ = 0.5 (blue) and 9 (red). We also slightly redefine the function to find the maximum permissible value of y to change the starting value for finding the root, otherwise inaccurate values would be obtained.

```
YMaxN[Ka_, R_, ρ_, x0_, y0_] :=
 y /. FindRoot[x0 + 1/ρ (Log[(y/y0)^R ((Ka - y)/(Ka - y0))^(Ka-R)]) == 0, {y, 3 * Ka/4}]
```

$$YMaxN[Ka\_, R\_, \rho\_, x0\_, y0\_] := y \,/.\, FindRoot\left[x0 + \frac{1}{\rho}\left(Log\left[\left(\frac{y}{y0}\right)^{R}\left(\frac{Ka - y}{Ka - y0}\right)^{Ka-R}\right]\right) == 0, \left\{y, \frac{3 * Ka}{4}\right\}\right]$$

```
a2 = Plot[{FX3[10 000, 1000, 9, 1, 20, y]}, {y, 21, YMaxN[10 000, 1000, 9, 1, 20]},
   PlotRange → All, PlotStyle → {Red}, AxesOrigin → {0, 0}];
a1 = Plot[{FX3[10 000, 1000, 0.5, 1, 20, y]},
   {y, 21, YMaxN[10 000, 1000, 0.5, 1, 20]}, AxesOrigin → {0, 0}];
Show[
 a1,
 a2]
```

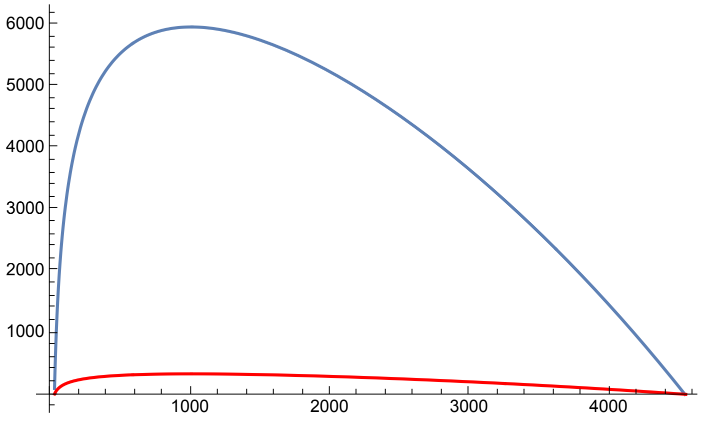

Equation 10 (dynamics of emergence probability Π as a function of immune size)

We first re-define the emergence probability Π:

```
YFixG1X[Ka_, R_, R2_, ρ_, x0_, y0_, y_] :=
 ((R2 - y0) / ((1 + FX3[Ka, R, ρ, x0, y0, y]) (Ka - y) y ρ + R2 - y0))
  (1 - Exp[- (2 (R2 - y))/(R2 + y)]) (* Mutated strain emergence, Π *)

YMaxGN[Ka_, R_, R2_, ρ_, x0_, y0_] :=
 Floor[y /. FindRoot[(1 + FX3[Ka, R, ρ, x0, y0, y]) (Ka - y) y ρ + R2 - y0 == 0, {y, (3 R)/2}]]
 (* Function to find when Π becomes inadmissable *)
```

Plots for K = 100, R1 = 60, ρ = 0.5, and R2 equals either 110, 130, or 150.

```
b1 = Plot[YFixG1X[100, 60, 110, 0.5, 1, 20, y],
    {y, 21, YMaxGN[100, 60, 110, 0.5, 1, 20]}];
b2 = Plot[YFixG1X[100, 60, 130, 0.5, 1, 20, y],
    {y, 21, YMaxGN[100, 60, 130, 0.5, 1, 20]}, PlotStyle → Red];
b3 = Plot[YFixG1X[100, 60, 150, 0.5, 1, 20, y],
    {y, 21, YMaxGN[100, 60, 150, 0.5, 1, 20]}, PlotStyle → Black];
Show[
 b1,
 b2,
 b3]
```

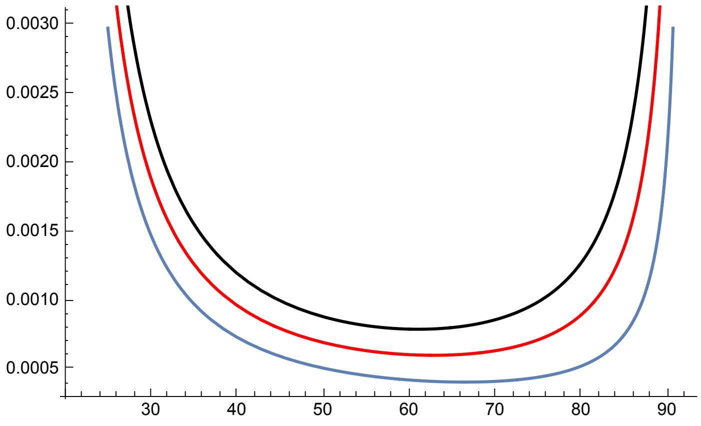

Plots for K = 100, R1 = 60, ρ = 9, and R2 equals either 110, 130, or 150.

```
b1 = Plot[YFixG1X[100, 60, 110, 9, 1, 20, y], {y, 21, YMaxGN[100, 60, 110, 9, 1, 20]}];
b2 = Plot[YFixG1X[100, 60, 130, 9, 1, 20, y],
   {y, 21, YMaxGN[100, 60, 130, 9, 1, 20]}, PlotStyle → Red];
b3 = Plot[YFixG1X[100, 60, 150, 9, 1, 20, y],
   {y, 21, YMaxGN[100, 60, 150, 9, 1, 20]}, PlotStyle → Black];
Show[
 b1,
 b2,
 b3]
```

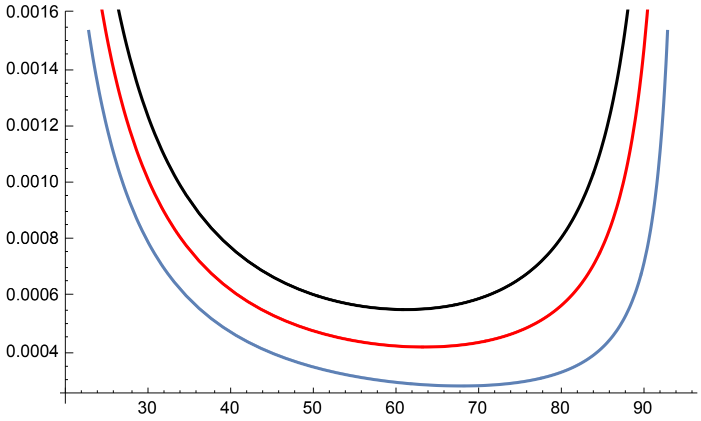

Redefining the function to find the maximum permissible value of y to change the starting value for finding the root.

```
YMaxGN[Ka_, R_, R2_, ρ_, x0_, y0_] :=
 Floor[y /. FindRoot[(1 + FX3[Ka, R, ρ, x0, y0, y]) (Ka - y) y ρ + R2 - y0 == 0, {y, 2 R}]]
 (* Function to find when Π becomes inadmissable *)
```

Plots for K = 1000, R1 = 100, ρ = 0.5, and R2 equals either 1150, 1250, or 1350.

```
b1 = Plot[YFixG1X[1000, 100, 1150, 0.5, 1, 20, y],
   {y, 21, YMaxGN[1000, 100, 1150, 0.5, 1, 20]}];
b2 = Plot[YFixG1X[1000, 100, 1250, 0.5, 1, 20, y],
   {y, 21, YMaxGN[1000, 100, 1250, 0.5, 1, 20]}, PlotStyle → Red];
b3 = Plot[YFixG1X[1000, 100, 1350, 0.5, 1, 20, y],
   {y, 21, YMaxGN[1000, 100, 1350, 0.5, 1, 20]}, PlotStyle → Black];
Show[
 b1,
 b2,
 b3]
```

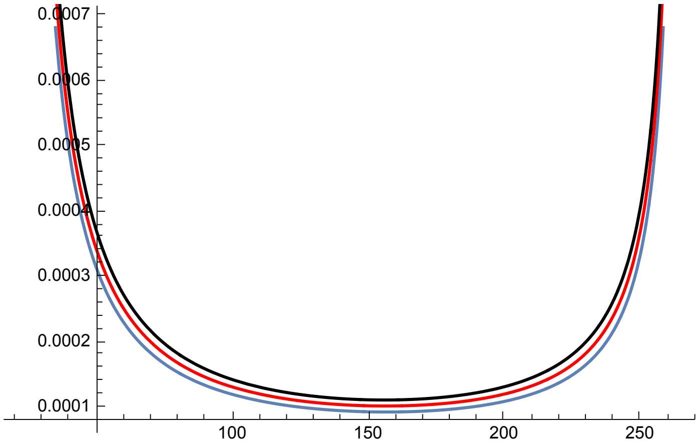

Plots for K = 1000, R1 = 100, ρ = 9, and R2 equals either 1150, 1250, or 1350.

```
b1 = Plot[YFixG1X[1000, 100, 1150, 9, 1, 20, y],
   {y, 21, YMaxGN[1000, 100, 1150, 9, 1, 20]}];
b2 = Plot[YFixG1X[1000, 100, 1250, 9, 1, 20, y],
   {y, 21, YMaxGN[1000, 100, 1250, 9, 1, 20]}, PlotStyle → Red];
b3 = Plot[YFixG1X[1000, 100, 1350, 9, 1, 20, y],
   {y, 21, YMaxGN[1000, 100, 1350, 9, 1, 20]}, PlotStyle → Black];
Show[
 b1,
 b2,
 b3]
```

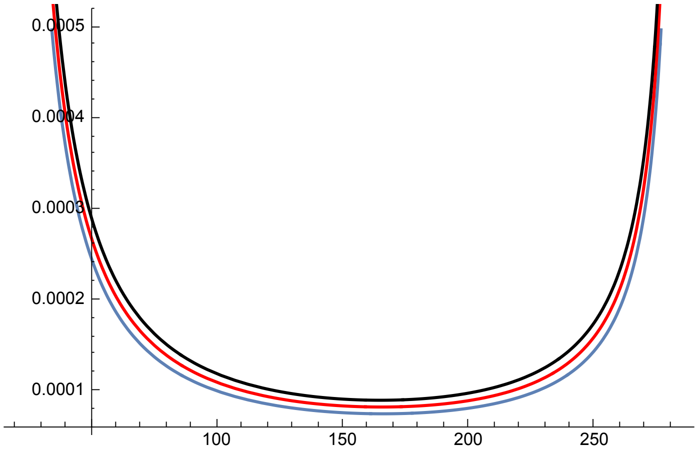

Redefining the function to find the maximum permissible value of y to change the starting value for finding the root.

```
YMaxGN[Ka_, R_, R2_, ρ_, x0_, y0_] :=
 Floor[y /. FindRoot[(1 + FX3[Ka, R, ρ, x0, y0, y]) (Ka - y) y ρ + R2 - y0 == 0, {y, Ka/2}]]
 (* Function to find when Π becomes inadmissable *)
```

Plots for K = 10,000, R1 = 1000, ρ = 0.5, and R2 equals either 11 150, 11 250, or 11 350.

```
b1 = Plot[YFixG1X[10 000, 1000, 11 500, 0.5, 1, 20, y],
   {y, 21, YMaxGN[10 000, 1000, 11 500, 0.5, 1, 20]}];
b2 = Plot[YFixG1X[10 000, 1000, 12 500, 0.5, 1, 20, y],
   {y, 21, YMaxGN[10 000, 1000, 12 500, 0.5, 1, 20]}, PlotStyle → Red];
b3 = Plot[YFixG1X[10 000, 1000, 13 500, 0.5, 1, 20, y],
   {y, 21, YMaxGN[10 000, 1000, 13 500, 0.5, 1, 20]}, PlotStyle → Black];
Show[
 b1,
 b2,
 b3]
```

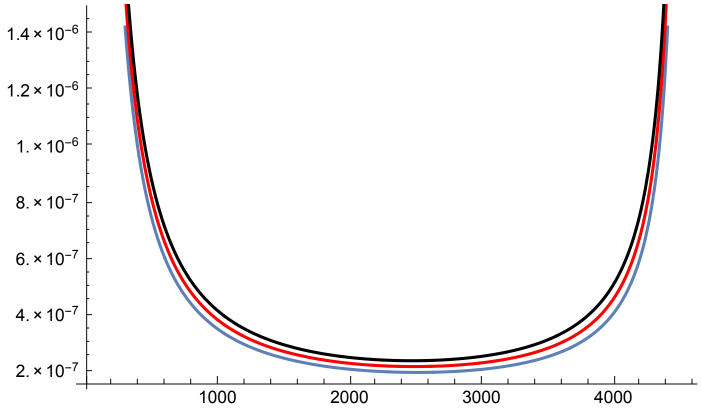

Plots for K = 10,000, R1 = 1000, ρ = 9, and R2 equals either 11 150, 11 250, or 11 350.

```
b1 = Plot[YFixG1X[10 000, 1000, 11 500, 9, 1, 20, y],
    {y, 21, YMaxGN[10 000, 1000, 11 500, 9, 1, 20]}];
b2 = Plot[YFixG1X[10 000, 1000, 12 500, 9, 1, 20, y],
    {y, 21, YMaxGN[10 000, 1000, 12 500, 9, 1, 20]}, PlotStyle → Red];
b3 = Plot[YFixG1X[10 000, 1000, 13 500, 9, 1, 20, y],
    {y, 21, YMaxGN[10 000, 1000, 13 500, 9, 1, 20]}, PlotStyle → Black];
Show[
 b1,
 b2,
 b3]
```

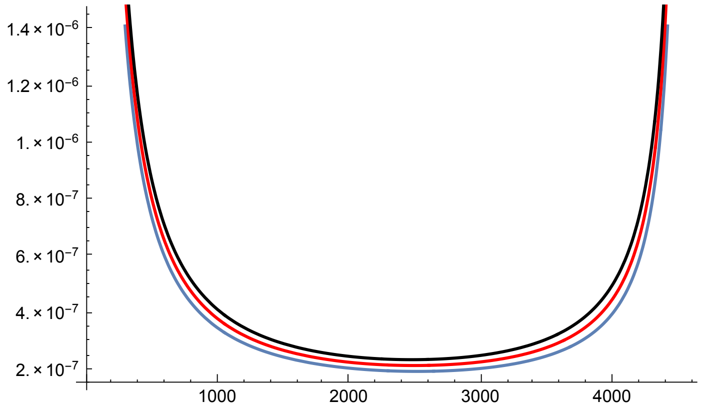

Supplementary Material S2: Testing against stochastic simulations

Function Definitions

First we list all the relevant equations needed for the computation, so they can be easily reloaded. Note that some of the plots below use the “ErrorBarLogPlots” package, which has to be downloaded separately (http://library.wolfram.com/infocenter/MathSource/6747/).

```
FX3[Ka_, R_, ρ_, x0_, y0_, y_] :=
 x0 + 1/ρ (Log[(y/y0)^R ((Ka - y)/(Ka - y0))^(Ka-R)]) (* First pathogen size x as a function of y *)

YFixG1X[Ka_, R_, R2_, ρ_, x0_, y0_, y_] :=
 ((R2 - y0) / ((1 + FX3[Ka, R, ρ, x0, y0, y]) (Ka - y) y ρ + R2 - y0))
  (1 - Exp[- (2 (R2 - y))/(R2 + y)]) (* Mutated strain emergence, Π *)

YMaxGN[Ka_, R_, R2_, ρ_, x0_, y0_] :=
 Floor[y /. FindRoot[(1 + FX3[Ka, R, ρ, x0, y0, y]) (Ka - y) y ρ + R2 - y0 == 0, {y, (3 * R)/2}]]
 (* Function to find when Π becomes inadmissable *)

YMaxN[Ka_, R_, ρ_, x0_, y0_] :=
 y /. FindRoot[x0 + 1/ρ (Log[(y/y0)^R ((Ka - y)/(Ka - y0))^(Ka-R)]) == 0, {y, 2 * R}]
  (* Function to find at what 'time' (immune size) when x becomes extinct *)
```

```
YMaxG2[Ka_, R_, R2_, ρ_, x0_, y0_] :=
 Min[YMaxGN[Ka, R, R2, ρ, x0, y0], YMaxN[Ka, R, ρ, x0, y0]]
 (* Calculating what event happens first - emergence becomes impossible,
 or first parasite becomes extinct *)
```

```
PEmerB[Ka_, R_, R2_, ρ_, x0_, y0_, μ_, yM_] := 1 - Exp[-μ *
    NIntegrate[FX3[Ka, R, ρ, x0, y0, y] * YFixG1X[Ka, R, R2, ρ, x0, y0, y], {y, y0, yM}]]
(* Calculating escape probability by integrating over entire
 timespan of first parasite infection *)
```

```
PEmerB2[Ka_, R_, R2_, ρ_, x0_, y0_, μ_] :=
 (1 - Exp[-2 (R - y0)/(R + y0)]) * PEmerB[Ka, R, R2, ρ, x0, y0, μ, YMaxG2[Ka, R, R2, ρ, x0, y0]]
(* Scaling down escape probability by emergence probability of first strain,
to ensure comparison with stochastic simulations *)
```

K = 100 simulations

Below commands load simulation output files used in analysis.

```
dataA = Import["results28thA.dat", "Table"];
(* μ = 0.001, 0.0001, 0.00001, 0.000005 *)
dataB = Import["results31st.dat", "Table"]; (* μ = 0.025, 0.005, 0.0025 *)
dataD = Import["results_2ndDec.dat", "Table"];
```

$\rho = 0.5$

$\mu = 0.001$

```
data1 = Transpose[Select[dataA, #[[4]] == 1.5 && #[[8]] == 0.001 &]];
data1X = Transpose[Select[dataD, #[[4]] == 1.5 && #[[8]] == 0.001 &]];
pts1 = Transpose[{data1[[3]], data1[[10]]}];
err1 = data1[[11]];
pts1X = Transpose[{data1X[[3]], data1X[[10]]}];
err1X = data1X[[11]];
p1 = Show[LogPlot[
   {PEmerB2[100, 60, R2, 0.5, 1, 20, 0.001]}, {R2, 105, 165},
   PlotRange → {Automatic, {0.000005, 0.01}},
   PlotStyle → {Black, Dashed}, AxesLabel → {"R2", "Pfix"}],
  ErrorListLogPlot[{{pts1X[[1]], ErrorBar[err1X[[1]]]},
    {pts1[[1]], ErrorBar[err1[[1]]]}, {pts1X[[2]], ErrorBar[err1X[[2]]]},
    {pts1[[2]], ErrorBar[err1[[2]]]}, {pts1[[3]], ErrorBar[err1[[3]]]},
    {pts1X[[3]], ErrorBar[err1X[[3]]]}},
   PlotStyle → {Black, PointSize[0.02]}], PlotRange → All
 ]
```

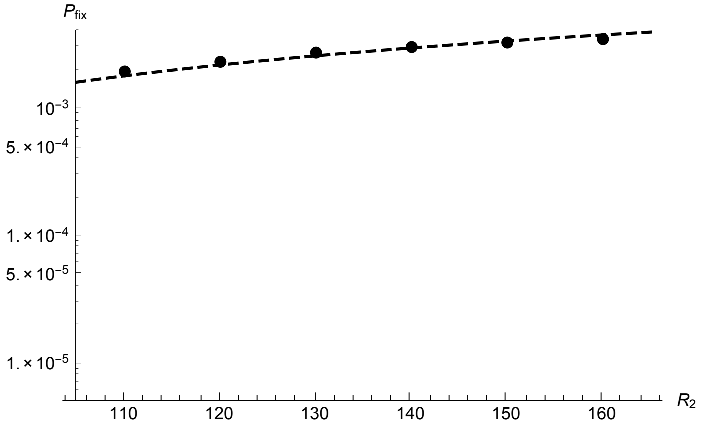

$\mu = 0.0001$

```
data1 = Transpose[Select[dataA, #[[4]] == 1.5 && #[[8]] == 0.0001 &]];
pts1 = Transpose[{data1[[3]], data1[[10]]}];
err1 = data1[[11]];
data1X = Transpose[Select[dataD, #[[4]] == 1.5 && #[[8]] == 0.0001 &]];
pts1X = Transpose[{data1X[[3]], data1X[[10]]}];
err1X = data1X[[11]];
p2 = Show[LogPlot[
    {PEmerB2[100, 60, R2, 0.5, 1, 20, 0.0001]}, {R2, 105, 165},
    PlotRange → {Automatic, {0.000005, 0.01}},
    PlotStyle → {Red}, AxesLabel → {"R2", "Pfix"}],
  ErrorListLogPlot[{{pts1X[[1]], ErrorBar[err1X[[1]]]},
     {pts1[[1]], ErrorBar[err1[[1]]]}, {pts1X[[2]], ErrorBar[err1X[[2]]]},
     {pts1[[2]], ErrorBar[err1[[2]]]}, {pts1X[[3]], ErrorBar[err1X[[3]]]},
     {pts1[[3]], ErrorBar[err1[[3]]]}},
    PlotStyle → {Red, PointSize[0.02]}], PlotRange → All
 ]
p2X = Show[Plot[
     {PEmerB2[100, 60, R2, 0.5, 1, 20, 0.0001]}, {R2, 105, 165},
     PlotRange → Automatic, PlotStyle → {Red}, AxesLabel → {"R2", "Pfix"}],
    ErrorListPlot[
     {{pts1X[[1]], ErrorBar[err1X[[1]]]}, {pts1[[1]], ErrorBar[err1[[1]]]},
      {pts1X[[2]], ErrorBar[err1X[[2]]]}, {pts1[[2]], ErrorBar[err1[[2]]]},
      {pts1X[[3]], ErrorBar[err1X[[3]]]}, {pts1[[3]], ErrorBar[err1[[3]]]}},
     PlotStyle → {Red, PointSize[0.02]}], PlotRange → All
  ];
```

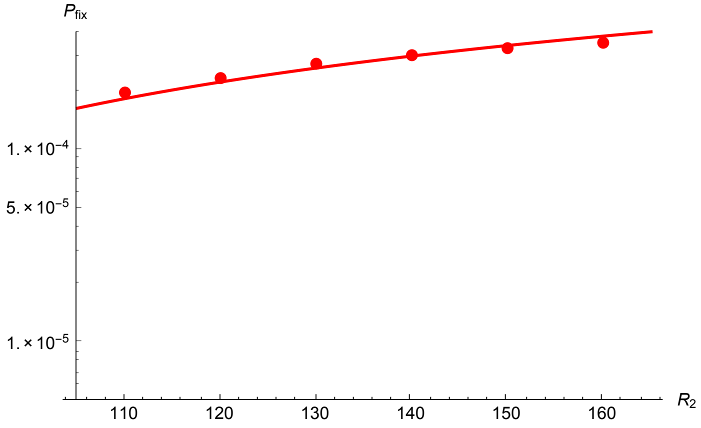

$\mu = 0.00001$

```
data1 = Transpose[Select[dataA, #[[4]] == 1.5 && #[[8]] == 0.00001 &]];
pts1 = Transpose[{data1[[3]], data1[[10]]}];
err1 = data1[[11]];
data1X = Transpose[Select[dataD, #[[4]] == 1.5 && #[[8]] == 0.00001 &]];
pts1X = Transpose[{data1X[[3]], data1X[[10]]}];
err1X = data1X[[11]];
p3 = Show[LogPlot[
   {PEmerB2[100, 60, R2, 0.5, 1, 20, 0.00001]}, {R2, 105, 165},
   PlotRange → {Automatic, {0.000005, 0.01}},
   PlotStyle → {Blue}, AxesLabel → {"R2", "Pfix"}],
  ErrorListLogPlot[{{pts1X[[1]], ErrorBar[err1X[[1]]]},
    {pts1[[1]], ErrorBar[err1[[1]]]}, {pts1X[[2]], ErrorBar[err1X[[2]]]},
    {pts1[[2]], ErrorBar[err1[[2]]]}, {pts1X[[3]], ErrorBar[err1X[[3]]]},
    {pts1[[3]], ErrorBar[err1[[3]]]}},
   PlotStyle → {Blue, PointSize[0.02]}], PlotRange → All
 ]
p3X = Show[Plot[
    {PEmerB2[100, 60, R2, 0.5, 1, 20, 0.00001]}, {R2, 105, 165},
    PlotRange → Automatic, PlotStyle → {Blue}, AxesLabel → {"R2", "Pfix"}],
   ErrorListPlot[
    {{pts1X[[1]], ErrorBar[err1X[[1]]]}, {pts1[[1]], ErrorBar[err1[[1]]]},
     {pts1X[[2]], ErrorBar[err1X[[2]]]}, {pts1[[2]], ErrorBar[err1[[2]]]},
     {pts1X[[3]], ErrorBar[err1X[[3]]]}, {pts1[[3]], ErrorBar[err1[[3]]]}},
    PlotStyle → {Blue, PointSize[0.02]}], PlotRange → All
  ];
```

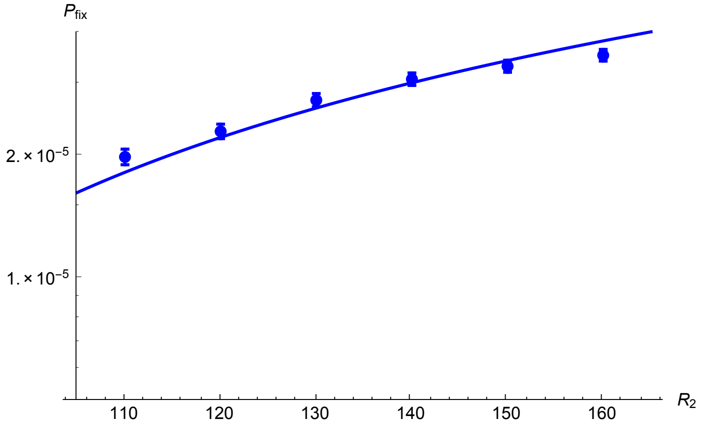

μ = 0.000005

```
data1 = Transpose[Select[dataA, #[[4]] == 1.5 && #[[8]] == 0.000005 &]];
pts1 = Transpose[{data1[[3]], data1[[10]]}];
err1 = data1[[11]];
data1X = Transpose[Select[dataD, #[[4]] == 1.5 && #[[8]] == 0.000005 &]];
pts1X = Transpose[{data1X[[3]], data1X[[10]]}];
err1X = data1X[[11]];
p4 = Show[LogPlot[
   {PEmerB2[100, 60, R2, 0.5, 1, 20, 0.000005]}, {R2, 105, 165},
   PlotRange → {Automatic, {0.000005, 0.01}},
   PlotStyle → {Green}, AxesLabel → {"R2", "Pfix"}],
  ErrorListLogPlot[{{pts1X[[1]], ErrorBar[err1X[[1]]]},
    {pts1[[1]], ErrorBar[err1[[1]]]}, {pts1X[[2]], ErrorBar[err1X[[2]]]},
    {pts1[[2]], ErrorBar[err1[[2]]]}, {pts1X[[3]], ErrorBar[err1X[[3]]]},
    {pts1[[3]], ErrorBar[err1[[3]]]}},
   PlotStyle → {Green, PointSize[0.02]}], PlotRange → All
 ]
p4X = Show[Plot[
    {PEmerB2[100, 60, R2, 0.5, 1, 20, 0.000005]}, {R2, 105, 165},
    PlotRange → Automatic, PlotStyle → {Green}, AxesLabel → {"R2", "Pfix"}],
   ErrorListPlot[
    {{pts1X[[1]], ErrorBar[err1X[[1]]]}, {pts1[[1]], ErrorBar[err1[[1]]]},
     {pts1X[[2]], ErrorBar[err1X[[2]]]}, {pts1[[2]], ErrorBar[err1[[2]]]},
     {pts1X[[3]], ErrorBar[err1X[[3]]]}, {pts1[[3]], ErrorBar[err1[[3]]]}},
    PlotStyle → {Green, PointSize[0.02]}], PlotRange → All
  ];
```

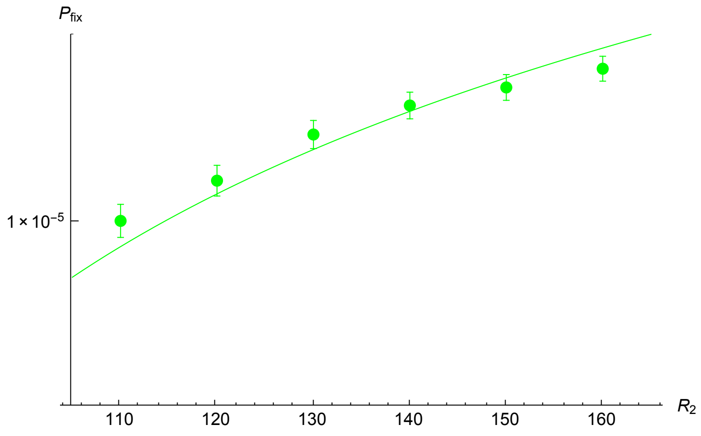

All μ together:

```
Show[p1, p2, p3, p4, PlotRange → All]
```

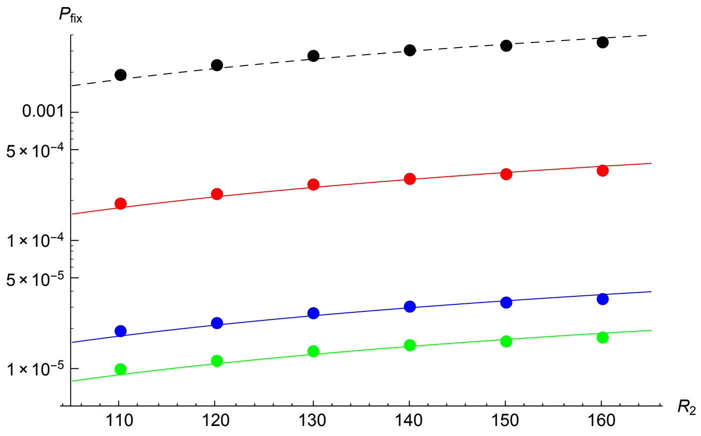


```
Show[p1X, p2X, p3X, p4X]
```

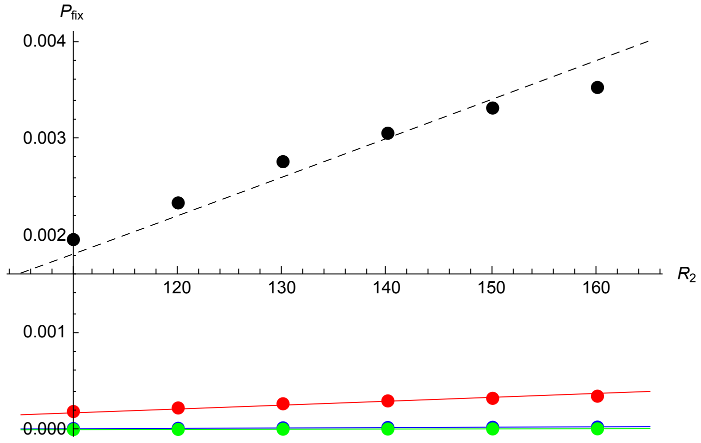

$\mu = 0.025$

```
data1 = Transpose[Select[dataB, #[[4]] == 1.5 && #[[8]] == 0.025 &]];
pts1  = Transpose[{data1[[3]], data1[[10]]}];
err1 = data1[[11]];
data1X = Transpose[Select[dataD, #[[4]] == 1.5 && #[[8]] == 0.025 &]];
pts1X  = Transpose[{data1X[[3]], data1X[[10]]}];
err1X = data1X[[11]];
p5 = Show[LogPlot[
    {PEmerB2[100, 60, R2, 0.5, 1, 20, 0.025]}, {R2, 105, 165},
    PlotRange → {Automatic, {0.000005, 0.5}},
    PlotStyle → {Magenta}, AxesLabel → {"R2", "Pfix"}],
  ErrorListLogPlot[{{pts1X[[1]], ErrorBar[err1X[[1]]]},
     {pts1[[1]], ErrorBar[err1[[1]]]}, {pts1X[[2]], ErrorBar[err1X[[2]]]},
     {pts1[[2]], ErrorBar[err1[[2]]]}, {pts1X[[3]], ErrorBar[err1X[[3]]]},
     {pts1[[3]], ErrorBar[err1[[3]]]}},
    PlotStyle → {Magenta, PointSize[0.02]}, PlotRange → All
 ]
p5X = Show[Plot[
     {PEmerB2[100, 60, R2, 0.5, 1, 20, 0.025]}, {R2, 115, 205},
     PlotRange → Automatic, PlotStyle → {Magenta}, AxesLabel → {"R2", "Pfix"}],
    ErrorListPlot[{{pts1[[1]], ErrorBar[err1[[1]]]},
      {pts1[[2]], ErrorBar[err1[[2]]]}, {pts1[[3]], ErrorBar[err1[[3]]]},
      {pts1[[4]], ErrorBar[err1[[4]]]}, {pts1[[5]], ErrorBar[err1[[5]]]}},
     PlotStyle → {Magenta, PointSize[0.02]}, PlotRange → All
  ];
```

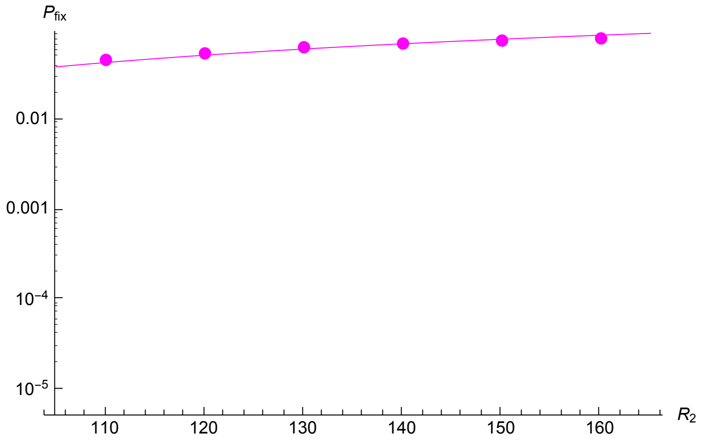

$\mu = 0.005$

```
data1 = Transpose[Select[dataB, #[[4]] == 1.5 && #[[8]] == 0.005 &]];
pts1  = Transpose[{data1[[3]], data1[[10]]}];
err1 = data1[[11]];
data1X = Transpose[Select[dataD, #[[4]] == 1.5 && #[[8]] == 0.005 &]];
pts1X  = Transpose[{data1X[[3]], data1X[[10]]}];
err1X = data1X[[11]];
p6 = Show[LogPlot[
    {PEmerB2[100, 60, R2, 0.5, 1, 20, 0.005]}, {R2, 105, 165},
    PlotRange → {Automatic, {0.000005, 0.5}},
    PlotStyle → {Orange}, AxesLabel → {"R2", "Pfix"}],
   ErrorListLogPlot[{{pts1X[[1]], ErrorBar[err1X[[1]]]},
     {pts1[[1]], ErrorBar[err1[[1]]]}, {pts1X[[2]], ErrorBar[err1X[[2]]]},
     {pts1[[2]], ErrorBar[err1[[2]]]}, {pts1X[[3]], ErrorBar[err1X[[3]]]},
     {pts1[[3]], ErrorBar[err1[[3]]]}},
    PlotStyle → {Orange, PointSize[0.02]}], PlotRange → All
  ]
p6X = Show[Plot[
     {PEmerB2[100, 60, R2, 0.5, 1, 20, 0.005]}, {R2, 115, 205},
     PlotRange → Automatic, PlotStyle → {Orange}, AxesLabel → {"R2", "Pfix"}],
    ErrorListPlot[{{pts1[[1]], ErrorBar[err1[[1]]]},
      {pts1[[2]], ErrorBar[err1[[2]]]}, {pts1[[3]], ErrorBar[err1[[3]]]},
      {pts1[[4]], ErrorBar[err1[[4]]]}, {pts1[[5]], ErrorBar[err1[[5]]]}},
     PlotStyle → {Orange, PointSize[0.02]}], PlotRange → All
   ];
```

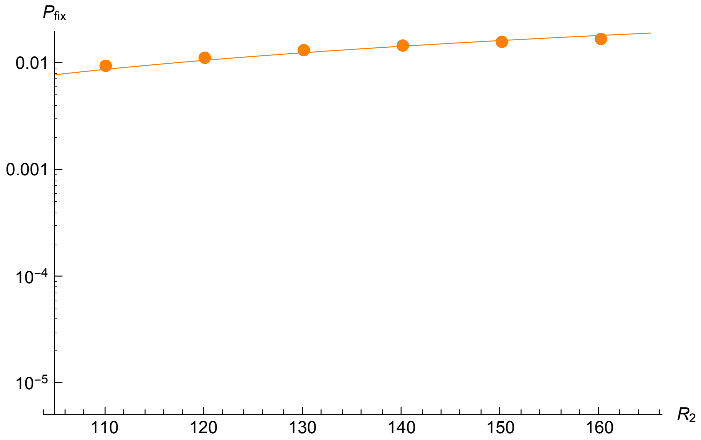

$\mu = 0.0025$

```
data1 = Transpose[Select[dataB, #[[4]] == 1.5 && #[[8]] == 0.0025 &]];
pts1  = Transpose[{data1[[3]], data1[[10]]}];
err1 = data1[[11]];
data1X = Transpose[Select[dataD, #[[4]] == 1.5 && #[[8]] == 0.0025 &]];
pts1X  = Transpose[{data1X[[3]], data1X[[10]]}];
err1X = data1X[[11]];
p7 = Show[LogPlot[
    {PEmerB2[100, 60, R2, 0.5, 1, 20, 0.0025]}, {R2, 105, 165},
    PlotRange → {Automatic, {0.000005, 0.5}},
    PlotStyle → {Brown}, AxesLabel → {"R2", "Pfix"}],
   ErrorListLogPlot[{{pts1X[[1]], ErrorBar[err1X[[1]]]},
     {pts1[[1]], ErrorBar[err1[[1]]]}, {pts1X[[2]], ErrorBar[err1X[[2]]]},
     {pts1[[2]], ErrorBar[err1[[2]]]}, {pts1X[[3]], ErrorBar[err1X[[3]]]},
     {pts1[[3]], ErrorBar[err1[[3]]]}},
    PlotStyle → {Brown, PointSize[0.02]}], PlotRange → All
  ]
p7X = Show[Plot[
     {PEmerB2[100, 60, R2, 0.5, 1, 20, 0.0025]}, {R2, 115, 205},
     PlotRange → Automatic, PlotStyle → {Brown}, AxesLabel → {"R2", "Pfix"}],
    ErrorListPlot[{{pts1[[1]], ErrorBar[err1[[1]]]},
      {pts1[[2]], ErrorBar[err1[[2]]]}, {pts1[[3]], ErrorBar[err1[[3]]]},
      {pts1[[4]], ErrorBar[err1[[4]]]}, {pts1[[5]], ErrorBar[err1[[5]]]}},
     PlotStyle → {Brown, PointSize[0.02]}], PlotRange → All
   ];
```

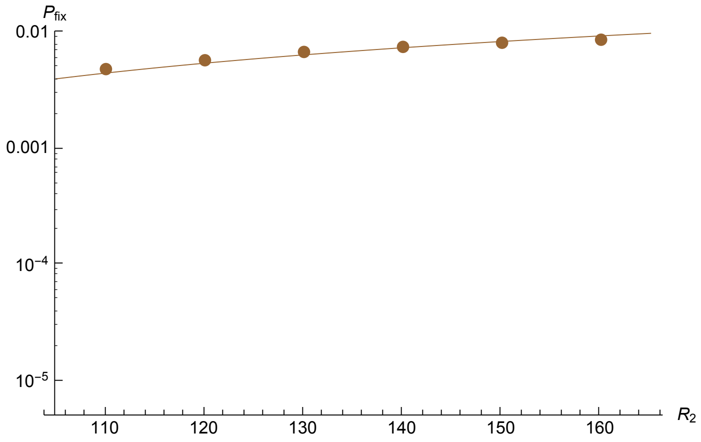

All μ together:

```
Show[p1, p2, p3, p4, p5, p6, p7]
```

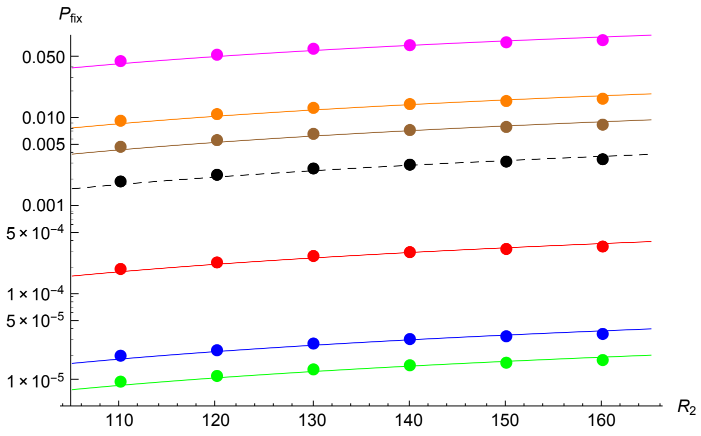

ρ = 1.0

μ = 0.001

```
data1 = Transpose[Select[dataA, #[[4]] == 2 && #[[8]] == 0.001 &]];
pts1 = Transpose[{data1[[3]], data1[[10]]}];
err1 = data1[[11]];
data1X = Transpose[Select[dataD, #[[4]] == 2 && #[[8]] == 0.001 &]];
pts1X = Transpose[{data1X[[3]], data1X[[10]]}];
err1X = data1X[[11]];
p1 = Show[LogPlot[
   {PEmerB2[100, 60, R2, 1, 1, 20, 0.001]}, {R2, 105, 165},
   PlotRange → {Automatic, {0.000005, 0.01}},
   PlotStyle → {Black, Dashed}, AxesLabel → {"R2", "Pfix"}],
  ErrorListLogPlot[{{pts1X[[1]], ErrorBar[err1X[[1]]]},
    {pts1[[1]], ErrorBar[err1[[1]]]}, {pts1X[[2]], ErrorBar[err1X[[2]]]},
    {pts1[[2]], ErrorBar[err1[[2]]]}, {pts1[[3]], ErrorBar[err1[[3]]]},
    {pts1X[[3]], ErrorBar[err1X[[3]]]}},
   PlotStyle → {Black, PointSize[0.02]}], PlotRange → All
 ]
p1X = Show[Plot[
    {PEmerB2[100, 60, R2, 1, 1, 20, 0.001]}, {R2, 105, 165},
    PlotRange → Automatic, PlotStyle → {Black, Dashed}, AxesLabel → {"R2", "Pfix"}],
   ErrorListPlot[
    {{pts1X[[1]], ErrorBar[err1X[[1]]]}, {pts1[[1]], ErrorBar[err1[[1]]]},
     {pts1X[[2]], ErrorBar[err1X[[2]]]}, {pts1[[2]], ErrorBar[err1[[2]]]},
     {pts1X[[3]], ErrorBar[err1X[[3]]]}, {pts1[[3]], ErrorBar[err1[[3]]]}},
    PlotStyle → {Black, PointSize[0.02]}], PlotRange → All
  ];
```

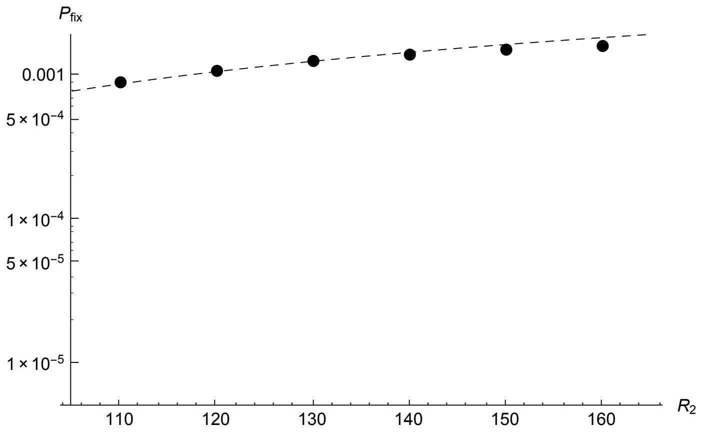

$\mu = 0.0001$

```
data1 = Transpose[Select[dataA, #[[4]] == 2 && #[[8]] == 0.0001 &]];
pts1 = Transpose[{data1[[3]], data1[[10]]}];
err1 = data1[[11]];
data1X = Transpose[Select[dataD, #[[4]] == 2 && #[[8]] == 0.0001 &]];
pts1X = Transpose[{data1X[[3]], data1X[[10]]}];
err1X = data1X[[11]];
p2 = Show[LogPlot[
    {PEmerB2[100, 60, R2, 1, 1, 20, 0.0001]}, {R2, 105, 165},
    PlotRange → {Automatic, {0.000005, 0.01}},
    PlotStyle → Red, AxesLabel → {"R2", "Pfix"}],
   ErrorListLogPlot[{{pts1X[[1]], ErrorBar[err1X[[1]]]},
     {pts1[[1]], ErrorBar[err1[[1]]]}, {pts1X[[2]], ErrorBar[err1X[[2]]]},
     {pts1[[2]], ErrorBar[err1[[2]]]}, {pts1[[3]], ErrorBar[err1[[3]]]},
     {pts1X[[3]], ErrorBar[err1X[[3]]]}},
    PlotStyle → {Red, PointSize[0.02]}], PlotRange → All
  ]
p2X = Show[Plot[
     {PEmerB2[100, 60, R2, 1, 1, 20, 0.0001]}, {R2, 105, 165},
     PlotRange → Automatic, PlotStyle → {Red}, AxesLabel → {"R2", "Pfix"}],
    ErrorListPlot[
     {{pts1X[[1]], ErrorBar[err1X[[1]]]}, {pts1[[1]], ErrorBar[err1[[1]]]},
      {pts1X[[2]], ErrorBar[err1X[[2]]]}, {pts1[[2]], ErrorBar[err1[[2]]]},
      {pts1X[[3]], ErrorBar[err1X[[3]]]}, {pts1[[3]], ErrorBar[err1[[3]]]}},
     PlotStyle → {Red, PointSize[0.02]}], PlotRange → All
   ];
```

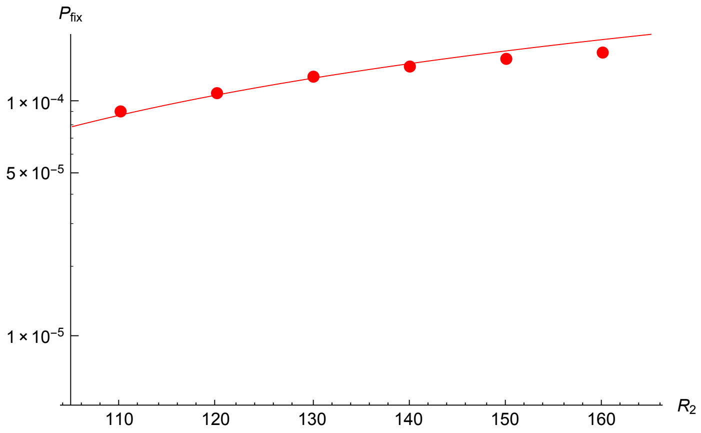

$\mu = 0.00001$

```
data1 = Transpose[Select[dataA, #[[4]] == 2 && #[[8]] == 0.00001 &]];
pts1  = Transpose[{data1[[3]], data1[[10]]}];
err1 = data1[[11]];
data1X = Transpose[Select[dataD, #[[4]] == 2 && #[[8]] == 0.00001 &]];
pts1X  = Transpose[{data1X[[3]], data1X[[10]]}];
err1X = data1X[[11]];
p3 = Show[LogPlot[
    {PEmerB2[100, 60, R2, 1, 1, 20, 0.00001]}, {R2, 105, 165},
    PlotRange → {Automatic, {0.000005, 0.01}},
    PlotStyle → Blue, AxesLabel → {"R2", "Pfix"}],
   ErrorListLogPlot[{{pts1X[[1]], ErrorBar[err1X[[1]]]},
     {pts1[[1]], ErrorBar[err1[[1]]]}, {pts1X[[2]], ErrorBar[err1X[[2]]]},
     {pts1[[2]], ErrorBar[err1[[2]]]}, {pts1[[3]], ErrorBar[err1[[3]]]},
     {pts1X[[3]], ErrorBar[err1X[[3]]]}},
    PlotStyle → {Blue, PointSize[0.02]}], PlotRange → All
  ]
p3X = Show[Plot[
     {PEmerB2[100, 60, R2, 1, 1, 20, 0.00001]}, {R2, 105, 165},
     PlotRange → Automatic, PlotStyle → {Blue}, AxesLabel → {"R2", "Pfix"}],
    ErrorListPlot[
     {{pts1X[[1]], ErrorBar[err1X[[1]]]}, {pts1[[1]], ErrorBar[err1[[1]]]},
      {pts1X[[2]], ErrorBar[err1X[[2]]]}, {pts1[[2]], ErrorBar[err1[[2]]]},
      {pts1X[[3]], ErrorBar[err1X[[3]]]}, {pts1[[3]], ErrorBar[err1[[3]]]}},
     PlotStyle → {Blue, PointSize[0.02]}], PlotRange → All
   ];
```

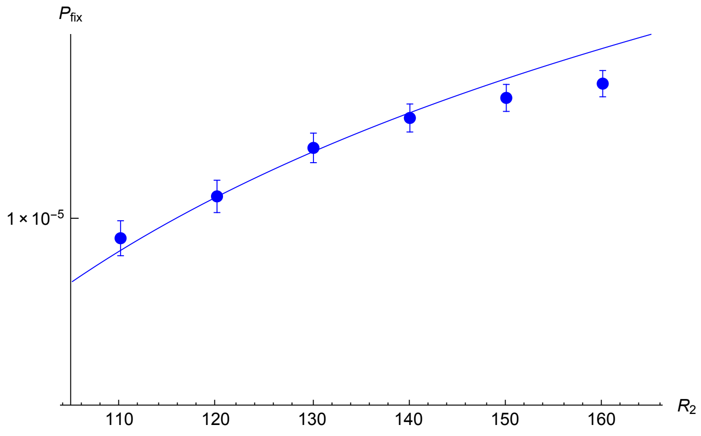

μ = 0.000005

```
data1 = Transpose[Select[dataA, #[[4]] == 2 && #[[8]] == 0.000005 &]];
pts1 = Transpose[{data1[[3]], data1[[10]]}];
err1 = data1[[11]];
data1X = Transpose[Select[dataD, #[[4]] == 2 && #[[8]] == 0.000005 &]];
pts1X = Transpose[{data1X[[3]], data1X[[10]]}];
err1X = data1X[[11]];
p4 = Show[LogPlot[
    {PEmerB2[100, 60, R2, 1, 1, 20, 0.000005]}, {R2, 105, 165},
    PlotRange → {Automatic, {0.0000025, 0.01}},
    PlotStyle → Green, AxesLabel → {"R2", "Pfix"}],
  ErrorListLogPlot[{{pts1X[[1]], ErrorBar[err1X[[1]]]},
     {pts1[[1]], ErrorBar[err1[[1]]]}, {pts1X[[2]], ErrorBar[err1X[[2]]]},
     {pts1[[2]], ErrorBar[err1[[2]]]}, {pts1[[3]], ErrorBar[err1[[3]]]},
     {pts1X[[3]], ErrorBar[err1X[[3]]]}},
    PlotStyle → {Green, PointSize[0.02]}], PlotRange → All
 ]
p4X = Show[Plot[
     {PEmerB2[100, 60, R2, 1, 1, 20, 0.000005]}, {R2, 105, 165},
     PlotRange → Automatic, PlotStyle → {Green}, AxesLabel → {"R2", "Pfix"}],
    ErrorListPlot[
     {{pts1X[[1]], ErrorBar[err1X[[1]]]}, {pts1[[1]], ErrorBar[err1[[1]]]},
      {pts1X[[2]], ErrorBar[err1X[[2]]]}, {pts1[[2]], ErrorBar[err1[[2]]]},
      {pts1X[[3]], ErrorBar[err1X[[3]]]}, {pts1[[3]], ErrorBar[err1[[3]]]}},
     PlotStyle → {Green, PointSize[0.02]}], PlotRange → All
  ];
```

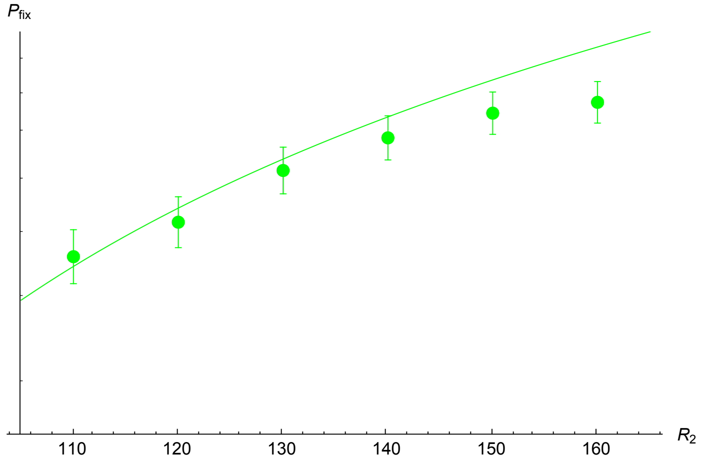

All μ together:

```
Show[p1, p2, p3, p4]
```

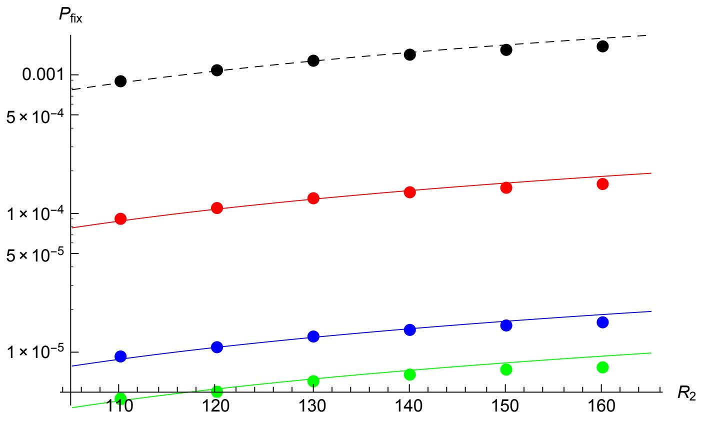


```
Show[p1X, p2X, p3X, p4X]
```

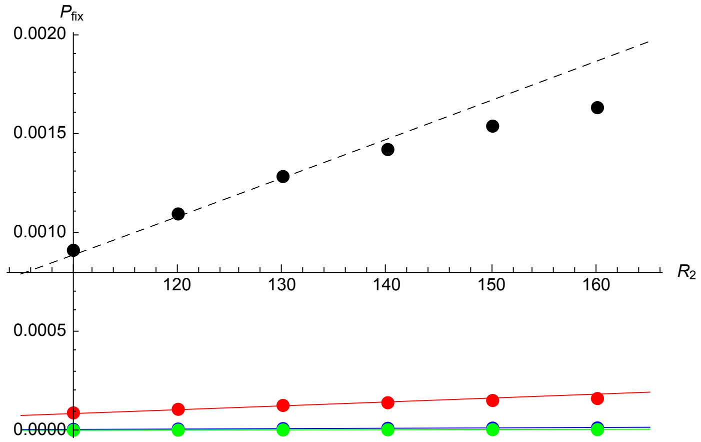

$\mu = 0.025$

```
data1 = Transpose[Select[dataB, #[[4]] == 2 && #[[8]] == 0.025 &]];
pts1  = Transpose[{data1[[3]], data1[[10]]}];
err1 = data1[[11]];
data1X = Transpose[Select[dataD, #[[4]] == 2 && #[[8]] == 0.025 &]];
pts1X  = Transpose[{data1X[[3]], data1X[[10]]}];
err1X = data1X[[11]];
p5 = Show[LogPlot[
    {PEmerB2[100, 60, R2, 1, 1, 20, 0.025]}, {R2, 105, 165},
    PlotRange → {Automatic, {0.000005, 0.1}},
    PlotStyle → Magenta, AxesLabel → {"R2", "Pfix"}],
   ErrorListLogPlot[{{pts1X[[1]], ErrorBar[err1X[[1]]]},
     {pts1[[1]], ErrorBar[err1[[1]]]}, {pts1X[[2]], ErrorBar[err1X[[2]]]},
     {pts1[[2]], ErrorBar[err1[[2]]]}, {pts1[[3]], ErrorBar[err1[[3]]]},
     {pts1X[[3]], ErrorBar[err1X[[3]]]}},
    PlotStyle → {Magenta, PointSize[0.02]}, PlotRange → All
  ]
p5X = Show[Plot[
     {PEmerB2[100, 60, R2, 1, 1, 20, 0.025]}, {R2, 115, 205},
     PlotRange → Automatic, PlotStyle → {Magenta}, AxesLabel → {"R2", "Pfix"}],
    ErrorListPlot[{{pts1[[1]], ErrorBar[err1[[1]]]},
      {pts1[[2]], ErrorBar[err1[[2]]]}, {pts1[[3]], ErrorBar[err1[[3]]]},
      {pts1[[4]], ErrorBar[err1[[4]]]}, {pts1[[5]], ErrorBar[err1[[5]]]}},
     PlotStyle → {Magenta, PointSize[0.02]}, PlotRange → All
   ];
```

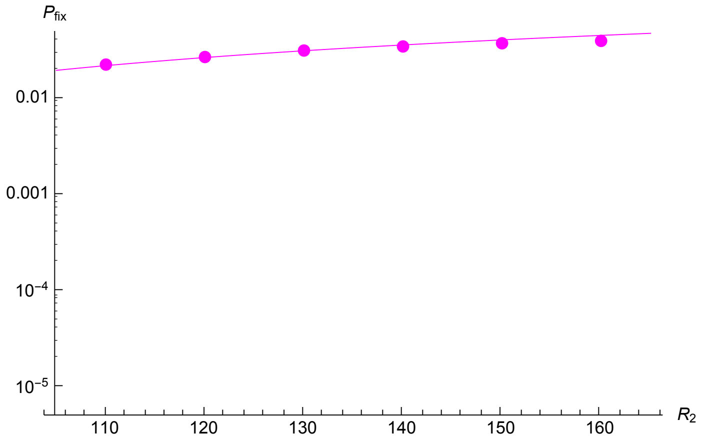

$\mu = 0.005$

```
data1 = Transpose[Select[dataB, #[[4]] == 2 && #[[8]] == 0.005 &]];
pts1  = Transpose[{data1[[3]], data1[[10]]}];
err1 = data1[[11]];
data1X = Transpose[Select[dataD, #[[4]] == 2 && #[[8]] == 0.005 &]];
pts1X  = Transpose[{data1X[[3]], data1X[[10]]}];
err1X = data1X[[11]];
p6 = Show[LogPlot[
    {PEmerB2[100, 60, R2, 1, 1, 20, 0.005]}, {R2, 105, 165},
    PlotRange → {Automatic, {0.000005, 0.1}},
    PlotStyle → Orange, AxesLabel → {"R2", "Pfix"}],
  ErrorListLogPlot[{{pts1X[[1]], ErrorBar[err1X[[1]]]},
     {pts1[[1]], ErrorBar[err1[[1]]]}, {pts1X[[2]], ErrorBar[err1X[[2]]]},
     {pts1[[2]], ErrorBar[err1[[2]]]}, {pts1[[3]], ErrorBar[err1[[3]]]},
     {pts1X[[3]], ErrorBar[err1X[[3]]]}},
    PlotStyle → {Orange, PointSize[0.02]}], PlotRange → All
 ]
p6X = Show[Plot[
     {PEmerB2[100, 60, R2, 1, 1, 20, 0.005]}, {R2, 115, 205},
     PlotRange → Automatic, PlotStyle → {Orange}, AxesLabel → {"R2", "Pfix"}],
    ErrorListPlot[{{pts1[[1]], ErrorBar[err1[[1]]]},
      {pts1[[2]], ErrorBar[err1[[2]]]}, {pts1[[3]], ErrorBar[err1[[3]]]},
      {pts1[[4]], ErrorBar[err1[[4]]]}, {pts1[[5]], ErrorBar[err1[[5]]]}},
     PlotStyle → {Orange, PointSize[0.02]}], PlotRange → All
  ];
```

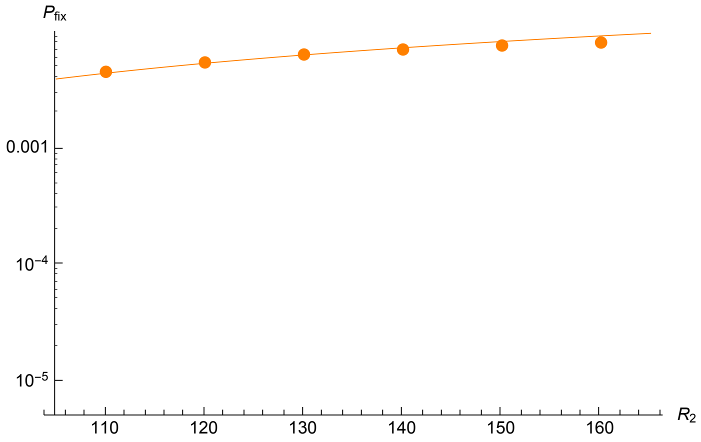

$\mu = 0.0025$

```
data1 = Transpose[Select[dataB, #[[4]] == 2 && #[[8]] == 0.0025 &]];
pts1  = Transpose[{data1[[3]], data1[[10]]}];
err1 = data1[[11]];
data1X = Transpose[Select[dataD, #[[4]] == 2 && #[[8]] == 0.0025 &]];
pts1X  = Transpose[{data1X[[3]], data1X[[10]]}];
err1X = data1X[[11]];
p7 = Show[LogPlot[
    {PEmerB2[100, 60, R2, 1, 1, 20, 0.0025]}, {R2, 105, 165},
    PlotRange → {Automatic, {0.000005, 0.1}},
    PlotStyle → Brown, AxesLabel → {"R2", "Pfix"}],
   ErrorListLogPlot[{{pts1X[[1]], ErrorBar[err1X[[1]]]},
     {pts1[[1]], ErrorBar[err1[[1]]]}, {pts1X[[2]], ErrorBar[err1X[[2]]]},
     {pts1[[2]], ErrorBar[err1[[2]]]}, {pts1[[3]], ErrorBar[err1[[3]]]},
     {pts1X[[3]], ErrorBar[err1X[[3]]]}},
    PlotStyle → {Brown, PointSize[0.02]}], PlotRange → All
  ]
p7X = Show[Plot[
     {PEmerB2[100, 60, R2, 1, 1, 20, 0.0025]}, {R2, 115, 205},
     PlotRange → Automatic, PlotStyle → {Brown}, AxesLabel → {"R2", "Pfix"}],
    ErrorListPlot[{{pts1[[1]], ErrorBar[err1[[1]]]},
      {pts1[[2]], ErrorBar[err1[[2]]]}, {pts1[[3]], ErrorBar[err1[[3]]]},
      {pts1[[4]], ErrorBar[err1[[4]]]}, {pts1[[5]], ErrorBar[err1[[5]]]}},
     PlotStyle → {Brown, PointSize[0.02]}], PlotRange → All
   ];
```

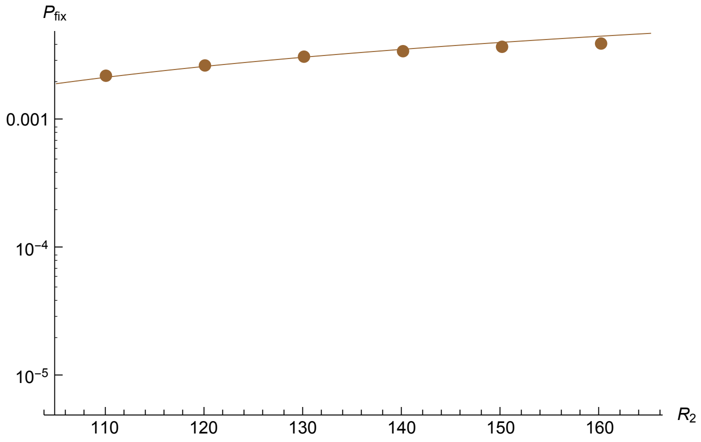

All μ together:

```
Show[p1, p2, p3, p4, p5, p6, p7]
```

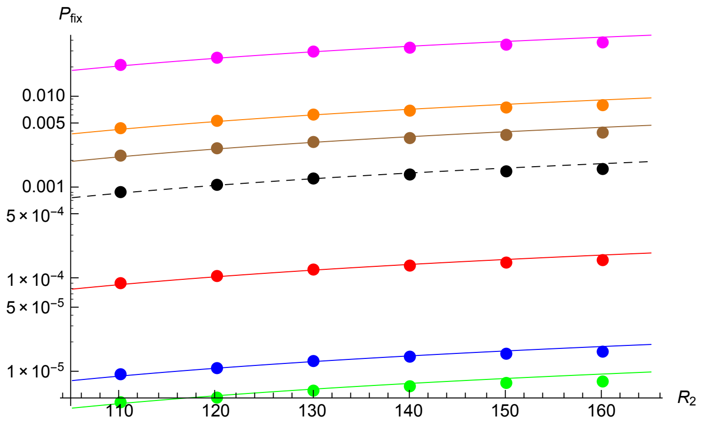

$\rho = 4$

$\mu = 0.001$

```
data1 = Transpose[Select[dataA, #[[4]] == 5 && #[[8]] == 0.001 &]];
pts1 = Transpose[{data1[[3]], data1[[10]]}];
err1 = data1[[11]];
data1X = Transpose[Select[dataD, #[[4]] == 5 && #[[8]] == 0.001 &]];
pts1X = Transpose[{data1X[[3]], data1X[[10]]}];
err1X = data1X[[11]];
p1 = Show[LogPlot[
   {PEmerB2[100, 60, R2, 4, 1, 20, 0.001]}, {R2, 105, 165},
   PlotRange → {Automatic, {0.0000005, 0.01}},
   PlotStyle → {Black, Dashed}, AxesLabel → {"R2", "Pfix"}],
  ErrorListLogPlot[{{pts1X[[1]], ErrorBar[err1X[[1]]]},
    {pts1[[1]], ErrorBar[err1[[1]]]}, {pts1X[[2]], ErrorBar[err1X[[2]]]},
    {pts1[[2]], ErrorBar[err1[[2]]]}, {pts1[[3]], ErrorBar[err1[[3]]]},
    {pts1X[[3]], ErrorBar[err1X[[3]]]}},
   PlotStyle → {Black, PointSize[0.02]}], PlotRange → All
 ]
p1X = Show[Plot[
    {PEmerB2[100, 60, R2, 4, 1, 20, 0.001]}, {R2, 105, 165},
    PlotRange → Automatic, PlotStyle → {Black, Dashed}, AxesLabel → {"R2", "Pfix"}],
   ErrorListPlot[
    {{pts1X[[1]], ErrorBar[err1X[[1]]]}, {pts1[[1]], ErrorBar[err1[[1]]]},
     {pts1X[[2]], ErrorBar[err1X[[2]]]}, {pts1[[2]], ErrorBar[err1[[2]]]},
     {pts1X[[3]], ErrorBar[err1X[[3]]]}, {pts1[[3]], ErrorBar[err1[[3]]]}},
    PlotStyle → {Black, PointSize[0.02]}], PlotRange → All
  ];
```

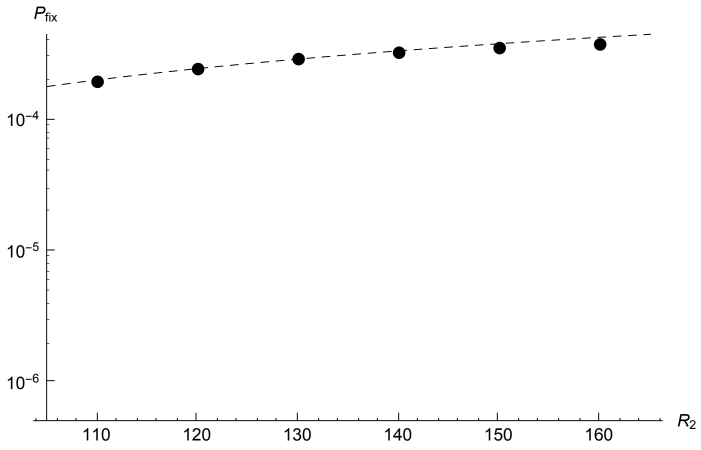

μ = 0.0001

```
data1 = Transpose[Select[dataA, #[[4]] == 5 && #[[8]] == 0.0001 &]];
pts1 = Transpose[{data1[[3]], data1[[10]]}];
err1 = data1[[11]];
data1X = Transpose[Select[dataD, #[[4]] == 5 && #[[8]] == 0.0001 &]];
pts1X = Transpose[{data1X[[3]], data1X[[10]]}];
err1X = data1X[[11]];
p2 = Show[LogPlot[
    {PEmerB2[100, 60, R2, 4, 1, 20, 0.0001]}, {R2, 105, 165},
    PlotRange → {Automatic, {0.0000005, 0.01}},
    PlotStyle → {Red}, AxesLabel → {"R2", "Pfix"}],
  ErrorListLogPlot[{{pts1X[[1]], ErrorBar[err1X[[1]]]},
     {pts1[[1]], ErrorBar[err1[[1]]]}, {pts1X[[2]], ErrorBar[err1X[[2]]]},
     {pts1[[2]], ErrorBar[err1[[2]]]}, {pts1[[3]], ErrorBar[err1[[3]]]},
     {pts1X[[3]], ErrorBar[err1X[[3]]]}},
    PlotStyle → {Red, PointSize[0.02]}], PlotRange → All
 ]
p2X = Show[Plot[
     {PEmerB2[100, 60, R2, 4, 1, 20, 0.0001]}, {R2, 105, 165},
     PlotRange → Automatic, PlotStyle → {Red}, AxesLabel → {"R2", "Pfix"}],
    ErrorListPlot[
     {{pts1X[[1]], ErrorBar[err1X[[1]]]}, {pts1[[1]], ErrorBar[err1[[1]]]},
      {pts1X[[2]], ErrorBar[err1X[[2]]]}, {pts1[[2]], ErrorBar[err1[[2]]]},
      {pts1X[[3]], ErrorBar[err1X[[3]]]}, {pts1[[3]], ErrorBar[err1[[3]]]}},
     PlotStyle → {Red, PointSize[0.02]}], PlotRange → All
  ];
```

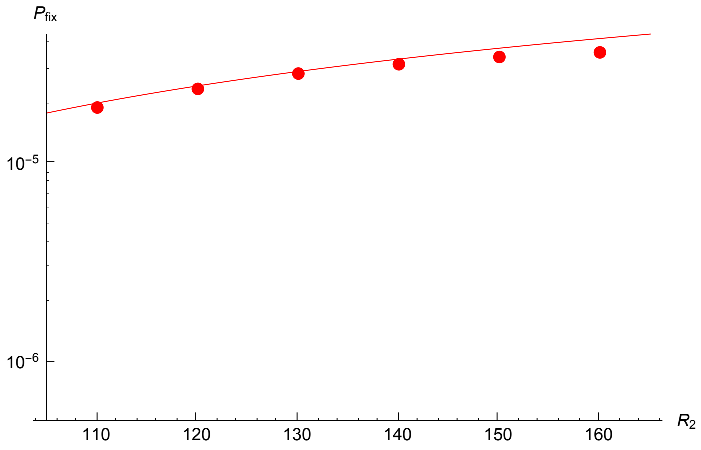

$\mu = 0.00001$

```
data1 = Transpose[Select[dataA, #[[4]] == 5 && #[[8]] == 0.00001 &]];
pts1 = Transpose[{data1[[3]], data1[[10]]}];
err1 = data1[[11]];
data1X = Transpose[Select[dataD, #[[4]] == 5 && #[[8]] == 0.00001 &]];
pts1X = Transpose[{data1X[[3]], data1X[[10]]}];
err1X = data1X[[11]];
p3 = Show[LogPlot[
    {PEmerB2[100, 60, R2, 4, 1, 20, 0.00001]}, {R2, 105, 165},
    PlotRange → {Automatic, {0.0000005, 0.01}},
    PlotStyle → {Blue}, AxesLabel → {"R₂", "Pfix"}],
   ErrorListLogPlot[{{pts1X[[1]], ErrorBar[err1X[[1]]]},
     {pts1[[1]], ErrorBar[err1[[1]]]}, {pts1X[[2]], ErrorBar[err1X[[2]]]},
     {pts1[[2]], ErrorBar[err1[[2]]]}, {pts1[[3]], ErrorBar[err1[[3]]]},
     {pts1X[[3]], ErrorBar[err1X[[3]]]}},
    PlotStyle → {Blue, PointSize[0.02]}], PlotRange → All
  ]
p3X = Show[Plot[
     {PEmerB2[100, 60, R2, 4, 1, 20, 0.00001]}, {R2, 105, 165},
     PlotRange → Automatic, PlotStyle → {Blue}, AxesLabel → {"R₂", "Pfix"}],
    ErrorListPlot[
     {{pts1X[[1]], ErrorBar[err1X[[1]]]}, {pts1[[1]], ErrorBar[err1[[1]]]},
      {pts1X[[2]], ErrorBar[err1X[[2]]]}, {pts1[[2]], ErrorBar[err1[[2]]]},
      {pts1X[[3]], ErrorBar[err1X[[3]]]}, {pts1[[3]], ErrorBar[err1[[3]]]}},
     PlotStyle → {Blue, PointSize[0.02]}], PlotRange → All
   ];
```

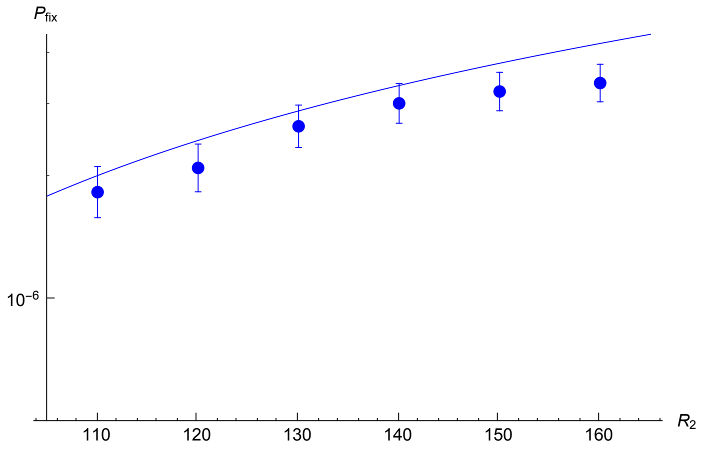

$\mu = 0.000005$

```
data1 = Transpose[Select[dataA, #[[4]] == 5 && #[[8]] == 0.000005 &]];
pts1 = Transpose[{data1[[3]], data1[[10]]}];
err1 = data1[[11]];
data1X = Transpose[Select[dataD, #[[4]] == 5 && #[[8]] == 0.000005 &]];
pts1X = Transpose[{data1X[[3]], data1X[[10]]}];
err1X = data1X[[11]];
p4 = Show[LogPlot[
   {PEmerB2[100, 60, R2, 4, 1, 20, 0.000005]}, {R2, 105, 165},
   PlotRange → {Automatic, {0.0000005, 0.01}},
   PlotStyle → {Green}, AxesLabel → {"R₂", "Pfix"}],
  ErrorListLogPlot[{{pts1X[[1]], ErrorBar[err1X[[1]]]},
    {pts1[[1]], ErrorBar[err1[[1]]]}, {pts1X[[2]], ErrorBar[err1X[[2]]]},
    {pts1[[2]], ErrorBar[err1[[2]]]}, {pts1[[3]], ErrorBar[err1[[3]]]},
    {pts1X[[3]], ErrorBar[err1X[[3]]]}},
   PlotStyle → {Green, PointSize[0.02]}], PlotRange → All
 ]
p4X = Show[Plot[
    {PEmerB2[100, 60, R2, 4, 1, 20, 0.000005]}, {R2, 105, 165},
    PlotRange → Automatic, PlotStyle → {Green}, AxesLabel → {"R₂", "Pfix"}],
   ErrorListPlot[
    {{pts1X[[1]], ErrorBar[err1X[[1]]]}, {pts1[[1]], ErrorBar[err1[[1]]]},
     {pts1X[[2]], ErrorBar[err1X[[2]]]}, {pts1[[2]], ErrorBar[err1[[2]]]},
     {pts1X[[3]], ErrorBar[err1X[[3]]]}, {pts1[[3]], ErrorBar[err1[[3]]]}},
    PlotStyle → {Green, PointSize[0.02]}], PlotRange → All
  ];
```

All μ together:

```
Show[p1, p2, p3, p4]
```

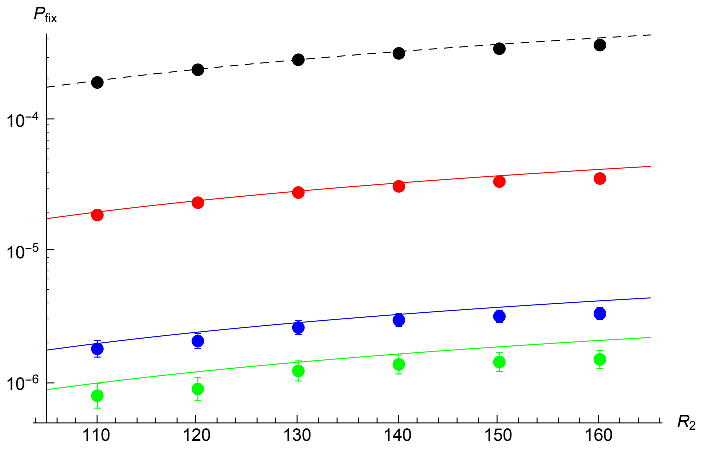

```
Show[p1X, p2X, p3X, p4X]
```

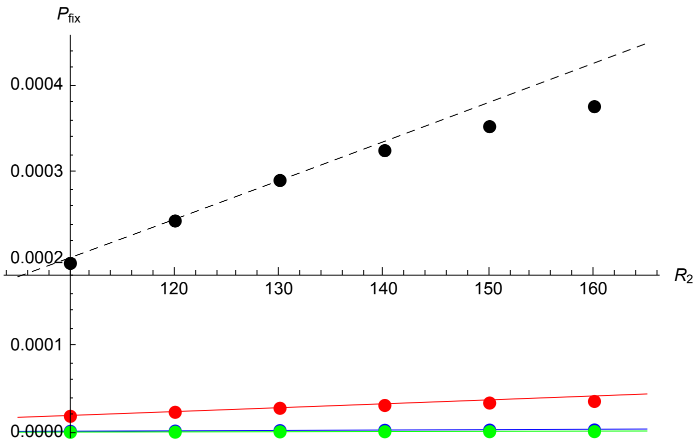

μ = 0.025

```
data1 = Transpose[Select[dataB, #[[4]] == 5 && #[[8]] == 0.025 &]];
pts1  = Transpose[{data1[[3]], data1[[10]]}];
err1 = data1[[11]];
data1X = Transpose[Select[dataD, #[[4]] == 5 && #[[8]] == 0.025 &]];
pts1X  = Transpose[{data1X[[3]], data1X[[10]]}];
err1X = data1X[[11]];
p5 = Show[LogPlot[
   {PEmerB2[100, 60, R2, 4, 1, 20, 0.025]}, {R2, 105, 165},
   PlotRange → {Automatic, {0.00001, 0.05}},
   PlotStyle → {Magenta}, AxesLabel → {"R₂", "Pfix"}],
  ErrorListLogPlot[{{pts1X[[1]], ErrorBar[err1X[[1]]]},
    {pts1[[1]], ErrorBar[err1[[1]]]}, {pts1X[[2]], ErrorBar[err1X[[2]]]},
    {pts1[[2]], ErrorBar[err1[[2]]]}, {pts1[[3]], ErrorBar[err1[[3]]]},
    {pts1X[[3]], ErrorBar[err1X[[3]]]}},
   PlotStyle → {Magenta, PointSize[0.02]}], PlotRange → All
 ]
p5X = Show[Plot[
    {PEmerB2[100, 60, R2, 4, 1, 20, 0.025]}, {R2, 115, 205},
    PlotRange → Automatic, PlotStyle → {Magenta}, AxesLabel → {"R₂", "Pfix"}],
   ErrorListPlot[{{pts1[[1]], ErrorBar[err1[[1]]]},
     {pts1[[2]], ErrorBar[err1[[2]]]}, {pts1[[3]], ErrorBar[err1[[3]]]},
     {pts1[[4]], ErrorBar[err1[[4]]]}, {pts1[[5]], ErrorBar[err1[[5]]]}},
    PlotStyle → {Magenta, PointSize[0.02]}], PlotRange → All
  ];
```

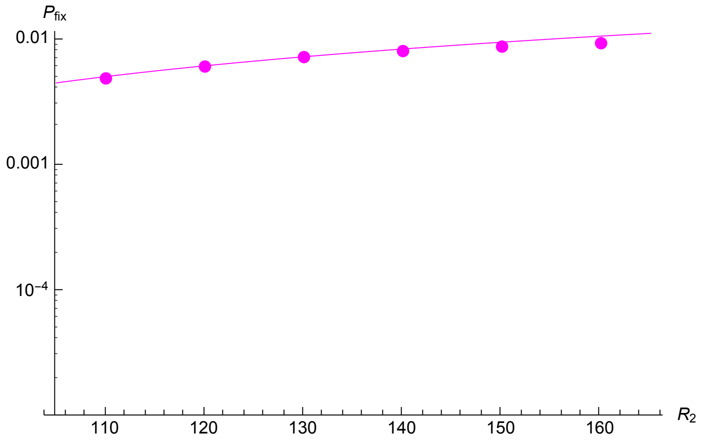

μ = 0.005

```
data1 = Transpose[Select[dataB, #[[4]] == 5 && #[[8]] == 0.005 &]];
pts1  = Transpose[{data1[[3]], data1[[10]]}];
err1 = data1[[11]];
data1X = Transpose[Select[dataD, #[[4]] == 5 && #[[8]] == 0.005 &]];
pts1X  = Transpose[{data1X[[3]], data1X[[10]]}];
err1X = data1X[[11]];
p6 = Show[LogPlot[
    {PEmerB2[100, 60, R2, 4, 1, 20, 0.005]}, {R2, 105, 165},
    PlotRange → {Automatic, {0.00001, 0.05}},
    PlotStyle → {Orange}, AxesLabel → {"R₂", "Pfix"}],
  ErrorListLogPlot[{{pts1X[[1]], ErrorBar[err1X[[1]]]},
     {pts1[[1]], ErrorBar[err1[[1]]]}, {pts1X[[2]], ErrorBar[err1X[[2]]]},
     {pts1[[2]], ErrorBar[err1[[2]]]}, {pts1[[3]], ErrorBar[err1[[3]]]},
     {pts1X[[3]], ErrorBar[err1X[[3]]]}},
    PlotStyle → {Orange, PointSize[0.02]}], PlotRange → All
 ]
p6X = Show[Plot[
     {PEmerB2[100, 60, R2, 4, 1, 20, 0.005]}, {R2, 115, 205},
     PlotRange → Automatic, PlotStyle → {Orange}, AxesLabel → {"R₂", "Pfix"}],
    ErrorListPlot[{{pts1[[1]], ErrorBar[err1[[1]]]},
      {pts1[[2]], ErrorBar[err1[[2]]]}, {pts1[[3]], ErrorBar[err1[[3]]]},
      {pts1[[4]], ErrorBar[err1[[4]]]}, {pts1[[5]], ErrorBar[err1[[5]]]}},
     PlotStyle → {Orange, PointSize[0.02]}], PlotRange → All
   ];
```

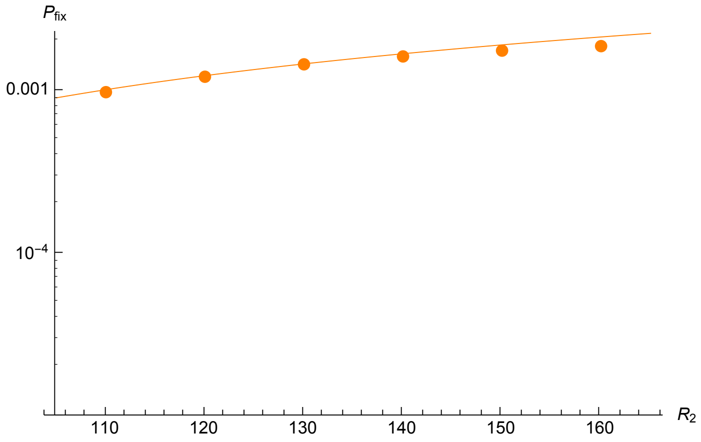

$\mu = 0.0025$

```
data1 = Transpose[Select[dataB, #[[4]] == 5 && #[[8]] == 0.0025 &]];
pts1 = Transpose[{data1[[3]], data1[[10]]}];
err1 = data1[[11]];
data1X = Transpose[Select[dataD, #[[4]] == 5 && #[[8]] == 0.0025 &]];
pts1X = Transpose[{data1X[[3]], data1X[[10]]}];
err1X = data1X[[11]];
p7 = Show[LogPlot[
    {PEmerB2[100, 60, R2, 4, 1, 20, 0.0025]}, {R2, 105, 165},
    PlotRange → {Automatic, {0.00001, 0.05}},
    PlotStyle → {Brown}, AxesLabel → {"R2", "Pfix"}],
   ErrorListLogPlot[{{pts1X[[1]], ErrorBar[err1X[[1]]]},
     {pts1[[1]], ErrorBar[err1[[1]]]}, {pts1X[[2]], ErrorBar[err1X[[2]]]},
     {pts1[[2]], ErrorBar[err1[[2]]]}, {pts1[[3]], ErrorBar[err1[[3]]]},
     {pts1X[[3]], ErrorBar[err1X[[3]]]}},
    PlotStyle → {Brown, PointSize[0.02]}], PlotRange → All
  ]
p7X = Show[Plot[
     {PEmerB2[100, 60, R2, 4, 1, 20, 0.0025]}, {R2, 115, 205},
     PlotRange → Automatic, PlotStyle → {Brown}, AxesLabel → {"R2", "Pfix"}],
    ErrorListPlot[{{pts1[[1]], ErrorBar[err1[[1]]]},
      {pts1[[2]], ErrorBar[err1[[2]]]}, {pts1[[3]], ErrorBar[err1[[3]]]},
      {pts1[[4]], ErrorBar[err1[[4]]]}, {pts1[[5]], ErrorBar[err1[[5]]]}},
     PlotStyle → {Brown, PointSize[0.02]}], PlotRange → All
   ];
```

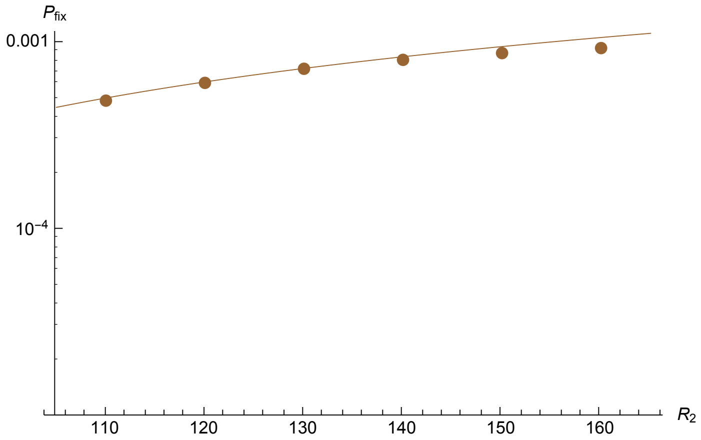

All μ together:

```
Show[p1, p2, p3, p4, p5, p6, p7]
```

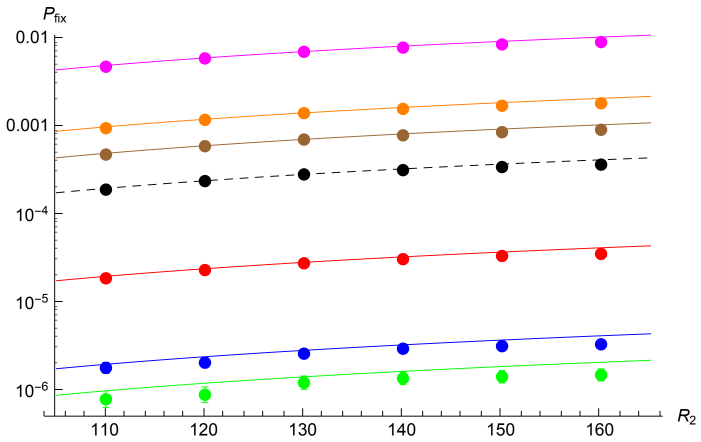

ρ = 6.5

μ = 0.001

```
data1 = Transpose[Select[dataA, #[[4]] == 7.5 && #[[8]] == 0.001 &]];
pts1 = Transpose[{data1[[3]], data1[[10]]}];
err1 = data1[[11]];
data1X = Transpose[Select[dataD, #[[4]] == 7.5 && #[[8]] == 0.001 &]];
pts1X = Transpose[{data1X[[3]], data1X[[10]]}];
err1X = data1X[[11]];
p1 = Show[LogPlot[
   {PEmerB2[100, 60, R2, 6.5, 1, 20, 0.001]}, {R2, 105, 165},
   PlotRange → {Automatic, {0.0000001, 0.01}},
   PlotStyle → {Black, Dashed}, AxesLabel → {"R2", "Pfix"}],
  ErrorListLogPlot[{{pts1X[[1]], ErrorBar[err1X[[1]]]},
    {pts1[[1]], ErrorBar[err1[[1]]]}, {pts1X[[2]], ErrorBar[err1X[[2]]]},
    {pts1[[2]], ErrorBar[err1[[2]]]}, {pts1[[3]], ErrorBar[err1[[3]]]},
    {pts1X[[3]], ErrorBar[err1X[[3]]]}},
   PlotStyle → {Black, PointSize[0.02]}], PlotRange → All
 ]
p1X = Show[Plot[
    {PEmerB2[100, 60, R2, 6.5, 1, 20, 0.001]}, {R2, 105, 165},
    PlotRange → Automatic, PlotStyle → {Black, Dashed}, AxesLabel → {"R2", "Pfix"}],
   ErrorListPlot[
    {{pts1X[[1]], ErrorBar[err1X[[1]]]}, {pts1[[1]], ErrorBar[err1[[1]]]},
     {pts1X[[2]], ErrorBar[err1X[[2]]]}, {pts1[[2]], ErrorBar[err1[[2]]]},
     {pts1X[[3]], ErrorBar[err1X[[3]]]}, {pts1[[3]], ErrorBar[err1[[3]]]}},
    PlotStyle → {Black, PointSize[0.02]}], PlotRange → All
  ];
```

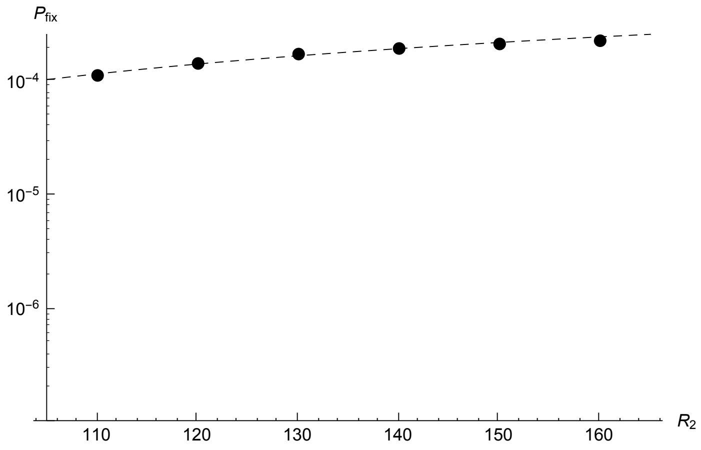

μ = 0.0001

```
data1 = Transpose[Select[dataA, #[[4]] == 7.5 && #[[8]] == 0.0001 &]];
pts1  = Transpose[{data1[[3]], data1[[10]]}];
err1 = data1[[11]];
data1X = Transpose[Select[dataD, #[[4]] == 7.5 && #[[8]] == 0.0001 &]];
pts1X  = Transpose[{data1X[[3]], data1X[[10]]}];
err1X = data1X[[11]];
p2 = Show[LogPlot[
    {PEmerB2[100, 60, R2, 6.5, 1, 20, 0.0001]}, {R2, 105, 165},
    PlotRange → {Automatic, {0.0000001, 0.01}},
    PlotStyle → {Red}, AxesLabel → {"R2", "Pfix"}],
   ErrorListLogPlot[{{pts1X[[1]], ErrorBar[err1X[[1]]]},
     {pts1[[1]], ErrorBar[err1[[1]]]}, {pts1X[[2]], ErrorBar[err1X[[2]]]},
     {pts1[[2]], ErrorBar[err1[[2]]]}, {pts1[[3]], ErrorBar[err1[[3]]]},
     {pts1X[[3]], ErrorBar[err1X[[3]]]}},
    PlotStyle → {Red, PointSize[0.02]}], PlotRange → All
  ]
p2X = Show[Plot[
     {PEmerB2[100, 60, R2, 6.5, 1, 20, 0.0001]}, {R2, 105, 165},
     PlotRange → Automatic, PlotStyle → {Red}, AxesLabel → {"R2", "Pfix"}],
    ErrorListPlot[
     {{pts1X[[1]], ErrorBar[err1X[[1]]]}, {pts1[[1]], ErrorBar[err1[[1]]]},
      {pts1X[[2]], ErrorBar[err1X[[2]]]}, {pts1[[2]], ErrorBar[err1[[2]]]},
      {pts1X[[3]], ErrorBar[err1X[[3]]]}, {pts1[[3]], ErrorBar[err1[[3]]]}},
     PlotStyle → {Red, PointSize[0.02]}], PlotRange → All
   ];
```

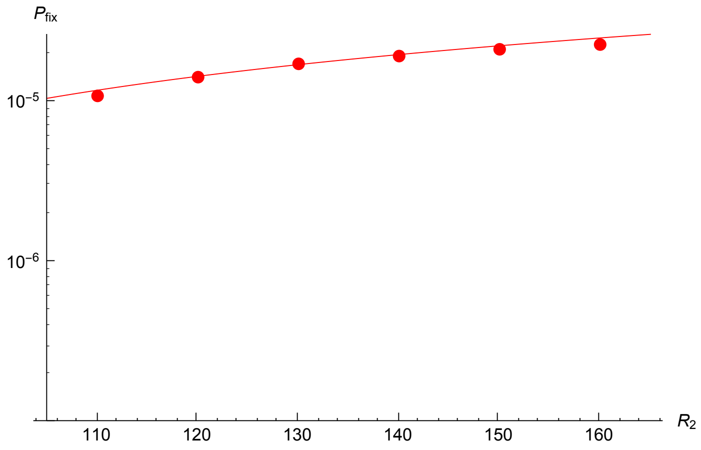

μ = 0.00001

```
data1 = Transpose[Select[dataA, #[[4]] == 7.5 && #[[8]] == 0.00001 &]];
pts1  = Transpose[{data1[[3]], data1[[10]]}];
err1 = data1[[11]];
data1X = Transpose[Select[dataD, #[[4]] == 7.5 && #[[8]] == 0.00001 &]];
pts1X  = Transpose[{data1X[[3]], data1X[[10]]}];
err1X = data1X[[11]];
p3 = Show[LogPlot[
    {PEmerB2[100, 60, R2, 6.5, 1, 20, 0.00001]}, {R2, 105, 165},
    PlotRange → {Automatic, {0.0000001, 0.01}},
    PlotStyle → {Blue}, AxesLabel → {"R₂", "Pfix"}],
   ErrorListLogPlot[{{pts1X[[1]], ErrorBar[err1X[[1]]]},
     {pts1[[1]], ErrorBar[err1[[1]]]}, {pts1X[[2]], ErrorBar[err1X[[2]]]},
     {pts1[[2]], ErrorBar[err1[[2]]]}, {pts1[[3]], ErrorBar[err1[[3]]]},
     {pts1X[[3]], ErrorBar[err1X[[3]]]}},
    PlotStyle → {Blue, PointSize[0.02]}], PlotRange → All
  ]
p3X = Show[Plot[
     {PEmerB2[100, 60, R2, 6.5, 1, 20, 0.00001]}, {R2, 105, 165},
     PlotRange → Automatic, PlotStyle → {Blue}, AxesLabel → {"R₂", "Pfix"}],
    ErrorListPlot[
     {{pts1X[[1]], ErrorBar[err1X[[1]]]}, {pts1[[1]], ErrorBar[err1[[1]]]},
      {pts1X[[2]], ErrorBar[err1X[[2]]]}, {pts1[[2]], ErrorBar[err1[[2]]]},
      {pts1X[[3]], ErrorBar[err1X[[3]]]}, {pts1[[3]], ErrorBar[err1[[3]]]}},
     PlotStyle → {Blue, PointSize[0.02]}], PlotRange → All
   ];
```

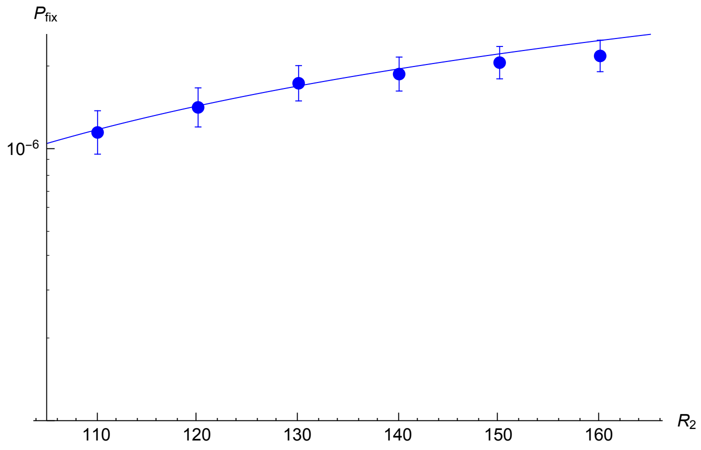

μ = 0.000005

```
data1 = Transpose[Select[dataA, #[[4]] == 7.5 && #[[8]] == 0.000005 &]];
pts1 = Transpose[{data1[[3]], data1[[10]]}];
err1 = data1[[11]];
data1X = Transpose[Select[dataD, #[[4]] == 7.5 && #[[8]] == 0.000005 &]];
pts1X = Transpose[{data1X[[3]], data1X[[10]]}];
err1X = data1X[[11]];
p4 = Show[LogPlot[
   {PEmerB2[100, 60, R2, 6.5, 1, 20, 0.000005]}, {R2, 105, 165},
   PlotRange → {Automatic, {0.0000001, 0.01}},
   PlotStyle → {Green}, AxesLabel → {"R2", "Pfix"}],
  ErrorListLogPlot[{{pts1X[[1]], ErrorBar[err1X[[1]]]},
    {pts1[[1]], ErrorBar[err1[[1]]]}, {pts1X[[2]], ErrorBar[err1X[[2]]]},
    {pts1[[2]], ErrorBar[err1[[2]]]}, {pts1[[3]], ErrorBar[err1[[3]]]},
    {pts1X[[3]], ErrorBar[err1X[[3]]]}},
   PlotStyle → {Green, PointSize[0.02]}], PlotRange → All
 ]
p4X = Show[Plot[
    {PEmerB2[100, 60, R2, 6.5, 1, 20, 0.000005]}, {R2, 105, 165},
    PlotRange → Automatic, PlotStyle → {Green}, AxesLabel → {"R2", "Pfix"}],
   ErrorListPlot[
    {{pts1X[[1]], ErrorBar[err1X[[1]]]}, {pts1[[1]], ErrorBar[err1[[1]]]},
     {pts1X[[2]], ErrorBar[err1X[[2]]]}, {pts1[[2]], ErrorBar[err1[[2]]]},
     {pts1X[[3]], ErrorBar[err1X[[3]]]}, {pts1[[3]], ErrorBar[err1[[3]]]}},
    PlotStyle → {Green, PointSize[0.02]}], PlotRange → All
  ];
```

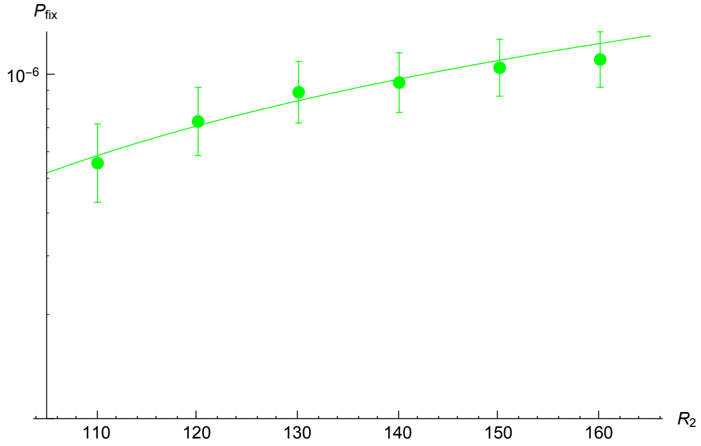

All μ together:

```
Show[p1, p2, p3, p4]
```

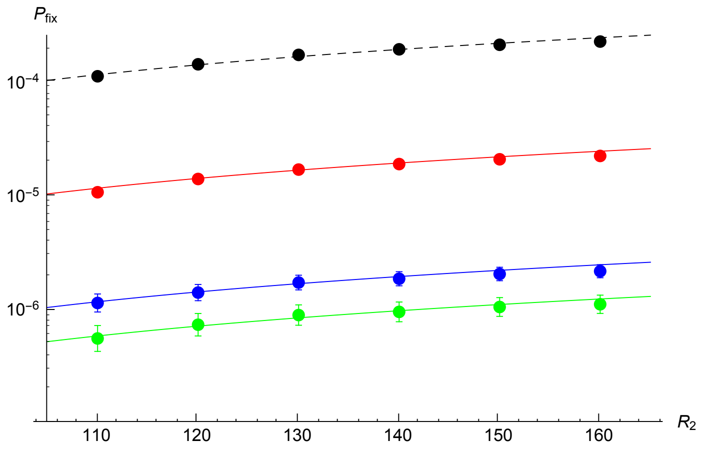


```
Show[p1X, p2X, p3X, p4X]
```

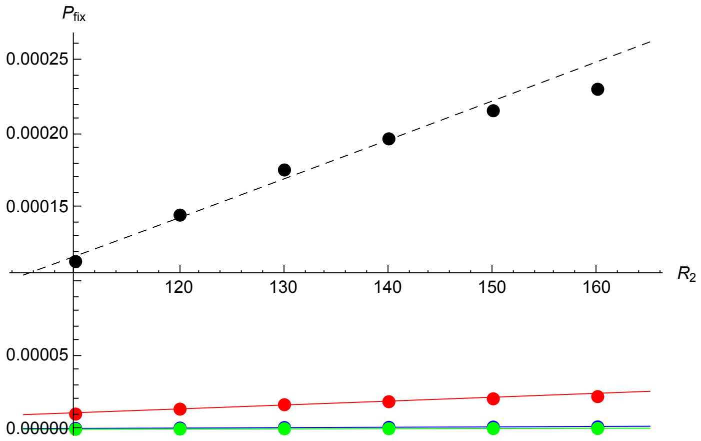

$\mu = 0.025$

```
data1 = Transpose[Select[dataB, #[[4]] == 7.5 && #[[8]] == 0.025 &]];
pts1 = Transpose[{data1[[3]], data1[[10]]}];
err1 = data1[[11]];
data1X = Transpose[Select[dataD, #[[4]] == 7.5 && #[[8]] == 0.025 &]];
pts1X = Transpose[{data1X[[3]], data1X[[10]]}];
err1X = data1X[[11]];
p5 = Show[LogPlot[
   {PEmerB2[100, 60, R2, 6.5, 1, 20, 0.025]}, {R2, 105, 165},
   PlotRange → {Automatic, {0.0000001, 0.01}},
   PlotStyle → {Magenta}, AxesLabel → {"R₂", "Pfix"}],
  ErrorListLogPlot[{{pts1X[[1]], ErrorBar[err1X[[1]]]},
    {pts1[[1]], ErrorBar[err1[[1]]]}, {pts1X[[2]], ErrorBar[err1X[[2]]]},
    {pts1[[2]], ErrorBar[err1[[2]]]}, {pts1[[3]], ErrorBar[err1[[3]]]},
    {pts1X[[3]], ErrorBar[err1X[[3]]]}},
   PlotStyle → {Magenta, PointSize[0.02]}], PlotRange → All
 ]
p5X = Show[Plot[
    {PEmerB2[100, 60, R2, 6.5, 1, 20, 0.025]}, {R2, 115, 205},
    PlotRange → Automatic, PlotStyle → {Magenta}, AxesLabel → {"R₂", "Pfix"}],
   ErrorListPlot[{{pts1[[1]], ErrorBar[err1[[1]]]},
     {pts1[[2]], ErrorBar[err1[[2]]]}, {pts1[[3]], ErrorBar[err1[[3]]]},
     {pts1[[4]], ErrorBar[err1[[4]]]}, {pts1[[5]], ErrorBar[err1[[5]]]}},
    PlotStyle → {Magenta, PointSize[0.02]}], PlotRange → All
  ];
```

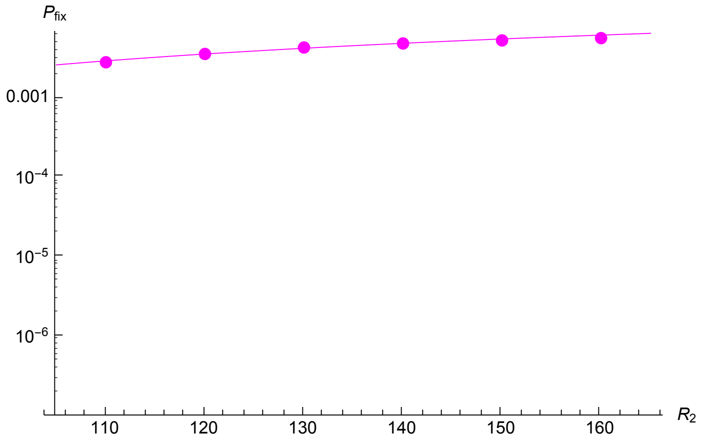

$\mu = 0.005$

```
data1 = Transpose[Select[dataB, #[[4]] == 7.5 && #[[8]] == 0.005 &]];
pts1 = Transpose[{data1[[3]], data1[[10]]}];
err1 = data1[[11]];
data1X = Transpose[Select[dataD, #[[4]] == 7.5 && #[[8]] == 0.005 &]];
pts1X = Transpose[{data1X[[3]], data1X[[10]]}];
err1X = data1X[[11]];
p6 = Show[LogPlot[
    {PEmerB2[100, 60, R2, 6.5, 1, 20, 0.005]}, {R2, 105, 165},
    PlotRange → {Automatic, {0.0000001, 0.01}},
    PlotStyle → {Orange}, AxesLabel → {"R2", "Pfix"}],
  ErrorListLogPlot[{{pts1X[[1]], ErrorBar[err1X[[1]]]},
     {pts1[[1]], ErrorBar[err1[[1]]]}, {pts1X[[2]], ErrorBar[err1X[[2]]]},
     {pts1[[2]], ErrorBar[err1[[2]]]}, {pts1[[3]], ErrorBar[err1[[3]]]},
     {pts1X[[3]], ErrorBar[err1X[[3]]]}},
    PlotStyle → {Orange, PointSize[0.02]}], PlotRange → All
 ]
p6X = Show[Plot[
     {PEmerB2[100, 60, R2, 6.5, 1, 20, 0.005]}, {R2, 115, 205},
     PlotRange → Automatic, PlotStyle → {Orange}, AxesLabel → {"R2", "Pfix"}],
    ErrorListPlot[{{pts1[[1]], ErrorBar[err1[[1]]]},
      {pts1[[2]], ErrorBar[err1[[2]]]}, {pts1[[3]], ErrorBar[err1[[3]]]},
      {pts1[[4]], ErrorBar[err1[[4]]]}, {pts1[[5]], ErrorBar[err1[[5]]]}},
     PlotStyle → {Orange, PointSize[0.02]}], PlotRange → All
  ];
```

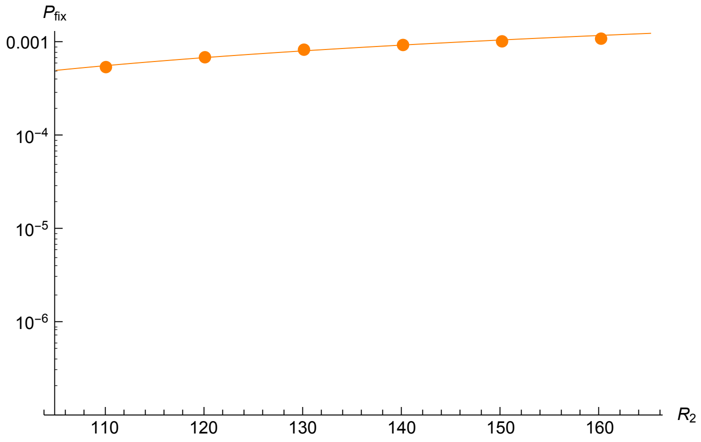

$\mu = 0.0025$

```
data1 = Transpose[Select[dataB, #[[4]] == 7.5 && #[[8]] == 0.0025 &]];
pts1  = Transpose[{data1[[3]], data1[[10]]}];
err1 = data1[[11]];
data1X = Transpose[Select[dataD, #[[4]] == 7.5 && #[[8]] == 0.0025 &]];
pts1X  = Transpose[{data1X[[3]], data1X[[10]]}];
err1X = data1X[[11]];
p7 = Show[LogPlot[
   {PEmerB2[100, 60, R2, 6.5, 1, 20, 0.0025]}, {R2, 105, 165},
   PlotRange → {Automatic, {0.0000001, 0.01}},
   PlotStyle → {Brown}, AxesLabel → {"R2", "Pfix"}],
  ErrorListLogPlot[{{pts1X[[1]], ErrorBar[err1X[[1]]]},
    {pts1[[1]], ErrorBar[err1[[1]]]}, {pts1X[[2]], ErrorBar[err1X[[2]]]},
    {pts1[[2]], ErrorBar[err1[[2]]]}, {pts1[[3]], ErrorBar[err1[[3]]]},
    {pts1X[[3]], ErrorBar[err1X[[3]]]}},
   PlotStyle → {Brown, PointSize[0.02]}], PlotRange → All
 ]
p7X = Show[Plot[
    {PEmerB2[100, 60, R2, 6.5, 1, 20, 0.0025]}, {R2, 115, 205},
    PlotRange → Automatic, PlotStyle → {Brown}, AxesLabel → {"R2", "Pfix"}],
   ErrorListPlot[{{pts1[[1]], ErrorBar[err1[[1]]]},
     {pts1[[2]], ErrorBar[err1[[2]]]}, {pts1[[3]], ErrorBar[err1[[3]]]},
     {pts1[[4]], ErrorBar[err1[[4]]]}, {pts1[[5]], ErrorBar[err1[[5]]]}},
    PlotStyle → {Brown, PointSize[0.02]}], PlotRange → All
  ];
```

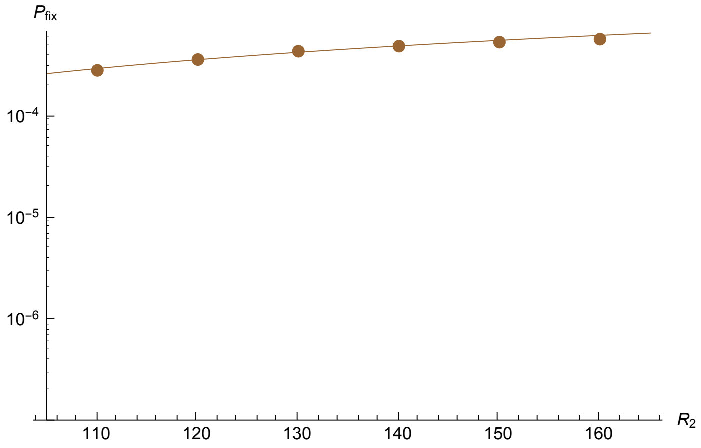

All μ together:

```
Show[p1, p2, p3, p4, p5, p6, p7]
```

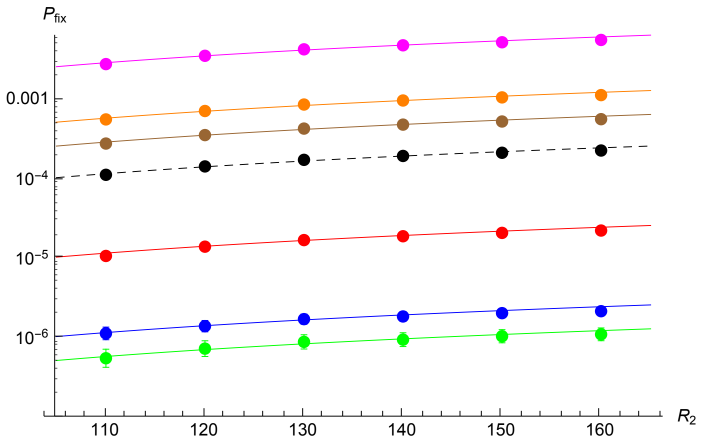

$\rho = 9$

$\mu = 0.001$

```
data1 = Transpose[Select[dataA, #[[4]] == 10 && #[[8]] == 0.001 &]];
pts1 = Transpose[{data1[[3]], data1[[10]]}];
err1 = data1[[11]];
data1X = Transpose[Select[dataD, #[[4]] == 10 && #[[8]] == 0.001 &]];
pts1X = Transpose[{data1X[[3]], data1X[[10]]}];
err1X = data1X[[11]];
p1 = Show[LogPlot[
   {PEmerB2[100, 60, R2, 9, 1, 20, 0.001]}, {R2, 105, 165},
   PlotRange → {Automatic, {0.0000001, 0.01}},
   PlotStyle → {Black, Dashed}, AxesLabel → {"R2", "Pfix"}],
  ErrorListLogPlot[{{pts1X[[1]], ErrorBar[err1X[[1]]]},
    {pts1[[1]], ErrorBar[err1[[1]]]}, {pts1X[[2]], ErrorBar[err1X[[2]]]},
    {pts1[[2]], ErrorBar[err1[[2]]]}, {pts1[[3]], ErrorBar[err1[[3]]]},
    {pts1X[[3]], ErrorBar[err1X[[3]]]}},
   PlotStyle → {Black, PointSize[0.02]}], PlotRange → All
 ]
p1X = Show[Plot[
    {PEmerB2[100, 60, R2, 9, 1, 20, 0.001]}, {R2, 105, 165},
    PlotRange → Automatic, PlotStyle → {Black, Dashed}, AxesLabel → {"R2", "Pfix"}],
   ErrorListPlot[
    {{pts1X[[1]], ErrorBar[err1X[[1]]]}, {pts1[[1]], ErrorBar[err1[[1]]]},
     {pts1X[[2]], ErrorBar[err1X[[2]]]}, {pts1[[2]], ErrorBar[err1[[2]]]},
     {pts1X[[3]], ErrorBar[err1X[[3]]]}, {pts1[[3]], ErrorBar[err1[[3]]]}},
    PlotStyle → {Black, PointSize[0.02]}], PlotRange → All
  ];
```

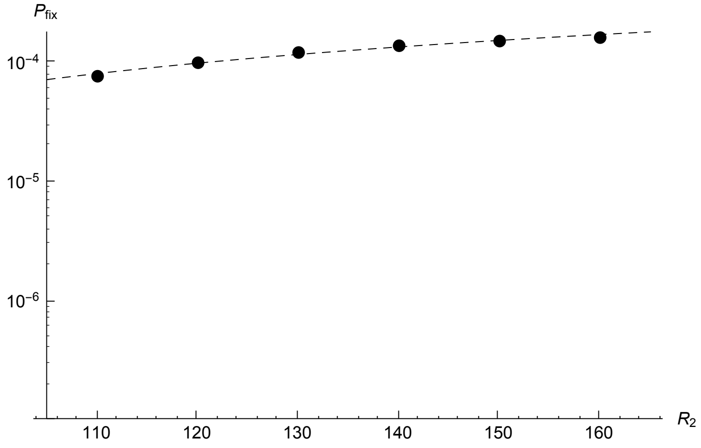

$\mu = 0.0001$

```
data1 = Transpose[Select[dataA, #[[4]] == 10 && #[[8]] == 0.0001 &]];
pts1 = Transpose[{data1[[3]], data1[[10]]}];
err1 = data1[[11]];
data1X = Transpose[Select[dataD, #[[4]] == 10 && #[[8]] == 0.0001 &]];
pts1X = Transpose[{data1X[[3]], data1X[[10]]}];
err1X = data1X[[11]];
p2 = Show[LogPlot[
    {PEmerB2[100, 60, R2, 9, 1, 20, 0.0001]}, {R2, 105, 165},
    PlotRange → {Automatic, {0.0000001, 0.01}},
    PlotStyle → {Red}, AxesLabel → {"R2", "Pfix"}],
   ErrorListLogPlot[{{pts1X[[1]], ErrorBar[err1X[[1]]]},
     {pts1[[1]], ErrorBar[err1[[1]]]}, {pts1X[[2]], ErrorBar[err1X[[2]]]},
     {pts1[[2]], ErrorBar[err1[[2]]]}, {pts1[[3]], ErrorBar[err1[[3]]]},
     {pts1X[[3]], ErrorBar[err1X[[3]]]}},
    PlotStyle → {Red, PointSize[0.02]}], PlotRange → All
  ]
p2X = Show[Plot[
     {PEmerB2[100, 60, R2, 9, 1, 20, 0.0001]}, {R2, 105, 165},
     PlotRange → Automatic, PlotStyle → {Red}, AxesLabel → {"R2", "Pfix"}],
    ErrorListPlot[
     {{pts1X[[1]], ErrorBar[err1X[[1]]]}, {pts1[[1]], ErrorBar[err1[[1]]]},
      {pts1X[[2]], ErrorBar[err1X[[2]]]}, {pts1[[2]], ErrorBar[err1[[2]]]},
      {pts1X[[3]], ErrorBar[err1X[[3]]]}, {pts1[[3]], ErrorBar[err1[[3]]]}},
     PlotStyle → {Red, PointSize[0.02]}], PlotRange → All
   ];
```

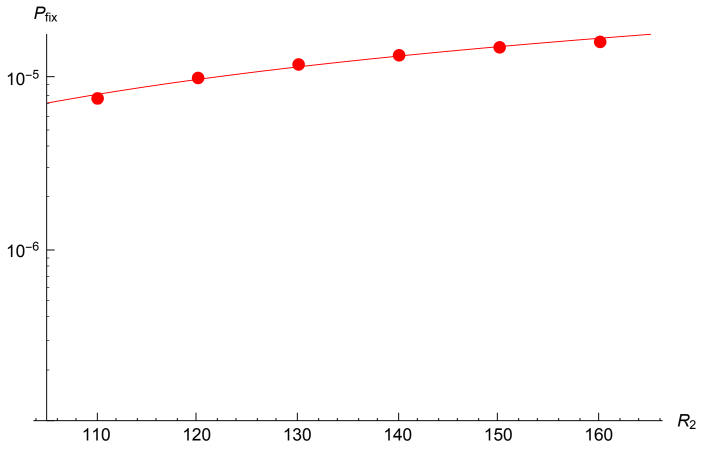

$\mu = 0.00001$

```
data1 = Transpose[Select[dataA, #[[4]] == 10 && #[[8]] == 0.00001 &]];
pts1 = Transpose[{data1[[3]], data1[[10]]}];
err1 = data1[[11]];
data1X = Transpose[Select[dataD, #[[4]] == 10 && #[[8]] == 0.00001 &]];
pts1X = Transpose[{data1X[[3]], data1X[[10]]}];
err1X = data1X[[11]];
p3 = Show[LogPlot[
   {PEmerB2[100, 60, R2, 9, 1, 20, 0.00001]}, {R2, 105, 165},
   PlotRange → {Automatic, {0.0000001, 0.01}},
   PlotStyle → {Blue}, AxesLabel → {"R2", "Pfix"}],
  ErrorListLogPlot[{{pts1X[[1]], ErrorBar[err1X[[1]]]},
    {pts1[[1]], ErrorBar[err1[[1]]]}, {pts1X[[2]], ErrorBar[err1X[[2]]]},
    {pts1[[2]], ErrorBar[err1[[2]]]}, {pts1[[3]], ErrorBar[err1[[3]]]},
    {pts1X[[3]], ErrorBar[err1X[[3]]]}},
   PlotStyle → {Blue, PointSize[0.02]}], PlotRange → All
 ]
p3X = Show[Plot[
    {PEmerB2[100, 60, R2, 9, 1, 20, 0.00001]}, {R2, 105, 165},
    PlotRange → Automatic, PlotStyle → {Blue}, AxesLabel → {"R2", "Pfix"}],
   ErrorListPlot[
    {{pts1X[[1]], ErrorBar[err1X[[1]]]}, {pts1[[1]], ErrorBar[err1[[1]]]},
     {pts1X[[2]], ErrorBar[err1X[[2]]]}, {pts1[[2]], ErrorBar[err1[[2]]]},
     {pts1X[[3]], ErrorBar[err1X[[3]]]}, {pts1[[3]], ErrorBar[err1[[3]]]}},
    PlotStyle → {Blue, PointSize[0.02]}], PlotRange → All
  ];
```

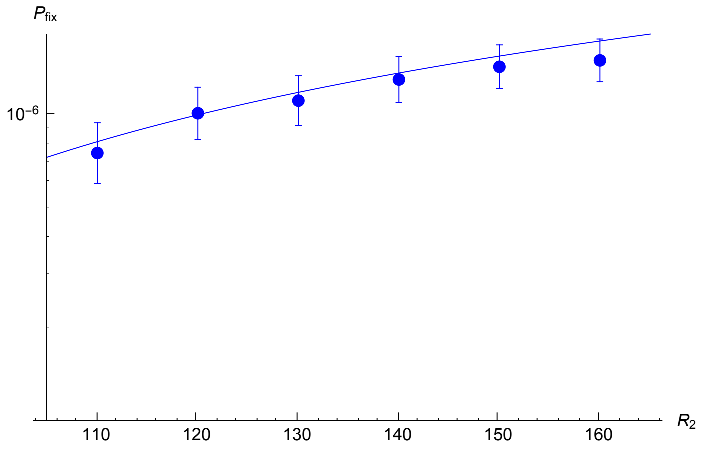

$\mu = 0.000005$

```
data1 = Transpose[Select[dataA, #[[4]] == 10 && #[[8]] == 0.000005 &]];
pts1  = Transpose[{data1[[3]], data1[[10]]}];
err1 = data1[[11]];
data1X = Transpose[Select[dataD, #[[4]] == 10 && #[[8]] == 0.000005 &]];
pts1X  = Transpose[{data1X[[3]], data1X[[10]]}];
err1X = data1X[[11]];
p4 = Show[LogPlot[
    {PEmerB2[100, 60, R2, 9, 1, 20, 0.000005]}, {R2, 105, 165},
    PlotRange → {Automatic, {0.0000001, 0.01}},
    PlotStyle → {Green}, AxesLabel → {"R2", "Pfix"}],
   ErrorListLogPlot[{{pts1X[[1]], ErrorBar[err1X[[1]]]},
     {pts1[[1]], ErrorBar[err1[[1]]]}, {pts1X[[2]], ErrorBar[err1X[[2]]]},
     {pts1[[2]], ErrorBar[err1[[2]]]}, {pts1[[3]], ErrorBar[err1[[3]]]},
     {pts1X[[3]], ErrorBar[err1X[[3]]]}},
    PlotStyle → {Green, PointSize[0.02]}], PlotRange → All
  ]
p4X = Show[Plot[
     {PEmerB2[100, 60, R2, 9, 1, 20, 0.000005]}, {R2, 105, 165},
     PlotRange → Automatic, PlotStyle → {Green}, AxesLabel → {"R2", "Pfix"}],
    ErrorListPlot[
     {{pts1X[[1]], ErrorBar[err1X[[1]]]}, {pts1[[1]], ErrorBar[err1[[1]]]},
      {pts1X[[2]], ErrorBar[err1X[[2]]]}, {pts1[[2]], ErrorBar[err1[[2]]]},
      {pts1X[[3]], ErrorBar[err1X[[3]]]}, {pts1[[3]], ErrorBar[err1[[3]]]}},
     PlotStyle → {Green, PointSize[0.02]}], PlotRange → All
   ];
```

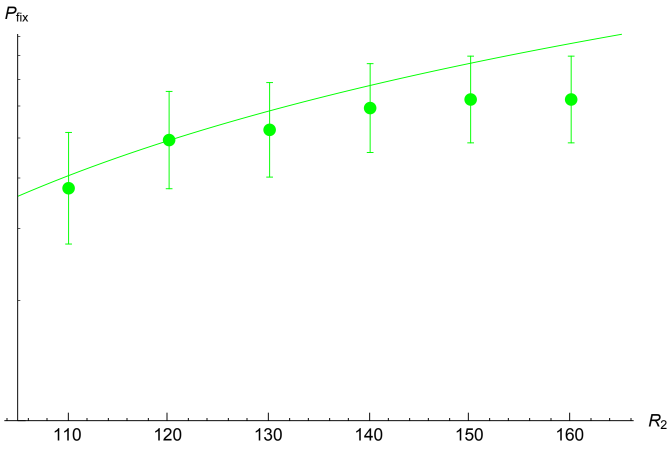

All μ together:

```
Show[p1, p2, p3, p4]
```

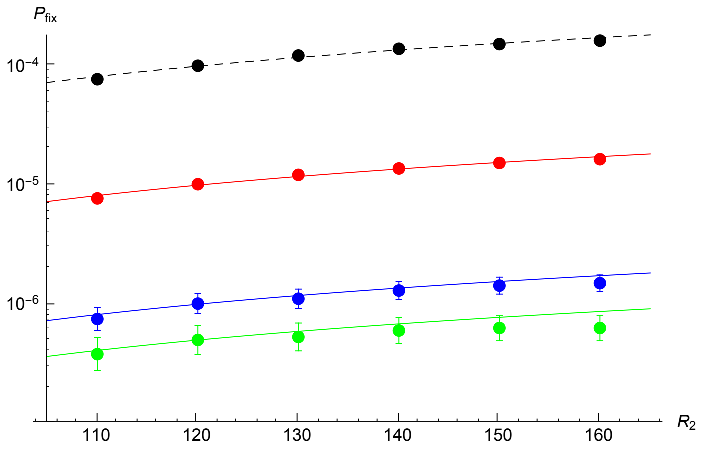


```
Show[p1X, p2X, p3X, p4X]
```

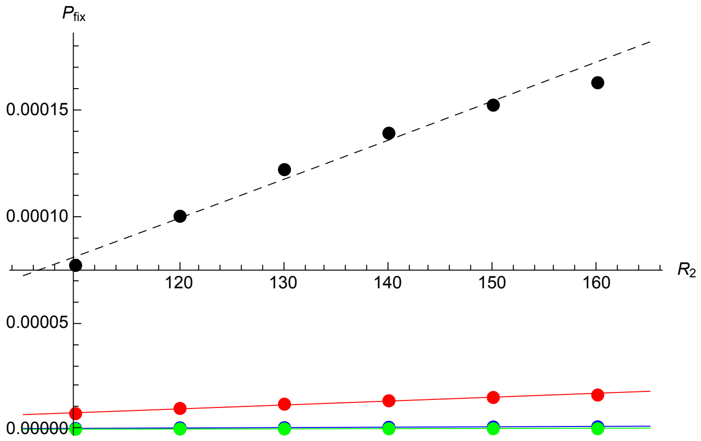

$\mu = 0.025$

```
data1 = Transpose[Select[dataB, #[[4]] == 10 && #[[8]] == 0.025 &]];
pts1  = Transpose[{data1[[3]], data1[[10]]}];
err1 = data1[[11]];
data1X = Transpose[Select[dataD, #[[4]] == 10 && #[[8]] == 0.025 &]];
pts1X  = Transpose[{data1X[[3]], data1X[[10]]}];
err1X = data1X[[11]];
p5 = Show[LogPlot[
    {PEmerB2[100, 60, R2, 9, 1, 20, 0.025]}, {R2, 105, 165},
    PlotRange → {Automatic, {0.000005, 0.01}},
    PlotStyle → {Magenta}, AxesLabel → {"R2", "Pfix"}],
   ErrorListLogPlot[{{pts1X[[1]], ErrorBar[err1X[[1]]]},
     {pts1[[1]], ErrorBar[err1[[1]]]}, {pts1X[[2]], ErrorBar[err1X[[2]]]},
     {pts1[[2]], ErrorBar[err1[[2]]]}, {pts1[[3]], ErrorBar[err1[[3]]]},
     {pts1X[[3]], ErrorBar[err1X[[3]]]}},
    PlotStyle → {Magenta, PointSize[0.02]}], PlotRange → All
  ]
p5X = Show[Plot[
     {PEmerB2[100, 60, R2, 9, 1, 20, 0.025]}, {R2, 115, 205},
     PlotRange → Automatic, PlotStyle → {Magenta}, AxesLabel → {"R2", "Pfix"}],
    ErrorListPlot[{{pts1[[1]], ErrorBar[err1[[1]]]},
      {pts1[[2]], ErrorBar[err1[[2]]]}, {pts1[[3]], ErrorBar[err1[[3]]]},
      {pts1[[4]], ErrorBar[err1[[4]]]}, {pts1[[5]], ErrorBar[err1[[5]]]}},
     PlotStyle → {Magenta, PointSize[0.02]}], PlotRange → All
   ];
```

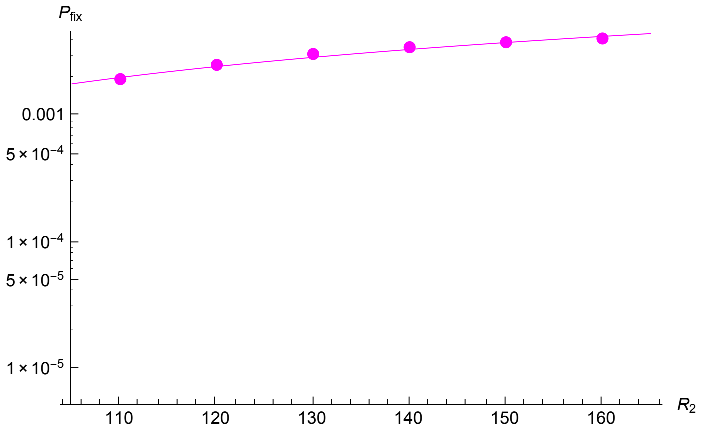

$\mu = 0.005$

```
data1 = Transpose[Select[dataB, #[[4]] == 10 && #[[8]] == 0.005 &]];
pts1  = Transpose[{data1[[3]], data1[[10]]}];
err1 = data1[[11]];
data1X = Transpose[Select[dataD, #[[4]] == 10 && #[[8]] == 0.005 &]];
pts1X  = Transpose[{data1X[[3]], data1X[[10]]}];
err1X = data1X[[11]];
p6 = Show[LogPlot[
    {PEmerB2[100, 60, R2, 9, 1, 20, 0.005]}, {R2, 105, 165},
    PlotRange → {Automatic, {0.000005, 0.01}},
    PlotStyle → {Orange}, AxesLabel → {"R2", "Pfix"}],
  ErrorListLogPlot[{{pts1X[[1]], ErrorBar[err1X[[1]]]},
     {pts1[[1]], ErrorBar[err1[[1]]]}, {pts1X[[2]], ErrorBar[err1X[[2]]]},
     {pts1[[2]], ErrorBar[err1[[2]]]}, {pts1[[3]], ErrorBar[err1[[3]]]},
     {pts1X[[3]], ErrorBar[err1X[[3]]]}},
    PlotStyle → {Orange, PointSize[0.02]}], PlotRange → All
 ]
p6X = Show[Plot[
     {PEmerB2[100, 60, R2, 9, 1, 20, 0.005]}, {R2, 115, 205},
     PlotRange → Automatic, PlotStyle → {Orange}, AxesLabel → {"R2", "Pfix"}],
    ErrorListPlot[{{pts1[[1]], ErrorBar[err1[[1]]]},
      {pts1[[2]], ErrorBar[err1[[2]]]}, {pts1[[3]], ErrorBar[err1[[3]]]},
      {pts1[[4]], ErrorBar[err1[[4]]]}, {pts1[[5]], ErrorBar[err1[[5]]]}},
     PlotStyle → {Orange, PointSize[0.02]}], PlotRange → All
   ];
```

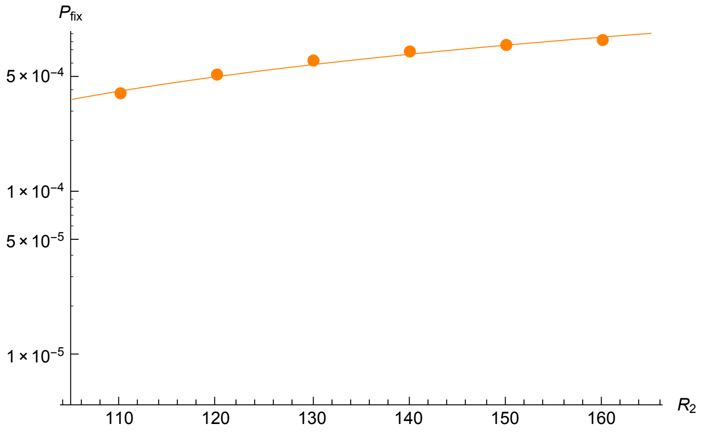

$\mu = 0.0025$

```
data1 = Transpose[Select[dataB, #[[4]] == 10 && #[[8]] == 0.0025 &]];
pts1 = Transpose[{data1[[3]], data1[[10]]}];
err1 = data1[[11]];
data1X = Transpose[Select[dataD, #[[4]] == 10 && #[[8]] == 0.0025 &]];
pts1X = Transpose[{data1X[[3]], data1X[[10]]}];
err1X = data1X[[11]];
p7 = Show[LogPlot[
   {PEmerB2[100, 60, R2, 9, 1, 20, 0.0025]}, {R2, 105, 165},
   PlotRange → {Automatic, {0.000005, 0.01}},
   PlotStyle → {Brown}, AxesLabel → {"R2", "Pfix"}],
  ErrorListLogPlot[{{pts1X[[1]], ErrorBar[err1X[[1]]]},
    {pts1[[1]], ErrorBar[err1[[1]]]}, {pts1X[[2]], ErrorBar[err1X[[2]]]},
    {pts1[[2]], ErrorBar[err1[[2]]]}, {pts1[[3]], ErrorBar[err1[[3]]]},
    {pts1X[[3]], ErrorBar[err1X[[3]]]}},
   PlotStyle → {Brown, PointSize[0.02]}], PlotRange → All
 ]
p7X = Show[Plot[
    {PEmerB2[100, 60, R2, 9, 1, 20, 0.0025]}, {R2, 115, 205},
    PlotRange → Automatic, PlotStyle → {Brown}, AxesLabel → {"R2", "Pfix"}],
   ErrorListPlot[{{pts1[[1]], ErrorBar[err1[[1]]]},
     {pts1[[2]], ErrorBar[err1[[2]]]}, {pts1[[3]], ErrorBar[err1[[3]]]},
     {pts1[[4]], ErrorBar[err1[[4]]]}, {pts1[[5]], ErrorBar[err1[[5]]]}},
    PlotStyle → {Brown, PointSize[0.02]}], PlotRange → All
  ];
```

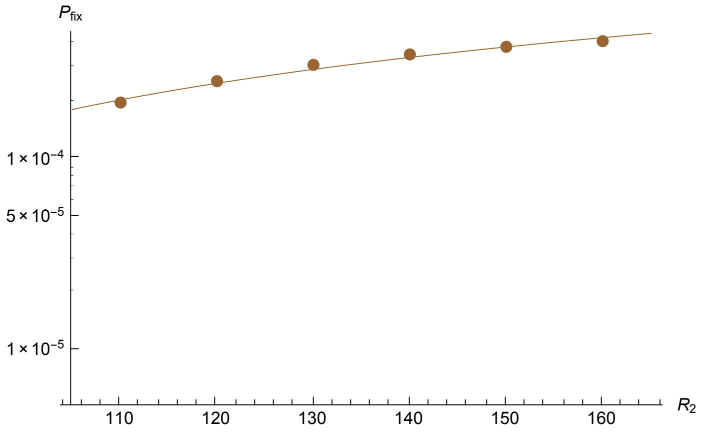

All μ together:

```
Show[p1, p2, p3, p4, p5, p6, p7]
```

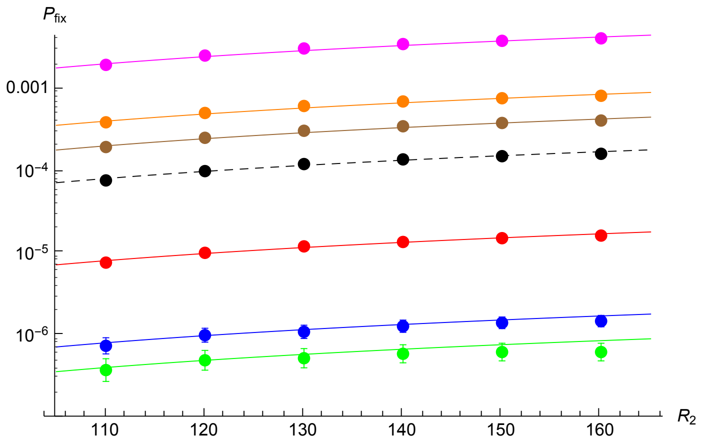

ρ = I I

μ = 0.001

```
data1 = Transpose[Select[dataA, #[[4]] == 12 && #[[8]] == 0.001 &]];
pts1 = Transpose[{data1[[3]], data1[[10]]}];
err1 = data1[[11]];
data1X = Transpose[Select[dataD, #[[4]] == 12 && #[[8]] == 0.001 &]];
pts1X = Transpose[{data1X[[3]], data1X[[10]]}];
err1X = data1X[[11]];
p1 = Show[LogPlot[
    {PEmerB2[100, 60, R2, 11, 1, 20, 0.001]}, {R2, 105, 165},
    PlotRange → {Automatic, {0.0000001, 0.01}},
    PlotStyle → {Black, Dashed}, AxesLabel → {"R2", "Pfix"}],
  ErrorListLogPlot[{{pts1X[[1]], ErrorBar[err1X[[1]]]},
     {pts1[[1]], ErrorBar[err1[[1]]]}, {pts1X[[2]], ErrorBar[err1X[[2]]]},
     {pts1[[2]], ErrorBar[err1[[2]]]}, {pts1[[3]], ErrorBar[err1[[3]]]},
     {pts1X[[3]], ErrorBar[err1X[[3]]]}},
    PlotStyle → {Black, PointSize[0.02]}], PlotRange → All
 ]
p1X = Show[Plot[
     {PEmerB2[100, 60, R2, 11, 1, 20, 0.001]}, {R2, 105, 165},
     PlotRange → Automatic, PlotStyle → {Black, Dashed}, AxesLabel → {"R2", "Pfix"}],
    ErrorListPlot[
     {{pts1X[[1]], ErrorBar[err1X[[1]]]}, {pts1[[1]], ErrorBar[err1[[1]]]},
      {pts1X[[2]], ErrorBar[err1X[[2]]]}, {pts1[[2]], ErrorBar[err1[[2]]]},
      {pts1X[[3]], ErrorBar[err1X[[3]]]}, {pts1[[3]], ErrorBar[err1[[3]]]}},
     PlotStyle → {Black, PointSize[0.02]}], PlotRange → All
  ];
```

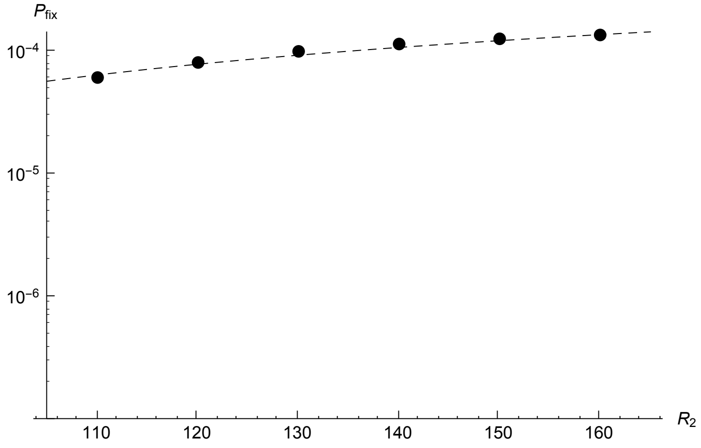

$\mu = 0.0001$

```
data1 = Transpose[Select[dataA, #[[4]] == 12 && #[[8]] == 0.0001 &]];
pts1 = Transpose[{data1[[3]], data1[[10]]}];
err1 = data1[[11]];
data1X = Transpose[Select[dataD, #[[4]] == 12 && #[[8]] == 0.0001 &]];
pts1X = Transpose[{data1X[[3]], data1X[[10]]}];
err1X = data1X[[11]];
p2 = Show[LogPlot[
    {PEmerB2[100, 60, R2, 11, 1, 20, 0.0001]}, {R2, 105, 165},
    PlotRange → {Automatic, {0.0000001, 0.01}},
    PlotStyle → {Red}, AxesLabel → {"R2", "Pfix"}],
   ErrorListLogPlot[{{pts1X[[1]], ErrorBar[err1X[[1]]]},
     {pts1[[1]], ErrorBar[err1[[1]]]}, {pts1X[[2]], ErrorBar[err1X[[2]]]},
     {pts1[[2]], ErrorBar[err1[[2]]]}, {pts1[[3]], ErrorBar[err1[[3]]]},
     {pts1X[[3]], ErrorBar[err1X[[3]]]}},
    PlotStyle → {Red, PointSize[0.02]}], PlotRange → All
  ]
p2X = Show[Plot[
     {PEmerB2[100, 60, R2, 11, 1, 20, 0.0001]}, {R2, 105, 165},
     PlotRange → Automatic, PlotStyle → {Red}, AxesLabel → {"R2", "Pfix"}],
    ErrorListPlot[
     {{pts1X[[1]], ErrorBar[err1X[[1]]]}, {pts1[[1]], ErrorBar[err1[[1]]]},
      {pts1X[[2]], ErrorBar[err1X[[2]]]}, {pts1[[2]], ErrorBar[err1[[2]]]},
      {pts1X[[3]], ErrorBar[err1X[[3]]]}, {pts1[[3]], ErrorBar[err1[[3]]]}},
     PlotStyle → {Red, PointSize[0.02]}], PlotRange → All
   ];
```

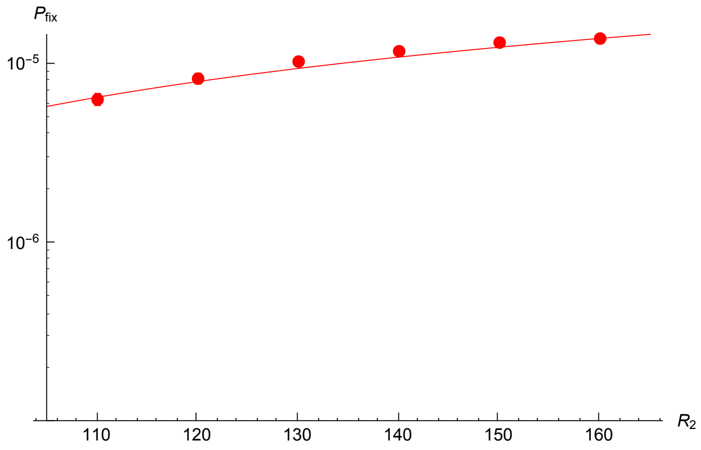

$\mu = 0.00001$

```
data1 = Transpose[Select[dataA, #[[4]] == 12 && #[[8]] == 0.00001 &]];
pts1 = Transpose[{data1[[3]], data1[[10]]}];
err1 = data1[[11]];
data1X = Transpose[Select[dataD, #[[4]] == 12 && #[[8]] == 0.00001 &]];
pts1X = Transpose[{data1X[[3]], data1X[[10]]}];
err1X = data1X[[11]];
p3 = Show[LogPlot[
    {PEmerB2[100, 60, R2, 11, 1, 20, 0.00001]}, {R2, 105, 165},
    PlotRange → {Automatic, {0.0000001, 0.01}},
    PlotStyle → {Blue}, AxesLabel → {"R2", "Pfix"}],
   ErrorListLogPlot[{{pts1X[[1]], ErrorBar[err1X[[1]]]},
     {pts1[[1]], ErrorBar[err1[[1]]]}, {pts1X[[2]], ErrorBar[err1X[[2]]]},
     {pts1[[2]], ErrorBar[err1[[2]]]}, {pts1[[3]], ErrorBar[err1[[3]]]},
     {pts1X[[3]], ErrorBar[err1X[[3]]]}},
    PlotStyle → {Blue, PointSize[0.02]}], PlotRange → All
  ]
p3X = Show[Plot[
     {PEmerB2[100, 60, R2, 11, 1, 20, 0.00001]}, {R2, 105, 165},
     PlotRange → Automatic, PlotStyle → {Blue}, AxesLabel → {"R2", "Pfix"}],
    ErrorListPlot[
     {{pts1X[[1]], ErrorBar[err1X[[1]]]}, {pts1[[1]], ErrorBar[err1[[1]]]},
      {pts1X[[2]], ErrorBar[err1X[[2]]]}, {pts1[[2]], ErrorBar[err1[[2]]]},
      {pts1X[[3]], ErrorBar[err1X[[3]]]}, {pts1[[3]], ErrorBar[err1[[3]]]}},
     PlotStyle → {Blue, PointSize[0.02]}], PlotRange → All
   ];
```

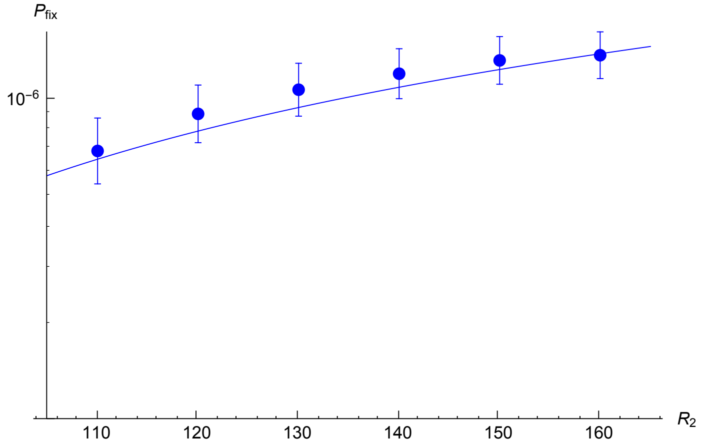

$\mu = 0.000005$

```
data1 = Transpose[Select[dataA, #[[4]] == 12 && #[[8]] == 0.000005 &]];
pts1  = Transpose[{data1[[3]], data1[[10]]}];
err1 = data1[[11]];
data1X = Transpose[Select[dataD, #[[4]] == 12 && #[[8]] == 0.000005 &]];
pts1X  = Transpose[{data1X[[3]], data1X[[10]]}];
err1X = data1X[[11]];
p4 = Show[LogPlot[
    {PEmerB2[100, 60, R2, 11, 1, 20, 0.000005]}, {R2, 105, 165},
    PlotRange → {Automatic, {0.0000001, 0.01}},
    PlotStyle → {Green}, AxesLabel → {"R2", "Pfix"}],
   ErrorListLogPlot[{{pts1X[[1]], ErrorBar[err1X[[1]]]},
     {pts1[[1]], ErrorBar[err1[[1]]]}, {pts1X[[2]], ErrorBar[err1X[[2]]]},
     {pts1[[2]], ErrorBar[err1[[2]]]}, {pts1[[3]], ErrorBar[err1[[3]]]},
     {pts1X[[3]], ErrorBar[err1X[[3]]]}},
    PlotStyle → {Green, PointSize[0.02]}], PlotRange → All
  ]
p4X = Show[Plot[
     {PEmerB2[100, 60, R2, 11, 1, 20, 0.000005]}, {R2, 105, 165},
     PlotRange → Automatic, PlotStyle → {Green}, AxesLabel → {"R2", "Pfix"}],
    ErrorListPlot[
     {{pts1X[[1]], ErrorBar[err1X[[1]]]}, {pts1[[1]], ErrorBar[err1[[1]]]},
      {pts1X[[2]], ErrorBar[err1X[[2]]]}, {pts1[[2]], ErrorBar[err1[[2]]]},
      {pts1X[[3]], ErrorBar[err1X[[3]]]}, {pts1[[3]], ErrorBar[err1[[3]]]}},
     PlotStyle → {Green, PointSize[0.02]}], PlotRange → All
   ];
```

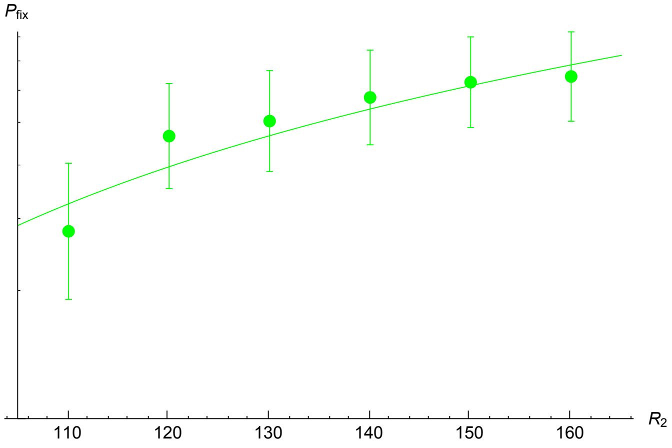

All μ together:

```
Show[p1, p2, p3, p4]
```

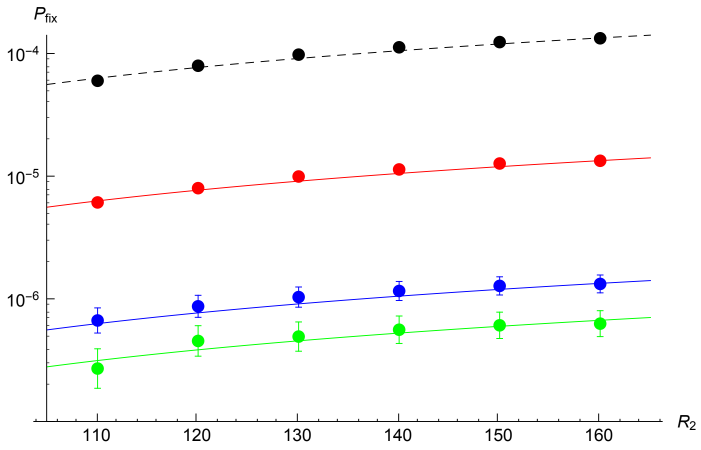


```
Show[p1X, p2X, p3X, p4X]
```

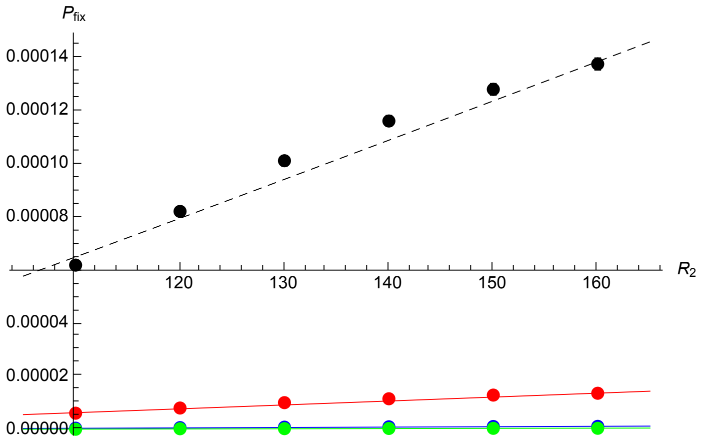

$\mu = 0.025$

```
data1 = Transpose[Select[dataB, #[[4]] == 12 && #[[8]] == 0.025 &]];
pts1  = Transpose[{data1[[3]], data1[[10]]}];
err1 = data1[[11]];
data1X = Transpose[Select[dataD, #[[4]] == 12 && #[[8]] == 0.025 &]];
pts1X  = Transpose[{data1X[[3]], data1X[[10]]}];
err1X = data1X[[11]];
p5 = Show[LogPlot[
    {PEmerB2[100, 60, R2, 11, 1, 20, 0.025]}, {R2, 105, 165},
    PlotRange → {Automatic, {0.000005, 0.01}},
    PlotStyle → {Magenta}, AxesLabel → {"R2", "Pfix"}],
  ErrorListLogPlot[{{pts1X[[1]], ErrorBar[err1X[[1]]]},
    {pts1[[1]], ErrorBar[err1[[1]]]}, {pts1X[[2]], ErrorBar[err1X[[2]]]},
    {pts1[[2]], ErrorBar[err1[[2]]]}, {pts1[[3]], ErrorBar[err1[[3]]]},
    {pts1X[[3]], ErrorBar[err1X[[3]]]}},
   PlotStyle → {Magenta, PointSize[0.02]}], PlotRange → All
 ]
p5X = Show[Plot[
    {PEmerB2[100, 60, R2, 11, 1, 20, 0.025]}, {R2, 115, 205},
    PlotRange → Automatic, PlotStyle → {Magenta}, AxesLabel → {"R2", "Pfix"}],
   ErrorListPlot[{{pts1[[1]], ErrorBar[err1[[1]]]},
     {pts1[[2]], ErrorBar[err1[[2]]]}, {pts1[[3]], ErrorBar[err1[[3]]]},
     {pts1[[4]], ErrorBar[err1[[4]]]}, {pts1[[5]], ErrorBar[err1[[5]]]}},
    PlotStyle → {Magenta, PointSize[0.02]}], PlotRange → All
  ];
```

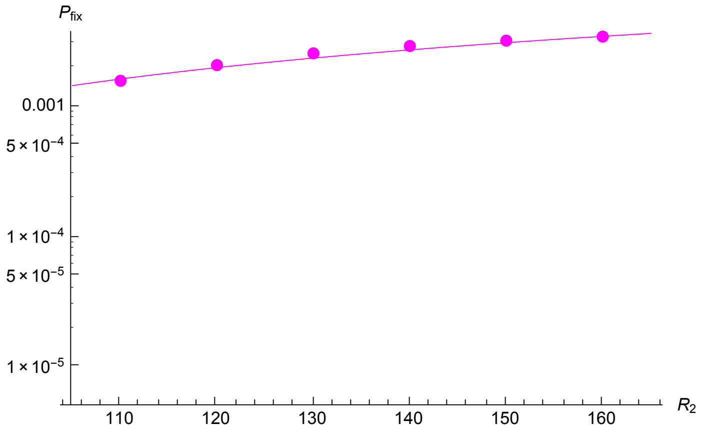

$\mu = 0.005$

```
data1 = Transpose[Select[dataB, #[[4]] == 12 && #[[8]] == 0.005 &]];
pts1 = Transpose[{data1[[3]], data1[[10]]}];
err1 = data1[[11]];
data1X = Transpose[Select[dataD, #[[4]] == 12 && #[[8]] == 0.005 &]];
pts1X = Transpose[{data1X[[3]], data1X[[10]]}];
err1X = data1X[[11]];
p6 = Show[LogPlot[
    {PEmerB2[100, 60, R2, 11, 1, 20, 0.005]}, {R2, 105, 165},
    PlotRange → {Automatic, {0.000005, 0.01}},
    PlotStyle → {Orange}, AxesLabel → {"R₂", "Pfix"}],
   ErrorListLogPlot[{{pts1X[[1]], ErrorBar[err1X[[1]]]},
     {pts1[[1]], ErrorBar[err1[[1]]]}, {pts1X[[2]], ErrorBar[err1X[[2]]]},
     {pts1[[2]], ErrorBar[err1[[2]]]}, {pts1[[3]], ErrorBar[err1[[3]]]},
     {pts1X[[3]], ErrorBar[err1X[[3]]]}},
    PlotStyle → {Orange, PointSize[0.02]}], PlotRange → All
  ]
p6X = Show[Plot[
     {PEmerB2[100, 60, R2, 11, 1, 20, 0.005]}, {R2, 115, 205},
     PlotRange → Automatic, PlotStyle → {Orange}, AxesLabel → {"R₂", "Pfix"}],
    ErrorListPlot[{{pts1[[1]], ErrorBar[err1[[1]]]},
      {pts1[[2]], ErrorBar[err1[[2]]]}, {pts1[[3]], ErrorBar[err1[[3]]]},
      {pts1[[4]], ErrorBar[err1[[4]]]}, {pts1[[5]], ErrorBar[err1[[5]]]}},
     PlotStyle → {Orange, PointSize[0.02]}], PlotRange → All
   ];
```

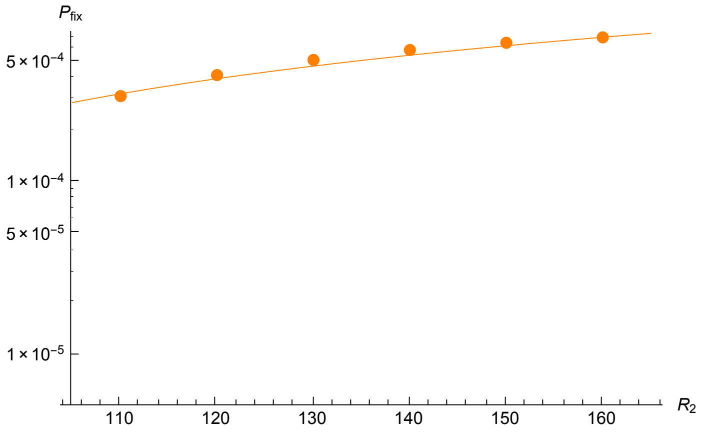

$\mu = 0.0025$

```
data1 = Transpose[Select[dataB, #[[4]] == 12 && #[[8]] == 0.0025 &]];
pts1 = Transpose[{data1[[3]], data1[[10]]}];
err1 = data1[[11]];
data1X = Transpose[Select[dataD, #[[4]] == 12 && #[[8]] == 0.0025 &]];
pts1X = Transpose[{data1X[[3]], data1X[[10]]}];
err1X = data1X[[11]];
p7 = Show[LogPlot[
   {PEmerB2[100, 60, R2, 11, 1, 20, 0.0025]}, {R2, 105, 165},
   PlotRange → {Automatic, {0.000005, 0.01}},
   PlotStyle → {Brown}, AxesLabel → {"R₂", "Pfix"}],
  ErrorListLogPlot[{{pts1X[[1]], ErrorBar[err1X[[1]]]},
    {pts1[[1]], ErrorBar[err1[[1]]]}, {pts1X[[2]], ErrorBar[err1X[[2]]]},
    {pts1[[2]], ErrorBar[err1[[2]]]}, {pts1[[3]], ErrorBar[err1[[3]]]},
    {pts1X[[3]], ErrorBar[err1X[[3]]]}},
   PlotStyle → {Brown, PointSize[0.02]}], PlotRange → All
 ]
p7X = Show[Plot[
    {PEmerB2[100, 60, R2, 11, 1, 20, 0.0025]}, {R2, 115, 205},
    PlotRange → Automatic, PlotStyle → {Brown}, AxesLabel → {"R₂", "Pfix"}],
   ErrorListPlot[{{pts1[[1]], ErrorBar[err1[[1]]]},
     {pts1[[2]], ErrorBar[err1[[2]]]}, {pts1[[3]], ErrorBar[err1[[3]]]},
     {pts1[[4]], ErrorBar[err1[[4]]]}, {pts1[[5]], ErrorBar[err1[[5]]]}},
    PlotStyle → {Brown, PointSize[0.02]}], PlotRange → All
  ];
```

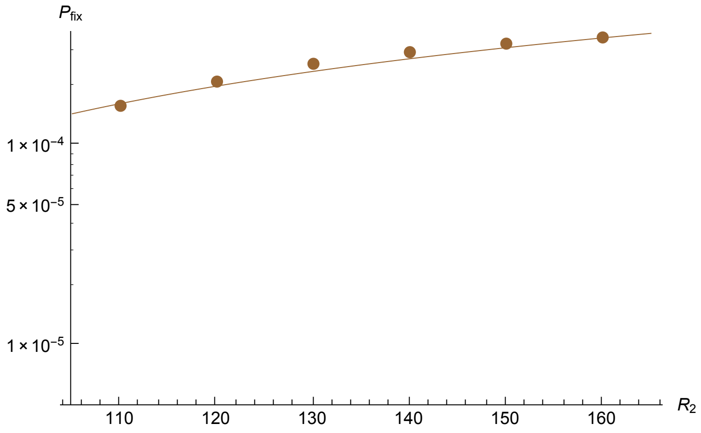

All μ together:

```
Show[p1, p2, p3, p4, p5, p6, p7]
```

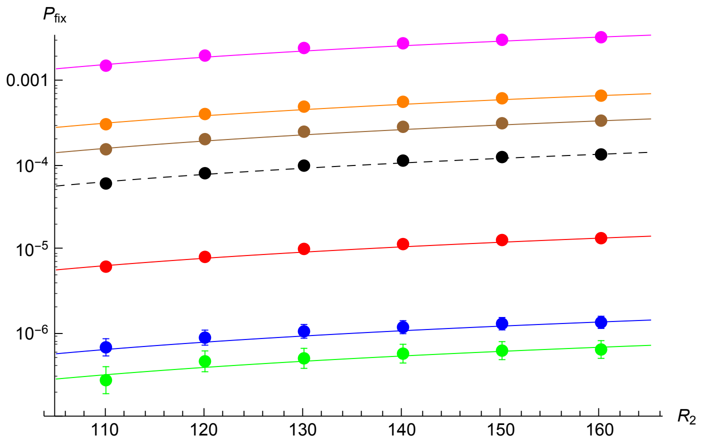

K = 250 simulations

```
dataC = Import["results_29Nov.dat", "Table"];
dataC2 = Import["results_29Nov2a.dat", "Table"];
```

ρ = 6.5

μ = 0.0025

```
data1 = Transpose[Select[dataC, #[[4]] == 7.5 && #[[8]] == 0.0025 &]];
pts1 = Transpose[{data1[[3]], data1[[10]]}];
err1 = data1[[11]];
p1 = Show[LogPlot[
   {PEmerB2[250, 100, R2, 6.5, 1, 20, 0.0025]}, {R2, 315, 405},
   PlotRange → {Automatic, {0.00025, 0.5}},
   PlotStyle → {Blue}, AxesLabel → {"R₂", "Pfix"}],
  ErrorListLogPlot[{{pts1[[1]], ErrorBar[err1[[1]]]},
    {pts1[[2]], ErrorBar[err1[[2]]]}, {pts1[[3]], ErrorBar[err1[[3]]]},
    {pts1[[4]], ErrorBar[err1[[4]]]}, {pts1[[5]], ErrorBar[err1[[5]]]}},
   PlotStyle → {Blue, PointSize[0.02]}, PlotRange → All
 ]
p1X = Show[Plot[
    {PEmerB2[250, 100, R2, 6.5, 1, 20, 0.0025]}, {R2, 315, 405},
    PlotRange → Automatic, PlotStyle → {Blue}, AxesLabel → {"R₂", "Pfix"}],
   ErrorListPlot[
    {{pts1[[1]], ErrorBar[err1[[1]]]}, {pts1[[2]], ErrorBar[err1[[2]]]},
     {pts1[[3]], ErrorBar[err1[[3]]]}, {pts1[[4]], ErrorBar[err1[[4]]]},
     {pts1[[5]], ErrorBar[err1[[5]]]}}, PlotStyle → {Blue}], PlotRange → All
  ];
```

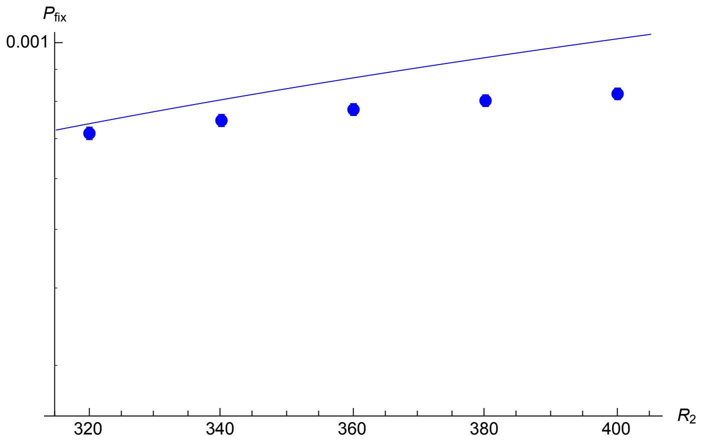

$\mu = 0.005$

```
data1 = Transpose[Select[dataC, #[[4]] == 7.5 && #[[8]] == 0.005 &]];
pts1 = Transpose[{data1[[3]], data1[[10]]}];
err1 = data1[[11]];
p2 = Show[LogPlot[
    {PEmerB2[250, 100, R2, 6.5, 1, 20, 0.005]}, {R2, 315, 405},
    PlotRange → {Automatic, {0.00025, 0.5}},
    PlotStyle → {Red}, AxesLabel → {"R2", "Pfix"}],
   ErrorListLogPlot[{{pts1[[1]], ErrorBar[err1[[1]]]},
     {pts1[[2]], ErrorBar[err1[[2]]]}, {pts1[[3]], ErrorBar[err1[[3]]]},
     {pts1[[4]], ErrorBar[err1[[4]]]}, {pts1[[5]], ErrorBar[err1[[5]]]}},
    PlotStyle → {Red, PointSize[0.02]}], PlotRange → All
  ]
p2X = Show[Plot[
     {PEmerB2[250, 100, R2, 6.5, 1, 20, 0.005]}, {R2, 315, 405},
     PlotRange → Automatic, PlotStyle → {Red}, AxesLabel → {"R2", "Pfix"}],
    ErrorListPlot[
     {{pts1[[1]], ErrorBar[err1[[1]]]}, {pts1[[2]], ErrorBar[err1[[2]]]},
      {pts1[[3]], ErrorBar[err1[[3]]]}, {pts1[[4]], ErrorBar[err1[[4]]]},
      {pts1[[5]], ErrorBar[err1[[5]]]}}, PlotStyle → {Red}], PlotRange → All
   ];
```

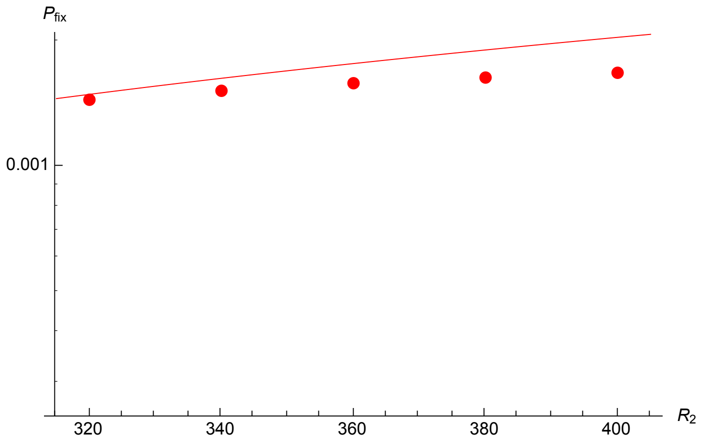

$\mu = 0.001$

```
data1 = Transpose[Select[dataC, #[[4]] == 7.5 && #[[8]] == 0.001 &]];
pts1 = Transpose[{data1[[3]], data1[[10]]}];
err1 = data1[[11]];
p3 = Show[LogPlot[
    {PEmerB2[250, 100, R2, 6.5, 1, 20, 0.001]}, {R2, 315, 405},
    PlotRange → {Automatic, {0.00025, 0.5}},
    PlotStyle → {Black, Dashed}, AxesLabel → {"R2", "Pfix"}],
  ErrorListLogPlot[{{pts1[[1]], ErrorBar[err1[[1]]]},
     {pts1[[2]], ErrorBar[err1[[2]]]}, {pts1[[3]], ErrorBar[err1[[3]]]},
     {pts1[[4]], ErrorBar[err1[[4]]]}, {pts1[[5]], ErrorBar[err1[[5]]]}},
    PlotStyle → {Black, PointSize[0.02]}], PlotRange → All
 ]
p3X = Show[Plot[
     {PEmerB2[250, 100, R2, 6.5, 1, 20, 0.001]}, {R2, 315, 405},
     PlotRange → Automatic, PlotStyle → {Black}, AxesLabel → {"R2", "Pfix"}],
    ErrorListPlot[
     {{pts1[[1]], ErrorBar[err1[[1]]]}, {pts1[[2]], ErrorBar[err1[[2]]]},
      {pts1[[3]], ErrorBar[err1[[3]]]}, {pts1[[4]], ErrorBar[err1[[4]]]},
      {pts1[[5]], ErrorBar[err1[[5]]]}}, PlotStyle → {Black}], PlotRange → All
  ];
```

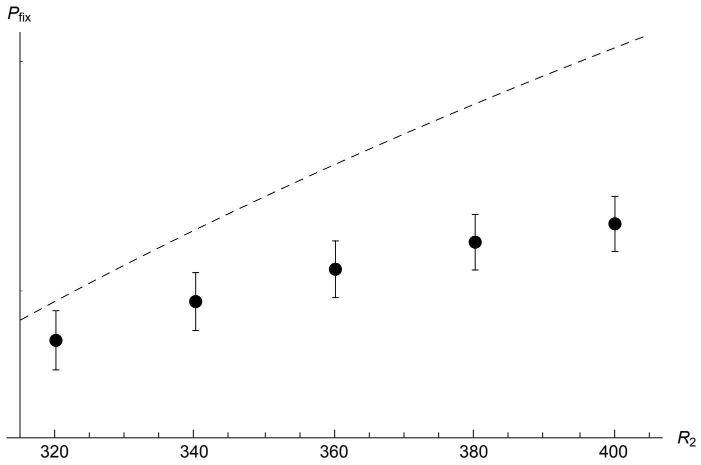

All μ together:

```
Show[p1, p2, p3]
```

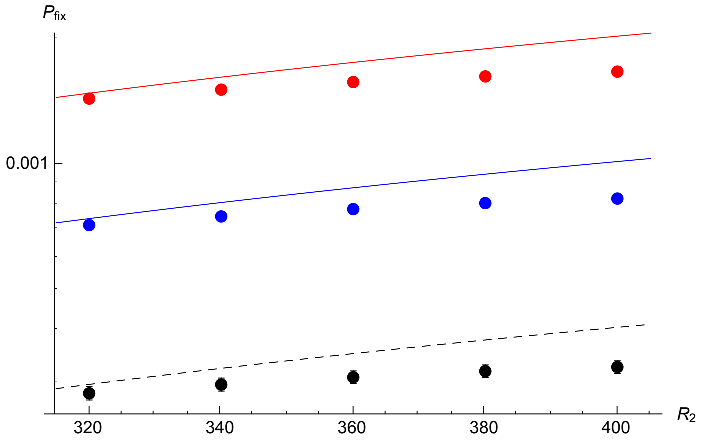

ρ = 9

μ = 0.001

```
data1 = Transpose[Select[dataC, #[[4]] == 10 && #[[8]] == 0.001 &]];
pts1 = Transpose[{data1[[3]], data1[[10]]}];
err1 = data1[[11]];
p1 = Show[LogPlot[
    {PEmerB2[250, 100, R2, 9, 1, 20, 0.001]}, {R2, 315, 405},
    PlotRange → {Automatic, {0.0001, 0.005}},
    PlotStyle → {Black, Dashed}, AxesLabel → {"R2", "Pfix"}],
  ErrorListLogPlot[{{pts1[[1]], ErrorBar[err1[[1]]]},
     {pts1[[2]], ErrorBar[err1[[2]]]}, {pts1[[3]], ErrorBar[err1[[3]]]},
     {pts1[[4]], ErrorBar[err1[[4]]]}, {pts1[[5]], ErrorBar[err1[[5]]]}},
    PlotStyle → {Black, PointSize[0.02]}], PlotRange → All
 ]
p1X = Show[Plot[
     {PEmerB2[250, 100, R2, 9, 1, 20, 0.001]}, {R2, 315, 405},
     PlotRange → Automatic, PlotStyle → {Black, Dashed}, AxesLabel → {"R2", "Pfix"}],
    ErrorListPlot[
     {{pts1[[1]], ErrorBar[err1[[1]]]}, {pts1[[2]], ErrorBar[err1[[2]]]},
      {pts1[[3]], ErrorBar[err1[[3]]]}, {pts1[[4]], ErrorBar[err1[[4]]]},
      {pts1[[5]], ErrorBar[err1[[5]]]}}, PlotStyle → {Black}], PlotRange → All
  ];
```

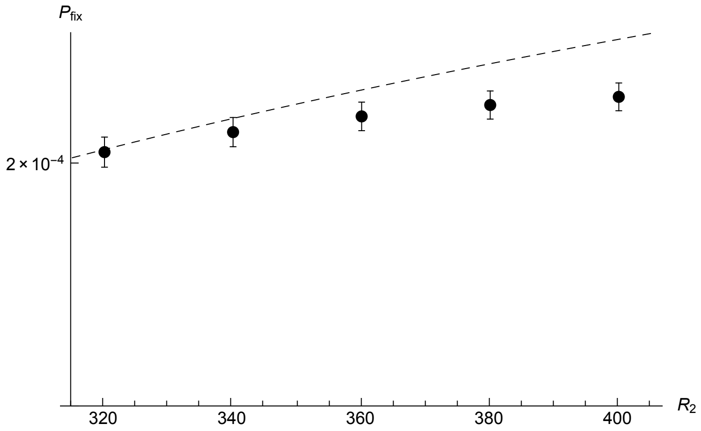

μ = 0.005

```
data1 = Transpose[Select[dataC, #[[4]] == 10 && #[[8]] == 0.005 &]];
pts1  = Transpose[{data1[[3]], data1[[10]]}];
err1 = data1[[11]];
p2 = Show[LogPlot[
    {PEmerB2[250, 100, R2, 9, 1, 20, 0.005]}, {R2, 315, 405},
    PlotRange → {Automatic, {0.0001, 0.005}},
    PlotStyle → {Red}, AxesLabel → {"R2", "Pfix"}],
   ErrorListLogPlot[{{pts1[[1]], ErrorBar[err1[[1]]]},
     {pts1[[2]], ErrorBar[err1[[2]]]}, {pts1[[3]], ErrorBar[err1[[3]]]},
     {pts1[[4]], ErrorBar[err1[[4]]]}, {pts1[[5]], ErrorBar[err1[[5]]]}},
    PlotStyle → {Red, PointSize[0.02]}], PlotRange → All
  ]
p2X = Show[Plot[
     {PEmerB2[250, 100, R2, 9, 1, 20, 0.005]}, {R2, 315, 405},
     PlotRange → Automatic, PlotStyle → {Red}, AxesLabel → {"R2", "Pfix"}],
    ErrorListPlot[
     {{pts1[[1]], ErrorBar[err1[[1]]]}, {pts1[[2]], ErrorBar[err1[[2]]]},
      {pts1[[3]], ErrorBar[err1[[3]]]}, {pts1[[4]], ErrorBar[err1[[4]]]},
      {pts1[[5]], ErrorBar[err1[[5]]]}}, PlotStyle → {Red}], PlotRange → All
   ];
```

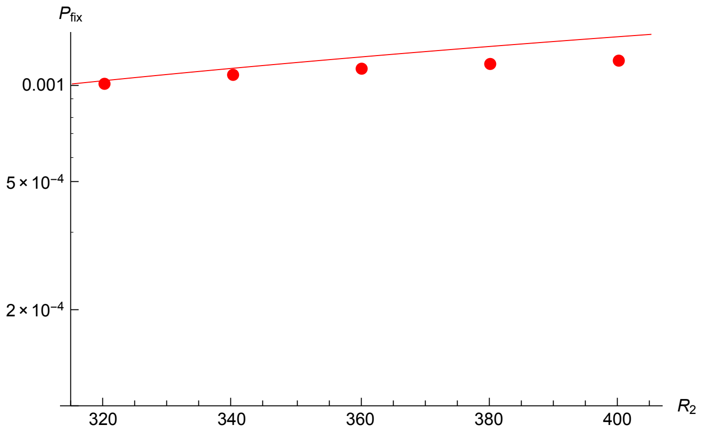

$\mu = 0.0025$

```
data1 = Transpose[Select[dataC, #[[4]] == 10 && #[[8]] == 0.0025 &]];
pts1 = Transpose[{data1[[3]], data1[[10]]}];
err1 = data1[[11]];
p3 = Show[LogPlot[
    {PEmerB2[250, 100, R2, 9, 1, 20, 0.0025]}, {R2, 315, 405},
    PlotRange → {Automatic, {0.0001, 0.005}},
    PlotStyle → {Blue}, AxesLabel → {"R₂", "Pfix"}],
  ErrorListLogPlot[{{pts1[[1]], ErrorBar[err1[[1]]]},
     {pts1[[2]], ErrorBar[err1[[2]]]}, {pts1[[3]], ErrorBar[err1[[3]]]},
     {pts1[[4]], ErrorBar[err1[[4]]]}, {pts1[[5]], ErrorBar[err1[[5]]]}},
    PlotStyle → {Blue, PointSize[0.02]}], PlotRange → All
 ]
p3X = Show[Plot[
     {PEmerB2[250, 100, R2, 9, 1, 20, 0.0025]}, {R2, 315, 405},
     PlotRange → Automatic, PlotStyle → {Blue}, AxesLabel → {"R₂", "Pfix"}],
    ErrorListPlot[
     {{pts1[[1]], ErrorBar[err1[[1]]]}, {pts1[[2]], ErrorBar[err1[[2]]]},
      {pts1[[3]], ErrorBar[err1[[3]]]}, {pts1[[4]], ErrorBar[err1[[4]]]},
      {pts1[[5]], ErrorBar[err1[[5]]]}}, PlotStyle → {Blue}], PlotRange → All
  ];
```

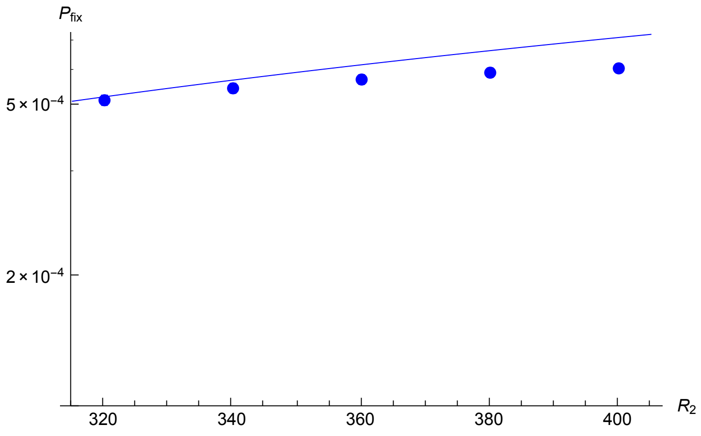

All μ together:

```
Show[p1, p2, p3]
```

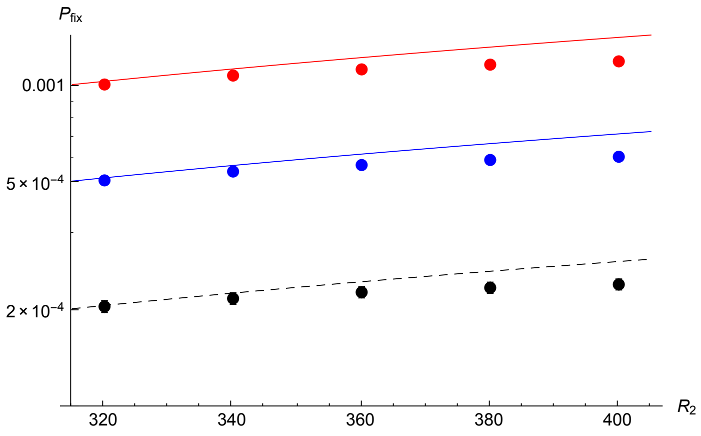

ρ = 11

μ = 0.001

```
data1 = Transpose[Select[dataC, #[[4]] == 12 && #[[8]] == 0.001 &]];
pts1  = Transpose[{data1[[3]], data1[[10]]}];
err1 = data1[[11]];
p1 = Show[LogPlot[
    {PEmerB2[250, 100, R2, 11, 1, 20, 0.001]}, {R2, 315, 405},
    PlotRange → {Automatic, {0.0001, 0.005}},
    PlotStyle → {Black, Dashed}, AxesLabel → {"R2", "Pfix"}],
  ErrorListLogPlot[{{pts1[[1]], ErrorBar[err1[[1]]]},
     {pts1[[2]], ErrorBar[err1[[2]]]}, {pts1[[3]], ErrorBar[err1[[3]]]},
     {pts1[[4]], ErrorBar[err1[[4]]]}, {pts1[[5]], ErrorBar[err1[[5]]]}},
    PlotStyle → {Black, PointSize[0.02]}], PlotRange → All
 ]
p1X = Show[Plot[
     {PEmerB2[250, 100, R2, 11, 1, 20, 0.001]}, {R2, 315, 405},
     PlotRange → Automatic, PlotStyle → {Black, Dashed}, AxesLabel → {"R2", "Pfix"}],
    ErrorListPlot[
     {{pts1[[1]], ErrorBar[err1[[1]]]}, {pts1[[2]], ErrorBar[err1[[2]]]},
      {pts1[[3]], ErrorBar[err1[[3]]]}, {pts1[[4]], ErrorBar[err1[[4]]]},
      {pts1[[5]], ErrorBar[err1[[5]]]}}, PlotStyle → {Black}], PlotRange → All
  ];
```

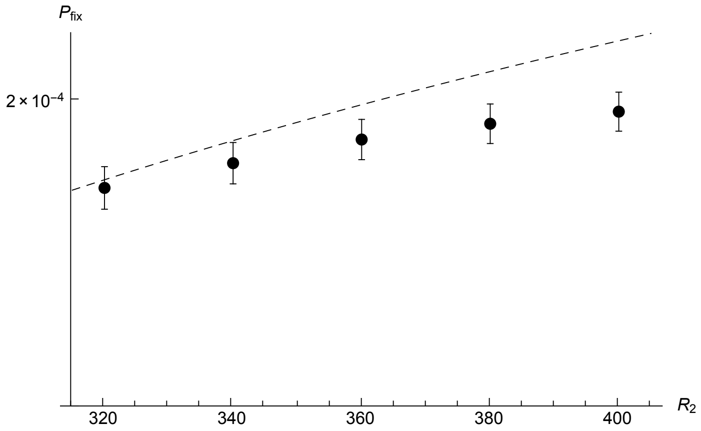

μ = 0.005

```
data1 = Transpose[Select[dataC, #[[4]] == 12 && #[[8]] == 0.005 &]];
pts1 = Transpose[{data1[[3]], data1[[10]]}];
err1 = data1[[11]];
p2 = Show[LogPlot[
    {PEmerB2[250, 100, R2, 11, 1, 20, 0.005]}, {R2, 315, 405},
    PlotRange → {Automatic, {0.0001, 0.005}},
    PlotStyle → {Red}, AxesLabel → {"R2", "Pfix"}],
  ErrorListLogPlot[{{pts1[[1]], ErrorBar[err1[[1]]]},
     {pts1[[2]], ErrorBar[err1[[2]]]}, {pts1[[3]], ErrorBar[err1[[3]]]},
     {pts1[[4]], ErrorBar[err1[[4]]]}, {pts1[[5]], ErrorBar[err1[[5]]]}},
    PlotStyle → {Red, PointSize[0.02]}], PlotRange → All
 ]
p2X = Show[Plot[
     {PEmerB2[250, 100, R2, 11, 1, 20, 0.005]}, {R2, 315, 405},
     PlotRange → Automatic, PlotStyle → {Red}, AxesLabel → {"R2", "Pfix"}],
    ErrorListPlot[
     {{pts1[[1]], ErrorBar[err1[[1]]]}, {pts1[[2]], ErrorBar[err1[[2]]]},
      {pts1[[3]], ErrorBar[err1[[3]]]}, {pts1[[4]], ErrorBar[err1[[4]]]},
      {pts1[[5]], ErrorBar[err1[[5]]]}}, PlotStyle → {Red}], PlotRange → All
  ];
```

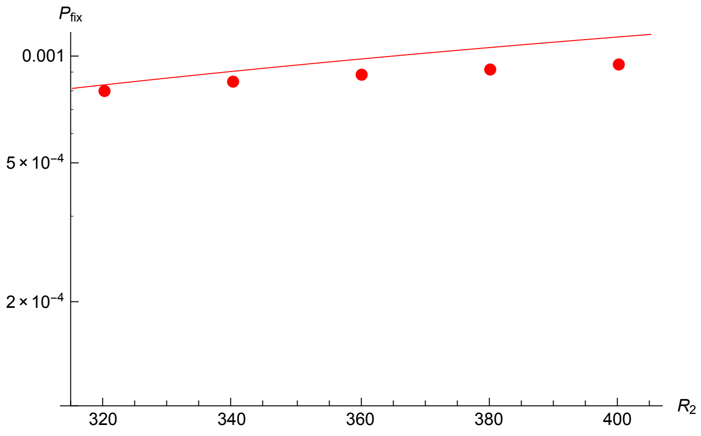

$\mu = 0.0025$

```
data1 = Transpose[Select[dataC, #[[4]] == 12 && #[[8]] == 0.0025 &]];
pts1  = Transpose[{data1[[3]], data1[[10]]}];
err1 = data1[[11]];
p3 = Show[LogPlot[
   {PEmerB2[250, 100, R2, 11, 1, 20, 0.0025]}, {R2, 315, 405},
   PlotRange → {Automatic, {0.0001, 0.005}},
   PlotStyle → {Blue}, AxesLabel → {"R₂", "Pfix"}],
  ErrorListLogPlot[{{pts1[[1]], ErrorBar[err1[[1]]]},
    {pts1[[2]], ErrorBar[err1[[2]]]}, {pts1[[3]], ErrorBar[err1[[3]]]},
    {pts1[[4]], ErrorBar[err1[[4]]]}, {pts1[[5]], ErrorBar[err1[[5]]]}},
   PlotStyle → {Blue, PointSize[0.02]}], PlotRange → All
 ]
p3X = Show[Plot[
    {PEmerB2[250, 100, R2, 11, 1, 20, 0.0025]}, {R2, 315, 405},
    PlotRange → Automatic, PlotStyle → {Blue}, AxesLabel → {"R₂", "Pfix"}],
   ErrorListPlot[
    {{pts1[[1]], ErrorBar[err1[[1]]]}, {pts1[[2]], ErrorBar[err1[[2]]]},
     {pts1[[3]], ErrorBar[err1[[3]]]}, {pts1[[4]], ErrorBar[err1[[4]]]},
     {pts1[[5]], ErrorBar[err1[[5]]]}}, PlotStyle → {Blue}], PlotRange → All
  ];
```

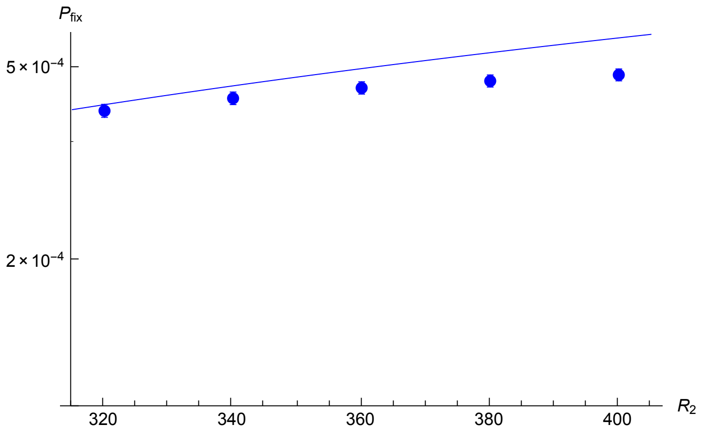

All μ together:

```
Show[p1, p2, p3]
```

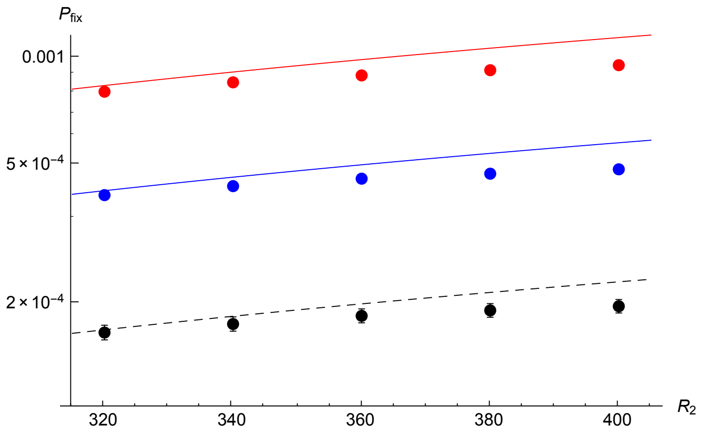

ρ = 14

μ = 0.001

```
data1 = Transpose[Select[dataC2, #[[4]] == 15 && #[[8]] == 0.001 &]];
pts1 = Transpose[{data1[[3]], data1[[10]]}];
err1 = data1[[11]];
p1 = Show[LogPlot[
    {PEmerB2[250, 100, R2, 14, 1, 20, 0.001]}, {R2, 315, 405},
    PlotRange → {Automatic, {0.0001, 0.005}},
    PlotStyle → {Black, Dashed}, AxesLabel → {"R2", "Pfix"}],
   ErrorListLogPlot[{{pts1[[1]], ErrorBar[err1[[1]]]},
     {pts1[[2]], ErrorBar[err1[[2]]]}, {pts1[[3]], ErrorBar[err1[[3]]]},
     {pts1[[4]], ErrorBar[err1[[4]]]}, {pts1[[5]], ErrorBar[err1[[5]]]}},
    PlotStyle → {Black, PointSize[0.02]}], PlotRange → All
  ]
p1X = Show[Plot[
     {PEmerB2[250, 100, R2, 14, 1, 20, 0.001]}, {R2, 315, 405},
     PlotRange → Automatic, PlotStyle → {Black, Dashed}, AxesLabel → {"R2", "Pfix"}],
    ErrorListPlot[
     {{pts1[[1]], ErrorBar[err1[[1]]]}, {pts1[[2]], ErrorBar[err1[[2]]]},
      {pts1[[3]], ErrorBar[err1[[3]]]}, {pts1[[4]], ErrorBar[err1[[4]]]},
      {pts1[[5]], ErrorBar[err1[[5]]]}}, PlotStyle → {Black}], PlotRange → All
   ];
```

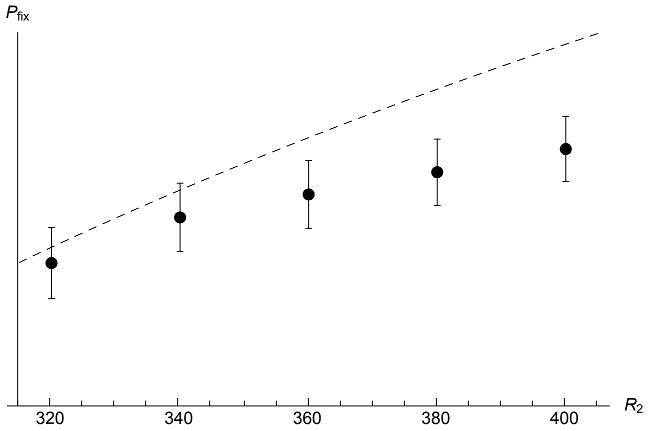

μ = 0.005

```
data1 = Transpose[Select[dataC2, #[[4]] == 15 && #[[8]] == 0.005 &]];
pts1 = Transpose[{data1[[3]], data1[[10]]}];
err1 = data1[[11]];
p2 = Show[LogPlot[
    {PEmerB2[250, 100, R2, 14, 1, 20, 0.005]}, {R2, 315, 405},
    PlotRange → {Automatic, {0.0001, 0.005}},
    PlotStyle → {Red}, AxesLabel → {"R2", "Pfix"}],
  ErrorListLogPlot[{{pts1[[1]], ErrorBar[err1[[1]]]},
     {pts1[[2]], ErrorBar[err1[[2]]]}, {pts1[[3]], ErrorBar[err1[[3]]]},
     {pts1[[4]], ErrorBar[err1[[4]]]}, {pts1[[5]], ErrorBar[err1[[5]]]}},
    PlotStyle → {Red, PointSize[0.02]}], PlotRange → All
 ]
p2X = Show[Plot[
     {PEmerB2[250, 100, R2, 14, 1, 20, 0.005]}, {R2, 315, 405},
     PlotRange → Automatic, PlotStyle → {Red}, AxesLabel → {"R2", "Pfix"}],
    ErrorListPlot[
     {{pts1[[1]], ErrorBar[err1[[1]]]}, {pts1[[2]], ErrorBar[err1[[2]]]},
      {pts1[[3]], ErrorBar[err1[[3]]]}, {pts1[[4]], ErrorBar[err1[[4]]]},
      {pts1[[5]], ErrorBar[err1[[5]]]}}, PlotStyle → {Red}], PlotRange → All
  ];
```

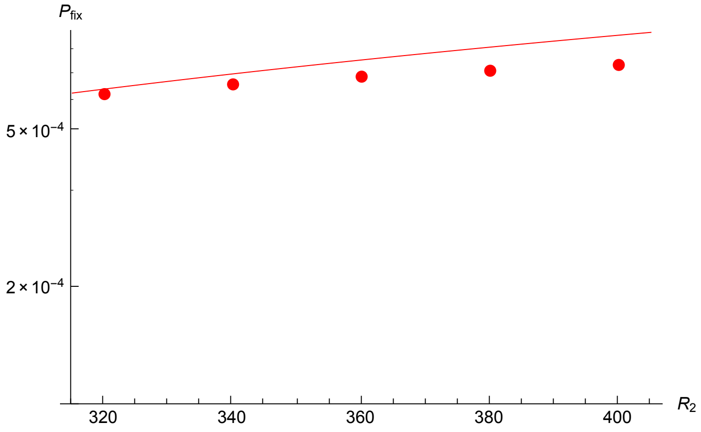

$\mu = 0.0025$

```
data1 = Transpose[Select[dataC2, #[[4]] == 15 && #[[8]] == 0.0025 &]];
pts1 = Transpose[{data1[[3]], data1[[10]]}];
err1 = data1[[11]];
p3 = Show[LogPlot[
    {PEmerB2[250, 100, R2, 14, 1, 20, 0.0025]}, {R2, 315, 405},
    PlotRange → {Automatic, {0.0001, 0.005}},
    PlotStyle → {Blue}, AxesLabel → {"R₂", "Pfix"}],
   ErrorListLogPlot[{{pts1[[1]], ErrorBar[err1[[1]]]},
     {pts1[[2]], ErrorBar[err1[[2]]]}, {pts1[[3]], ErrorBar[err1[[3]]]},
     {pts1[[4]], ErrorBar[err1[[4]]]}, {pts1[[5]], ErrorBar[err1[[5]]]}},
    PlotStyle → {Blue, PointSize[0.02]}], PlotRange → All
  ]
p3X = Show[Plot[
     {PEmerB2[250, 100, R2, 14, 1, 20, 0.0025]}, {R2, 315, 405},
     PlotRange → Automatic, PlotStyle → {Blue}, AxesLabel → {"R₂", "Pfix"}],
    ErrorListPlot[
     {{pts1[[1]], ErrorBar[err1[[1]]]}, {pts1[[2]], ErrorBar[err1[[2]]]},
      {pts1[[3]], ErrorBar[err1[[3]]]}, {pts1[[4]], ErrorBar[err1[[4]]]},
      {pts1[[5]], ErrorBar[err1[[5]]]}}, PlotStyle → {Blue}], PlotRange → All
  ];
```

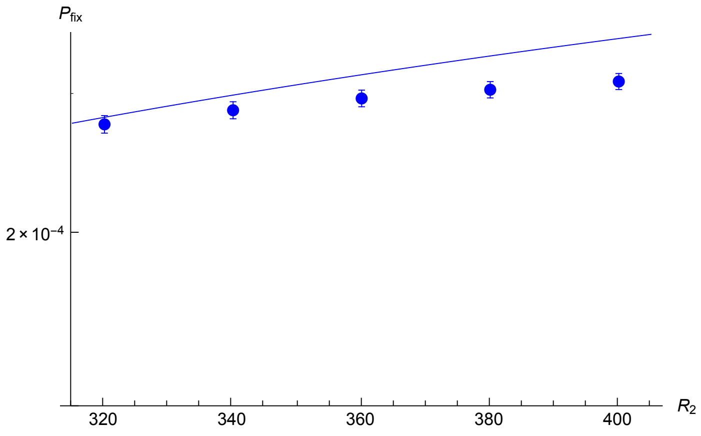

All μ together:

```
Show[p1, p2, p3]
```

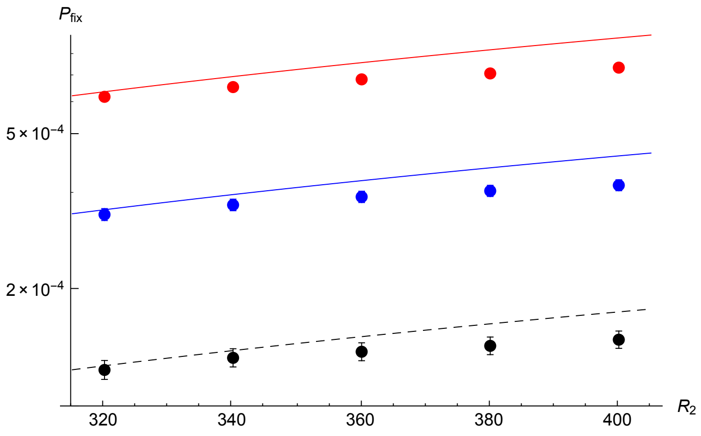

$\rho = 19$

$\mu = 0.001$

```
data1 = Transpose[Select[dataC2, #[[4]] == 20 && #[[8]] == 0.001 &]];
pts1 = Transpose[{data1[[3]], data1[[10]]}];
err1 = data1[[11]];
p1 = Show[LogPlot[
    {PEmerB2[250, 100, R2, 19, 1, 20, 0.001]}, {R2, 315, 405},
    PlotRange → {Automatic, {0.00005, 0.005}},
    PlotStyle → {Black, Dashed}, AxesLabel → {"R₂", "Pfix"}],
   ErrorListLogPlot[{{pts1[[1]], ErrorBar[err1[[1]]]},
     {pts1[[2]], ErrorBar[err1[[2]]]}, {pts1[[3]], ErrorBar[err1[[3]]]},
     {pts1[[4]], ErrorBar[err1[[4]]]}, {pts1[[5]], ErrorBar[err1[[5]]]}},
    PlotStyle → {Black, PointSize[0.02]}], PlotRange → All
  ]
p1X = Show[Plot[
     {PEmerB2[250, 100, R2, 19, 1, 20, 0.001]}, {R2, 315, 405},
     PlotRange → Automatic, PlotStyle → {Black, Dashed}, AxesLabel → {"R₂", "Pfix"}],
    ErrorListPlot[
     {{pts1[[1]], ErrorBar[err1[[1]]]}, {pts1[[2]], ErrorBar[err1[[2]]]},
      {pts1[[3]], ErrorBar[err1[[3]]]}, {pts1[[4]], ErrorBar[err1[[4]]]},
      {pts1[[5]], ErrorBar[err1[[5]]]}}, PlotStyle → {Black}], PlotRange → All
  ];
```

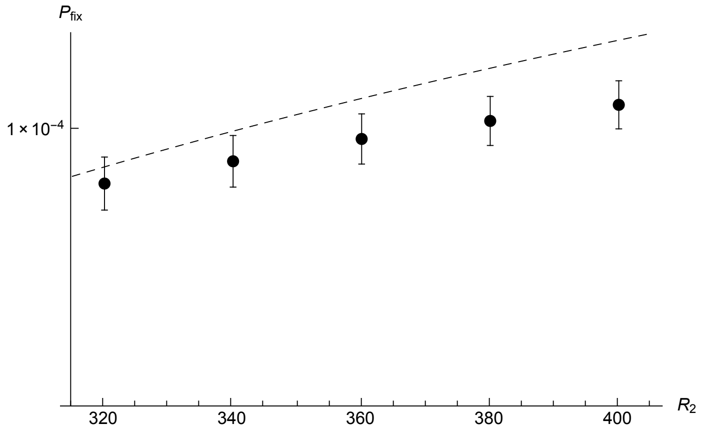

$\mu = 0.005$

```
data1 = Transpose[Select[dataC2, #[[4]] == 20 && #[[8]] == 0.005 &]];
pts1 = Transpose[{data1[[3]], data1[[10]]}];
err1 = data1[[11]];
p2 = Show[LogPlot[
    {PEmerB2[250, 100, R2, 19, 1, 20, 0.005]}, {R2, 315, 405},
    PlotRange → {Automatic, {0.00005, 0.005}},
    PlotStyle → {Red}, AxesLabel → {"R₂", "Pfix"}],
   ErrorListLogPlot[{{pts1[[1]], ErrorBar[err1[[1]]]},
     {pts1[[2]], ErrorBar[err1[[2]]]}, {pts1[[3]], ErrorBar[err1[[3]]]},
     {pts1[[4]], ErrorBar[err1[[4]]]}, {pts1[[5]], ErrorBar[err1[[5]]]}},
    PlotStyle → {Red, PointSize[0.02]}], PlotRange → All
  ]
p2X = Show[Plot[
     {PEmerB2[250, 100, R2, 19, 1, 20, 0.005]}, {R2, 315, 405},
     PlotRange → Automatic, PlotStyle → {Red}, AxesLabel → {"R₂", "Pfix"}],
    ErrorListPlot[
     {{pts1[[1]], ErrorBar[err1[[1]]]}, {pts1[[2]], ErrorBar[err1[[2]]]},
      {pts1[[3]], ErrorBar[err1[[3]]]}, {pts1[[4]], ErrorBar[err1[[4]]]},
      {pts1[[5]], ErrorBar[err1[[5]]]}}, PlotStyle → {Red}], PlotRange → All
   ];
```

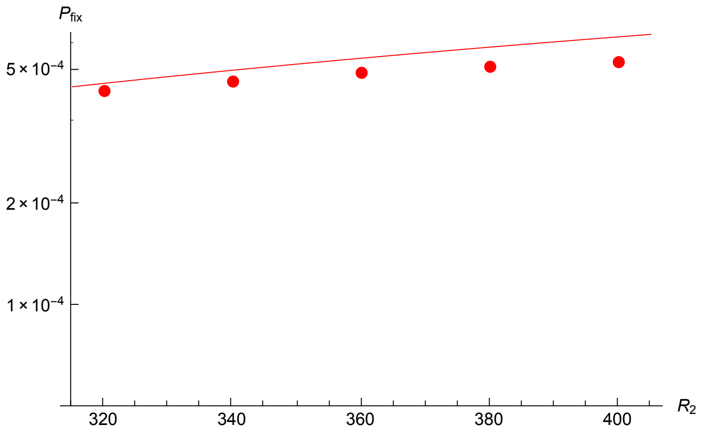

μ = 0.0025

```
data1 = Transpose[Select[dataC2, #[[4]] == 20 && #[[8]] == 0.0025 &]];
pts1 = Transpose[{data1[[3]], data1[[10]]}];
err1 = data1[[11]];
p3 = Show[LogPlot[
    {PEmerB2[250, 100, R2, 19, 1, 20, 0.0025]}, {R2, 315, 405},
    PlotRange → {Automatic, {0.00005, 0.005}},
    PlotStyle → {Blue}, AxesLabel → {"R₂", "P_fix"}],
   ErrorListLogPlot[{{pts1[[1]], ErrorBar[err1[[1]]]},
     {pts1[[2]], ErrorBar[err1[[2]]]}, {pts1[[3]], ErrorBar[err1[[3]]]},
     {pts1[[4]], ErrorBar[err1[[4]]]}, {pts1[[5]], ErrorBar[err1[[5]]]}},
    PlotStyle → {Blue, PointSize[0.02]}], PlotRange → All
  ]
p3X = Show[Plot[
     {PEmerB2[250, 100, R2, 19, 1, 20, 0.0025]}, {R2, 315, 405},
     PlotRange → Automatic, PlotStyle → {Blue}, AxesLabel → {"R₂", "P_fix"}],
    ErrorListPlot[
     {{pts1[[1]], ErrorBar[err1[[1]]]}, {pts1[[2]], ErrorBar[err1[[2]]]},
      {pts1[[3]], ErrorBar[err1[[3]]]}, {pts1[[4]], ErrorBar[err1[[4]]]},
      {pts1[[5]], ErrorBar[err1[[5]]]}}, PlotStyle → {Blue}], PlotRange → All
   ];
```

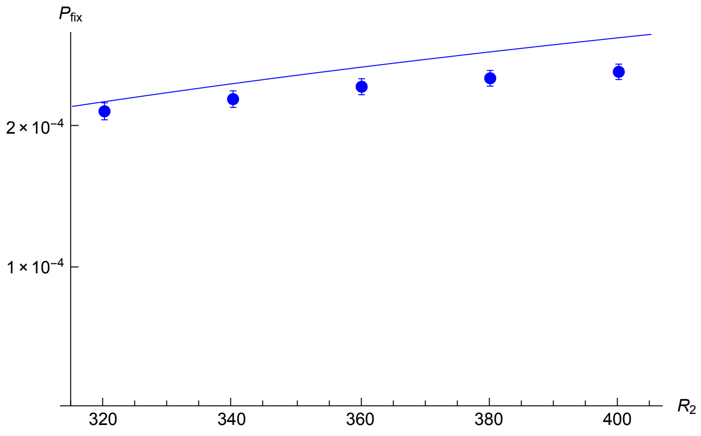

All μ together:

```
Show[p1, p2, p3]
```

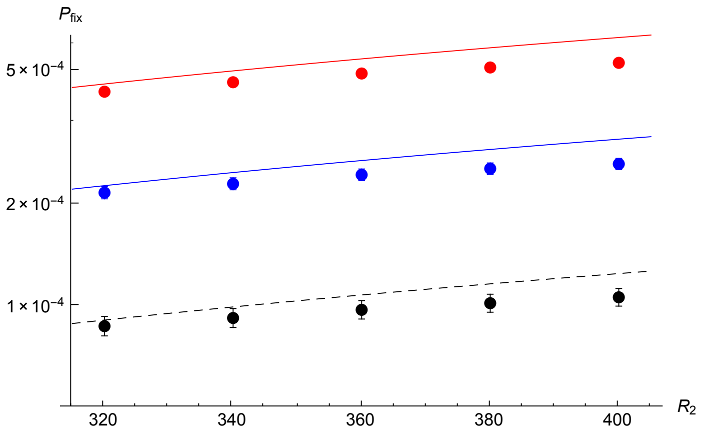

K = 1,000 simulations

```
dataF = Import["results_6thFeb.dat", "Table"];
dataF2 = Import["results_10thFeb.dat", "Table"];
```

Redefining ‘YMax’ function to change initial search value, in order to correct function for this parameter range

```
YMaxGN[Ka_, R_, R2_, ρ_, x0_, y0_] :=
 Floor[y /. FindRoot[(1 + FX3[Ka, R, ρ, x0, y0, y]) (Ka - y) y ρ + R2 == 0, {y, 2 * R}]]
```

```
YMaxN[Ka_, R_, ρ_, x0_, y0_] :=
```

$$y\ /.\ \mathrm{FindRoot}\left[x0 + \frac{1}{\rho}\left(\mathrm{Log}\left[\left(\frac{y}{y0}\right)^{R}\left(\frac{Ka - y}{Ka - y0}\right)^{Ka-R}\right]\right) == 0,\ \{y,\ 2 * R\}\right]$$

ρ = 0.5

μ = 0.025

```
data1 = Transpose[Select[dataF, #[[4]] == 1.5 && #[[8]] == 0.025 &]];
data1a = Transpose[Select[dataF2, #[[4]] == 1.5 && #[[8]] == 0.025 &]];
pts1 = Transpose[{data1[[3]], data1[[10]]}];
pts1a = Transpose[{data1a[[3]], data1a[[10]]}];
err1 = data1[[11]];
err1a = data1a[[11]];
p1 = Show[LogPlot[{PEmerB2[1000, 100, R2, 0.5, 1, 20, 0.025]}, {R2, 1110, 1310},
   PlotRange → {Automatic, {0.00001, 0.25}},
   PlotStyle → {Black, Dashed}, AxesLabel → {"R₂", "Pfix"}], ErrorListLogPlot[
   {{pts1[[1]], ErrorBar[err1[[1]]]}, {pts1[[2]], ErrorBar[err1[[2]]]},
    {pts1[[3]], ErrorBar[err1[[3]]]}, {pts1[[4]], ErrorBar[err1[[4]]]},
    {pts1[[5]], ErrorBar[err1[[5]]]}, {pts1a[[1]], ErrorBar[err1a[[1]]]},
    {pts1a[[2]], ErrorBar[err1a[[2]]]}, {pts1a[[3]], ErrorBar[err1a[[3]]]},
    {pts1a[[4]], ErrorBar[err1a[[4]]]}, {pts1a[[5]], ErrorBar[err1a[[5]]]}},
   PlotStyle → {Black, PointSize[0.02]}]
 ]
p1X = Show[Plot[
    {PEmerB2[1000, 100, R2, 0.5, 1, 20, 0.025]}, {R2, 1110, 1310},
    PlotRange → Automatic, PlotStyle → {Black}, AxesLabel → {"R₂", "Pfix"}],
   ErrorListPlot[{{pts1[[1]], ErrorBar[err1[[1]]]},
     {pts1[[2]], ErrorBar[err1[[2]]]}, {pts1[[3]], ErrorBar[err1[[3]]]},
     {pts1[[4]], ErrorBar[err1[[4]]]}, {pts1[[5]], ErrorBar[err1[[5]]]},
     {pts1a[[1]], ErrorBar[err1a[[1]]]}, {pts1a[[2]], ErrorBar[err1a[[2]]]},
     {pts1a[[3]], ErrorBar[err1a[[3]]]}, {pts1a[[4]], ErrorBar[err1a[[4]]]},
     {pts1a[[5]], ErrorBar[err1a[[5]]]}}, PlotStyle → {Black}], PlotRange → All
  ];
```

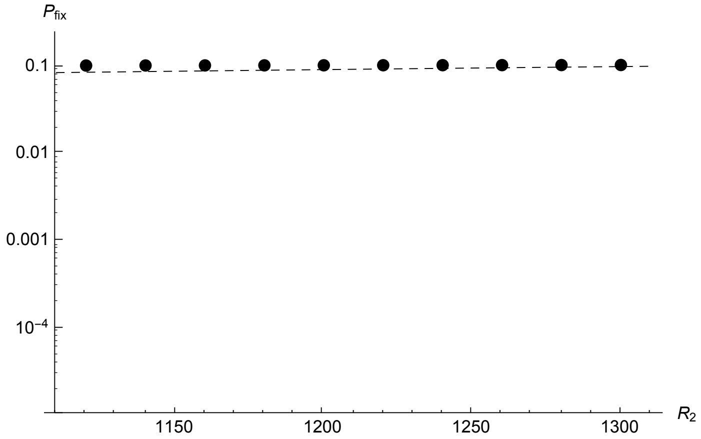

$\mu = 0.0025$

```
data1 = Transpose[Select[dataF, #[[4]] == 1.5 && #[[8]] == 0.0025 &]];
data1a = Transpose[Select[dataF2, #[[4]] == 1.5 && #[[8]] == 0.0025 &]];
pts1  = Transpose[{data1[[3]], data1[[10]]}];
pts1a  = Transpose[{data1a[[3]], data1a[[10]]}];
err1 = data1[[11]];
err1a = data1a[[11]];
p2 = Show[LogPlot[
    {PEmerB2[1000, 100, R2, 0.5, 1, 20, 0.0025]}, {R2, 1110, 1310},
    PlotRange → {Automatic, {0.00001, 0.5}},
    PlotStyle → {Red}, AxesLabel → {"R2", "Pfix"}],
  ErrorListLogPlot[{{pts1[[1]], ErrorBar[err1[[1]]]},
    {pts1[[2]], ErrorBar[err1[[2]]]}, {pts1[[3]], ErrorBar[err1[[3]]]},
    {pts1[[4]], ErrorBar[err1[[4]]]}, {pts1[[5]], ErrorBar[err1[[5]]]},
    {pts1a[[1]], ErrorBar[err1a[[1]]]}, {pts1a[[2]], ErrorBar[err1a[[2]]]},
    {pts1a[[3]], ErrorBar[err1a[[3]]]}, {pts1a[[4]], ErrorBar[err1a[[4]]]},
    {pts1a[[5]], ErrorBar[err1a[[5]]]}}, PlotStyle → {Red, PointSize[0.02]}]
 ]
p2X = Show[Plot[
    {PEmerB2[1000, 100, R2, 0.5, 1, 20, 0.0025]}, {R2, 1110, 1310},
    PlotRange → Automatic, PlotStyle → {Red}, AxesLabel → {"R2", "Pfix"}],
   ErrorListPlot[{{pts1[[1]], ErrorBar[err1[[1]]]},
     {pts1[[2]], ErrorBar[err1[[2]]]}, {pts1[[3]], ErrorBar[err1[[3]]]},
     {pts1[[4]], ErrorBar[err1[[4]]]}, {pts1[[5]], ErrorBar[err1[[5]]]},
     {pts1a[[1]], ErrorBar[err1a[[1]]]}, {pts1a[[2]], ErrorBar[err1a[[2]]]},
     {pts1a[[3]], ErrorBar[err1a[[3]]]}, {pts1a[[4]], ErrorBar[err1a[[4]]]},
     {pts1a[[5]], ErrorBar[err1a[[5]]]}}, PlotStyle → {Red}], PlotRange → All
  ];
```

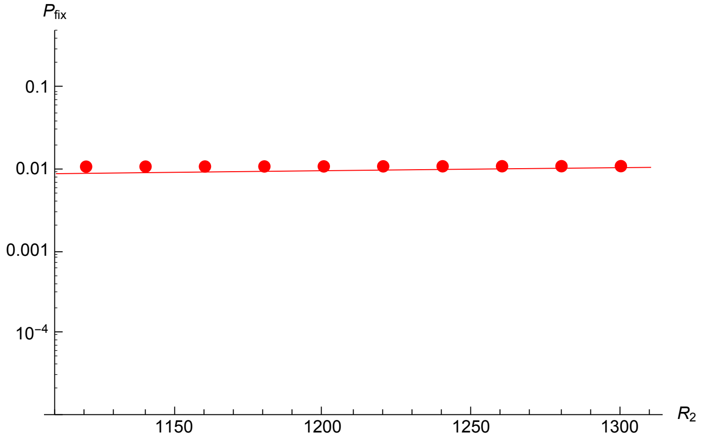

$\mu = 0.001$

```
data1 = Transpose[Select[dataF, #[[4]] == 1.5 && #[[8]] == 0.000025 &]];
data1a = Transpose[Select[dataF2, #[[4]] == 1.5 && #[[8]] == 0.000025 &]];
pts1  = Transpose[{data1[[3]], data1[[10]]}];
pts1a  = Transpose[{data1a[[3]], data1a[[10]]}];
err1 = data1[[11]];
err1a = data1a[[11]];
p3 = Show[LogPlot[
    {PEmerB2[1000, 100, R2, 0.5, 1, 20, 0.000025]}, {R2, 1110, 1310},
    PlotRange → {Automatic, {0.00001, 0.5}},
    PlotStyle → {Blue}, AxesLabel → {"R2", "Pfix"}],
  ErrorListLogPlot[{{pts1[[1]], ErrorBar[err1[[1]]]},
     {pts1[[2]], ErrorBar[err1[[2]]]}, {pts1[[3]], ErrorBar[err1[[3]]]},
     {pts1[[4]], ErrorBar[err1[[4]]]}, {pts1[[5]], ErrorBar[err1[[5]]]},
     {pts1a[[1]], ErrorBar[err1a[[1]]]}, {pts1a[[2]], ErrorBar[err1a[[2]]]},
     {pts1a[[3]], ErrorBar[err1a[[3]]]}, {pts1a[[4]], ErrorBar[err1a[[4]]]},
     {pts1a[[5]], ErrorBar[err1a[[5]]]}},
    PlotStyle → {Blue, PointSize[0.02]}], PlotRange → All
 ]
p3X = Show[Plot[
     {PEmerB2[1000, 100, R2, 0.5, 1, 20, 0.000025]}, {R2, 1110, 1310},
     PlotRange → Automatic, PlotStyle → {Blue}, AxesLabel → {"R2", "Pfix"}],
    ErrorListPlot[{{pts1[[1]], ErrorBar[err1[[1]]]},
      {pts1[[2]], ErrorBar[err1[[2]]]}, {pts1[[3]], ErrorBar[err1[[3]]]},
      {pts1[[4]], ErrorBar[err1[[4]]]}, {pts1[[5]], ErrorBar[err1[[5]]]},
      {pts1a[[1]], ErrorBar[err1a[[1]]]}, {pts1a[[2]], ErrorBar[err1a[[2]]]},
      {pts1a[[3]], ErrorBar[err1a[[3]]]}, {pts1a[[4]], ErrorBar[err1a[[4]]]},
      {pts1a[[5]], ErrorBar[err1a[[5]]]}}, PlotStyle → {Blue}], PlotRange → All
  ];
```

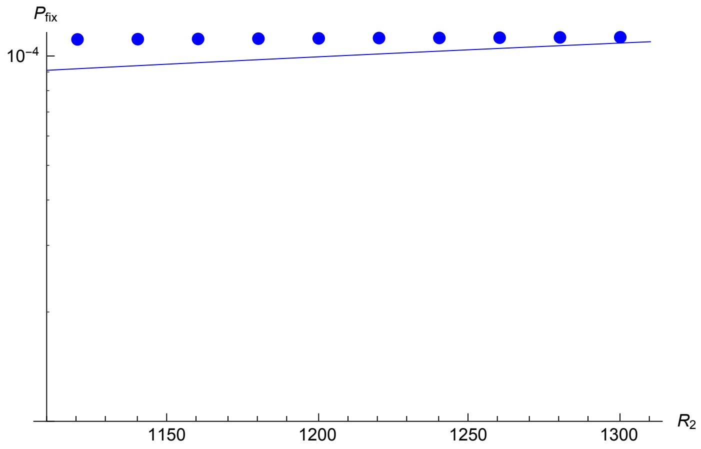

All μ together:

Show[p1, p2, p3]

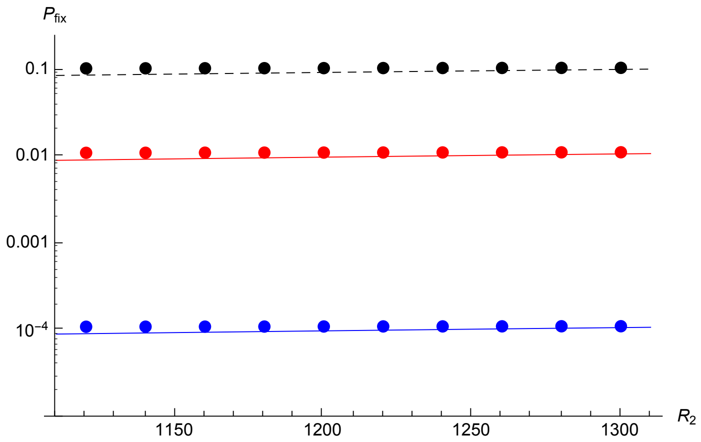

ρ = I

μ = 0.025

```
data1 = Transpose[Select[dataF, #[[4]] == 2 && #[[8]] == 0.025 &]];
data1a = Transpose[Select[dataF2, #[[4]] == 2 && #[[8]] == 0.025 &]];
pts1 = Transpose[{data1[[3]], data1[[10]]}];
pts1a = Transpose[{data1a[[3]], data1a[[10]]}];
err1 = data1[[11]];
err1a = data1a[[11]];
p1 = Show[LogPlot[
    {PEmerB2[1000, 100, R2, 1, 1, 20, 0.025]}, {R2, 1110, 1310},
    PlotRange → {Automatic, {0.000025, 0.1}},
    PlotStyle → {Black, Dashed}, AxesLabel → {"R₂", "Pfix"}],
  ErrorListLogPlot[{{pts1[[1]], ErrorBar[err1[[1]]]},
     {pts1[[2]], ErrorBar[err1[[2]]]}, {pts1[[3]], ErrorBar[err1[[3]]]},
     {pts1[[4]], ErrorBar[err1[[4]]]}, {pts1[[5]], ErrorBar[err1[[5]]]},
     {pts1a[[1]], ErrorBar[err1a[[1]]]}, {pts1a[[2]], ErrorBar[err1a[[2]]]},
     {pts1a[[3]], ErrorBar[err1a[[3]]]}, {pts1a[[4]], ErrorBar[err1a[[4]]]},
     {pts1a[[5]], ErrorBar[err1a[[5]]]}}, PlotStyle → {Black, PointSize[0.02]}]
 ]
p1X = Show[Plot[
     {PEmerB2[1000, 100, R2, 1, 1, 20, 0.025]}, {R2, 1110, 1310},
     PlotRange → Automatic, PlotStyle → Black, AxesLabel → {"R₂", "Pfix"}],
   ErrorListPlot[{{pts1[[1]], ErrorBar[err1[[1]]]},
      {pts1[[2]], ErrorBar[err1[[2]]]}, {pts1[[3]], ErrorBar[err1[[3]]]},
      {pts1[[4]], ErrorBar[err1[[4]]]}, {pts1[[5]], ErrorBar[err1[[5]]]},
      {pts1a[[1]], ErrorBar[err1a[[1]]]}, {pts1a[[2]], ErrorBar[err1a[[2]]]},
      {pts1a[[3]], ErrorBar[err1a[[3]]]}, {pts1a[[4]], ErrorBar[err1a[[4]]]},
      {pts1a[[5]], ErrorBar[err1a[[5]]]}}, PlotStyle → {Black}], PlotRange → All
  ];
```

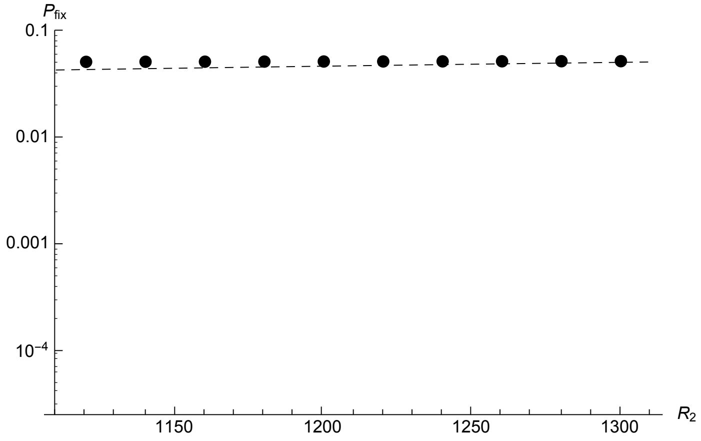

$\mu = 0.0025$

```
data1 = Transpose[Select[dataF, #[[4]] == 2 && #[[8]] == 0.0025 &]];
data1a = Transpose[Select[dataF2, #[[4]] == 2 && #[[8]] == 0.0025 &]];
pts1  = Transpose[{data1[[3]], data1[[10]]}];
pts1a =  Transpose[{data1a[[3]], data1a[[10]]}];
err1 = data1[[11]];
err1a = data1a[[11]];
p2 = Show[LogPlot[
    {PEmerB2[1000, 100, R2, 1, 1, 20, 0.0025]}, {R2, 1110, 1310},
    PlotRange → {Automatic, {0.000025, 0.1}},
    PlotStyle → {Red}, AxesLabel → {"R2", "Pfix"}],
  ErrorListLogPlot[{{pts1[[1]], ErrorBar[err1[[1]]]},
     {pts1[[2]], ErrorBar[err1[[2]]]}, {pts1[[3]], ErrorBar[err1[[3]]]},
     {pts1[[4]], ErrorBar[err1[[4]]]}, {pts1[[5]], ErrorBar[err1[[5]]]},
     {pts1a[[1]], ErrorBar[err1a[[1]]]}, {pts1a[[2]], ErrorBar[err1a[[2]]]},
     {pts1a[[3]], ErrorBar[err1a[[3]]]}, {pts1a[[4]], ErrorBar[err1a[[4]]]},
     {pts1a[[5]], ErrorBar[err1a[[5]]]}}, PlotStyle → {Red, PointSize[0.02]}]
 ]
p2X = Show[Plot[
     {PEmerB2[1000, 100, R2, 1, 1, 20, 0.0025]}, {R2, 1110, 1310},
     PlotRange → Automatic, PlotStyle → {Red}, AxesLabel → {"R2", "Pfix"}],
    ErrorListPlot[{{pts1[[1]], ErrorBar[err1[[1]]]},
      {pts1[[2]], ErrorBar[err1[[2]]]}, {pts1[[3]], ErrorBar[err1[[3]]]},
      {pts1[[4]], ErrorBar[err1[[4]]]}, {pts1[[5]], ErrorBar[err1[[5]]]},
      {pts1a[[1]], ErrorBar[err1a[[1]]]}, {pts1a[[2]], ErrorBar[err1a[[2]]]},
      {pts1a[[3]], ErrorBar[err1a[[3]]]}, {pts1a[[4]], ErrorBar[err1a[[4]]]},
      {pts1a[[5]], ErrorBar[err1a[[5]]]}}, PlotStyle → {Red}], PlotRange → All
  ];
```

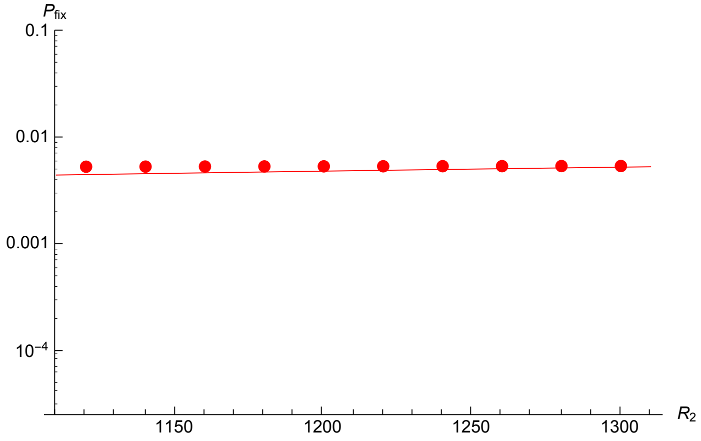

μ = 0.000025

```
data1 = Transpose[Select[dataF, #[[4]] == 2 && #[[8]] == 0.000025 &]];
data1a = Transpose[Select[dataF2, #[[4]] == 2 && #[[8]] == 0.000025 &]];
pts1 = Transpose[{data1[[3]], data1[[10]]}];
pts1a = Transpose[{data1a[[3]], data1a[[10]]}];
err1 = data1[[11]];
err1a = data1a[[11]];
p3 = Show[LogPlot[
    {PEmerB2[1000, 100, R2, 1, 1, 20, 0.000025]}, {R2, 1110, 1310},
    PlotRange → {Automatic, {0.000025, 0.1}},
    PlotStyle → {Blue}, AxesLabel → {"R₂", "Pfix"}],
  ErrorListLogPlot[{{pts1[[1]], ErrorBar[err1[[1]]]},
     {pts1[[2]], ErrorBar[err1[[2]]]}, {pts1[[3]], ErrorBar[err1[[3]]]},
     {pts1[[4]], ErrorBar[err1[[4]]]}, {pts1[[5]], ErrorBar[err1[[5]]]},
     {pts1a[[1]], ErrorBar[err1a[[1]]]}, {pts1a[[2]], ErrorBar[err1a[[2]]]},
     {pts1a[[3]], ErrorBar[err1a[[3]]]}, {pts1a[[4]], ErrorBar[err1a[[4]]]},
     {pts1a[[5]], ErrorBar[err1a[[5]]]}}, PlotStyle → {Blue, PointSize[0.02]}]
 ]
p3X = Show[Plot[
     {PEmerB2[1000, 100, R2, 1, 1, 20, 0.000025]}, {R2, 1110, 1310},
     PlotRange → Automatic, PlotStyle → Blue, AxesLabel → {"R₂", "Pfix"}],
   ErrorListPlot[{{pts1[[1]], ErrorBar[err1[[1]]]},
      {pts1[[2]], ErrorBar[err1[[2]]]}, {pts1[[3]], ErrorBar[err1[[3]]]},
      {pts1[[4]], ErrorBar[err1[[4]]]}, {pts1[[5]], ErrorBar[err1[[5]]]},
      {pts1a[[1]], ErrorBar[err1a[[1]]]}, {pts1a[[2]], ErrorBar[err1a[[2]]]},
      {pts1a[[3]], ErrorBar[err1a[[3]]]}, {pts1a[[4]], ErrorBar[err1a[[4]]]},
      {pts1a[[5]], ErrorBar[err1a[[5]]]}}, PlotStyle → {Blue}], PlotRange → All
  ];
```

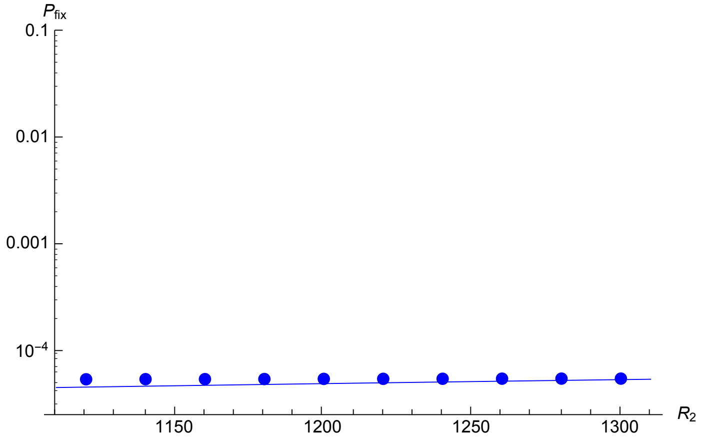

All μ together:

```
Show[p1, p2, p3]
```

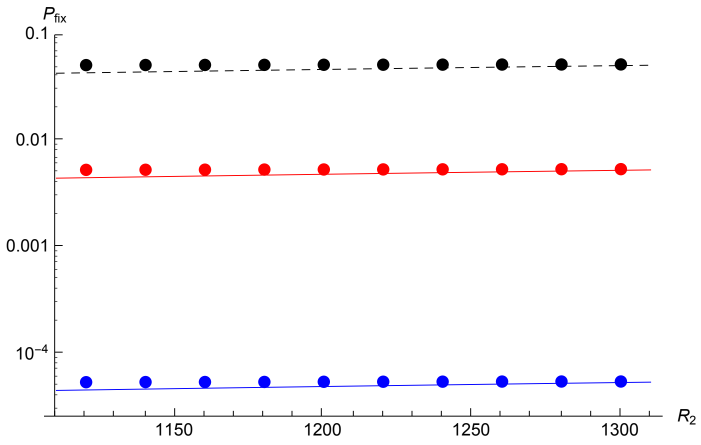

$\rho = 4$

$\mu = 0.025$

```
data1 = Transpose[Select[dataF, #[[4]] == 5 && #[[8]] == 0.025 &]];
data1a = Transpose[Select[dataF2, #[[4]] == 5 && #[[8]] == 0.025 &]];
pts1 = Transpose[{data1[[3]], data1[[10]]}];
pts1a = Transpose[{data1a[[3]], data1a[[10]]}];
err1 = data1[[11]];
err1a = data1a[[11]];
p1 = Show[LogPlot[
    {PEmerB2[1000, 100, R2, 4, 1, 20, 0.025]}, {R2, 1110, 1310},
    PlotRange → {Automatic, {0.000005, 0.05}},
    PlotStyle → {Black, Dashed}, AxesLabel → {"R2", "Pfix"}],
  ErrorListLogPlot[{{pts1[[1]], ErrorBar[err1[[1]]]},
     {pts1[[2]], ErrorBar[err1[[2]]]}, {pts1[[3]], ErrorBar[err1[[3]]]},
     {pts1[[4]], ErrorBar[err1[[4]]]}, {pts1[[5]], ErrorBar[err1[[5]]]},
     {pts1a[[1]], ErrorBar[err1a[[1]]]}, {pts1a[[2]], ErrorBar[err1a[[2]]]},
     {pts1a[[3]], ErrorBar[err1a[[3]]]}, {pts1a[[4]], ErrorBar[err1a[[4]]]},
     {pts1a[[5]], ErrorBar[err1a[[5]]]}}, PlotStyle → {Black, PointSize[0.02]}]
 ]
p1X = Show[Plot[
     {PEmerB2[1000, 100, R2, 4, 1, 20, 0.025]}, {R2, 1110, 1310},
     PlotRange → Automatic, PlotStyle → {Black, Dashed}, AxesLabel → {"R2", "Pfix"}],
    ErrorListPlot[{{pts1[[1]], ErrorBar[err1[[1]]]},
      {pts1[[2]], ErrorBar[err1[[2]]]}, {pts1[[3]], ErrorBar[err1[[3]]]},
      {pts1[[4]], ErrorBar[err1[[4]]]}, {pts1[[5]], ErrorBar[err1[[5]]]},
      {pts1a[[1]], ErrorBar[err1a[[1]]]}, {pts1a[[2]], ErrorBar[err1a[[2]]]},
      {pts1a[[3]], ErrorBar[err1a[[3]]]}, {pts1a[[4]], ErrorBar[err1a[[4]]]},
      {pts1a[[5]], ErrorBar[err1a[[5]]]}}, PlotStyle → {Black}], PlotRange → All
  ];
```

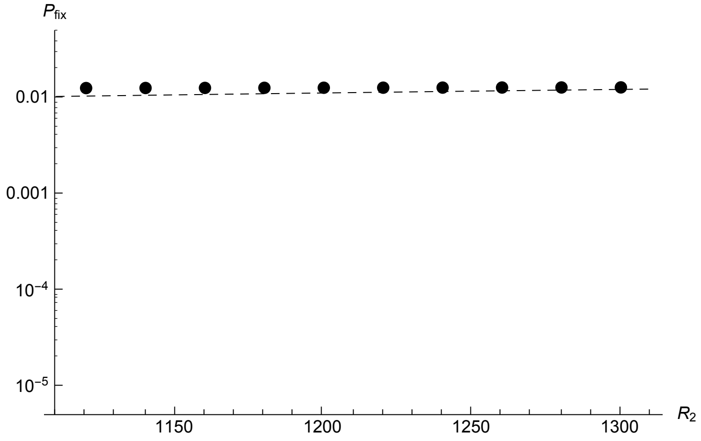

$\mu = 0.0025$

```
data1 = Transpose[Select[dataF, #[[4]] == 5 && #[[8]] == 0.0025 &]];
data1a = Transpose[Select[dataF2, #[[4]] == 5 && #[[8]] == 0.0025 &]];
pts1  = Transpose[{data1[[3]], data1[[10]]}];
pts1a  = Transpose[{data1a[[3]], data1a[[10]]}];
err1 = data1[[11]];
err1a = data1a[[11]];
p2 = Show[LogPlot[
    {PEmerB2[1000, 100, R2, 4, 1, 20, 0.0025]}, {R2, 1110, 1310},
    PlotRange → {Automatic, {0.000005, 0.05}},
    PlotStyle → {Red}, AxesLabel → {"R2", "Pfix"}],
  ErrorListLogPlot[{{pts1[[1]], ErrorBar[err1[[1]]]},
     {pts1[[2]], ErrorBar[err1[[2]]]}, {pts1[[3]], ErrorBar[err1[[3]]]},
     {pts1[[4]], ErrorBar[err1[[4]]]}, {pts1[[5]], ErrorBar[err1[[5]]]},
     {pts1a[[1]], ErrorBar[err1a[[1]]]}, {pts1a[[2]], ErrorBar[err1a[[2]]]},
     {pts1a[[3]], ErrorBar[err1a[[3]]]}, {pts1a[[4]], ErrorBar[err1a[[4]]]},
     {pts1a[[5]], ErrorBar[err1a[[5]]]}}, PlotStyle → {Red, PointSize[0.02]}]
 ]
p2X = Show[Plot[
     {PEmerB2[1000, 100, R2, 4, 1, 20, 0.0025]}, {R2, 1110, 1310},
     PlotRange → Automatic, PlotStyle → {Red}, AxesLabel → {"R2", "Pfix"}],
    ErrorListPlot[{{pts1[[1]], ErrorBar[err1[[1]]]},
      {pts1[[2]], ErrorBar[err1[[2]]]}, {pts1[[3]], ErrorBar[err1[[3]]]},
      {pts1[[4]], ErrorBar[err1[[4]]]}, {pts1[[5]], ErrorBar[err1[[5]]]},
      {pts1a[[1]], ErrorBar[err1a[[1]]]}, {pts1a[[2]], ErrorBar[err1a[[2]]]},
      {pts1a[[3]], ErrorBar[err1a[[3]]]}, {pts1a[[4]], ErrorBar[err1a[[4]]]},
      {pts1a[[5]], ErrorBar[err1a[[5]]]}}, PlotStyle → {Red}], PlotRange → All
  ];
```

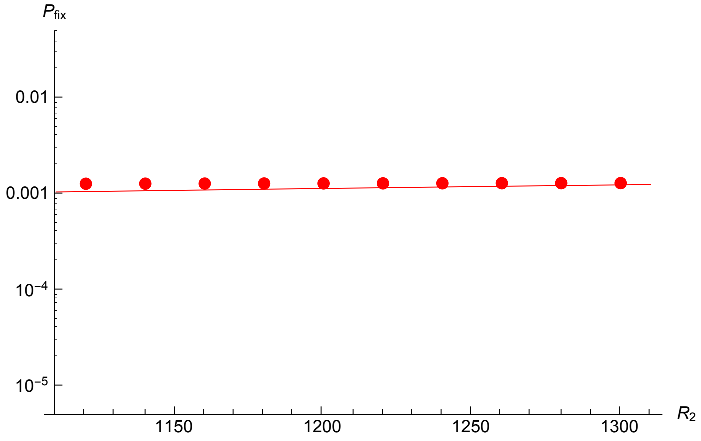

μ = 0.000025

```
data1 = Transpose[Select[dataF, #[[4]] == 5 && #[[8]] == 0.000025 &]];
data1a = Transpose[Select[dataF2, #[[4]] == 5 && #[[8]] == 0.000025 &]];
pts1  = Transpose[{data1[[3]], data1[[10]]}];
pts1a  = Transpose[{data1a[[3]], data1a[[10]]}];
err1 = data1[[11]];
err1a = data1a[[11]];
p3 = Show[LogPlot[
    {PEmerB2[1000, 100, R2, 4, 1, 20, 0.000025]}, {R2, 1110, 1310},
    PlotRange → {Automatic, {0.000005, 0.05}},
    PlotStyle → {Blue}, AxesLabel → {"R2", "Pfix"}],
  ErrorListLogPlot[{{pts1[[1]], ErrorBar[err1[[1]]]},
     {pts1[[2]], ErrorBar[err1[[2]]]}, {pts1[[3]], ErrorBar[err1[[3]]]},
     {pts1[[4]], ErrorBar[err1[[4]]]}, {pts1[[5]], ErrorBar[err1[[5]]]},
     {pts1a[[1]], ErrorBar[err1a[[1]]]}, {pts1a[[2]], ErrorBar[err1a[[2]]]},
     {pts1a[[3]], ErrorBar[err1a[[3]]]}, {pts1a[[4]], ErrorBar[err1a[[4]]]},
     {pts1a[[5]], ErrorBar[err1a[[5]]]}}, PlotStyle → {Blue, PointSize[0.02]}]
 ]
p3X = Show[Plot[
     {PEmerB2[1000, 100, R2, 4, 1, 20, 0.000025]}, {R2, 1110, 1310},
     PlotRange → Automatic, PlotStyle → {Blue}, AxesLabel → {"R2", "Pfix"}],
    ErrorListPlot[{{pts1[[1]], ErrorBar[err1[[1]]]},
      {pts1[[2]], ErrorBar[err1[[2]]]}, {pts1[[3]], ErrorBar[err1[[3]]]},
      {pts1[[4]], ErrorBar[err1[[4]]]}, {pts1[[5]], ErrorBar[err1[[5]]]},
      {pts1a[[1]], ErrorBar[err1a[[1]]]}, {pts1a[[2]], ErrorBar[err1a[[2]]]},
      {pts1a[[3]], ErrorBar[err1a[[3]]]}, {pts1a[[4]], ErrorBar[err1a[[4]]]},
      {pts1a[[5]], ErrorBar[err1a[[5]]]}}, PlotStyle → {Blue}], PlotRange → All
  ];
```

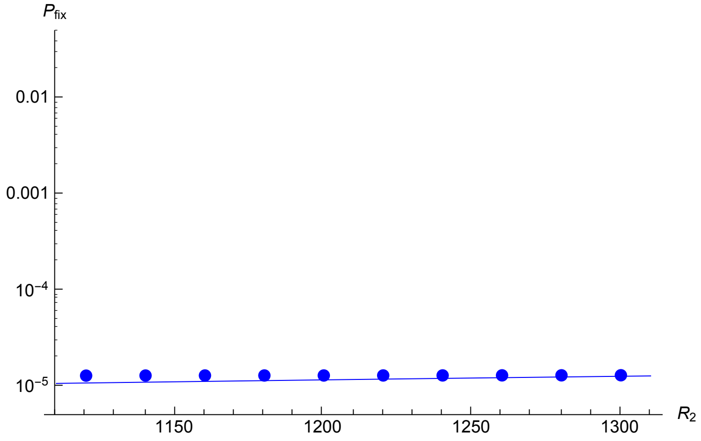

All μ together:

```
Show[p1, p2, p3]
```

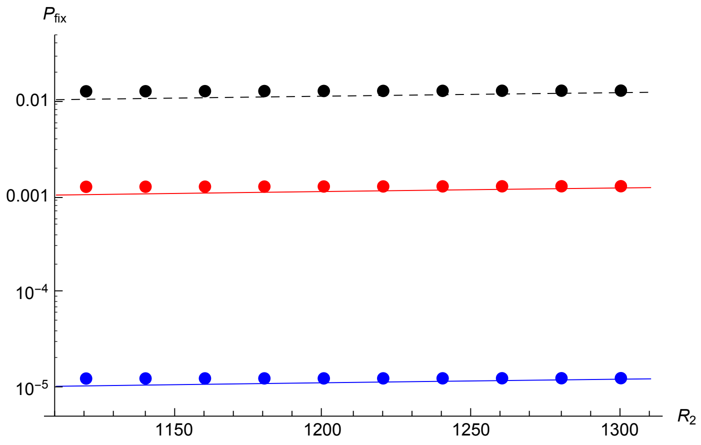

$\rho = 9$

$\mu = 0.025$

```
data1 = Transpose[Select[dataF, #[[4]] == 10 && #[[8]] == 0.025 &]];
data1a = Transpose[Select[dataF2, #[[4]] == 10 && #[[8]] == 0.025 &]];
pts1 = Transpose[{data1[[3]], data1[[10]]}];
pts1a = Transpose[{data1a[[3]], data1a[[10]]}];
err1 = data1[[11]];
err1a = data1a[[11]];
p1 = Show[LogPlot[
    {PEmerB2[1000, 100, R2, 9, 1, 20, 0.025]}, {R2, 1110, 1310},
    PlotRange → {Automatic, {0.000001, 0.05}},
    PlotStyle → {Black, Dashed}, AxesLabel → {"R2", "Pfix"}],
  ErrorListLogPlot[{{pts1[[1]], ErrorBar[err1[[1]]]},
     {pts1[[2]], ErrorBar[err1[[2]]]}, {pts1[[3]], ErrorBar[err1[[3]]]},
     {pts1[[4]], ErrorBar[err1[[4]]]}, {pts1[[5]], ErrorBar[err1[[5]]]},
     {pts1a[[1]], ErrorBar[err1a[[1]]]}, {pts1a[[2]], ErrorBar[err1a[[2]]]},
     {pts1a[[3]], ErrorBar[err1a[[3]]]}, {pts1a[[4]], ErrorBar[err1a[[4]]]},
     {pts1a[[5]], ErrorBar[err1a[[5]]]}}, PlotStyle → {Black, PointSize[0.02]}]
 ]
p1X = Show[Plot[
     {PEmerB2[1000, 100, R2, 9, 1, 20, 0.025]}, {R2, 1110, 1310},
     PlotRange → Automatic, PlotStyle → {Black, Dashed}, AxesLabel → {"R2", "Pfix"}],
   ErrorListPlot[{{pts1[[1]], ErrorBar[err1[[1]]]},
      {pts1[[2]], ErrorBar[err1[[2]]]}, {pts1[[3]], ErrorBar[err1[[3]]]},
      {pts1[[4]], ErrorBar[err1[[4]]]}, {pts1[[5]], ErrorBar[err1[[5]]]},
      {pts1a[[1]], ErrorBar[err1a[[1]]]}, {pts1a[[2]], ErrorBar[err1a[[2]]]},
      {pts1a[[3]], ErrorBar[err1a[[3]]]}, {pts1a[[4]], ErrorBar[err1a[[4]]]},
      {pts1a[[5]], ErrorBar[err1a[[5]]]}}, PlotStyle → {Black}], PlotRange → All
  ];
```

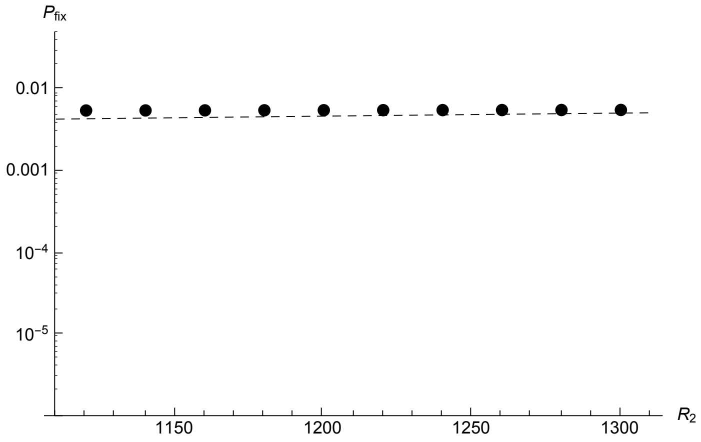

$\mu = 0.0025$

```
data1 = Transpose[Select[dataF, #[[4]] == 10 && #[[8]] == 0.0025 &]];
data1a = Transpose[Select[dataF2, #[[4]] == 10 && #[[8]] == 0.0025 &]];
pts1 = Transpose[{data1[[3]], data1[[10]]}];
pts1a = Transpose[{data1a[[3]], data1a[[10]]}];
err1 = data1[[11]];
err1a = data1a[[11]];
p2 = Show[LogPlot[
    {PEmerB2[1000, 100, R2, 9, 1, 20, 0.0025]}, {R2, 1110, 1310},
    PlotRange → {Automatic, {0.000001, 0.05}},
    PlotStyle → {Red}, AxesLabel → {"R₂", "Pfix"}],
  ErrorListLogPlot[{{pts1[[1]], ErrorBar[err1[[1]]]},
     {pts1[[2]], ErrorBar[err1[[2]]]}, {pts1[[3]], ErrorBar[err1[[3]]]},
     {pts1[[4]], ErrorBar[err1[[4]]]}, {pts1[[5]], ErrorBar[err1[[5]]]},
     {pts1a[[1]], ErrorBar[err1a[[1]]]}, {pts1a[[2]], ErrorBar[err1a[[2]]]},
     {pts1a[[3]], ErrorBar[err1a[[3]]]}, {pts1a[[4]], ErrorBar[err1a[[4]]]},
     {pts1a[[5]], ErrorBar[err1a[[5]]]}}, PlotStyle → {Red, PointSize[0.02]}]
 ]
p2X = Show[Plot[
     {PEmerB2[1000, 100, R2, 9, 1, 20, 0.0025]}, {R2, 1110, 1310},
     PlotRange → Automatic, PlotStyle → {Red}, AxesLabel → {"R₂", "Pfix"}],
   ErrorListPlot[{{pts1[[1]], ErrorBar[err1[[1]]]},
      {pts1[[2]], ErrorBar[err1[[2]]]}, {pts1[[3]], ErrorBar[err1[[3]]]},
      {pts1[[4]], ErrorBar[err1[[4]]]}, {pts1[[5]], ErrorBar[err1[[5]]]},
      {pts1a[[1]], ErrorBar[err1a[[1]]]}, {pts1a[[2]], ErrorBar[err1a[[2]]]},
      {pts1a[[3]], ErrorBar[err1a[[3]]]}, {pts1a[[4]], ErrorBar[err1a[[4]]]},
      {pts1a[[5]], ErrorBar[err1a[[5]]]}}, PlotStyle → {Red}], PlotRange → All
  ];
```

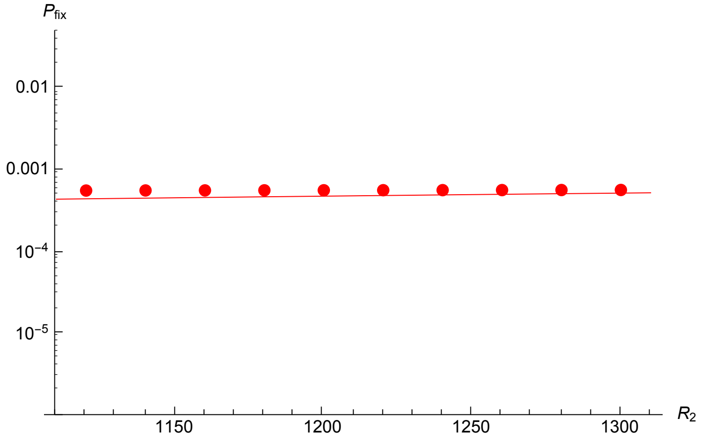

$\mu = 0.000025$

```
data1 = Transpose[Select[dataF, #[[4]] == 10 && #[[8]] == 0.000025 &]];
data1a = Transpose[Select[dataF2, #[[4]] == 10 && #[[8]] == 0.000025 &]];
pts1 = Transpose[{data1[[3]], data1[[10]]}];
pts1a = Transpose[{data1a[[3]], data1a[[10]]}];
err1 = data1[[11]];
err1a = data1a[[11]];
p3 = Show[LogPlot[
   {PEmerB2[1000, 100, R2, 9, 1, 20, 0.000025]}, {R2, 1110, 1310},
   PlotRange → {Automatic, {0.000001, 0.05}},
   PlotStyle → {Blue}, AxesLabel → {"R2", "Pfix"}],
  ErrorListLogPlot[{{pts1[[1]], ErrorBar[err1[[1]]]},
    {pts1[[2]], ErrorBar[err1[[2]]]}, {pts1[[3]], ErrorBar[err1[[3]]]},
    {pts1[[4]], ErrorBar[err1[[4]]]}, {pts1[[5]], ErrorBar[err1[[5]]]},
    {pts1a[[1]], ErrorBar[err1a[[1]]]}, {pts1a[[2]], ErrorBar[err1a[[2]]]},
    {pts1a[[3]], ErrorBar[err1a[[3]]]}, {pts1a[[4]], ErrorBar[err1a[[4]]]},
    {pts1a[[5]], ErrorBar[err1a[[5]]]}}, PlotStyle → {Blue, PointSize[0.02]}]
 ]
p3X = Show[Plot[
    {PEmerB2[1000, 100, R2, 9, 1, 20, 0.000025]}, {R2, 1110, 1310},
    PlotRange → Automatic, PlotStyle → {Blue}, AxesLabel → {"R2", "Pfix"}],
   ErrorListPlot[{{pts1[[1]], ErrorBar[err1[[1]]]},
     {pts1[[2]], ErrorBar[err1[[2]]]}, {pts1[[3]], ErrorBar[err1[[3]]]},
     {pts1[[4]], ErrorBar[err1[[4]]]}, {pts1[[5]], ErrorBar[err1[[5]]]},
     {pts1a[[1]], ErrorBar[err1a[[1]]]}, {pts1a[[2]], ErrorBar[err1a[[2]]]},
     {pts1a[[3]], ErrorBar[err1a[[3]]]}, {pts1a[[4]], ErrorBar[err1a[[4]]]},
     {pts1a[[5]], ErrorBar[err1a[[5]]]}}, PlotStyle → {Blue}], PlotRange → All
  ];
```

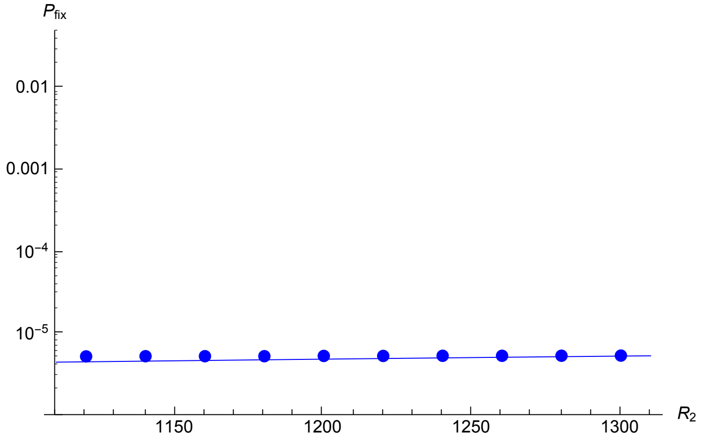

All μ together:

```
Show[p1, p2, p3]
```

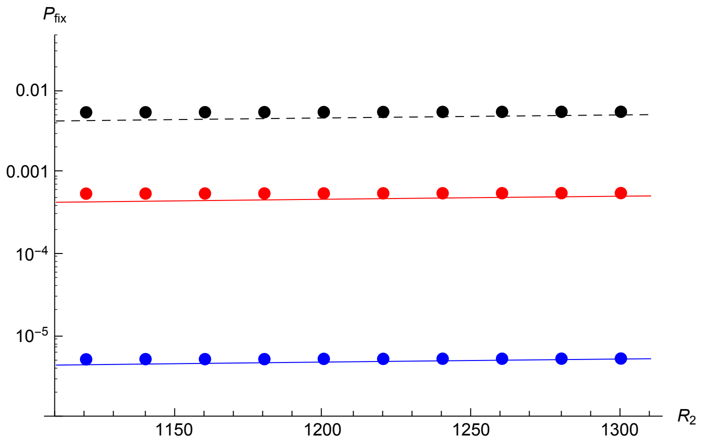

K = 10,000 simulations

```
dataG = Import["results_21STFeb.dat", "Table"];
```

```
YMaxGN[Ka_, R_, R2_, ρ_, x0_, y0_] :=
 Floor[y /. FindRoot[(1 + FX3[Ka, R, ρ, x0, y0, y]) (Ka - y) y ρ + R2 == 0, {y, 3 * Ka / 4}]]
```

```
YMaxN[Ka_, R_, ρ_, x0_, y0_] :=
 y /. FindRoot[x0 + 1/ρ (Log[(y/y0)^R ((Ka - y)/(Ka - y0))^(Ka-R)]) == 0, {y, 3 * Ka / 4}]
```

ρ = 0.5

μ = 0.025

```
data1 = Transpose[Select[dataG, #[[4]] == 1.5 && #[[8]] == 0.025 &]];
pts1 = Transpose[{data1[[3]], data1[[10]]}];
err1 = data1[[11]];
p1 = Show[LogPlot[{PEmerB2[10 000, 2000, R2, 0.5, 1, 20, 0.025]}, {R2, 10 900, 13 100},
   PlotRange → {Automatic, {0.00001, 0.5}},
   PlotStyle → {Black, Dashed}, AxesLabel → {"R2", "Pfix"}], ErrorListLogPlot[
   {{pts1[[1]], ErrorBar[err1[[1]]]}, {pts1[[2]], ErrorBar[err1[[2]]]},
    {pts1[[3]], ErrorBar[err1[[3]]]}, {pts1[[4]], ErrorBar[err1[[4]]]},
    {pts1[[5]], ErrorBar[err1[[5]]]}}, PlotStyle → {Black, PointSize[0.02]}]
 ]
p1X = Show[Plot[
    {PEmerB2[10 000, 2000, R2, 0.5, 1, 20, 0.025]}, {R2, 10 900, 13 100},
    PlotRange → Automatic, PlotStyle → {Black}, AxesLabel → {"R2", "Pfix"}],
   ErrorListPlot[
    {{pts1[[1]], ErrorBar[err1[[1]]]}, {pts1[[2]], ErrorBar[err1[[2]]]},
     {pts1[[3]], ErrorBar[err1[[3]]]}, {pts1[[4]], ErrorBar[err1[[4]]]},
     {pts1[[5]], ErrorBar[err1[[5]]]}}, PlotStyle → {Black}], PlotRange → All
  ];
```

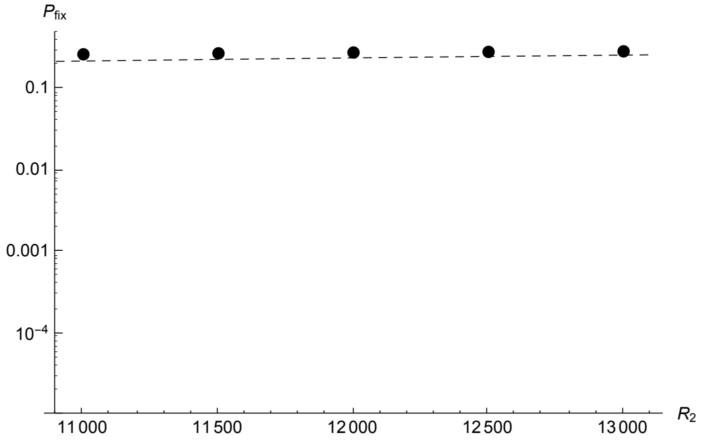

$\mu = 0.0025$

```
data1 = Transpose[Select[dataG, #[[4]] == 1.5 && #[[8]] == 0.0025 &]];
pts1  = Transpose[{data1[[3]], data1[[10]]}];
err1 = data1[[11]];
p2 = Show[LogPlot[PEmerB2[10 000, 2000, R2, 0.5, 1, 20, 0.0025], {R2, 10 900, 13 100},
    PlotRange → {Automatic, {0.00001, 0.5}},
    PlotStyle → {Red}, AxesLabel → {"R2", "Pfix"}],
   ErrorListLogPlot[{{pts1[[1]], ErrorBar[err1[[1]]]},
     {pts1[[2]], ErrorBar[err1[[2]]]}, {pts1[[3]], ErrorBar[err1[[3]]]},
     {pts1[[4]], ErrorBar[err1[[4]]]}, {pts1[[5]], ErrorBar[err1[[5]]]}},
    PlotStyle → {Red, PointSize[0.02]}]
  ]
p2X = Show[Plot[
     {PEmerB2[10 000, 2000, R2, 0.5, 1, 20, 0.0025]}, {R2, 10 900, 13 100},
     PlotRange → Automatic, PlotStyle → {Red}, AxesLabel → {"R2", "Pfix"}],
    ErrorListPlot[
     {{pts1[[1]], ErrorBar[err1[[1]]]}, {pts1[[2]], ErrorBar[err1[[2]]]},
      {pts1[[3]], ErrorBar[err1[[3]]]}, {pts1[[4]], ErrorBar[err1[[4]]]},
      {pts1[[5]], ErrorBar[err1[[5]]]}}, PlotStyle → {Red}], PlotRange → All
   ];
```

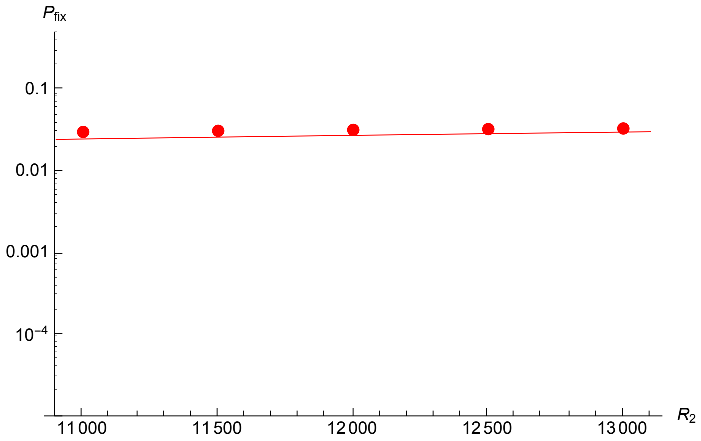

$\mu = 0.001$

```
data1 = Transpose[Select[dataG, #[[4]] == 1.5 && #[[8]] == 0.000025 &]];
pts1 = Transpose[{data1[[3]], data1[[10]]}];
err1 = data1[[11]];
p3 = Show[LogPlot[
    {PEmerB2[10 000, 2000, R2, 0.5, 1, 20, 0.000025]}, {R2, 10 900, 13 100},
    PlotRange → {Automatic, {0.00001, 0.5}},
    PlotStyle → {Blue}, AxesLabel → {"R2", "Pfix"}],
   ErrorListLogPlot[{{pts1[[1]], ErrorBar[err1[[1]]]},
     {pts1[[2]], ErrorBar[err1[[2]]]}, {pts1[[3]], ErrorBar[err1[[3]]]},
     {pts1[[4]], ErrorBar[err1[[4]]]}, {pts1[[5]], ErrorBar[err1[[5]]]}},
    PlotStyle → {Blue, PointSize[0.02]}], PlotRange → All
  ]
p3X = Show[Plot[
     {PEmerB2[10 000, 2000, R2, 0.5, 1, 20, 0.000025]}, {R2, 10 900, 13 100},
     PlotRange → Automatic, PlotStyle → {Blue}, AxesLabel → {"R2", "Pfix"}],
    ErrorListPlot[
     {{pts1[[1]], ErrorBar[err1[[1]]]}, {pts1[[2]], ErrorBar[err1[[2]]]},
      {pts1[[3]], ErrorBar[err1[[3]]]}, {pts1[[4]], ErrorBar[err1[[4]]]},
      {pts1[[5]], ErrorBar[err1[[5]]]}}, PlotStyle → {Blue}], PlotRange → All
   ];
```

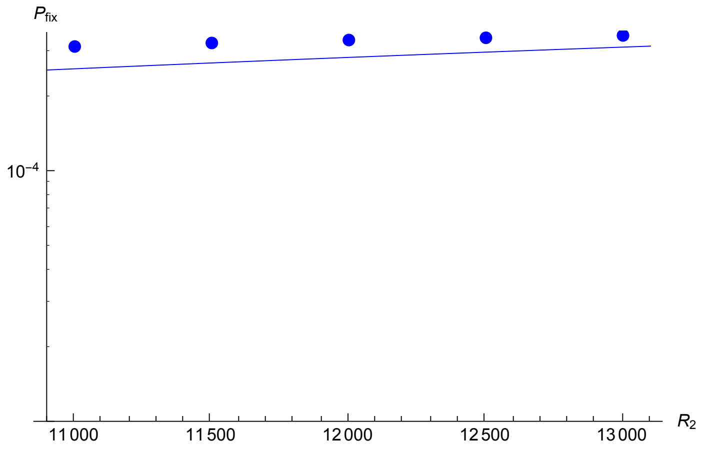

All μ together:

```
Show[p1, p2, p3]
```

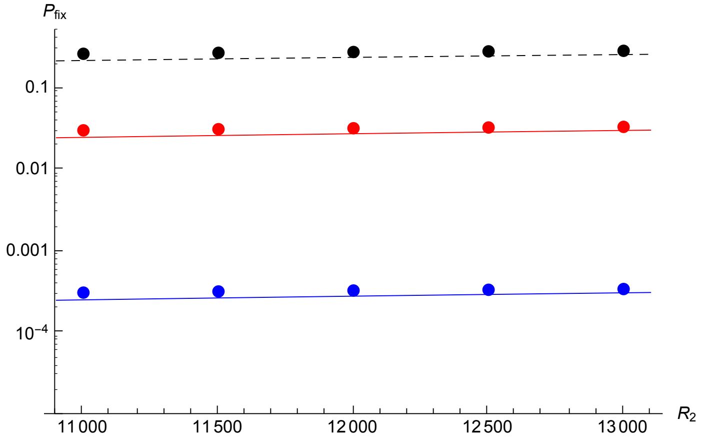

$\rho = 1$

$\mu = 0.025$

```
data1 = Transpose[Select[dataG, #[[4]] == 2 && #[[8]] == 0.025 &]];
pts1 = Transpose[{data1[[3]], data1[[10]]}];
err1 = data1[[11]];
p1 = Show[LogPlot[
    {PEmerB2[10 000, 2000, R2, 1, 1, 20, 0.025]}, {R2, 10 900, 13 100},
    PlotRange → {Automatic, {0.000025, 0.25}},
    PlotStyle → {Black, Dashed}, AxesLabel → {"R₂", "Pfix"}],
   ErrorListLogPlot[{{pts1[[1]], ErrorBar[err1[[1]]]},
     {pts1[[2]], ErrorBar[err1[[2]]]}, {pts1[[3]], ErrorBar[err1[[3]]]},
     {pts1[[4]], ErrorBar[err1[[4]]]}, {pts1[[5]], ErrorBar[err1[[5]]]}},
    PlotStyle → {Black, PointSize[0.02]}]
  ]
p1X = Show[Plot[
     {PEmerB2[10 000, 2000, R2, 1, 1, 20, 0.025]}, {R2, 10 900, 13 100},
     PlotRange → Automatic, PlotStyle → Black, AxesLabel → {"R₂", "Pfix"}],
    ErrorListPlot[
     {{pts1[[1]], ErrorBar[err1[[1]]]}, {pts1[[2]], ErrorBar[err1[[2]]]},
      {pts1[[3]], ErrorBar[err1[[3]]]}, {pts1[[4]], ErrorBar[err1[[4]]]},
      {pts1[[5]], ErrorBar[err1[[5]]]}}, PlotStyle → {Black}], PlotRange → All
   ];
```

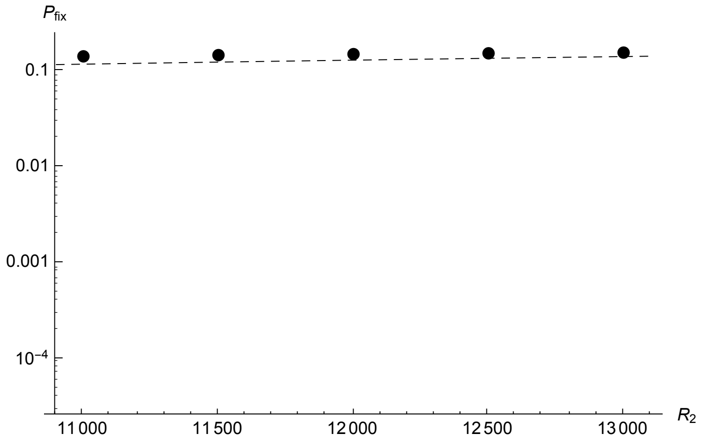

$\mu = 0.0025$

```
data1 = Transpose[Select[dataG, #[[4]] == 2 && #[[8]] == 0.0025 &]];
pts1  = Transpose[{data1[[3]], data1[[10]]}];
err1 = data1[[11]];
p2 = Show[LogPlot[
    {PEmerB2[10 000, 2000, R2, 1, 1, 20, 0.0025]}, {R2, 10 900, 13 100},
    PlotRange → {Automatic, {0.000025, 0.1}},
    PlotStyle → {Red}, AxesLabel → {"R2", "Pfix"}],
   ErrorListLogPlot[{{pts1[[1]], ErrorBar[err1[[1]]]},
     {pts1[[2]], ErrorBar[err1[[2]]]}, {pts1[[3]], ErrorBar[err1[[3]]]},
     {pts1[[4]], ErrorBar[err1[[4]]]}, {pts1[[5]], ErrorBar[err1[[5]]]}},
    PlotStyle → {Red, PointSize[0.02]}]
  ]
p2X = Show[Plot[
     {PEmerB2[10 000, 2000, R2, 1, 1, 20, 0.0025]}, {R2, 10 900, 13 100},
     PlotRange → Automatic, PlotStyle → {Red}, AxesLabel → {"R2", "Pfix"}],
    ErrorListPlot[
     {{pts1[[1]], ErrorBar[err1[[1]]]}, {pts1[[2]], ErrorBar[err1[[2]]]},
      {pts1[[3]], ErrorBar[err1[[3]]]}, {pts1[[4]], ErrorBar[err1[[4]]]},
      {pts1[[5]], ErrorBar[err1[[5]]]}}, PlotStyle → {Red}], PlotRange → All
   ];
```

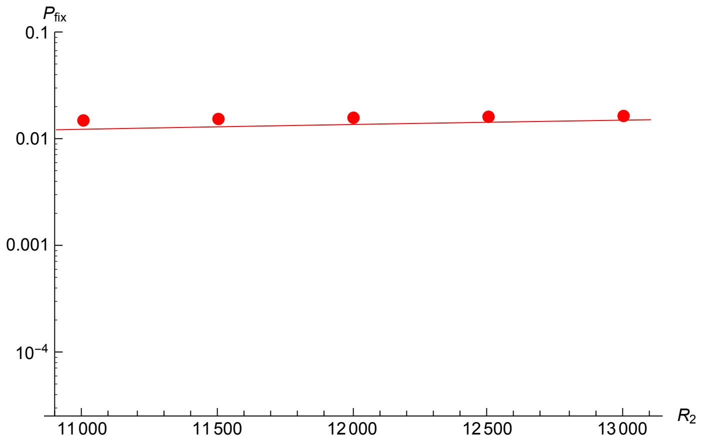

μ = 0.000025

```
data1 = Transpose[Select[dataG, #[[4]] == 2 && #[[8]] == 0.000025 &]];
pts1 = Transpose[{data1[[3]], data1[[10]]}];
err1 = data1[[11]];
p3 = Show[LogPlot[
    {PEmerB2[10 000, 2000, R2, 1, 1, 20, 0.000025]}, {R2, 10 900, 13 100},
    PlotRange → {Automatic, {0.000025, 0.1}},
    PlotStyle → {Blue}, AxesLabel → {"R2", "Pfix"}],
  ErrorListLogPlot[{{pts1[[1]], ErrorBar[err1[[1]]]},
     {pts1[[2]], ErrorBar[err1[[2]]]}, {pts1[[3]], ErrorBar[err1[[3]]]},
     {pts1[[4]], ErrorBar[err1[[4]]]}, {pts1[[5]], ErrorBar[err1[[5]]]}},
    PlotStyle → {Blue, PointSize[0.02]}]
 ]
p3X = Show[Plot[
     {PEmerB2[10 000, 2000, R2, 1, 1, 20, 0.000025]}, {R2, 10 900, 13 100},
     PlotRange → Automatic, PlotStyle → Blue, AxesLabel → {"R2", "Pfix"}],
    ErrorListPlot[
     {{pts1[[1]], ErrorBar[err1[[1]]]}, {pts1[[2]], ErrorBar[err1[[2]]]},
      {pts1[[3]], ErrorBar[err1[[3]]]}, {pts1[[4]], ErrorBar[err1[[4]]]},
      {pts1[[5]], ErrorBar[err1[[5]]]}}, PlotStyle → {Blue}], PlotRange → All
  ];
```

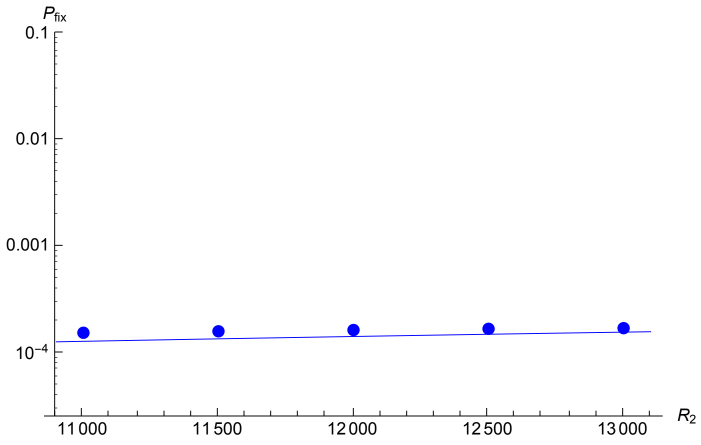

All μ together:

```
Show[p1, p2, p3]
```

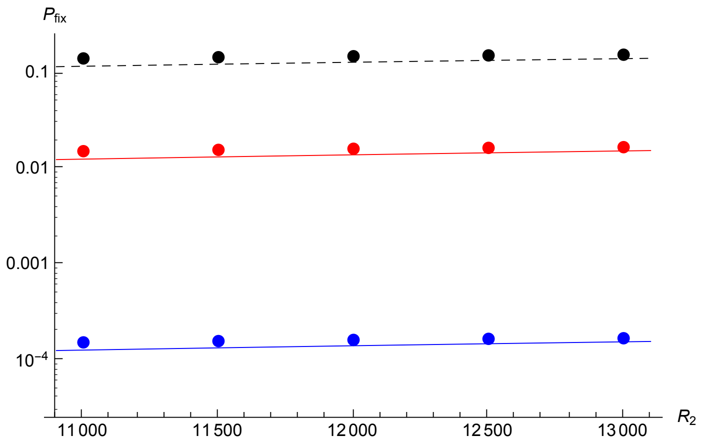

$\rho = 4$

$\mu = 0.025$

```
data1 = Transpose[Select[dataG, #[[4]] == 5 && #[[8]] == 0.025 &]];
pts1 = Transpose[{data1[[3]], data1[[10]]}];
err1 = data1[[11]];
p1 = Show[LogPlot[
   {PEmerB2[10 000, 2000, R2, 4, 1, 20, 0.025]}, {R2, 10 900, 13 100},
   PlotRange → {Automatic, {0.000005, 0.05}},
   PlotStyle → {Black, Dashed}, AxesLabel → {"R2", "Pfix"}],
  ErrorListLogPlot[{{pts1[[1]], ErrorBar[err1[[1]]]},
    {pts1[[2]], ErrorBar[err1[[2]]]}, {pts1[[3]], ErrorBar[err1[[3]]]},
    {pts1[[4]], ErrorBar[err1[[4]]]}, {pts1[[5]], ErrorBar[err1[[5]]]}},
   PlotStyle → {Black, PointSize[0.02]}]
 ]
p1X = Show[Plot[
    {PEmerB2[10 000, 2000, R2, 4, 1, 20, 0.025]}, {R2, 10 900, 13 100},
    PlotRange → Automatic, PlotStyle → {Black, Dashed}, AxesLabel → {"R2", "Pfix"}],
   ErrorListPlot[
    {{pts1[[1]], ErrorBar[err1[[1]]]}, {pts1[[2]], ErrorBar[err1[[2]]]},
     {pts1[[3]], ErrorBar[err1[[3]]]}, {pts1[[4]], ErrorBar[err1[[4]]]},
     {pts1[[5]], ErrorBar[err1[[5]]]}}, PlotStyle → {Black}], PlotRange → All
  ];
```

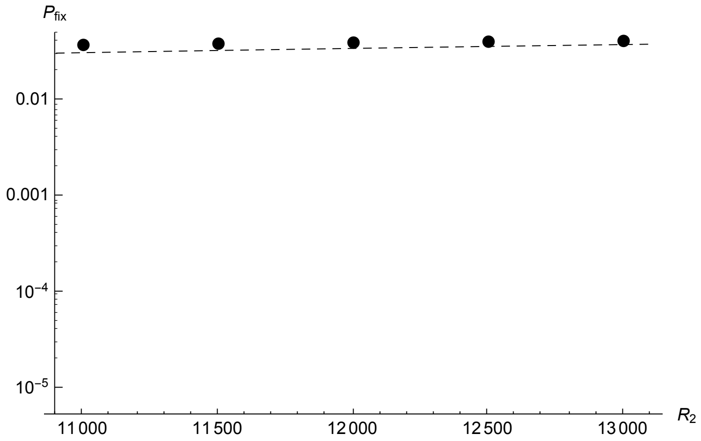

$\mu = 0.0025$

```
data1 = Transpose[Select[dataG, #[[4]] == 5 && #[[8]] == 0.0025 &]];
pts1 = Transpose[{data1[[3]], data1[[10]]}];
err1 = data1[[11]];
p2 = Show[LogPlot[
    {PEmerB2[10 000, 2000, R2, 4, 1, 20, 0.0025]}, {R2, 10 900, 13 100},
    PlotRange → {Automatic, {0.000005, 0.05}},
    PlotStyle → {Red}, AxesLabel → {"R2", "Pfix"}],
   ErrorListLogPlot[{{pts1[[1]], ErrorBar[err1[[1]]]},
     {pts1[[2]], ErrorBar[err1[[2]]]}, {pts1[[3]], ErrorBar[err1[[3]]]},
     {pts1[[4]], ErrorBar[err1[[4]]]}, {pts1[[5]], ErrorBar[err1[[5]]]}},
    PlotStyle → {Red, PointSize[0.02]}]
  ]
p2X = Show[Plot[
     {PEmerB2[10 000, 2000, R2, 4, 1, 20, 0.0025]}, {R2, 10 900, 13 100},
     PlotRange → Automatic, PlotStyle → {Red}, AxesLabel → {"R2", "Pfix"}],
    ErrorListPlot[
     {{pts1[[1]], ErrorBar[err1[[1]]]}, {pts1[[2]], ErrorBar[err1[[2]]]},
      {pts1[[3]], ErrorBar[err1[[3]]]}, {pts1[[4]], ErrorBar[err1[[4]]]},
      {pts1[[5]], ErrorBar[err1[[5]]]}}, PlotStyle → {Red}], PlotRange → All
   ];
```

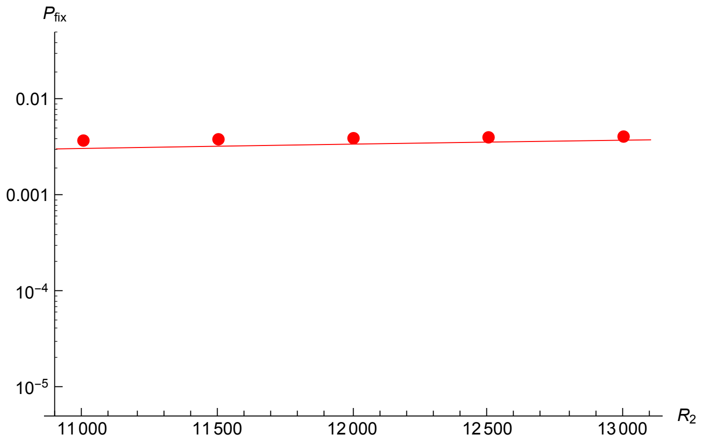

$\mu = 0.000025$

```
data1 = Transpose[Select[dataG, #[[4]] == 5 && #[[8]] == 0.000025 &]];
pts1 = Transpose[{data1[[3]], data1[[10]]}];
err1 = data1[[11]];
p3 = Show[LogPlot[
    {PEmerB2[10 000, 2000, R2, 4, 1, 20, 0.000025]}, {R2, 10 900, 13 100},
    PlotRange → {Automatic, {0.000005, 0.05}},
    PlotStyle → {Blue}, AxesLabel → {"R₂", "Pfix"}],
  ErrorListLogPlot[{{pts1[[1]], ErrorBar[err1[[1]]]},
     {pts1[[2]], ErrorBar[err1[[2]]]}, {pts1[[3]], ErrorBar[err1[[3]]]},
     {pts1[[4]], ErrorBar[err1[[4]]]}, {pts1[[5]], ErrorBar[err1[[5]]]}},
    PlotStyle → {Blue, PointSize[0.02]}]
 ]
p3X = Show[Plot[
     {PEmerB2[10 000, 2000, R2, 4, 1, 20, 0.000025]}, {R2, 10 900, 13 100},
     PlotRange → Automatic, PlotStyle → {Blue}, AxesLabel → {"R₂", "Pfix"}],
    ErrorListPlot[
     {{pts1[[1]], ErrorBar[err1[[1]]]}, {pts1[[2]], ErrorBar[err1[[2]]]},
      {pts1[[3]], ErrorBar[err1[[3]]]}, {pts1[[4]], ErrorBar[err1[[4]]]},
      {pts1[[5]], ErrorBar[err1[[5]]]}}, PlotStyle → {Blue}], PlotRange → All
  ];
```

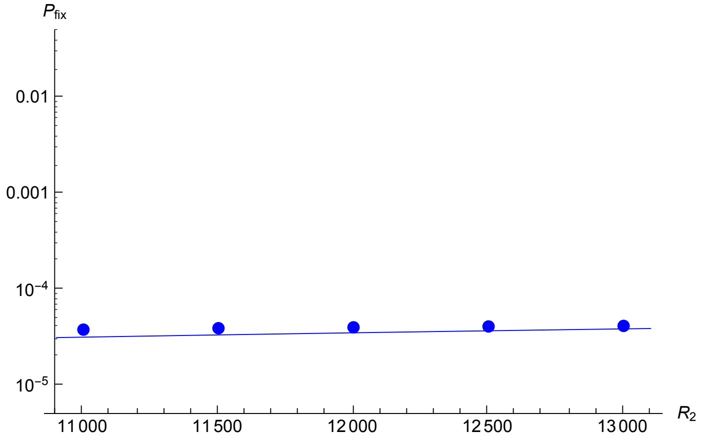

All μ together:

```
Show[p1, p2, p3]
```

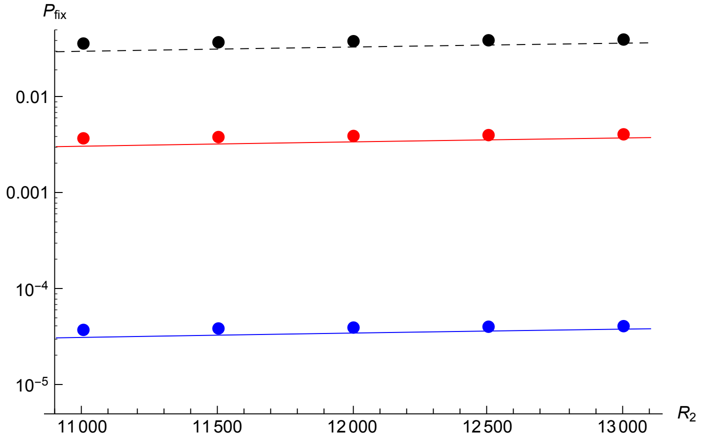

$\rho = 9$

$\mu = 0.025$

```
data1 = Transpose[Select[dataG, #[[4]] == 10 && #[[8]] == 0.025 &]];
pts1  = Transpose[{data1[[3]], data1[[10]]}];
err1 = data1[[11]];
p1 = Show[LogPlot[
    {PEmerB2[10 000, 2000, R2, 9, 1, 20, 0.025]}, {R2, 10 900, 13 100},
    PlotRange → {Automatic, {0.000001, 0.05}},
    PlotStyle → {Black, Dashed}, AxesLabel → {"R2", "Pfix"}],
   ErrorListLogPlot[{{pts1[[1]], ErrorBar[err1[[1]]]},
     {pts1[[2]], ErrorBar[err1[[2]]]}, {pts1[[3]], ErrorBar[err1[[3]]]},
     {pts1[[4]], ErrorBar[err1[[4]]]}, {pts1[[5]], ErrorBar[err1[[5]]]}},
    PlotStyle → {Black, PointSize[0.02]}]
  ]
p1X = Show[Plot[
     {PEmerB2[10 000, 2000, R2, 9, 1, 20, 0.025]}, {R2, 10 900, 13 100},
     PlotRange → Automatic, PlotStyle → {Black, Dashed}, AxesLabel → {"R2", "Pfix"}],
    ErrorListPlot[
     {{pts1[[1]], ErrorBar[err1[[1]]]}, {pts1[[2]], ErrorBar[err1[[2]]]},
      {pts1[[3]], ErrorBar[err1[[3]]]}, {pts1[[4]], ErrorBar[err1[[4]]]},
      {pts1[[5]], ErrorBar[err1[[5]]]}}, PlotStyle → {Black}], PlotRange → All
   ];
```

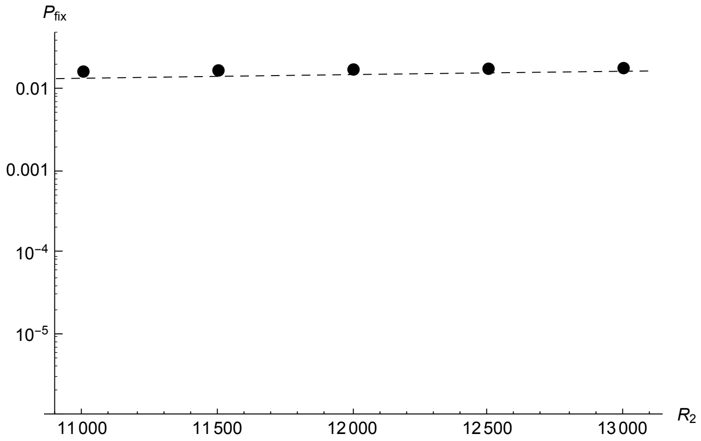

$\mu = 0.0025$

```
data1 = Transpose[Select[dataG, #[[4]] == 10 && #[[8]] == 0.0025 &]];
pts1 = Transpose[{data1[[3]], data1[[10]]}];
err1 = data1[[11]];
p2 = Show[LogPlot[
    {PEmerB2[10 000, 2000, R2, 9, 1, 20, 0.0025]}, {R2, 10 900, 13 100},
    PlotRange → {Automatic, {0.000001, 0.05}},
    PlotStyle → {Red}, AxesLabel → {"R2", "Pfix"}],
   ErrorListLogPlot[{{pts1[[1]], ErrorBar[err1[[1]]]},
     {pts1[[2]], ErrorBar[err1[[2]]]}, {pts1[[3]], ErrorBar[err1[[3]]]},
     {pts1[[4]], ErrorBar[err1[[4]]]}, {pts1[[5]], ErrorBar[err1[[5]]]}},
    PlotStyle → {Red, PointSize[0.02]}]
  ]
p2X = Show[Plot[
     {PEmerB2[10 000, 2000, R2, 9, 1, 20, 0.0025]}, {R2, 10 900, 13 100},
     PlotRange → Automatic, PlotStyle → {Red}, AxesLabel → {"R2", "Pfix"}],
    ErrorListPlot[
     {{pts1[[1]], ErrorBar[err1[[1]]]}, {pts1[[2]], ErrorBar[err1[[2]]]},
      {pts1[[3]], ErrorBar[err1[[3]]]}, {pts1[[4]], ErrorBar[err1[[4]]]},
      {pts1[[5]], ErrorBar[err1[[5]]]}}, PlotStyle → {Red}], PlotRange → All
   ];
```

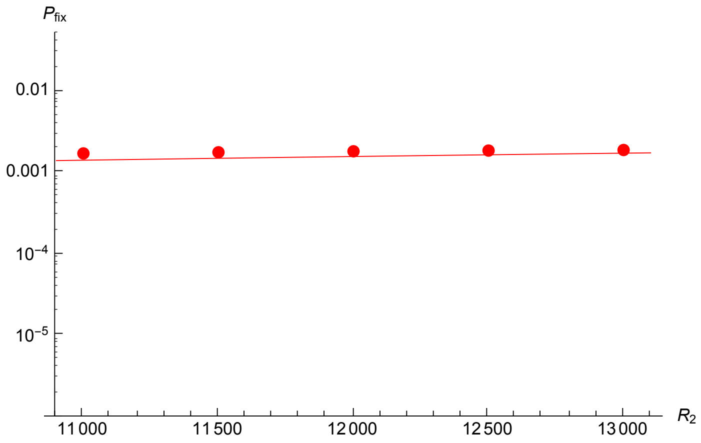

$\mu = 0.000025$

```
data1 = Transpose[Select[dataG, #[[4]] == 10 && #[[8]] == 0.000025 &]];
pts1 = Transpose[{data1[[3]], data1[[10]]}];
err1 = data1[[11]];
p3 = Show[LogPlot[
    {PEmerB2[10 000, 2000, R2, 9, 1, 20, 0.000025]}, {R2, 10 900, 13 100},
    PlotRange → {Automatic, {0.000001, 0.05}},
    PlotStyle → {Blue}, AxesLabel → {"R₂", "Pfix"}],
  ErrorListLogPlot[{{pts1[[1]], ErrorBar[err1[[1]]]},
     {pts1[[2]], ErrorBar[err1[[2]]]}, {pts1[[3]], ErrorBar[err1[[3]]]},
     {pts1[[4]], ErrorBar[err1[[4]]]}, {pts1[[5]], ErrorBar[err1[[5]]]}},
    PlotStyle → {Blue, PointSize[0.02]}]
 ]
p3X = Show[Plot[
     {PEmerB2[10 000, 2000, R2, 9, 1, 20, 0.000025]}, {R2, 10 900, 13 100},
     PlotRange → Automatic, PlotStyle → {Blue}, AxesLabel → {"R₂", "Pfix"}],
    ErrorListPlot[
     {{pts1[[1]], ErrorBar[err1[[1]]]}, {pts1[[2]], ErrorBar[err1[[2]]]},
      {pts1[[3]], ErrorBar[err1[[3]]]}, {pts1[[4]], ErrorBar[err1[[4]]]},
      {pts1[[5]], ErrorBar[err1[[5]]]}}, PlotStyle → {Blue}], PlotRange → All
  ];
```

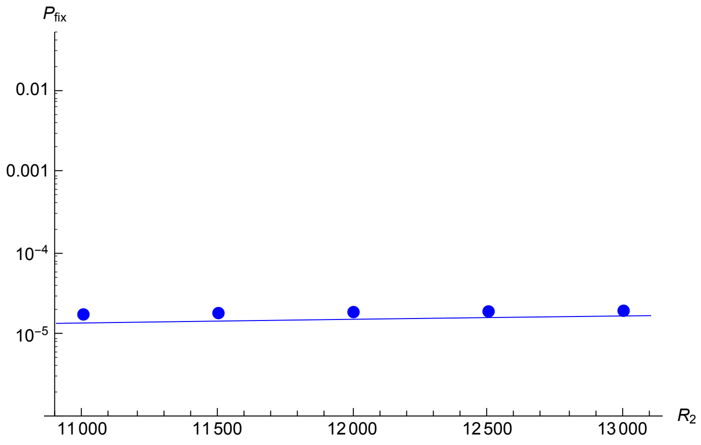

All μ together:

```
Show[p1, p2, p3]
```

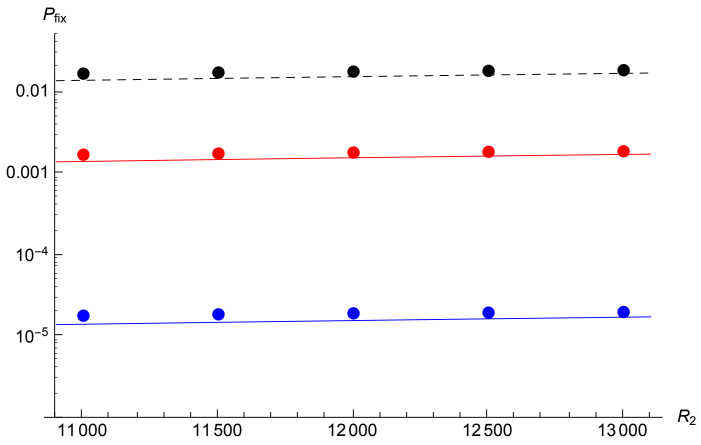

K = 100 simulations, R2 < K

Below commands load simulation output files used in analysis.

```
dataH = Import["results_17Dec2014.dat", "Table"];
```

Redefining “YMaxN” to find correct root:

```
YMaxN[Ka_, R_, ρ_, x0_, y0_] :=
  y /. FindRoot[x0 + 1/ρ (Log[(y/y0)^R ((Ka - y)/(Ka - y0))^(Ka-R)]) == 0, {y, R}]
   (* Function to find at what 'time' (immune size) when x becomes extinct *)
```

ρ = 0.5

μ = 0.0001

```
data1 = Transpose[Select[dataH, #[[4]] == 1.5 && #[[8]] == 0.0001 &]];
pts1  = Transpose[{data1[[3]], data1[[10]]}];
err1 = data1[[11]];
p1 = Show[LogPlot[
    {PEmerB2[100, 60, R2, 0.5, 1, 20, 0.0001]}, {R2, 75, 105},
    PlotRange → {Automatic, {0.00005, 0.05}},
    PlotStyle → {Black, Dashed}, AxesLabel → {"R₂", "Pfix"}],
   ErrorListLogPlot[{{pts1[[1]], ErrorBar[err1[[1]]]},
     {pts1[[2]], ErrorBar[err1[[2]]]}, {pts1[[3]], ErrorBar[err1[[3]]]},
     {pts1[[4]], ErrorBar[err1[[4]]]}, {pts1[[5]], ErrorBar[err1[[5]]]}},
    PlotStyle → {Black, PointSize[0.02]}], PlotRange → All
  ]
```

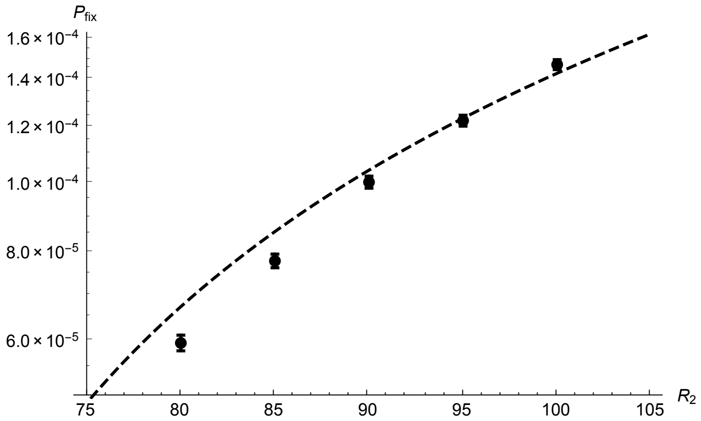

μ = 0.0025

```
data1 = Transpose[Select[dataH, #[[4]] == 1.5 && #[[8]] == 0.0025 &]];
pts1 = Transpose[{data1[[3]], data1[[10]]}];
err1 = data1[[11]];
p2 = Show[LogPlot[
   {PEmerB2[100, 60, R2, 0.5, 1, 20, 0.0025]}, {R2, 75, 105},
   PlotRange → {Automatic, {0.00005, 0.05}},
   PlotStyle → {Red}, AxesLabel → {"R2", "Pfix"}],
  ErrorListLogPlot[{{pts1[[1]], ErrorBar[err1[[1]]]},
    {pts1[[2]], ErrorBar[err1[[2]]]}, {pts1[[3]], ErrorBar[err1[[3]]]},
    {pts1[[4]], ErrorBar[err1[[4]]]}, {pts1[[5]], ErrorBar[err1[[5]]]}},
   PlotStyle → {Red, PointSize[0.02]}], PlotRange → All
 ]
```

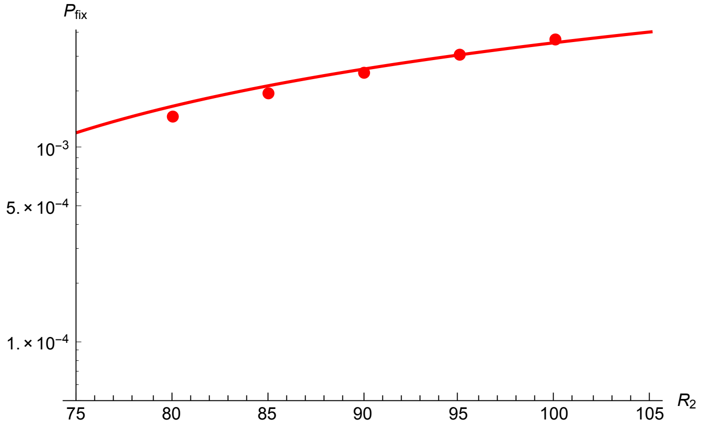

$\mu = 0.025$

```
data1 = Transpose[Select[dataH, #[[4]] == 1.5 && #[[8]] == 0.025 &]];
pts1 = Transpose[{data1[[3]], data1[[10]]}];
err1 = data1[[11]];
p3 = Show[LogPlot[
   {PEmerB2[100, 60, R2, 0.5, 1, 20, 0.025]}, {R2, 75, 105},
   PlotRange → {Automatic, {0.00005, 0.05}},
   PlotStyle → {Blue}, AxesLabel → {"R2", "Pfix"}],
  ErrorListLogPlot[{{pts1[[1]], ErrorBar[err1[[1]]]},
    {pts1[[2]], ErrorBar[err1[[2]]]}, {pts1[[3]], ErrorBar[err1[[3]]]},
    {pts1[[4]], ErrorBar[err1[[4]]]}, {pts1[[5]], ErrorBar[err1[[5]]]}},
   PlotStyle → {Blue, PointSize[0.02]}], PlotRange → All
 ]
```

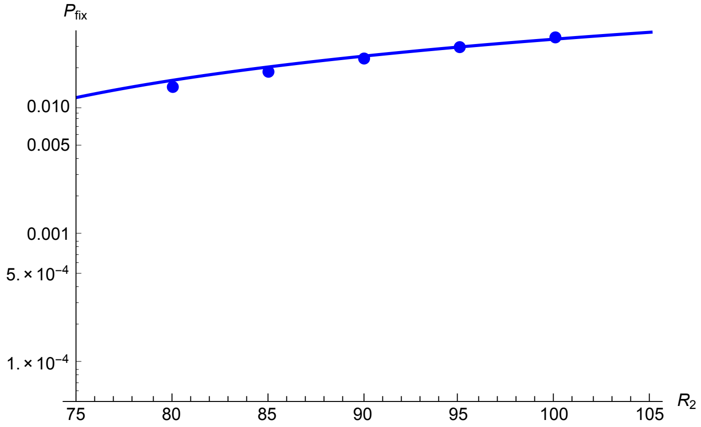

All μ together:

```
Show[p1, p2, p3, PlotRange → All]
```

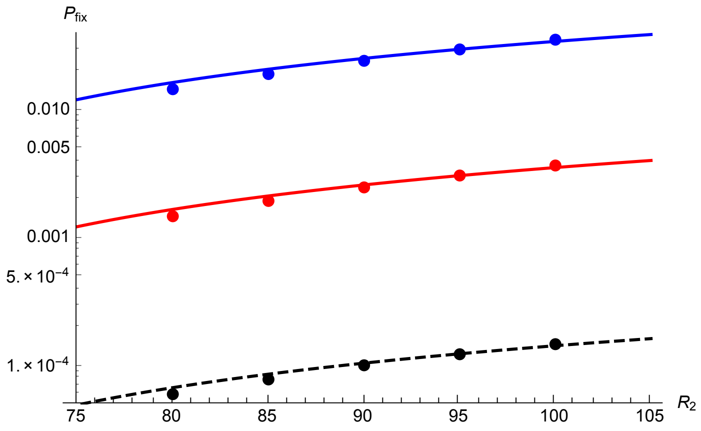

$\rho = 9$

$\mu = 0.0001$

```
data1 = Transpose[Select[dataH, #[[4]] == 10 && #[[8]] == 0.0001 &]];
pts1  = Transpose[{data1[[3]], data1[[10]]}];
err1 = data1[[11]];
p1 = Show[LogPlot[
    {PEmerB2[100, 60, R2, 9, 1, 20, 0.0001]}, {R2, 75, 105},
    PlotRange → {Automatic, {0.0000001, 0.01}},
    PlotStyle → {Black, Dashed}, AxesLabel → {"R2", "Pfix"}],
   ErrorListLogPlot[{{pts1[[1]], ErrorBar[err1[[1]]]},
     {pts1[[2]], ErrorBar[err1[[2]]]}, {pts1[[3]], ErrorBar[err1[[3]]]},
     {pts1[[4]], ErrorBar[err1[[4]]]}, {pts1[[5]], ErrorBar[err1[[5]]]}},
    PlotStyle → {Black, PointSize[0.02]}], PlotRange → All
  ]
```

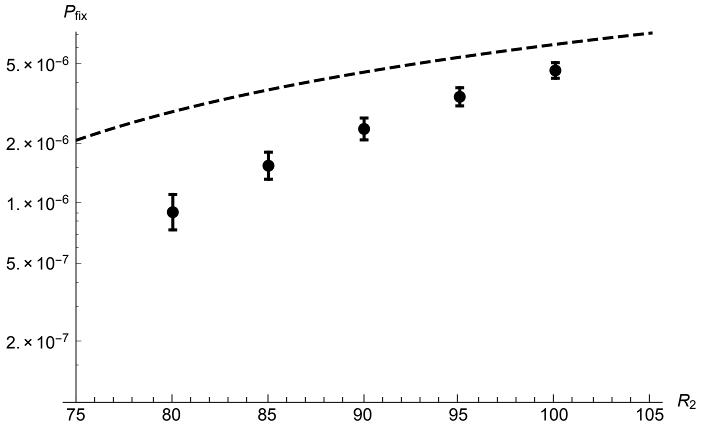

$\mu = 0.0025$

```
data1 = Transpose[Select[dataH, #[[4]] == 10 && #[[8]] == 0.0025 &]];
pts1  = Transpose[{data1[[3]], data1[[10]]}];
err1 = data1[[11]];
p2 = Show[LogPlot[
    {PEmerB2[100, 60, R2, 9, 1, 20, 0.0025]}, {R2, 75, 105},
    PlotRange → {Automatic, {0.0000001, 0.01}},
    PlotStyle → {Red}, AxesLabel → {"R₂", "Pfix"}],
  ErrorListLogPlot[{{pts1[[1]], ErrorBar[err1[[1]]]},
    {pts1[[2]], ErrorBar[err1[[2]]]}, {pts1[[3]], ErrorBar[err1[[3]]]},
    {pts1[[4]], ErrorBar[err1[[4]]]}, {pts1[[5]], ErrorBar[err1[[5]]]}},
    PlotStyle → {Red, PointSize[0.02]}], PlotRange → All
 ]
```

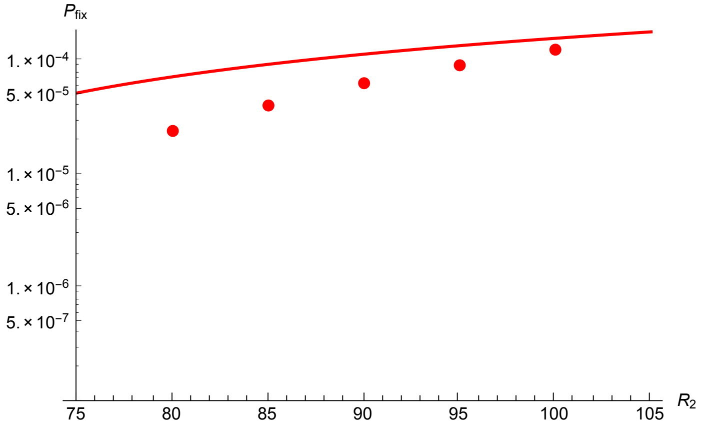

$\mu = 0.025$

```
data1 = Transpose[Select[dataH, #[[4]] == 10 && #[[8]] == 0.025 &]];
pts1  = Transpose[{data1[[3]], data1[[10]]}];
err1 = data1[[11]];
p3 = Show[LogPlot[
    {PEmerB2[100, 60, R2, 9, 1, 20, 0.025]}, {R2, 75, 105},
    PlotRange → {Automatic, {0.0000001, 0.01}},
    PlotStyle → {Blue}, AxesLabel → {"R₂", "Pfix"}],
  ErrorListLogPlot[{{pts1[[1]], ErrorBar[err1[[1]]]},
    {pts1[[2]], ErrorBar[err1[[2]]]}, {pts1[[3]], ErrorBar[err1[[3]]]},
    {pts1[[4]], ErrorBar[err1[[4]]]}, {pts1[[5]], ErrorBar[err1[[5]]]}},
    PlotStyle → {Blue, PointSize[0.02]}], PlotRange → All
 ]
```

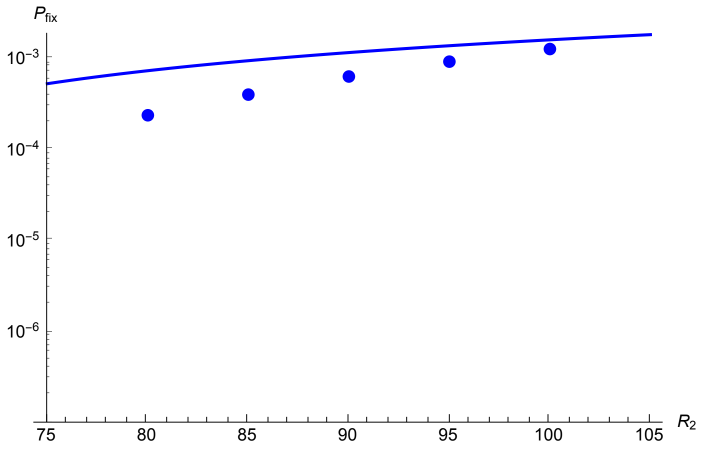

All μ together:

```
Show[p1, p2, p3]
```

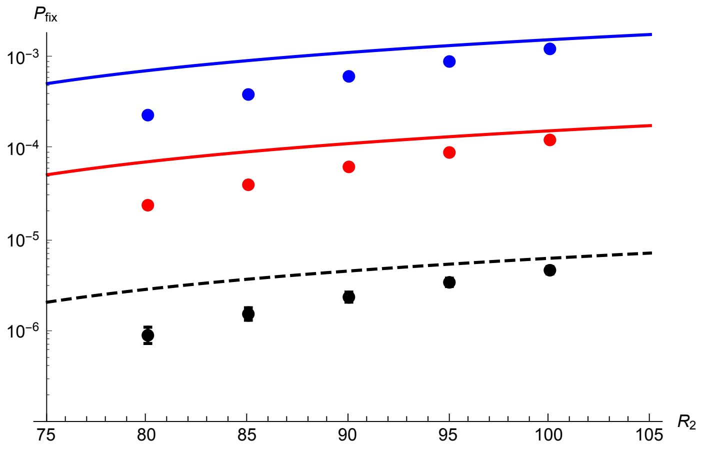

K = 1,000 simulations, R2 < K

Below commands load simulation output files used in analysis.

```
dataJ = Import["results_18Dec2014.dat", "Table"];
```

Redefining “YMaxGN” to find correct root:

```
YMaxGN[Ka_, R_, R2_, ρ_, x0_, y0_] :=
 Floor[y /. FindRoot[(1 + FX3[Ka, R, ρ, x0, y0, y]) (Ka - y) y ρ + R2 - y0 == 0, {y, Ka/4}]]
 (* Function to find when Π becomes inadmissable *)
```

ρ = 0.5

μ = 0.0001

```
data1 = Transpose[Select[dataJ, #[[4]] == 1.5 && #[[8]] == 0.0001 &]];
pts1 = Transpose[{data1[[3]], data1[[10]]}];
err1 = data1[[11]];
p1 = Show[LogPlot[
   {PEmerB2[1000, 100, R2, 0.5, 1, 20, 0.0001]}, {R2, 175, 925},
   PlotRange → {Automatic},
   PlotStyle → {Black, Dashed}, AxesLabel → {"R2", "Pfix"}],
  ErrorListLogPlot[{{pts1[[1]], ErrorBar[err1[[1]]]},
    {pts1[[2]], ErrorBar[err1[[2]]]}, {pts1[[3]], ErrorBar[err1[[3]]]},
    {pts1[[4]], ErrorBar[err1[[4]]]}, {pts1[[5]], ErrorBar[err1[[5]]]}},
   PlotStyle → {Black, PointSize[0.02]}], PlotRange → All
 ]
```

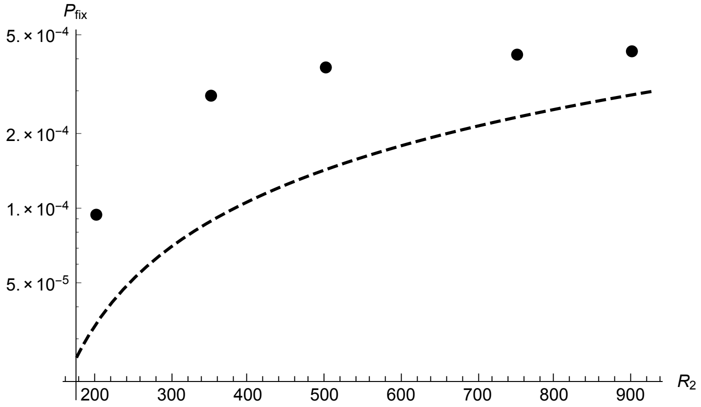

$\mu = 0.0025$

```
data1 = Transpose[Select[dataJ, #[[4]] == 1.5 && #[[8]] == 0.0025 &]];
pts1 = Transpose[{data1[[3]], data1[[10]]}];
err1 = data1[[11]];
p2 = Show[LogPlot[
   {PEmerB2[1000, 100, R2, 0.5, 1, 20, 0.0025]}, {R2, 175, 925},
   PlotRange → {Automatic}, PlotStyle → {Red}, AxesLabel → {"R2", "Pfix"}],
  ErrorListLogPlot[{{pts1[[1]], ErrorBar[err1[[1]]]},
    {pts1[[2]], ErrorBar[err1[[2]]]}, {pts1[[3]], ErrorBar[err1[[3]]]},
    {pts1[[4]], ErrorBar[err1[[4]]]}, {pts1[[5]], ErrorBar[err1[[5]]]}},
   PlotStyle → {Red, PointSize[0.02]}], PlotRange → All
 ]
```

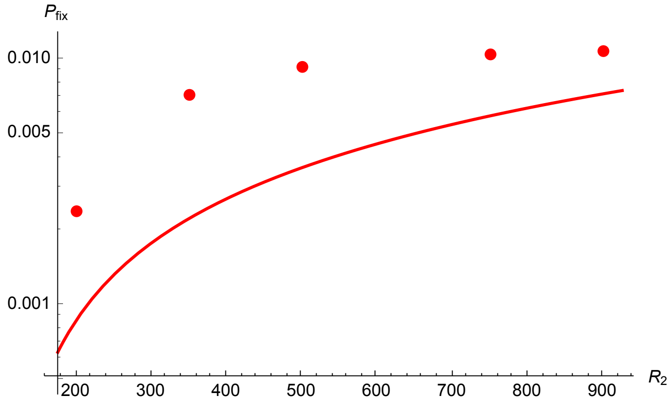

$\mu = 0.025$

```
data1 = Transpose[Select[dataJ, #[[4]] == 1.5 && #[[8]] == 0.025 &]];
pts1 = Transpose[{data1[[3]], data1[[10]]}];
err1 = data1[[11]];
p3 = Show[LogPlot[
   {PEmerB2[1000, 100, R2, 0.5, 1, 20, 0.025]}, {R2, 175, 925},
   PlotRange → {Automatic}, PlotStyle → {Blue}, AxesLabel → {"R₂", "Pfix"}],
  ErrorListLogPlot[{{pts1[[1]], ErrorBar[err1[[1]]]},
    {pts1[[2]], ErrorBar[err1[[2]]]}, {pts1[[3]], ErrorBar[err1[[3]]]},
    {pts1[[4]], ErrorBar[err1[[4]]]}, {pts1[[5]], ErrorBar[err1[[5]]]}},
   PlotStyle → {Blue, PointSize[0.02]}], PlotRange → All
 ]
```

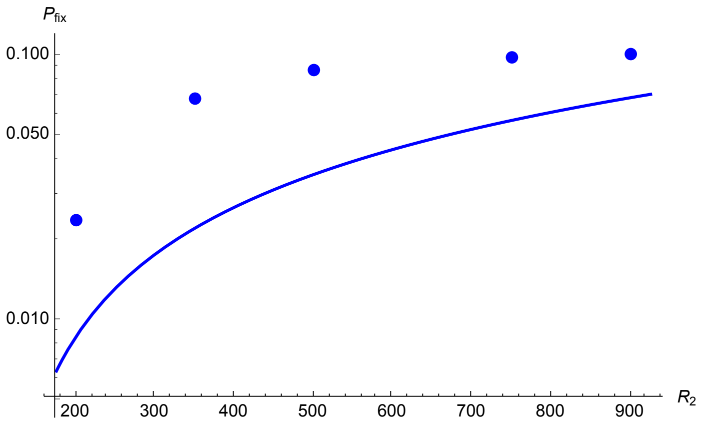

All μ together:

```
Show[p1, p2, p3, PlotRange → All]
```

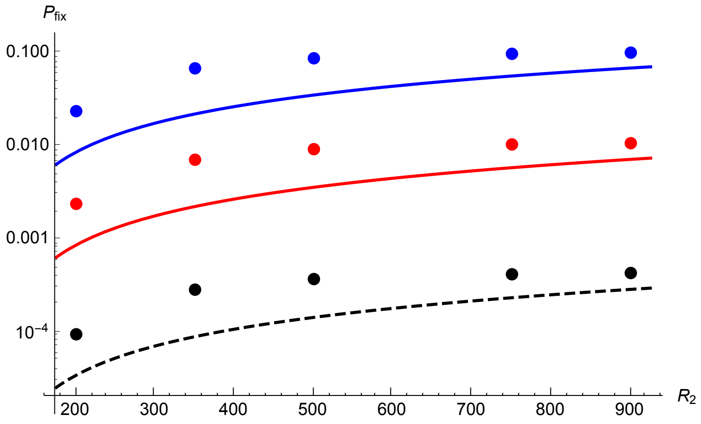

$\rho = 9$

$\mu = 0.0001$

```
data1 = Transpose[Select[dataJ, #[[4]] == 10 && #[[8]] == 0.0001 &]];
pts1  = Transpose[{data1[[3]], data1[[10]]}];
err1 = data1[[11]];
p1 = Show[LogPlot[
    {PEmerB2[1000, 100, R2, 9, 1, 20, 0.0001]}, {R2, 175, 925},
    PlotRange → {Automatic},
    PlotStyle → {Black, Dashed}, AxesLabel → {"R₂", "Pfix"}],
   ErrorListLogPlot[{{pts1[[1]], ErrorBar[err1[[1]]]},
     {pts1[[2]], ErrorBar[err1[[2]]]}, {pts1[[3]], ErrorBar[err1[[3]]]},
     {pts1[[4]], ErrorBar[err1[[4]]]}, {pts1[[5]], ErrorBar[err1[[5]]]}},
    PlotStyle → {Black, PointSize[0.02]}], PlotRange → All
  ]
```

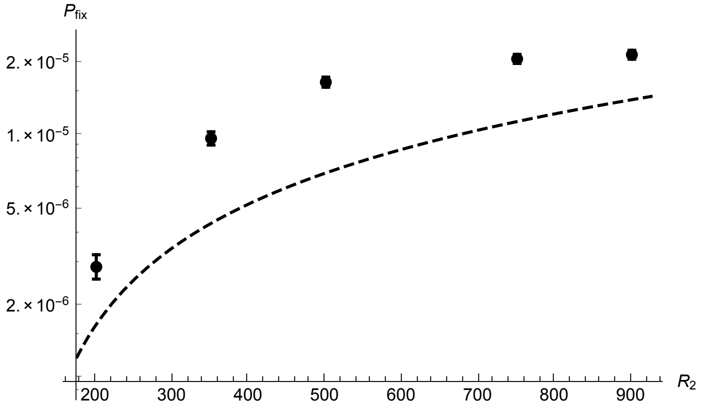

$\mu = 0.0025$

```
data1 = Transpose[Select[dataJ, #[[4]] == 10 && #[[8]] == 0.0025 &]];
pts1  = Transpose[{data1[[3]], data1[[10]]}];
err1 = data1[[11]];
p2 = Show[LogPlot[
    {PEmerB2[1000, 100, R2, 9, 1, 20, 0.0025]}, {R2, 175, 925},
    PlotRange → {Automatic}, PlotStyle → {Red}, AxesLabel → {"R₂", "Pfix"}],
   ErrorListLogPlot[{{pts1[[1]], ErrorBar[err1[[1]]]},
     {pts1[[2]], ErrorBar[err1[[2]]]}, {pts1[[3]], ErrorBar[err1[[3]]]},
     {pts1[[4]], ErrorBar[err1[[4]]]}, {pts1[[5]], ErrorBar[err1[[5]]]}},
    PlotStyle → {Red, PointSize[0.02]}], PlotRange → All
  ]
```

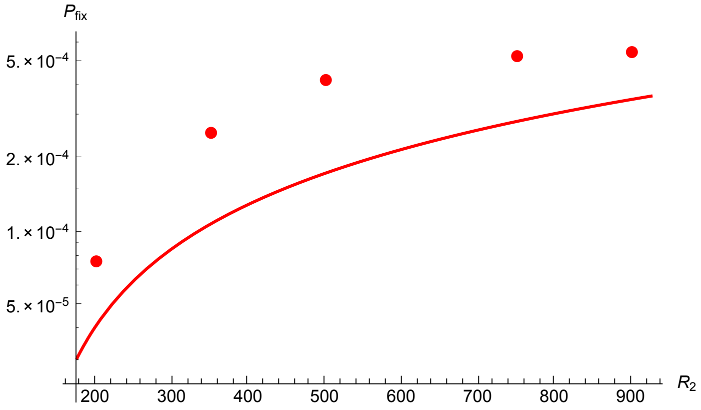

$\mu = 0.025$

```
data1 = Transpose[Select[dataJ, #[[4]] == 10 && #[[8]] == 0.025 &]];
pts1 = Transpose[{data1[[3]], data1[[10]]}];
err1 = data1[[11]];
p3 = Show[LogPlot[
    {PEmerB2[1000, 100, R2, 9, 1, 20, 0.025]}, {R2, 175, 925},
    PlotRange → {Automatic}, PlotStyle → {Blue}, AxesLabel → {"R₂", "Pfix"}],
   ErrorListLogPlot[{{pts1[[1]], ErrorBar[err1[[1]]]},
     {pts1[[2]], ErrorBar[err1[[2]]]}, {pts1[[3]], ErrorBar[err1[[3]]]},
     {pts1[[4]], ErrorBar[err1[[4]]]}, {pts1[[5]], ErrorBar[err1[[5]]]}},
    PlotStyle → {Blue, PointSize[0.02]}, PlotRange → All
 ]
```

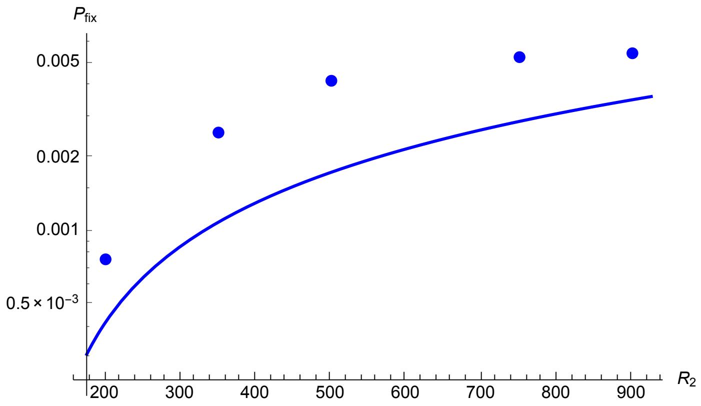

All μ together:

```
Show[p1, p2, p3]
```

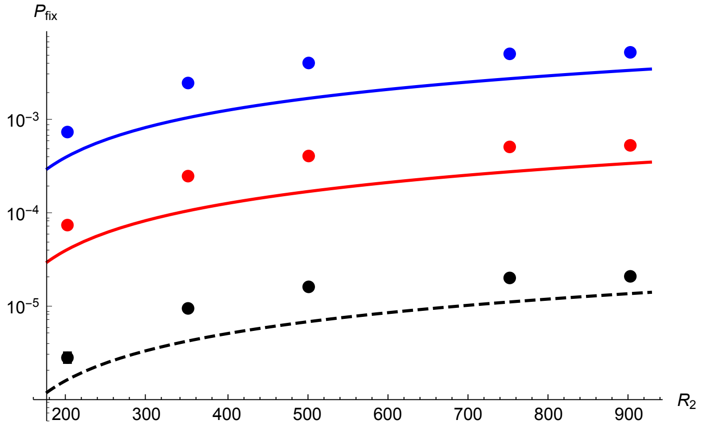

K = 10,000 simulations, R2 < K

Below commands load simulation output files used in analysis.

```
dataK = Import["results_19Dec2014.dat", "Table"];
```

Redefining “YMaxN” and “YMaxGN” to find correct roots:

```
YMaxN[Ka_, R_, ρ_, x0_, y0_] :=
 y /. FindRoot[x0 + 1/ρ (Log[(y/y0)^R ((Ka - y)/(Ka - y0))^(Ka-R)]) == 0, {y, 2 R}]
```

```
YMaxGN[Ka_, R_, R2_, ρ_, x0_, y0_] :=
 Floor[y /. FindRoot[(1 + FX3[Ka, R, ρ, x0, y0, y]) (Ka - y) y ρ + R2 - y0 == 0, {y, Ka/4}]]
 (* Function to find when Π becomes inadmissable *)
```

$\rho = 0.5$

$\mu = 0.0001$

```
data1 = Transpose[Select[dataK, #[[4]] == 1.5 && #[[8]] == 0.0001 &]];
pts1 = Transpose[{data1[[3]], data1[[10]]}];
err1 = data1[[11]];
p1 = Show[LogPlot[
    {PEmerB2[10 000, 1000, R2, 0.5, 1, 20, 0.0001]}, {R2, 2800, 9200},
    PlotRange → Automatic, PlotStyle → {Black, Dashed}, AxesLabel → {"R2", "Pfix"}],
   ErrorListLogPlot[
    {{pts1[[1]], ErrorBar[err1[[1]]]}, {pts1[[2]], ErrorBar[err1[[2]]]},
     {pts1[[3]], ErrorBar[err1[[3]]]}, {pts1[[4]], ErrorBar[err1[[4]]]},
     {pts1[[5]], ErrorBar[err1[[5]]]}, {pts1[[6]], ErrorBar[err1[[6]]]}},
    PlotStyle → {Black, PointSize[0.02]}], PlotRange → All
 ]
```

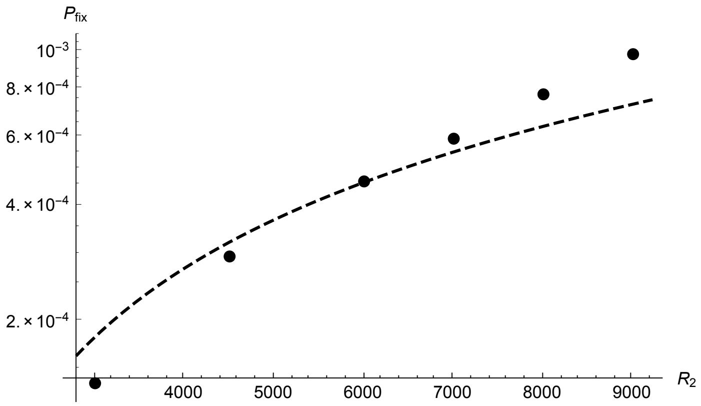

$\mu = 0.0025$

```
data1 = Transpose[Select[dataK, #[[4]] == 1.5 && #[[8]] == 0.0025 &]];
pts1 = Transpose[{data1[[3]], data1[[10]]}];
err1 = data1[[11]];
p2 = Show[LogPlot[
    {PEmerB2[10 000, 1000, R2, 0.5, 1, 20, 0.0025]}, {R2, 2800, 9200},
    PlotRange → {Automatic}, PlotStyle → {Red}, AxesLabel → {"R2", "Pfix"}],
  ErrorListLogPlot[
    {{pts1[[1]], ErrorBar[err1[[1]]]}, {pts1[[2]], ErrorBar[err1[[2]]]},
     {pts1[[3]], ErrorBar[err1[[3]]]}, {pts1[[4]], ErrorBar[err1[[4]]]},
     {pts1[[5]], ErrorBar[err1[[5]]]}, {pts1[[6]], ErrorBar[err1[[6]]]}},
    PlotStyle → {Red, PointSize[0.02]}], PlotRange → All
 ]
```

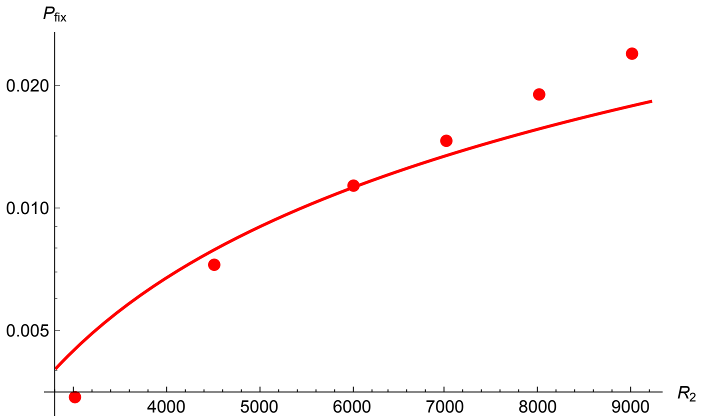

$\mu = 0.025$

```
data1 = Transpose[Select[dataK, #[[4]] == 1.5 && #[[8]] == 0.025 &]];
pts1 = Transpose[{data1[[3]], data1[[10]]}];
err1 = data1[[11]];
p3 = Show[LogPlot[
    {PEmerB2[10 000, 1000, R2, 0.5, 1, 20, 0.025]}, {R2, 2800, 9200},
    PlotRange → {Automatic}, PlotStyle → {Blue}, AxesLabel → {"R2", "Pfix"}],
  ErrorListLogPlot[
    {{pts1[[1]], ErrorBar[err1[[1]]]}, {pts1[[2]], ErrorBar[err1[[2]]]},
     {pts1[[3]], ErrorBar[err1[[3]]]}, {pts1[[4]], ErrorBar[err1[[4]]]},
     {pts1[[5]], ErrorBar[err1[[5]]]}, {pts1[[6]], ErrorBar[err1[[6]]]}},
    PlotStyle → {Blue, PointSize[0.02]}], PlotRange → All
 ]
```

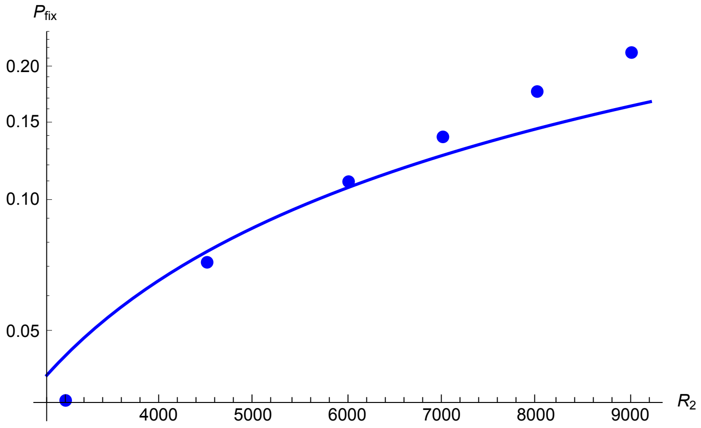

All μ together:

```
Show[p1, p2, p3, PlotRange → All]
```

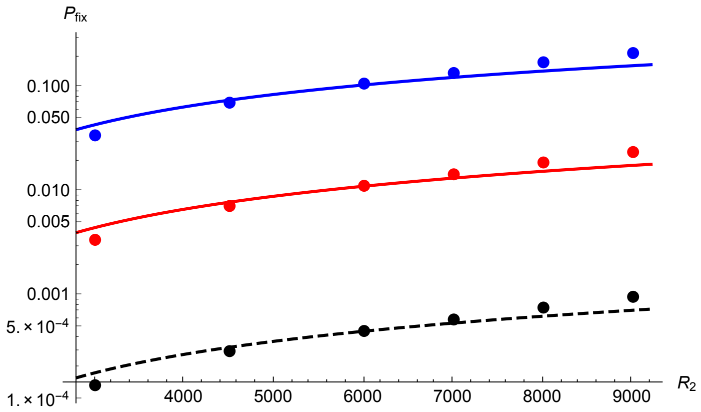

$\rho = 9$

$\mu = 0.0001$

```
data1 = Transpose[Select[dataK, #[[4]] == 10 && #[[8]] == 0.0001 &]];
pts1  = Transpose[{data1[[3]], data1[[10]]}];
err1 = data1[[11]];
p1 = Show[LogPlot[
    {PEmerB2[10 000, 1000, R2, 9, 1, 20, 0.0001]}, {R2, 2800, 9200},
    PlotRange → {Automatic},
    PlotStyle → {Black, Dashed}, AxesLabel → {"R2", "Pfix"}],
  ErrorListLogPlot[{{pts1[[1]], ErrorBar[err1[[1]]]},
     {pts1[[2]], ErrorBar[err1[[2]]]}, {pts1[[3]], ErrorBar[err1[[3]]]},
     {pts1[[4]], ErrorBar[err1[[4]]]}, {pts1[[5]], ErrorBar[err1[[5]]]},
     {pts1[[6]], ErrorBar[err1[[6]]]}},
    PlotStyle → {Black, PointSize[0.02]}], PlotRange → All
 ]
```

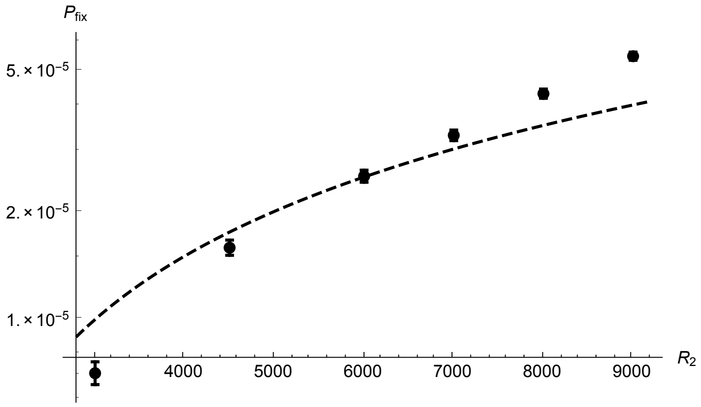

$\mu = 0.0025$

```
data1 = Transpose[Select[dataK, #[[4]] == 10 && #[[8]] == 0.0025 &]];
pts1 = Transpose[{data1[[3]], data1[[10]]}];
err1 = data1[[11]];
p2 = Show[LogPlot[
   {PEmerB2[10 000, 1000, R2, 9, 1, 20, 0.0025]}, {R2, 2800, 9200},
   PlotRange → {Automatic}, PlotStyle → {Red}, AxesLabel → {"R₂", "Pfix"}],
  ErrorListLogPlot[
   {{pts1[[1]], ErrorBar[err1[[1]]]}, {pts1[[2]], ErrorBar[err1[[2]]]},
    {pts1[[3]], ErrorBar[err1[[3]]]}, {pts1[[4]], ErrorBar[err1[[4]]]},
    {pts1[[5]], ErrorBar[err1[[5]]]}, {pts1[[6]], ErrorBar[err1[[6]]]}},
   PlotStyle → {Red, PointSize[0.02]}], PlotRange → All
 ]
```

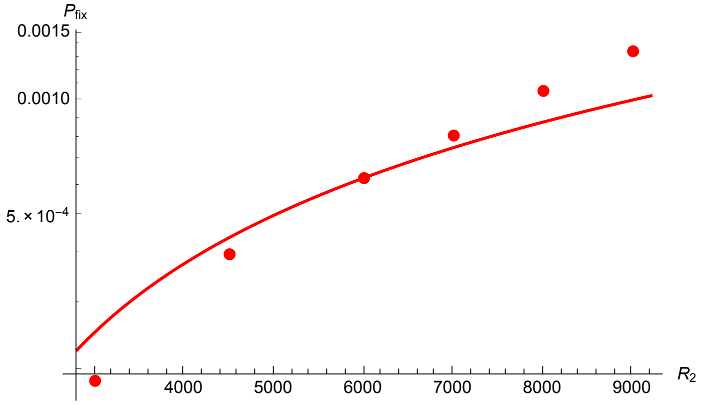

$\mu = 0.025$

```
data1 = Transpose[Select[dataK, #[[4]] == 10 && #[[8]] == 0.025 &]];
pts1 = Transpose[{data1[[3]], data1[[10]]}];
err1 = data1[[11]];
p3 = Show[LogPlot[
   {PEmerB2[10 000, 1000, R2, 9, 1, 20, 0.025]}, {R2, 2800, 9200},
   PlotRange → {Automatic}, PlotStyle → {Blue}, AxesLabel → {"R₂", "Pfix"}],
  ErrorListLogPlot[
   {{pts1[[1]], ErrorBar[err1[[1]]]}, {pts1[[2]], ErrorBar[err1[[2]]]},
    {pts1[[3]], ErrorBar[err1[[3]]]}, {pts1[[4]], ErrorBar[err1[[4]]]},
    {pts1[[5]], ErrorBar[err1[[5]]]}, {pts1[[6]], ErrorBar[err1[[6]]]}},
   PlotStyle → {Blue, PointSize[0.02]}], PlotRange → All
 ]
```

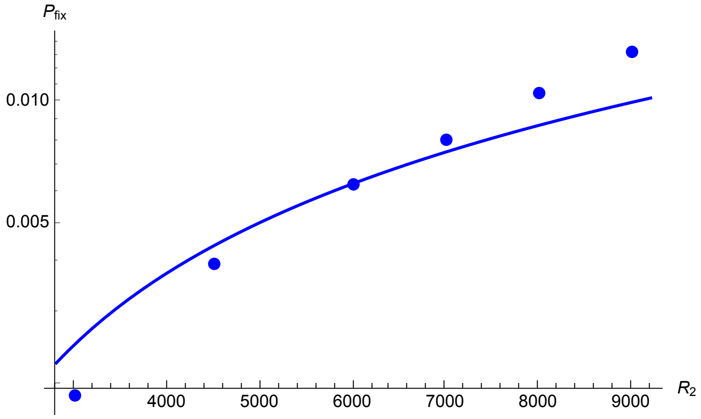

All μ together:

```
Show[p1, p2, p3]
```

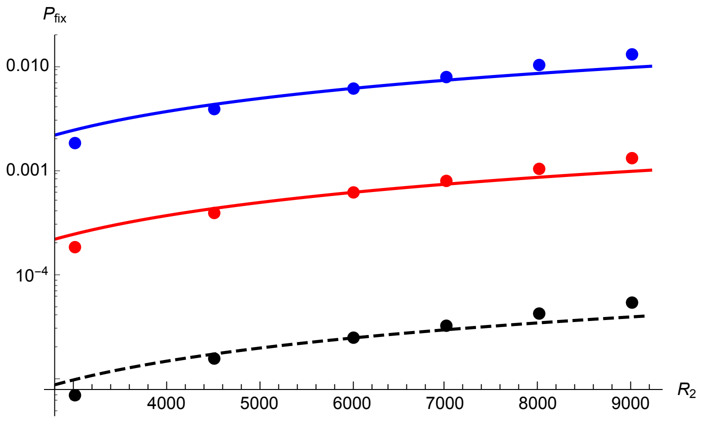

Supplementary Material S3: Mathematical analysis of analytical solution

Comparing models with and without feedbacks

1) Comparing this model to one not assuming feedbacks affecting emergence probability

First, we redefine the system of numerical equations to be solved, to create a solution using a ‘naive’ estimate of emergence probability.

```
YFixG1N[R2_] := (1 - 1/R2)

PEmerBN[Ka_, R_, R2_, ρ_, x0_, y0_, μ_, yM_] :=
 1 - Exp[-μ * NIntegrate[FX3[Ka, R, ρ, x0, y0, y] * YFixG1N[R2], {y, y0, yM}]]

PEmerBN2[Ka_, R_, R2_, ρ_, x0_, y0_, μ_] :=
 (1 - Exp[-2 (R - y0)/(R + y0)]) * PEmerBN[Ka, R, R2, ρ, x0, y0, μ, YMaxN[Ka, R, ρ, x0, y0]]
```

The ratio function below compares the current model estimate to the naive solution that does not consider epidemilogical feedbacks. Irrespective of the parameters used, one sees that the naive estimate (no feedbacks) greatly underestimates actual emergence probability.

```
Ratio[Ka_, R_, R2_, ρ_, x0_, y0_, μ_] := PEmerB2[Ka, R, R2, ρ, x0, y0, μ] / PEmerBN2[Ka, R, R2, ρ, x0, y0, μ]
```

```
Plot3D[Ratio[1000, 50, R2, 5, 1, 20, μ], {R2, 1100, 1200}, {μ, 0.000001, 0.001}]
```

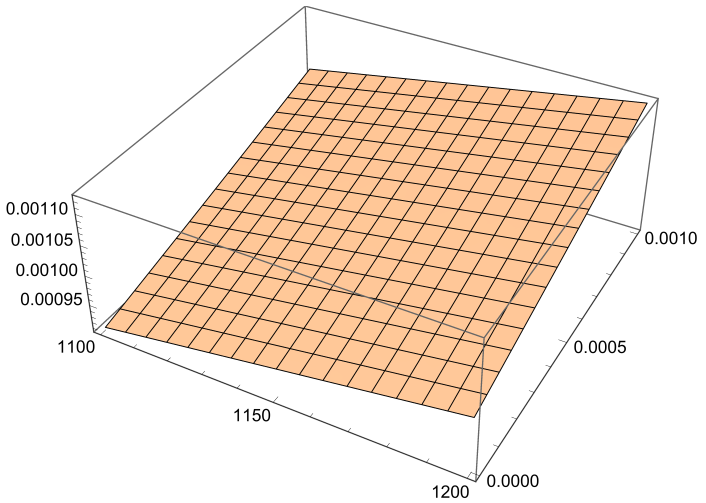


```
Plot3D[Ratio[100, 60, R2, 5, 1, 20, μ],
 {R2, 120, 200}, {μ, 0.000001, 0.001}, AxesLabel → Automatic]
```

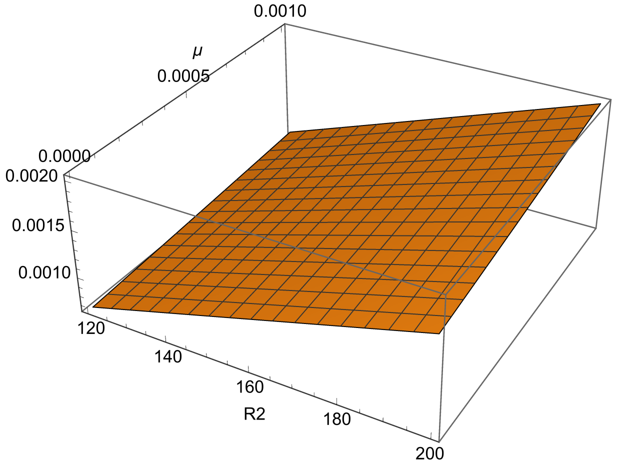

2) Comparing this model to one assuming feedbacks up to emergence only

Redefining the system of numerical equations to be solved, creating a solution that only considers feedbacks restricting emergence probability up to the point when the mutated parasite appears.

```
YFixG1M[R2_, y_] := (1 - Exp[- 2 (R2 - y) / (R2 + y)])

PEmerBM[Ka_, R_, R2_, ρ_, x0_, y0_, μ_, yM_] :=
 1 - Exp[-μ * NIntegrate[FX3[Ka, R, ρ, x0, y0, y] * YFixG1M[R2, y], {y, y0, yM}]]

YMaxG2M[Ka_, R_, R2_, ρ_, x0_, y0_] := Min[R2, YMaxN[Ka, R, ρ, x0, y0]]

PEmerBM2[Ka_, R_, R2_, ρ_, x0_, y0_, μ_] :=
 (1 - Exp[-2 (R - y0) / (R + y0)]) * PEmerBM[Ka, R, R2, ρ, x0, y0, μ, YMaxG2M[Ka, R, R2, ρ, x0, y0]]

Ratio2[Ka_, R_, R2_, ρ_, x0_, y0_, μ_] := PEmerB2[Ka, R, R2, ρ, x0, y0, μ] / PEmerBM2[Ka, R, R2, ρ, x0, y0, μ]
```

Again, irrespective of parameters used, this value still greatly underestimates the emergence probability accounting for immune feedbacks.

```
Plot3D[Ratio2[1000, 50, R2, 5, 1, 20, μ], {R2, 1100, 1200}, {μ, 0.000001, 0.001}]
```

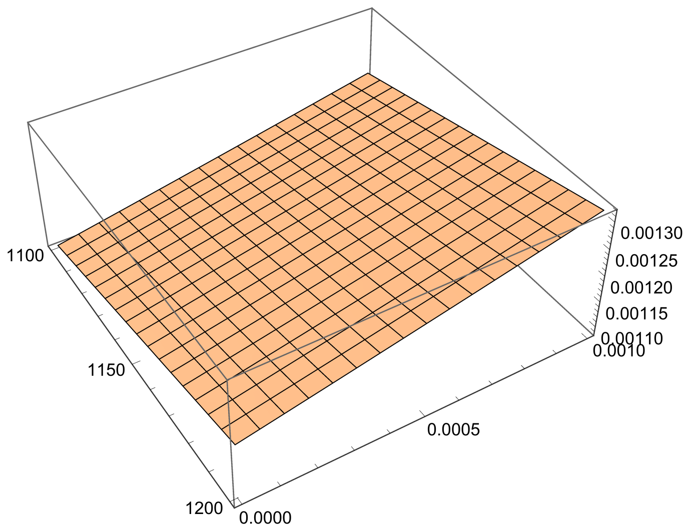

What effects emergence – increasing growth rate (φ) or tolerating immunity (increased σ)?

Scaling arguments of the initial model

Where there are two strains present, the unscaled form of dx2/dy is equal to:

$$\frac{x2\ (\phi - y\ \sigma)}{r\ (x1 + x2)\ \left(1 - \frac{y}{Ka}\right)\ y} \text{ // Simplify}$$

$$\frac{Ka\ x2\ (-y\ \sigma + \phi)}{r\ (x1 + x2)\ (Ka - y)\ y}$$

If we make the substitution $\phi \to \phi$ * c (so changing ϕ by a constant, c):

$$\frac{Ka\ x2\ (-y\ \sigma + \phi)}{r\ (x1 + x2)\ (Ka - y)\ y} \text{ /. } \{\phi \to \phi * c\}$$

$$\frac{Ka\ x2\ (-y\ \sigma + c\ \phi)}{r\ (x1 + x2)\ (Ka - y)\ y}$$

Then making the further substitutions for R, ρ:

$$\frac{Ka\ x2\ (-y\ \sigma + c\ \phi)}{r\ (x1 + x2)\ (Ka - y)\ y} \text{ /. } \{\phi \to R\ \sigma\} \text{ // FullSimplify}$$

$$\frac{Ka\ x2\ (c\ R - y)\ \sigma}{r\ (x1 + x2)\ (Ka - y)\ y}$$

```
(Ka x2 (c R - y) σ)/(r (x1 + x2) (Ka - y) y) /. {r → ρ σ1} // FullSimplify
```

$$\frac{\text{Ka x2 (c R - y) } \sigma}{\text{(x1 + x2) (Ka - y) y } \rho \, \sigma 1}$$

We can re-write this form as:

```
(Ka x2 (c R - y))/((x1 + x2) (Ka - y) y ρ) (σ/σ1) - {(Ka x2 (c R - y) σ)/((x1 + x2) (Ka - y) y ρ σ1)} // Simplify
```

{0}

Alternatively, one can instead scale $\sigma \to \sigma$ / c, and make substitutions accordingly:

```
(Ka x2 (-y σ + ϕ))/(r (x1 + x2) (Ka - y) y) /. {σ → σ / c}
```

$$\frac{\text{Ka x2} \left(-\frac{y\sigma}{c} + \phi\right)}{\text{r (x1 + x2) (Ka - y) y}}$$

```
(Ka x2 (-(y σ)/c + ϕ))/(r (x1 + x2) (Ka - y) y) /. {ϕ → R σ} // FullSimplify
```

$$\frac{\text{Ka x2} \left(R - \frac{y}{c}\right) \sigma}{\text{r (x1 + x2) (Ka - y) y}}$$

```
(Ka x2 (R - y/c) σ)/(r (x1 + x2) (Ka - y) y) /. {r → ρ σ1} // FullSimplify
```

$$\frac{\text{Ka x2} \left(R - \frac{y}{c}\right) \sigma}{\text{(x1 + x2) (Ka - y) y } \rho \, \sigma 1}$$

This scaled form can be rewritten as:

```
(Ka x2 (c R - y))/((x1 + x2) (Ka - y) y ρ c) (σ/σ1) - {(Ka x2 (R - y/c) σ)/((x1 + x2) (Ka - y) y ρ σ1)} // Simplify
```

{0}

As explained in the main manuscript, these scaling arguments show how scaling down σ appears to have a larger negative impact on emergence probability (assuming $\sigma = \sigma 1$). Therefore it would be more beneficial for the growth rate φ to be increased instead. This intution is verified in the following numerical plots below.

```
YMaxGN[Ka_, R_, R2_, ρ_, x0_, y0_] :=
 Floor[y /. FindRoot[(1 + FX3[Ka, R, ρ, x0, y0, y]) (Ka - y) y ρ + R2 == 0, {y, 2 * R}]]
```

```
YMaxN[Ka_, R_, ρ_, x0_, y0_] :=
 y /. FindRoot[x0 + 1/ρ (Log[(y/y0)^R ((Ka - y)/(Ka - y0))^(Ka-R)]) == 0, {y, 2 * R}]
```

```
Plot3D[PEmerB2[1000, 100, R2 * 1.01, 1.01 * ρ, 1, 20, 0.025] /
  PEmerB2[1000, 100, R2 * 1.01, ρ, 1, 20, 0.025],
 {ρ, 0.5, 15}, {R2, 1100, 1200}, AxesLabel → Automatic]
```

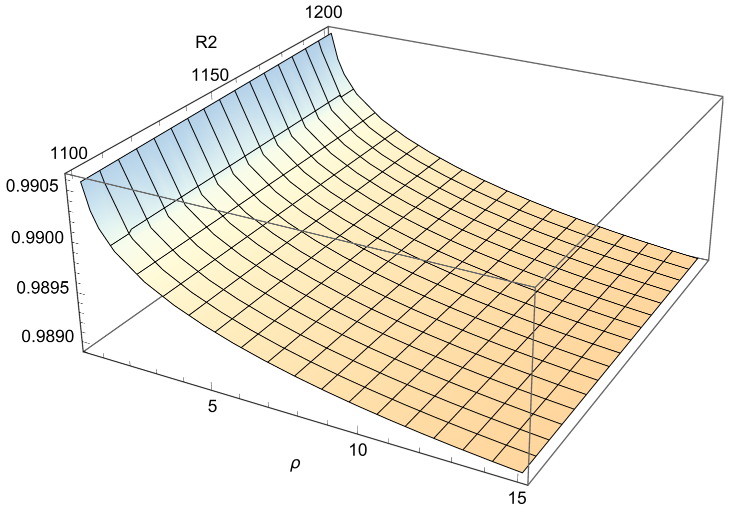


```
Plot3D[PEmerB2[1000, 100, R2 * 1, 1.01 * ρ, 1, 20, 0.025] /
  PEmerB2[1000, 100, R2 * 1, ρ, 1, 20, 0.025],
 {ρ, 0.5, 15}, {R2, 1100, 1200}, AxesLabel → Automatic]
```

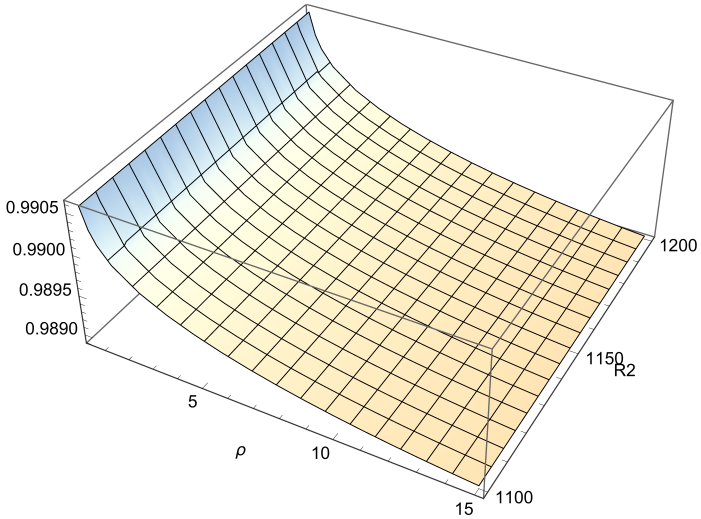

```
Plot3D[PEmerB2[1000, 60, R2, 0.984 * ρ, 1, 20, 0.0025] /
  PEmerB2[1000, 62, R2, ρ, 1, 20, 0.0025], {ρ, 3, 10}, {R2, 1100, 1200}]
```

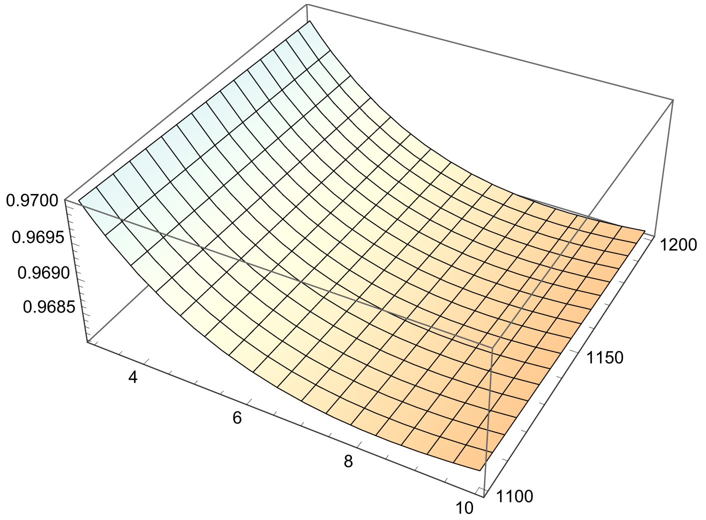

Using the immune response from Alizon 2008

We can repeat the above analysis, but instead on an equation that has the following form for dy/dt = r ϕ x y (1-$\frac{y}{K}$). This is the more general immune-response form used in Alizon 2008, assuming that immune proliferation is not just dependent on the presence of a pathogen, but also on its growth rate (see main text for the rationale behind this function).

By increasing φ by a constant c, we obtain the following formula:

$$\frac{\text{x2}\ (\phi - y\,\sigma)}{r\,\phi\ (\text{x1} + \text{x2})\ \left(1 - \frac{y}{\text{Ka}}\right)\ y} \ \text{// Simplify}$$

$$\frac{\text{Ka}\ \text{x2}\ (-y\,\sigma + \phi)}{r\ (\text{x1} + \text{x2})\ (\text{Ka} - y)\ y\ \phi}$$

$$\frac{\text{Ka}\ \text{x2}\ (-y\,\sigma + \phi)}{r\ (\text{x1} + \text{x2})\ (\text{Ka} - y)\ y\ \phi} \ \text{/. } \{\phi \to \phi * c\}$$

$$\frac{\text{Ka}\ \text{x2}\ (-y\,\sigma + c\,\phi)}{c\ r\ (\text{x1} + \text{x2})\ (\text{Ka} - y)\ y\ \phi}$$

$$\frac{\text{Ka}\ \text{x2}\ (-y\,\sigma + c\,\phi)}{c\ r\ (\text{x1} + \text{x2})\ (\text{Ka} - y)\ y\ \phi} \ \text{/. } \{\phi \to R\,\sigma\}\ \text{// FullSimplify}$$

$$\frac{\text{Ka}\ \text{x2}\ (c\,R - y)}{c\ r\ R\ (\text{x1} + \text{x2})\ (\text{Ka} - y)\ y}$$

$$\frac{\text{Ka}\ \text{x2}\ (c\,R - y)}{c\ r\ R\ (\text{x1} + \text{x2})\ (\text{Ka} - y)\ y} \ \text{/. } \{r \to \rho\,\sigma 1\}\ \text{// FullSimplify}$$

$$\frac{\text{Ka}\ \text{x2}\ (c\,R - y)}{c\ R\ (\text{x1} + \text{x2})\ (\text{Ka} - y)\ y\ \rho\ \sigma 1}$$

We can rewrite this as, giving Equation 14 in the main text:

$$\frac{\text{Ka}\ (\text{c R}-\text{y})}{\text{c}\ \rho\ \text{R y}\ (\text{Ka}-\text{y})\ \sigma 1}\left(\frac{\text{x2}}{\text{x1}+\text{x2}}\right)-\left\{\frac{\text{Ka x2}\ (\text{c R}-\text{y})}{\text{c R}\ (\text{x1}+\text{x2})\ (\text{Ka}-\text{y})\ \text{y}\ \rho\ \sigma 1}\right\}\ //\ \text{Simplify}$$

{0}

Alternatively, we can scale $\sigma \to \sigma/c$ as before:

$$\frac{\text{Ka x2}\ (-\text{y}\ \sigma+\phi)}{\text{r}\ (\text{x1}+\text{x2})\ (\text{Ka}-\text{y})\ \text{y}\ \phi}\ /.\ \{\sigma \to \sigma / \text{c}\}$$

$$\frac{\text{Ka x2}\ \left(-\frac{\text{y}\ \sigma}{\text{c}}+\phi\right)}{\text{r}\ (\text{x1}+\text{x2})\ (\text{Ka}-\text{y})\ \text{y}\ \phi}$$

$$\frac{\text{Ka x2}\ \left(-\frac{\text{y}\ \sigma}{\text{c}}+\phi\right)}{\text{r}\ (\text{x1}+\text{x2})\ (\text{Ka}-\text{y})\ \text{y}\ \phi}\ /.\ \{\phi \to \text{R}\ \sigma\}\ //\ \text{FullSimplify}$$

$$\frac{\text{Ka x2}\ (\text{c R}-\text{y})}{\text{c r R}\ (\text{x1}+\text{x2})\ (\text{Ka}-\text{y})\ \text{y}}$$

$$\frac{\text{Ka x2}\ (\text{c R}-\text{y})}{\text{c r R}\ (\text{x1}+\text{x2})\ (\text{Ka}-\text{y})\ \text{y}}\ /.\ \{\text{r} \to \rho\ \sigma 1\}\ //\ \text{FullSimplify}$$

$$\frac{\text{Ka x2}\ (\text{c R}-\text{y})}{\text{c R}\ (\text{x1}+\text{x2})\ (\text{Ka}-\text{y})\ \text{y}\ \rho\ \sigma 1}$$

After simplifying, we see that this term is also equal to Equation 14 in the main text:

$$\frac{\text{Ka}\ (\text{c R}-\text{y})}{\text{c}\ \rho\ \text{R y}\ (\text{Ka}-\text{y})\ \sigma 1}\left(\frac{\text{x2}}{\text{x1}+\text{x2}}\right)-\left\{\frac{\text{Ka x2}\ (\text{c R}-\text{y})}{\text{c R}\ (\text{x1}+\text{x2})\ (\text{Ka}-\text{y})\ \text{y}\ \rho\ \sigma 1}\right\}\ //\ \text{Simplify}$$

To test whether this alternative immune response will lead to different outcomes concerning increasing growth against tolerance, we rederive the system of equations as above, but using Alizon's (2008) immune function instead.

First, we rederive x[y] by solving dx/dy when there is one strain:

$$\text{DSolve}\left[\left\{\text{x}'[\text{y}] == \frac{\text{Ka}\ (\text{R}-\text{y})}{\text{R}\ (\text{Ka}-\text{y})\ \text{y}\ \rho\ \sigma},\ \text{x}[\text{y0}] == \text{x0}\right\},\ \text{x}[\text{y}],\ \text{y}\right]\ //\ \text{Simplify}$$

$$\left\{\left\{\text{x}[\text{y}] \to \frac{1}{\text{R}\ \rho\ \sigma}(\text{R x0}\ \rho\ \sigma+\text{R Log}[\text{y}]+(\text{Ka}-\text{R})\ \text{Log}[-\text{Ka}+\text{y}]-\text{R Log}[\text{y0}]-\text{Ka Log}[-\text{Ka}+\text{y0}]+\text{R Log}[-\text{Ka}+\text{y0}])\right\}\right\}$$

This long solution can be simplifed to:

$$\text{x0}+\frac{1}{\text{R}\ \rho\ \sigma}\left(\text{Log}\left[\left(\frac{\text{y}}{\text{y0}}\right)^{\text{R}}\left(\frac{\text{Ka}-\text{y}}{\text{Ka}-\text{y0}}\right)^{\text{Ka}-\text{R}}\right]\right)$$

We check this by differentiating it and noting that it is equivalent to the original differential equation term:

$$\text{D}\left[\text{x0}+\frac{1}{\text{R}\ \rho\ \sigma}\left(\text{Log}\left[\left(\frac{\text{y}}{\text{y0}}\right)^{\text{R}}\left(\frac{\text{Ka}-\text{y}}{\text{Ka}-\text{y0}}\right)^{\text{Ka}-\text{R}}\right]\right),\ \text{y}\right]\ //\ \text{FullSimplify}$$

$$\frac{\text{Ka R}-\text{Ka y}}{\text{Ka R y}\ \rho\ \sigma-\text{R y}^2\ \rho\ \sigma}-\left\{\frac{\text{Ka}\ (\text{R}-\text{y})}{\text{R}\ (\text{Ka}-\text{y})\ \text{y}\ \rho\ \sigma}\right\}\ //\ \text{Simplify}$$

{0}

Therefore, rewriting the system of equations as follows:

```
FX3a[Ka_, R_, ρ_, σ_, x0_, y0_, y_] := x0 + 1/(R ρ σ) (Log[(y/y0)^R ((Ka - y)/(Ka - y0))^(Ka-R)])

YFixG1X[Ka_, R_, R2_, ρ_, σ_, x0_, y0_, y_] :=
 ((R2 - y0) / ((1 + FX3a[Ka, R, ρ, σ, x0, y0, y]) (Ka - y) R y ρ σ + R2 - y0))
  (1 - Exp[- (2 (R2 - y))/(R2 + y)])

YMaxGN[Ka_, R_, R2_, ρ_, σ_, x0_, y0_] := Floor[
  y /. FindRoot[(1 + FX3a[Ka, R, ρ, σ, x0, y0, y]) (Ka - y) R y ρ σ + R2 - y0 == 0, {y, (2 Ka)/3}]]

YMaxN[Ka_, R_, ρ_, σ_, x0_, y0_] :=
 y /. FindRoot[(x0 + 1/(R ρ σ) (Log[(y/y0)^R ((Ka - y)/(Ka - y0))^(Ka-R)])) == 0, {y, (2 Ka)/3}]

YMaxG2[Ka_, R_, R2_, ρ_, σ_, x0_, y0_] :=
 Min[YMaxGN[Ka, R, R2, ρ, σ, x0, y0], YMaxN[Ka, R, ρ, σ, x0, y0]]

PEmerB[Ka_, R_, R2_, ρ_, σ_, x0_, y0_, μ_, yM_] :=
 1 - Exp[-μ * NIntegrate[FX3a[Ka, R, ρ, σ, x0, y0, y] *
      YFixG1X[Ka, R, R2, ρ, σ, x0, y0, y], {y, y0, yM}]]

PEmerB2[Ka_, R_, R2_, ρ_, σ_, x0_, y0_, μ_] := (1 - Exp[-2 (R - y0)/(R + y0)]) *
  PEmerB[Ka, R, R2, ρ, σ, x0, y0, μ, YMaxG2[Ka, R, R2, ρ, σ, x0, y0]]
```

Comparing increased tolerance against increase growth shows that increased growth performs better in terms of emergence probability, as explained in the main text.

```
Plot3D[PEmerB2[1000, 100, R2 * 1.01, 1.01 * ρ, 1, 1, 20, 0.025] / PEmerB2[1000, 100, R2 * 1.01, ρ, 1, 1, 20, 0.025],
 {ρ, 2, 15}, {R2, 1100, 1200}, AxesLabel → Automatic]
```

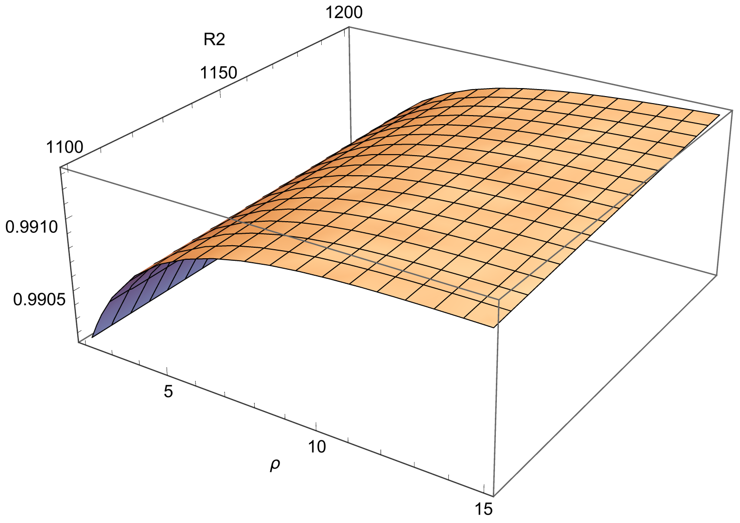

Bibliography

- Alizon S and van Baalen, M. "Acute or Chronic? Within–Host Models with Immune Dynamics, Infection Outcome, and Parasite Evolution." Am Nat. 2008; **172**(6): E244–E256.

- Alizon S “Transmission–Recovery Trade–Offs to Study Parasite Evolution.” Am Nat. 2008; **172**(3): E113--E121.
